# Conjugated 1,8 and 1,6 Addition of Bis*-*Trimethylsilylketene Acetal to Activated *p*-Quinone Methides via Trifluoromethanesulfonic Anhydride

**DOI:** 10.1021/acs.joc.4c02852

**Published:** 2025-03-31

**Authors:** Luis J. Benitez-Puebla, Ricardo Ballinas-Indili, Marcos Flores-Alamo, José M. Guevara-Vela, Tomas Rocha-Rinza, Saulo Rosales-Amezcua, Cecilio Alvarez-Toledano

**Affiliations:** † Universidad Nacional Autónoma de México, Instituto de Química, Circuito Exterior, Ciudad Universitaria, Alcaldía Coyoacán, Ciudad de Mexico 04510, México; ‡ Departamento de Ciencias Químicas, Facultad de Estudios Superiores Cuautitlán-UNAM, Campo 1, Avenida 1ro. de mayo s/n, Cuautitlán Izcalli, Estado de México C.P 54740, México; § Universidad Nacional Autónoma de México, Unidad de Servicios y Apoyo a la Investigación e Industria (USAII), Edificio H Mario Molina, Circuito Mario de la Cueva, Esquina circuito de la investigación Científica, Coyoacán, Ciudad de Mexico CU 04510, México; ∥ Departamento de Química Física Aplicada, Universidad Autónoma de Madrid, Madrid 28049, Spain

## Abstract

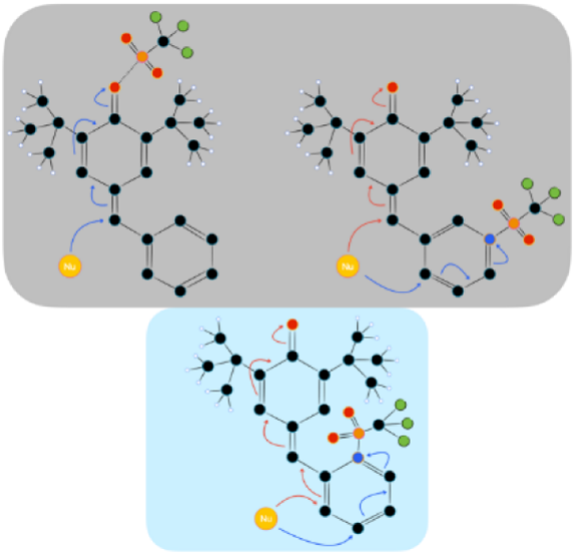

In this work, we studied the conjugated additions of bis-trimethylsilylacetalketene acetals (bis-TMSKA) to *para*-quinone methides (*p*-QMs), which are one of the most explored molecules for the study of conjugated additions and gained significant attention in organic chemistry due to their versatile reactivity, particularly in Michael addition reactions. In this study, trifluoromethanesulfonic anhydride (Tf_2_O) was used as an activating agent for *p*-QMs, aiming to achieve 1,6-Michael addition products and the least reported 1,8-Michael addition with pyridine substituents. The reactivity of *p*-QMs derived from pyridine demonstrated distinct reaction pathways, leading to the formation of δ and γ lactones. The investigation also involved synthesizing a 1-indanone derived from the carboxylic acids obtained from the 1,6-addition.

## Introduction

The synthesis of molecules with high synthetic value has garnered significant attention in the pursuit of new methods to create complex organic compounds; *p*-QMs have emerged as a promising class of compounds due to their ability to engage in a wide range of reactions, including Michael reactions. In the literature, numerous reports of Michael-type conjugate additions to *p*-QMs can be found, with a particular focus on 1,6 additions^1−5^ and asymmetric reactions.^6−9^ The presence of a conjugated diene motif confers exceptional versatility to these molecules, thereby rendering them excellent building blocks for synthesis. The current approach consists of the activation of *p*-QMs under a wide array of conditions,^5−11^ including metal- and Lewis-Brønsted acid-based,^12−16^ phosphine-mediated catalysis,^17^ organocatalysis,^6,18^ and light-induced activation.^19−21^

Michael addition reactions are particularly useful for the formation of new carbon–carbon bonds. The reaction of *p*-QMs through Michael additions to either γ,δ-unsaturated systems or the relatively less explored ε,ζ-unsaturated counterparts is a potent methodology for the synthesis of complex organic molecules.^2,22−35^ These synthetic scaffolds hold immense promise for diverse applications in areas such as biologically active compounds, materials science, and natural product synthesis.^2,36^

**Figure 1:**
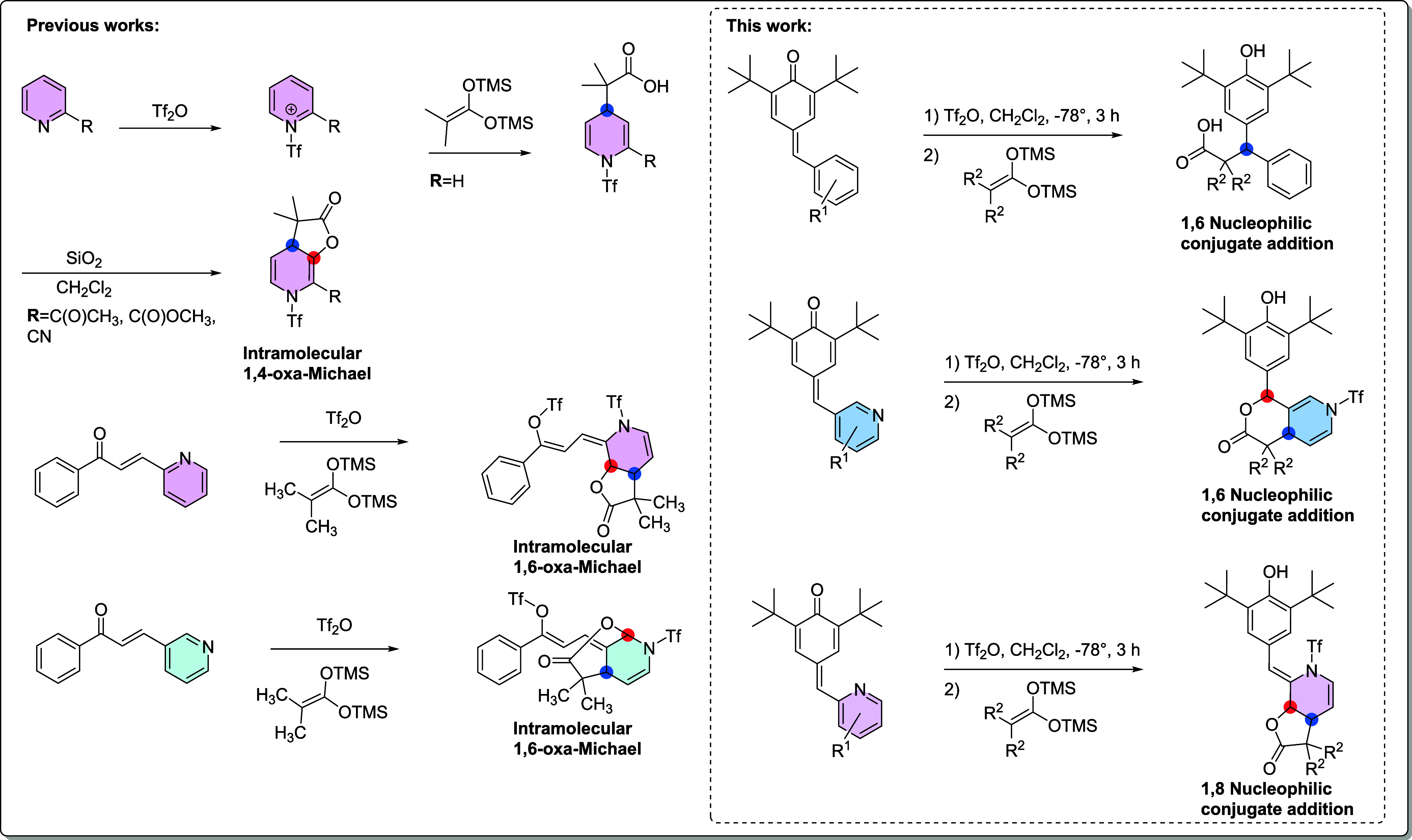
Dihydropyridine synthesis through Tf_2_O activation.

In addition to Michael’s additions, the activation of pyridines using trifluoromethanesulfonic anhydride has been extensively investigated in scientific research. Other activation strategies have also been explored, including *N*-acylation, *N*-imidate salts, *N*-alkylation, *N*-oxides, *N*-acyliminium, and transition metal-mediated functionalization.^37−39^ The research group studied these activations involving pyridine as the substrate, which subsequently underwent nucleophilic attack by a bis-TMSKA (Figure 1). Notably, the preferential site of nucleophilic attack was the 4-position of the pyridine ring, leading to the formation of a dihydropyridine moiety.^40^

As shown in Figure 1,^41^ pyridines bearing a substituent in the 2-position, specifically electron-withdrawing groups, were subjected to similar activation procedures. Remarkably, the outcomes of these studies consistently revealed that the 4-position remained the favored site for nucleophilic attack. Despite this common trend, an intriguing twist was observed during the compound purification process. Contrary to the anticipated dihydropyridine product, the final result presented a distinct molecular structure: a lactone formed through a 1,4-oxa-Michael.

Finally, another study involving chalcones with pyridine rings bonded in the β-position confirms that, following activation with trifluoromethanesulfonic anhydride, once again the first nucleophilic attack occurs at the 4-position. Subsequently, the cyclization to form lactone can take place either at the 2- or the 3- position, depending on the specific substrate.^42^

This additional information further supports the regioselectivity observed in our previous^42^ studies and highlights the versatility of the reaction. The ability of the reaction to proceed with multiple regiochemical outcomes opens up intriguing possibilities for the synthesis of diverse lactone derivatives, which could possess varying biological activities and applications. These cumulative findings demonstrate the complexity and significance of the trifluoromethanesulfonic anhydride activation method,^43^ providing valuable insights into its synthetic potential and serving as a foundation for the development of innovative methodologies in the field of organic chemistry.

Herein, we report the activation of *p*-QMs through the utilization of trifluoromethanesulfonic anhydride (Tf_2_O). The aim was to obtain products resulting from 1,6- and 1,8-Michael additions, where 1,6 additions were obtained with aromatic rings and pyridine-substituted *p*-QMs showed 1,8 additions. Upon the introduction of a pyridine substituent, we observed the formation of 1,8-Michael addition adducts, which represent a less common outcome. This unexpected finding highlights the influence of the pyridine moiety on the regioselectivity of the reaction and underscores the potential for accessing novel and unconventional Michael addition products. Furthermore, it highlights the vast potential for accessing a wide range of novel and unconventional Michael addition products by leveraging specific substituents in the reaction system.

## Results and Discussion

**Scheme 1:**
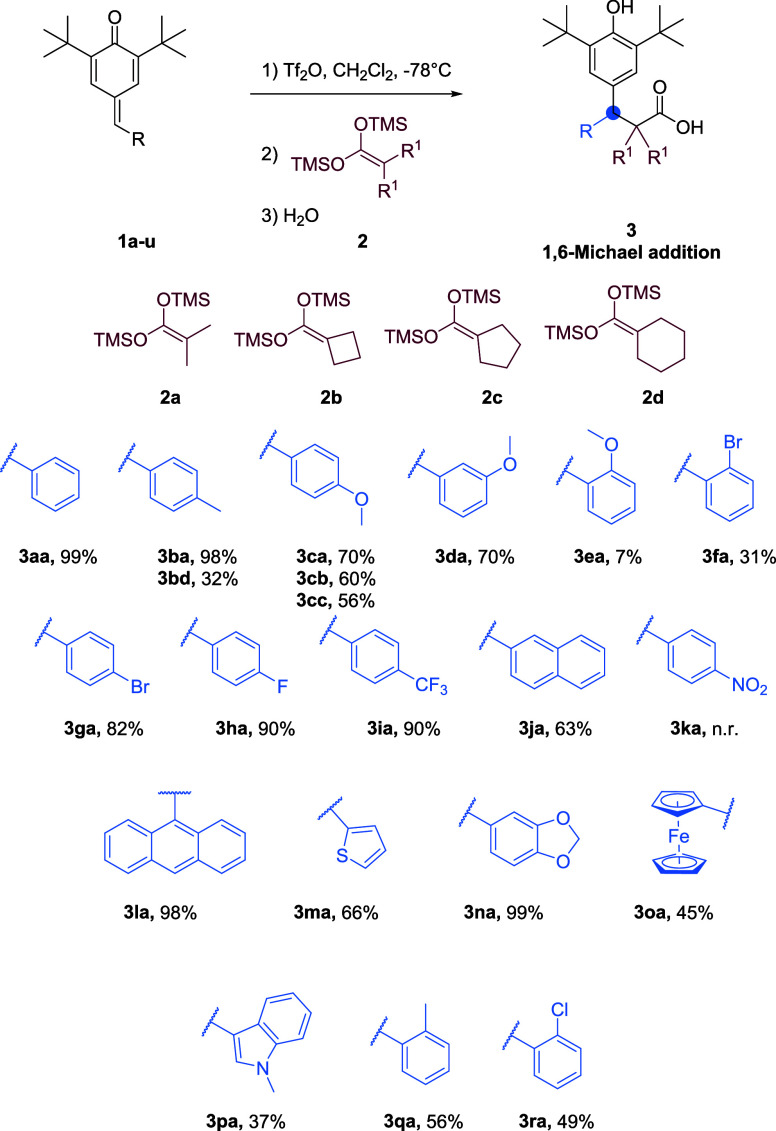
Synthesis and Isolated Yields of Carboxylic Acids via Michael 1,6-Addition, Reaction Conditions: (1) Tf_2_O 1.2 Equiv. 3 h, (2) 2 1.5 Equiv. 16 h, and (3) H_2_O

In the initial phase of our study, we embarked upon the synthesis of a diverse array of *p*-QMs under the reported conditions.^44^ Our primary objective revolved around the attainment of derivatives bearing a broad spectrum of functional groups (Scheme 1), including both electron-withdrawing (EWG) and electron-donating (EDG) moieties, as well as heterocycles. By incorporating such substituents, we aimed to investigate the impact of varying electronic and structural factors on the reactivity and subsequent transformations of these *p*-QM.

In Table 1, we conducted a series of experiments to identify the optimized reaction conditions by testing various activating agents. In our initial tests, we found that both TfOH and Cu(OTf)_2_ (entries 2–4, Table 1) exhibited lower yields compared to Tf_2_O, which was established as a more effective activating agent. Interestingly, BF_3_·OEt_2_ also yielded results that were similar to Tf_2_O. Notably, when we omitted the activating reagent altogether, we observed that no reaction occurred (entry 5, Table 1). Furthermore, we investigated the effect of increasing the amount of activating reagent. This adjustment had a slightly negative impact on the yield (entry 6, Table 1). For our solvent selection, we chose dichloromethane (DCM) due to its superior performance compared to toluene, which resulted in a significantly lower yield of only 10% (entry 7, Table 1). Finally, we assessed the impact of varying temperatures on the reaction. Our findings revealed that higher temperatures led to a noticeable decrease in yield, which dropped to 51% at elevated temperatures (entries 8 and 9, Table 1). Additionally, BF_3_·OEt_2_ demonstrated yields similar to those obtained with Tf_2_O when subjected to the same “high-temperature” conditions (Entry 10).

**Table 1:**
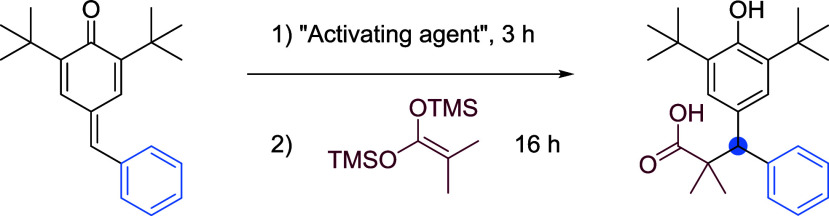
Optimization of the Reaction Conditions^a^

Entry	Activating agent (equiv.)	Solvent	Temperature (°C)	Yield (%)
1	Tf_2_O (1.2)	CH_2_Cl_2_	–78	99
2	TfOH (1.2)	CH_2_Cl_2_	–78	52
3	BF_3·_OEt_2_ (1.2)	CH_2_Cl_2_	–78	99
4	Cu(TfO)_2_ (1.2)	CH_2_Cl_2_	–78	81
5	–	CH_2_Cl_2_	–78	0
6	Tf_2_O (2.4)	CH_2_Cl_2_	–78	72
7	Tf_2_O (1.2)	Toluene	–78	10
8	Tf_2_O (1.2)	CH_2_Cl_2_	0	71
9	Tf_2_O (1.2)	CH_2_Cl_2_	25	51
10	BF_3·_OEt_2_ (1.2)	CH_2_Cl_2_	25	51

a All optimization reactions were carried out with 0.5 mmol of starting material and 5 mL of DCM.

With the optimized reaction conditions, we focused on the synthesis of carboxylic acids through a 1,6-Michael addition reaction. The procedure involved the activation of *p*-QM using trifluoromethanesulfonic anhydride for 3 h at a temperature of −78 °C. Subsequently, the corresponding bis-TMSKA was added to the reaction mixture. Encouragingly, most of the *p*-QMs exhibited high to moderate yields, indicating successful 1,6-addition reactions (Scheme 1).

**Figure 2:**
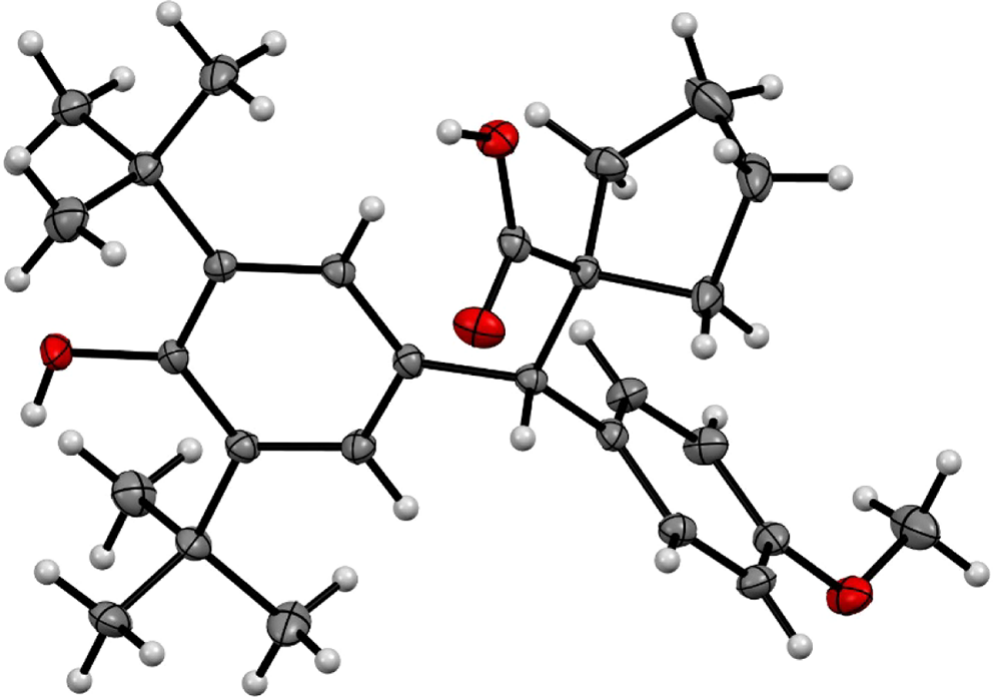
ORTEP diagram 50% probability for compound **3cc**.

The synthesis of carboxylic acids in compound **3ea** demonstrates a notable reduction in yield due to steric hindrance. In contrast, other *ortho*-substituted compounds featuring methyl and bromo substituents do not exhibit similar decreases in yield. Last, it is worth noting that a nitro substituent in the reaction system did not yield the desired product. Despite our efforts, the 1,6-Michael addition did not occur in this case. This observation suggests that the nitro group might exert an alternative way of activation, rendering it unproductive.^43^ An X-ray crystal structure was isolated from **3cc**, which is presented in Figure 2.

In contrast to the anticipated formation of carboxylic acids, the activation of *p*-QMs by trifluoromethanesulfonic anhydride (Tf_2_O) occurs through the pyridine ring. This activated pyridine ring possesses two electrophilic sites located at positions 4 and 2. Interestingly, both *p*-QMs derived from pyridine demonstrate that the first attack occurs at the 4 position. The reactivity of *p*-QMs derived from pyridinecarboxaldehyde, particularly those substituted at the 2 and 3 positions, demonstrates behavior analogous to that observed in our antecedent investigations, in which we obtained a γ-lactone through a 1,6-oxa-Michael addition (Figure 2), an identical behavior is observed in the present reaction. As for the reactions carried out with a pyridine substituted in the 2 position, a novel but expected 1,8-oxa-Michael addition is observed, and the pyridine ring present facilitates the observation of novel reactivity.

**Scheme 2:**
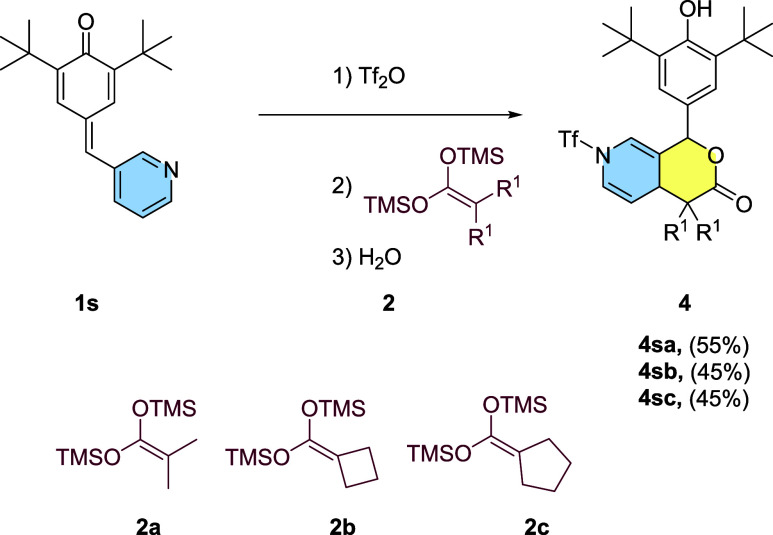
δ-lactone Synthesis, Reaction Conditions: (1) Tf_2_O 1.2 Equiv. 3 h, (2) 2 1.5 Equiv. 16 h, and (3) H_2_O

**Figure 3:**
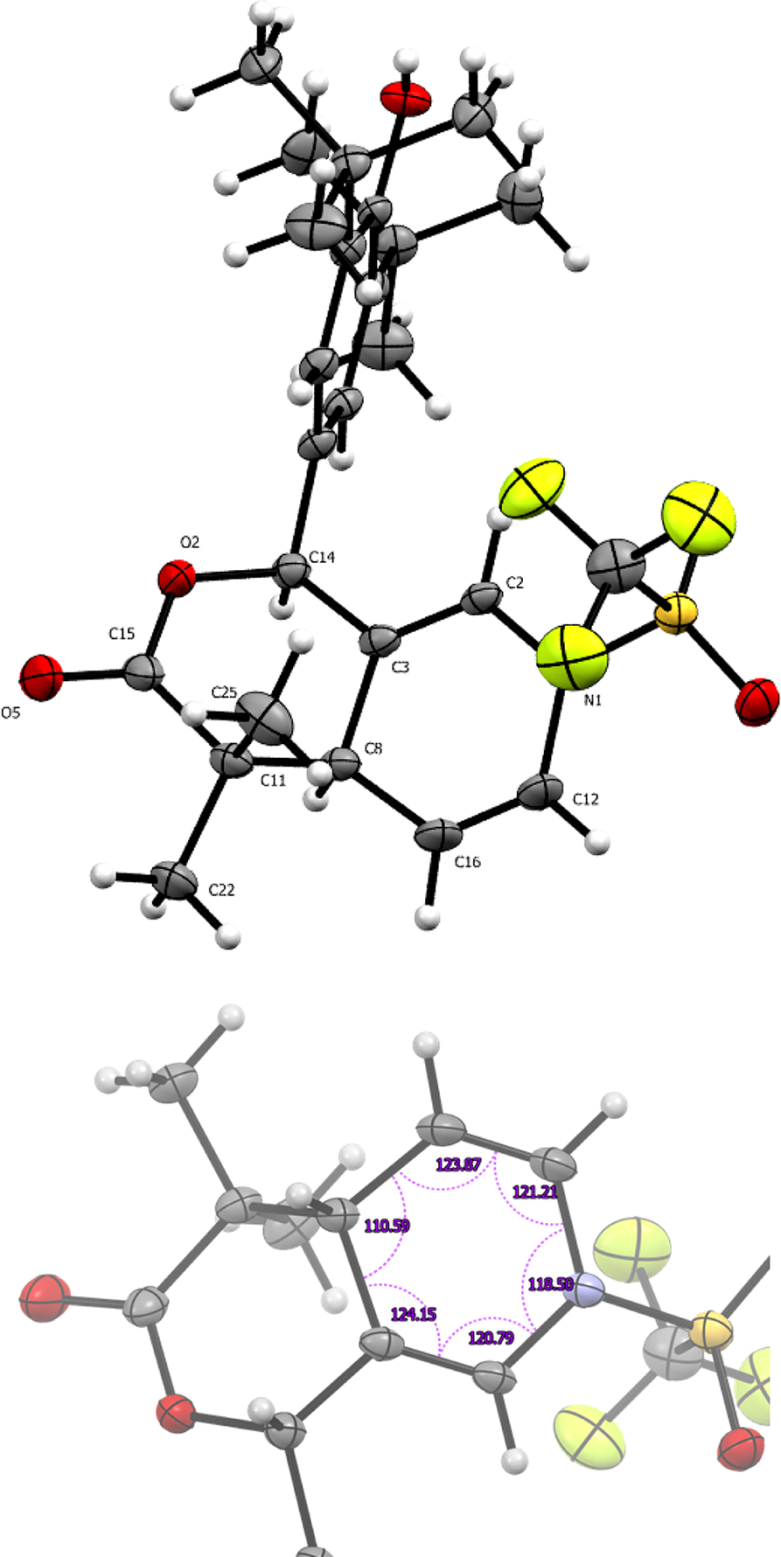
ORTEP diagram 50% probability for compound **4sa**.

However, the subsequent attack takes place differently for each compound. In the case of *p*-QM **1s**, the first attack leads to a reaction with the methine carbon, resulting in the formation of a δ-lactone (Scheme 2). We obtained X-ray data from several compounds; the data for compound **4s** is presented in Figure 3. As observed, the nitrogen of the former aromatic pyridine group bears a trifluoromethane sulfonic group. The loss of aromaticity due to the transformation into a dihydropyridine is clearly observed in the measured bond angles in the cycle. As observed, the sp^3^ carbon now shows a bond angle of 110.59°, which is characteristic of this hybridization. Double-bonded carbons show a 120–124° angle characteristic of an sp^2^ carbon (Figure 3).

**Scheme 3:**
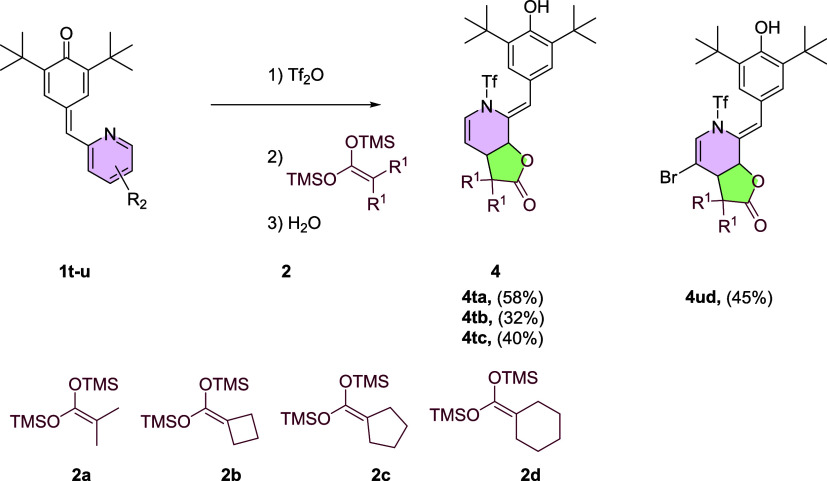
γ-lactone Synthesis, Reaction Conditions: (1) Tf_2_O 1.2 Equiv. 3 h, (2) 2 1.5 Equiv. 16 h, and (3) H_2_O

**Figure 4:**
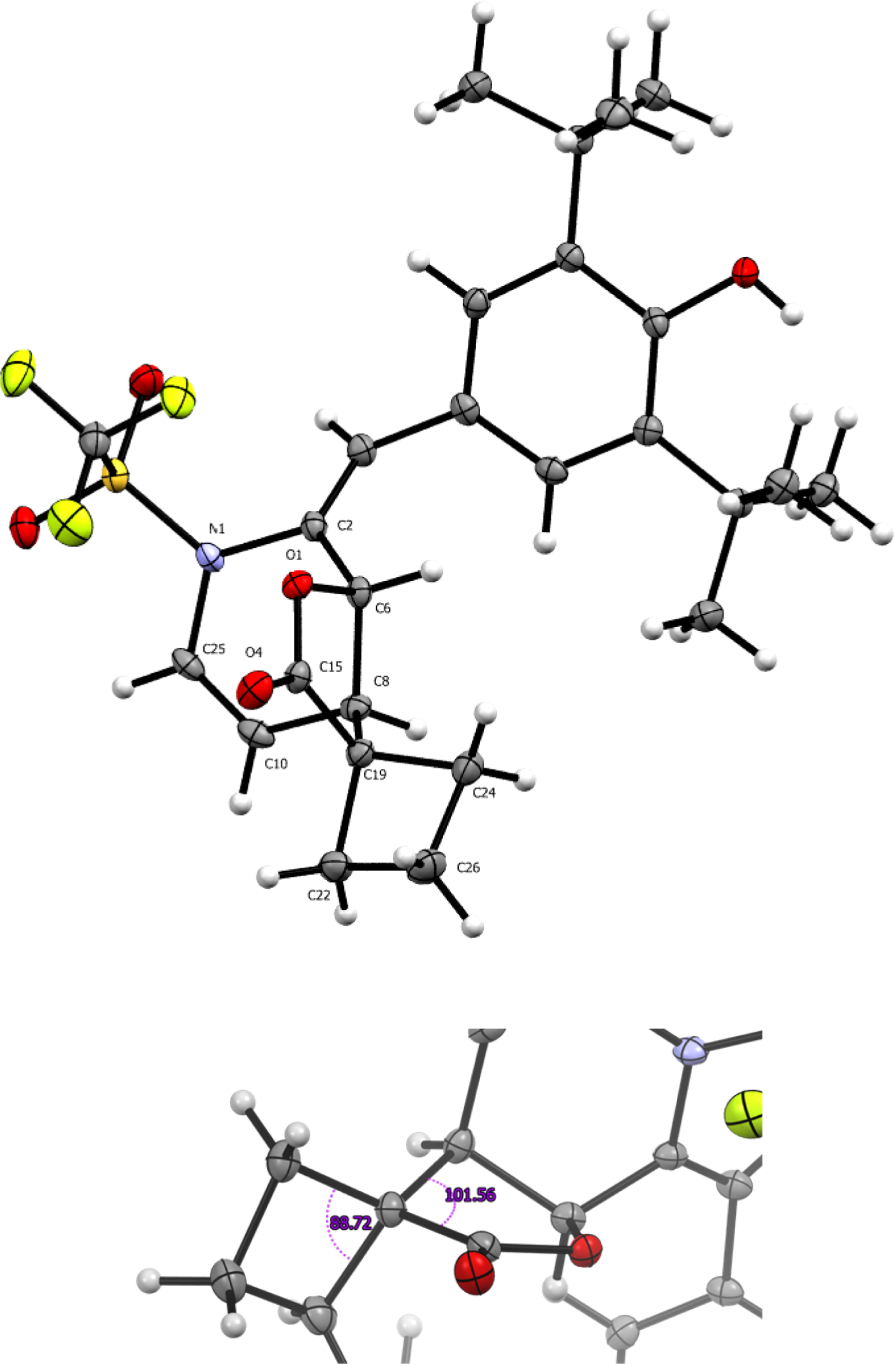
ORTEP diagram 50% probability for compound **4ta**.

On the other hand, *p*-QM **4t** yields γ lactone as a result of the second attack on position2 of the pyridine (Scheme 3). These distinct reaction pathways highlight the influence of the substituent position in the pyridine ring on the regioselectivity and product formation during the reactivity of *p*-QMs. A high-quality crystal was obtained from compound **4t**, revealing bond angles of 109.76° and 113.76° indicative of an sp^3^ carbon presence. Additionally, the exo-cyclic double bond displays a 112.25° angle due to ring torsion. The reaction with a bis-TMSKA derived from a cyclic carboxylic acid resulted in the formation of a noteworthy spiro compound featuring a five- and four-membered cycle ring, with the four-membered ring displaying an 88.7° internal angle and the five-membered ring exhibiting a 101.5° angle (Figure 4).

Understanding these divergent reactivity patterns provides valuable insights into the design of synthetic strategies and the development of novel compounds with specific structural motifs. The *tert*-butyl group’s bulkiness shields the phenol’s reactive site from direct interaction with Tf_2_O, preventing the anticipated O-triflation. Nonetheless, the *tert*-butyl group’s presence remains indispensable as it serves as an activation trigger for the substrate. Without the activating influence of Tf_2_O, the substrate remains unreactive, leading to unproductive outcomes in the reaction. Furthermore, the *tert*-butyl group strategically blocks the β position, acting as a protective barrier against undesired additions in this region. By doing so, it safeguards the molecule’s structural integrity and guides the reaction toward the desired product.

**Figure 5:**
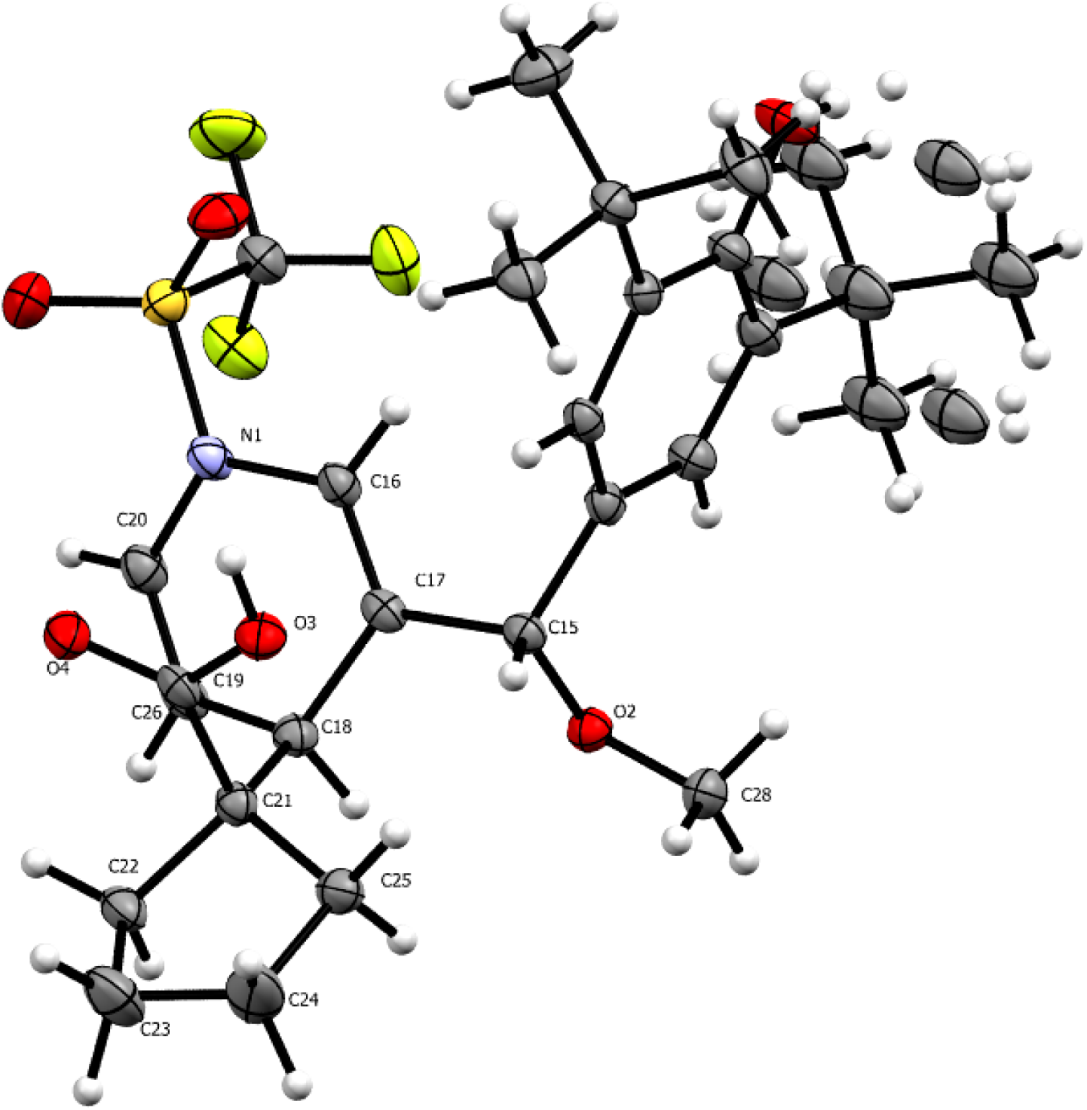
ORTEP diagram 50% probability for open lactone (**4tc-RO**) in methanol.

We successfully obtained a crystal from the open lactone through our efforts. In a methanol solution, lactones demonstrate an open structure, as evidenced in Figure 5. Methanol initiates a nucleophilic attack, leading to the opening of lactone (**4tc-RO**) and the subsequent formation of the corresponding carboxylic acid. Notably, this transformation occurred at an ambient temperature over a duration of 1 week.

**Scheme 4:**
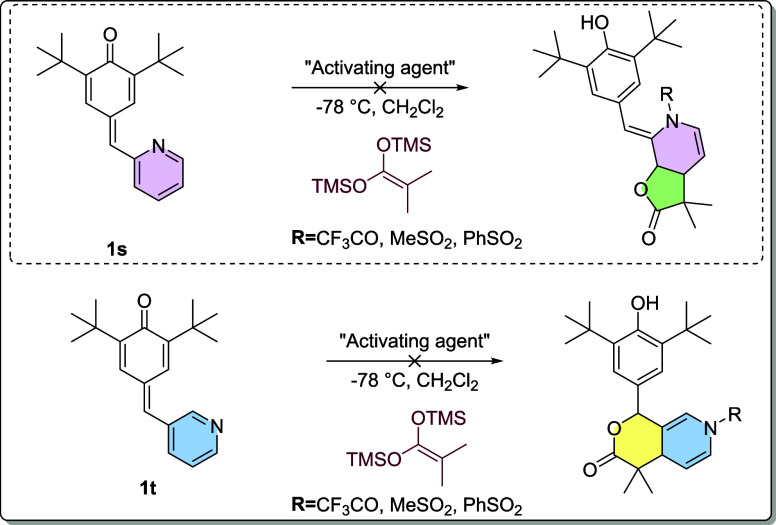
Assay of Reactivity with Other Activating Agents

We conducted further experiments to explore the potential of various activating agents in synthesizing lactone compounds (Scheme 4). Despite our efforts, these attempts did not yield any positive results. Specifically, we tested several agents, including trifluoromethanesulfonic anhydride, trifluoroacetic anhydride, and benzenesulfonic chloride, in hopes of facilitating the reaction. Unfortunately, rather than producing the anticipated lactone compounds, the reactions resulted in only the recovery of the starting materials. This outcome indicates that these activating agents may not be suitable for promoting the desired lactonization process.

## Theoretical Calculations

**Scheme 5:**
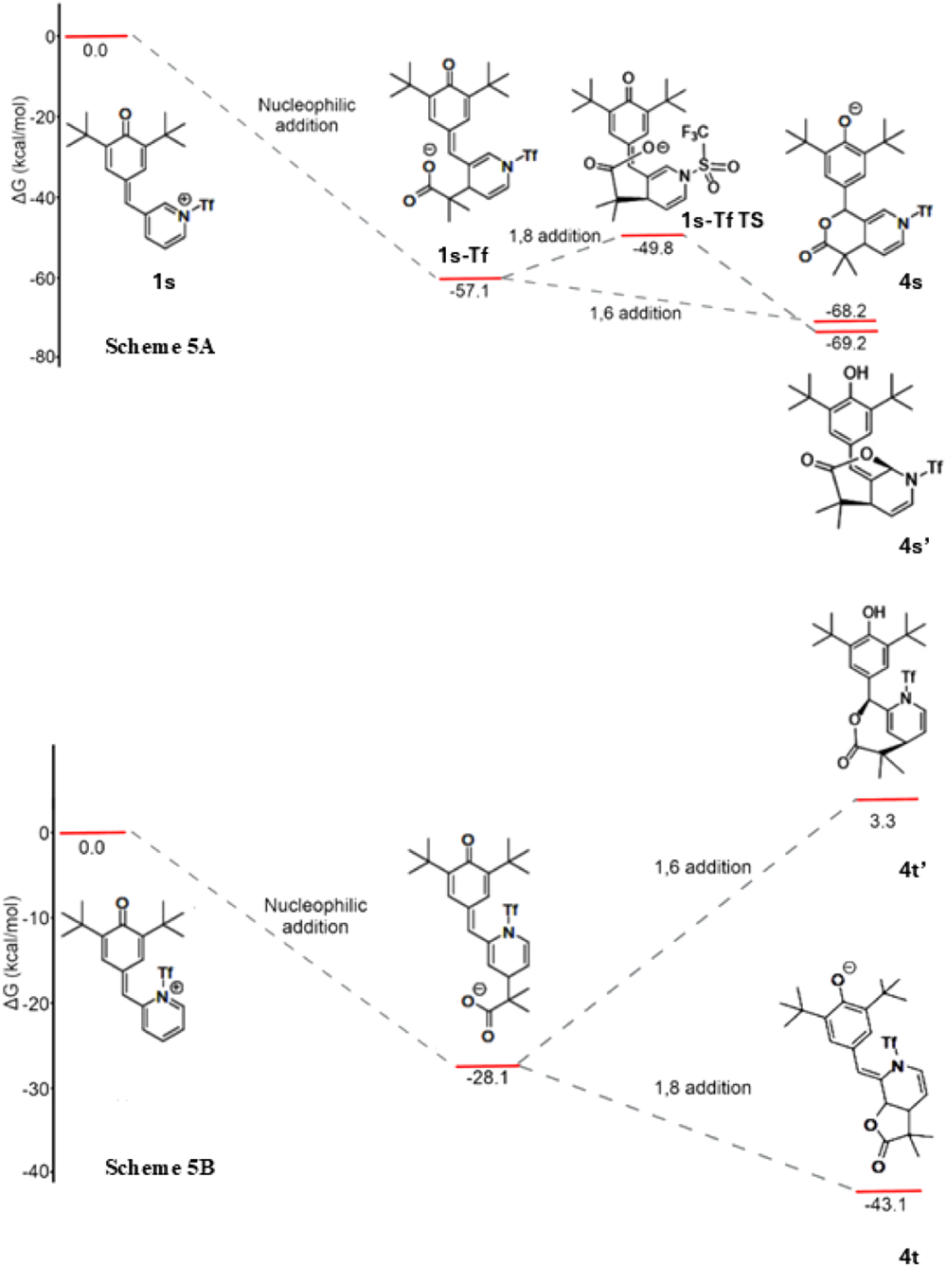
Gibbs Free Energy Profiles for the Reactions Displayed in Schemes 2 and 3 for the Substrates 1s and 1t, Respectively We started with the triflated species, **1s-Tf**, and then, we considered the coupling of **1n-Tf** and **1t-Tf** with the nucleophile ^–^:C(CH_3_)_2_-COO^–^. Afterward, we considered the 1,6 and 1,8 Michael additions for both systems. The diagrams show that the 1,6-addition for the compound **1n-Tf** is barrierless, as demonstrated in Scheme 5A. Similarly, the 1,8 addition for the compound 1t-Tf is barrierless, as shown in Scheme 5B.

We investigated the 1,6 and 1,8 Michael additions of the triflated systems **1s** and **1t** (Schemes 2 and 3) following the coupling of the nucleophile ^–^:C(CH_3_)_2_-COO^–^ to the para-position with respect to the pyridinic nitrogen, as indicated in Scheme 5. Once this nucleophile is bound to **1s-Tf** and **1t-Tf**, the organic substrates can undergo nucleophilic Michael additions. There is overall good agreement between the quantum chemical calculations and the experimental results discussed above. Namely, compound **1n** presents a barrierless Michael 1,6 addition, which results in a δ-lactone, as shown in the top part of Scheme 5. On the other hand, the corresponding 1,8 Michael addition involves a change of conformation, which entails an activation energy of 7.3 kcal/mol. Concerning the **1t-Tf** system (Scheme 5), it suffers a barrierless 1,8 Michael addition, whereas the Gibbs free energy of the corresponding product for the 1,6 Michael addition is 31.4 kcal/mol above the system **1t-Tf**.

**Figure 6:**
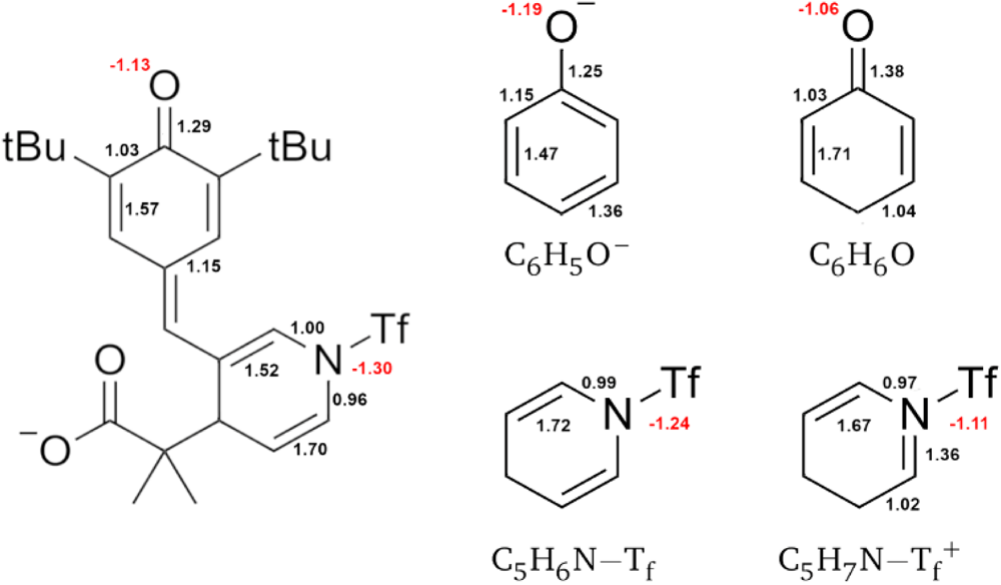
QTAIM charges (red) and delocalization indices (black) of the intermediate shown in Scheme 6 (left) and those of the phenoxide ion (top center) and a doubly conjugated cyclohexenone (top right), a triflated amine (bottom center), and a conjugated triflated enamine (bottom right).

**Scheme 6:**
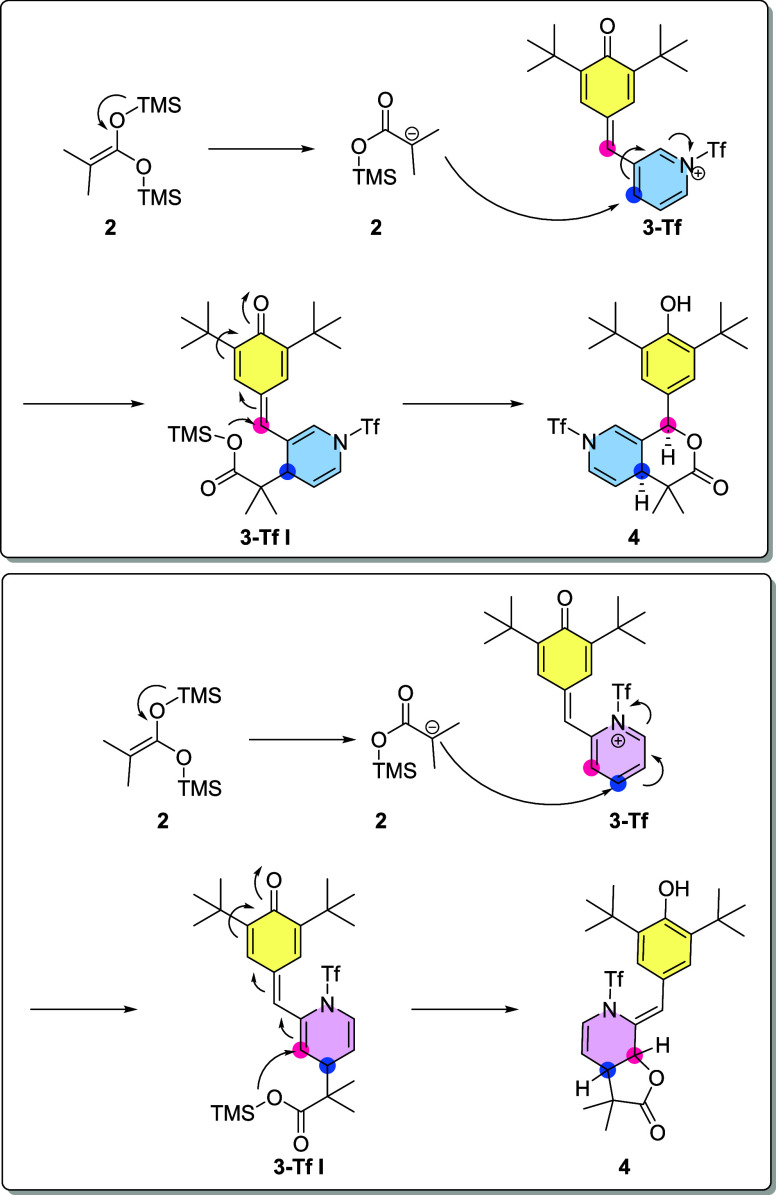
Mechanism Reaction for the 1,6 (Top) and 1,8-Michael Additions (Bottom)

We also carried out wave function analyses, which allow us to further examine the 1,6-Michael addition for systems **1s-Tf**, in particular, the two mechanism possibilities. The first possibility is that after the triflate attacks the para-position of the pyridine ring, there is an electron reorganization that corresponds to the formation of a zwitterionic species, i.e., a phenolate along with a pyridinium moiety. Subsequently, the formation of the lactone alleviates the electronic charge deficit of the pyridinium ion. The second possibility involves the formation of a neutral species after the nucleophilic attack of the triflate and the origination of the phenolate moiety following the closure of the δ-lactone ring (Scheme 6). QTAIM charges and delocalization indices (DIs) of the O–C_6_H_3_(C(CH_3_)_3_)_2_- group in **1s-Tf**, as reported in Figure 6, indicates that the chemical bonding scenario in this moiety is intermediate between that of the phenoxide ion (C_6_H_5_O^–^) and a doubly conjugated cyclohexenone (C_6_H_6_O). For example, the QTAIM charge of the O atom in the O–C_6_H_3_(C(CH_3_)_3_)_2_- moiety (−1.13 au) is intermediate between the oxygen in C_6_H_5_O^–^ (−1.19 au) and in C_6_H_6_O (−1.06 au). Likewise, for the DIs of the carbon–oxygen atoms, i.e., DI(C,O) = 1.29 au in O–C_6_H_3_(C(CH_3_)_3_)_2_, while the corresponding quantities in C_6_H_5_O^–^ and C_6_H_6_O are DI(C,O) = 1.25 au and DI(C,O) = 1.38 au. It is similar for the DIs connecting the C2–C3 and C5–C6 atoms in the form of an O–C_6_H_3_(C(CH_3_)_3_)_2_-. A different situation arises for the analysis of the pyridine moiety. The QTAIM charge of the nitrogen atom in the C_5_H_3_N(C(CH_3_)_2_COOTMS)- group (q(N) = −1.30 au) is closer to that in C_5_H_6_N-Tf (q(N) = −1.24 au) than it is to that in C_5_H_7_N-Tf^+^ (q(N) = −1.1079 au). Likewise, for the C–N bonds in the C_5_H_3_N(C(CH_3_)_2_COOTMS)- moiety, DI(C,N) = 1.00 and 0.96 in comparison to those in C_5_H_6_N-Tf, i.e., 0.99 for both C–N bonds and those in C_5_H_7_N-Tf^+^, i.e., 0.97 and 1.36 au. Overall, the wave function analyses indicate that the mechanism of the 1,6 addition of **1s-Tf** resembles more closely that of a formally neutral structure (Scheme 6) than that of a zwitterionic species.

**Scheme 7:**
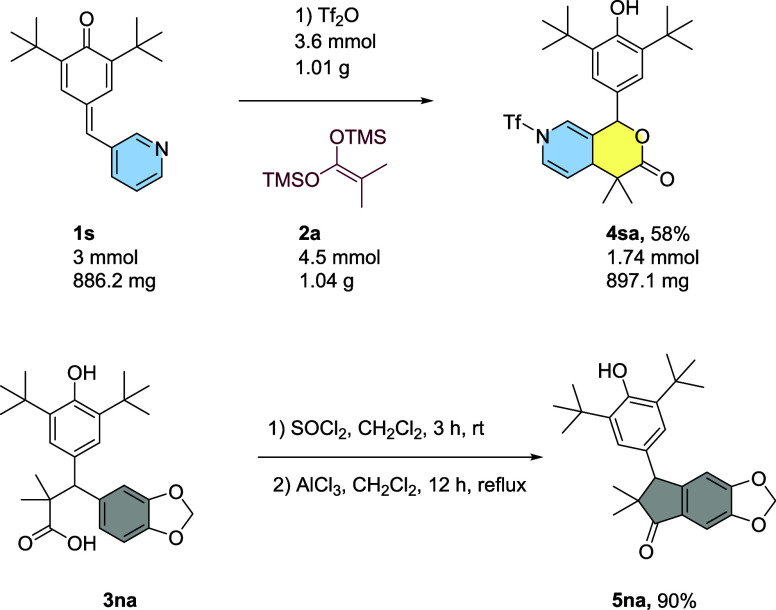
Gram-Scale Synthesis and Transformation of Product **3na** to 1-Indanone **5na**

The reaction was conducted on a gram scale under our optimal conditions (refer to Table 1, entry 1), using substrate **1s**, yielding 58% (Scheme 7). The synthesized carboxylic acids were also investigated for their potential in the synthesis of 1-indanones, which are valuable compounds in medicinal chemistry due to their diverse biological activities and applications. In an exploratory experiment, these carboxylic acids were efficiently converted into acyl chlorides by using thionyl chloride after a reaction time of 4 h (Scheme 7). Following the formation of acyl chloride, a Lewis acid, specifically aluminum chloride (AlCl_3_), was introduced into the reaction mixture. After the purification process, the reaction mixture yielded 90%.

**Figure 7:**
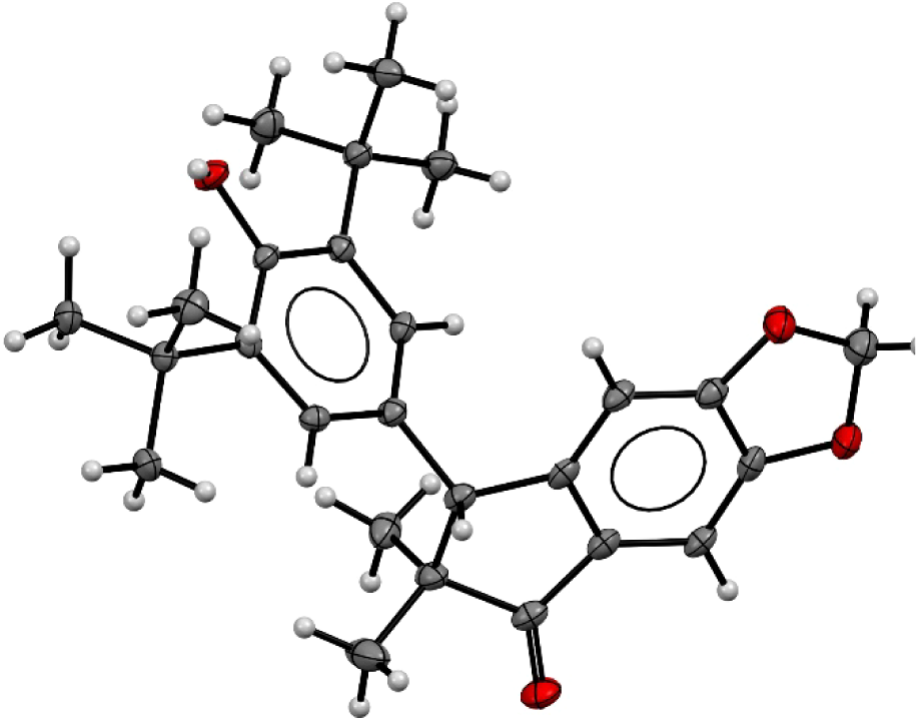
ORTEP diagram 50% probability for compound **5na**.

As a result, the reaction successfully yielded the desired 1-indanone product, and Figure 7 shows the results of X-ray crystallography confirming the structure. Future studies could focus on optimizing reaction conditions, evaluating the yields of different derivatives, and exploring the biological activities of the resulting 1-indanones.^45^

## Conclusion

These findings underscore the significance of substituent effects on the outcome of the 1,6-Michael addition reactions. They highlight the importance of considering electronic and steric factors when designing synthetic routes and optimizing the reaction conditions. By comprehensively investigating these variations, we aim to develop strategies for achieving higher yields and improved regioselectivity in the synthesis of carboxylic acids via Michael additions. As researchers continue to explore the intricacies of these reactions, the door is opened to new avenues of discovery and the potential for unlocking transformative applications in the synthesis of bioactive molecules and functional materials. Overall, this research contributes to expanding our understanding of *p*-QM reactivity and its potential in the synthesis of new 1-indanones.

## Experimental Section

### General Procedure for Carboxylic Acids (**3**) and Lactones (**4**)

In a 20 mL flask, 0.5 mmol (1 equiv) of the corresponding *p*-QM (**1)** was added, followed by 10 mL of anhydrous DCM under N_2_ atmosphere. The flask was cooled down to −78 °C in an acetone bath with an immersion chiller. Then, 0.6 mmol (1.2 equiv) of Tf_2_O was added. After 3 h of activation time, 0.75 mmol (1.5 equiv) of the corresponding bis-TMSKA was added, and the reaction was kept at −78 °C for an additional 16 h. After this time, 5 mL of water was added and stirred for 1 h at room temperature. The reaction mixture was extracted and purified through a silica gel column using an appropriate Hex:EtOAc gradient to obtain compounds **3** and **4**.

### General Procedure for Indanones (**5**)

In a 50 mL Schlenk flask, 0.5 mmol (1 equiv) of the corresponding carboxylic acid (**3**) was added, followed by 15 mL of anhydrous DCM under N_2_ atmosphere. Then, 1.5 mmol (1.5 equiv) of SOCl_2_ was added. After 3 h of reaction, the mixture was evaporated, and fresh 15 mL of anhydrous DCM was added. A condenser was connected to the Schlenk flask, and 1 mmol (1 equiv) of AlCl_3_ was added to the reaction mixture, which was then heated at reflux temperature for 12 h. The reaction mixture was extracted and purified through a silica gel column using an appropriate Hex:EtOAc gradient to obtain compound **5.**

## Data Availability

data underlying this study are available in the published article and its Supporting Information.

## Supporting Information Available

Full spectroscopic characterization of all products, including ^1^H-NMR, ^13^C-NMR, HRMS, computational details, and X-ray (PDF)
