# Supplementary material for: Conjugated 1,8 and 1,6 Addition of Bis-Trimethylsilylketene Acetal to Activated p-Quinone Methides via Trifluoromethanesulfonic Anhydride
Source: J Org Chem. 2025 Mar 31;90(17):5795–804. doi: 10.1021/acs.joc.4c02852 (PMC12053956; doi:10.1021/acs.joc.4c02852)
Supplement: Supplementary file 1 — jo4c02852_si_001.pdf [file jo4c02852_si_001.pdf]

Conjugated 1,8 and 1,6 Addition of *bis*-trimethylsilylketene Acetal to Activated *p*-Quinone Methides *via* trifluoromethanesulfonic anhydride.

Luis J. Benitez-Puebla[1], Ricardo Ballinas-Indili[1], Marcos Flores-Alamo[2], José M. Guevara-Vela[3], Tomas Rocha-Rinza[1], Saulo Rosales-Amezcua[1], Cecilio Alvarez-Toledano[1]*

[1] Universidad Nacional Autónoma de México, Instituto de Química, Circuito Exterior, Ciudad Universitaria, Alcaldía Coyoacán, C.P. 04510, Cd. Mx.

[2] Departamento de Ciencias Químicas, Facultad de Estudios Superiores Cuautitlán-UNAM, Campo 1, Avenida 1ro. de mayo s/n, Cuautitlán Izcalli, C.P. 54740, Estado de México, México

[3] Universidad Nacional Autónoma de México, Unidad de Servicios y Apoyo a la Investigación e Industria (USAII), Edificio H Mario Molina, Circuito Mario de la Cueva, Esquina circuito de la investigación Científica, CU., Coyoacán, 04510, Cd. Mx.

[4] Departamento de Química Física Aplicada, Universidad Autónoma de Madrid, Madrid 28049, Spain

*cecilio@unam.mx

GENERAL PROCEDURES----------S2

NMR AND HRMS SPECTRA.----------S3

X-RAY DATA----------S83

COMPUTATIONAL DETAILS.----------S138

General procedures

p-QMs were obtained with the methodology reported by Roiser.[1]

(1) Roiser, L.; Waser, M. Enantioselective Spirocyclopropanation of *Para* -Quinone Methides Using Ammonium Ylides. *Org. Lett.* **2017**, *19* (9), 2338–2341. https://doi.org/10.1021/acs.orglett.7b00869.

In a 20 ml flask 0.5 mmol(1 equiv.) of the corresponding *p*-QM **(1)** was added, followed by 10 mL(0.05 M) of anhydrous DCM under N_2 atmosphere, the flask was cooled down to -78 °C at an acetone bath with an immersion chiller, 0.6 mmol(1.2 equiv.) of Tf_2O was added (**Caution!:** Tf_2O is extremely reactive and will produce toxic fumes if not handle under inert atmosphere, reacts violently with water), after 3 hours of activation time 0.75 mmol(1.5 equiv.) of the corresponding acetal was added and the reactions was kept at -78 °C for an additional 16 h, after this time, 5 mL of water was added and stirred for 1 h, the reaction was extracted with water and purified by flash chromatography with a 7:3 Hex:AcOEt mixture to obtain compounds **3**.

Compounds **4** were synthetized under the same methodology. However, the reactions was purified by flash chromatography with a 5:5 Hex:AcOEt mixture to obtain the pure compounds.

NMR spectra were recorded in a Bruker-Avance 300 MHz at room temperature, HRMS were recorded in a Accu-TOF DART spectrometer.

Single crystals **5na, 4ta, 4sa** were obtained by slow evaporation with a mixture of Hex:DCM 2:1 at room temperature after one week, **4tc-RO**, was obtained after slow evaporation with a mixture of MeOH:DCM:Hexane 1:1:4 at room temperature after three days. A suitable single crystal of compounds **5na, 4ta**, **4sa** and **4tc-RO** were mounted on a glass fiber and crystallographic data were collected at 130 K with an Oxford Diffraction Gemini diffractometer ($l_{MoK}a$ = 0.71073 Å, monochromator: graphite) with a CCD-atlas area detector. CrysAlisPro and CrysAlis RED software packages were used for data collection and integration. The double pass method of scanning was used to exclude any noise. The collected frames were integrated by using an orientation matrix determined from the narrow frame scans. Final cell constants were determined by a global refinement. Final cell constants were determined by a global refinement; data were collected and corrected for absorbance using analytical numerical absorption correction with a multifaceted crystal model based on expressions upon the Laue symmetry with equivalent reflections. Structures solutions and refinement were carried out with the SHELXS-2018 and SHELXL-2018 packages. WinGX v2023 software was used to prepare material for publication. Full-matrix least-squares refinement was carried out by minimizing $(Fo^2 – Fc^2)^2$. All non-hydrogen atoms were refined anisotropically. H atoms attached to C atoms were placed in geometrically idealized positions and refined as riding on their parent atoms, with C–H = 0.98 – 1.00 Å with $U_{iso}(H)$ = $1.2U_{eq}(C)$ for methylene and methine groups and $U_{iso}(H)$ = $1.5U_{eq}(C)$ for methyl groups. Table 1 shows the crystallographic data summary. The crystallographic data has been deposited with the Cambridge Crystallographic Data Centre as supplementary publication no. CCDC 2366599-2366601. Copies of the data can be obtained free of charge on application to CCDC, 12 Union Road, Cambridge, CB2 1EZ, UK (fax: (+44) 1223-336-033, e-mail: deposit@ccdc.cam.ac.uk).

NMR and HRMS spectra.

3-(3,5-di-tert-butyl-4-hydroxyphenyl)-2,2-dimethyl-3-phenylpropanoic acid (3aa): 190 mg, 99% yield, 114 mg (White solid), ^{1}H-NMR (300 MHz, Chloroform-*d*) δ 7.46 – 7.16 (m, 6H), 7.11 (s, 2H), 4.34 (s, 1H), 1.40 (s, 18H), 1.26 (d, J = 4.5 Hz, 6H); ^{13}C{^{1}H} NMR (75 MHz, $CDCl_3$) δ 184.08, 152.20, 141.78, 135.00, 131.44, 129.91, 127.92, 126.39, 126.29, 58.75, 46.68, 34.29, 30.40, 30.37, 30.32, 24.30, 23.90; HRMS (DART-ESI^{+}) m/z: $[M+NH_4]^+$ Calcd for $C_{25}H_{38}NO_3$, 400.2846, Found, 400.2834.

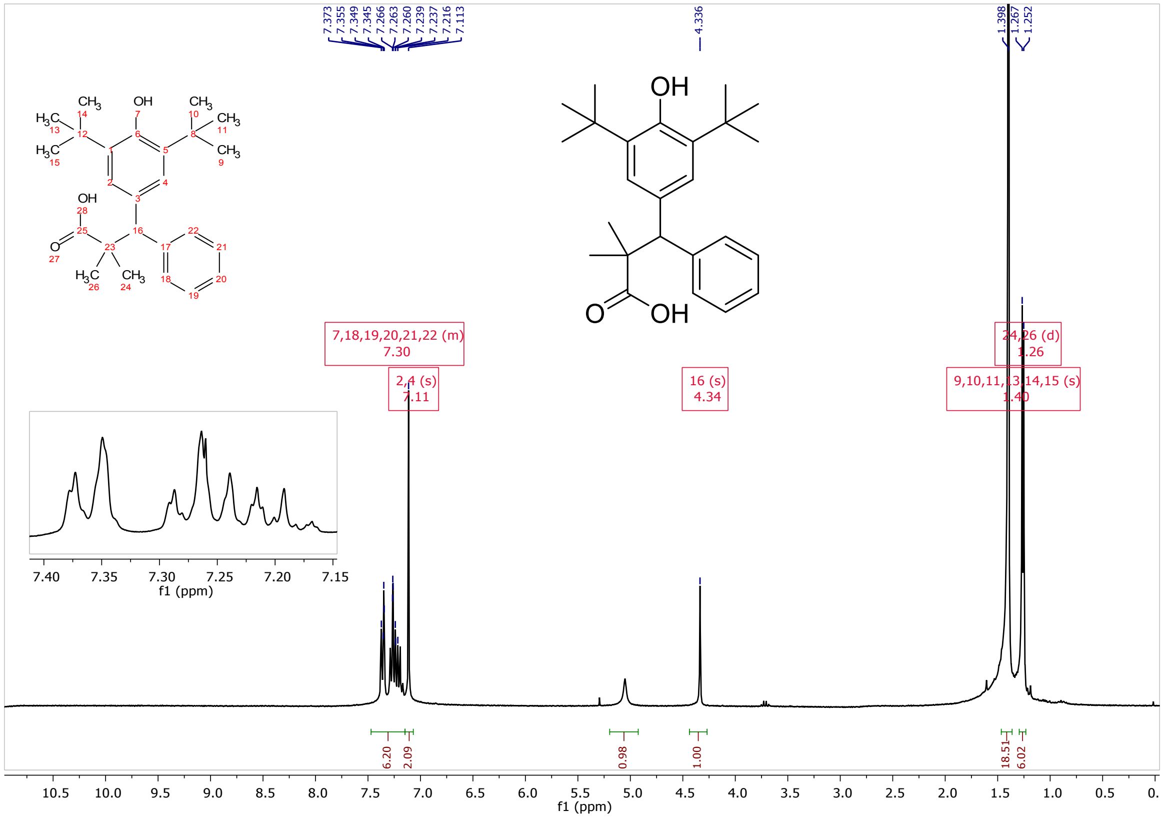

Figure S1. ^{1}**H-NMR, 300 MHz,** $CDCl_3$ **for 3aa**

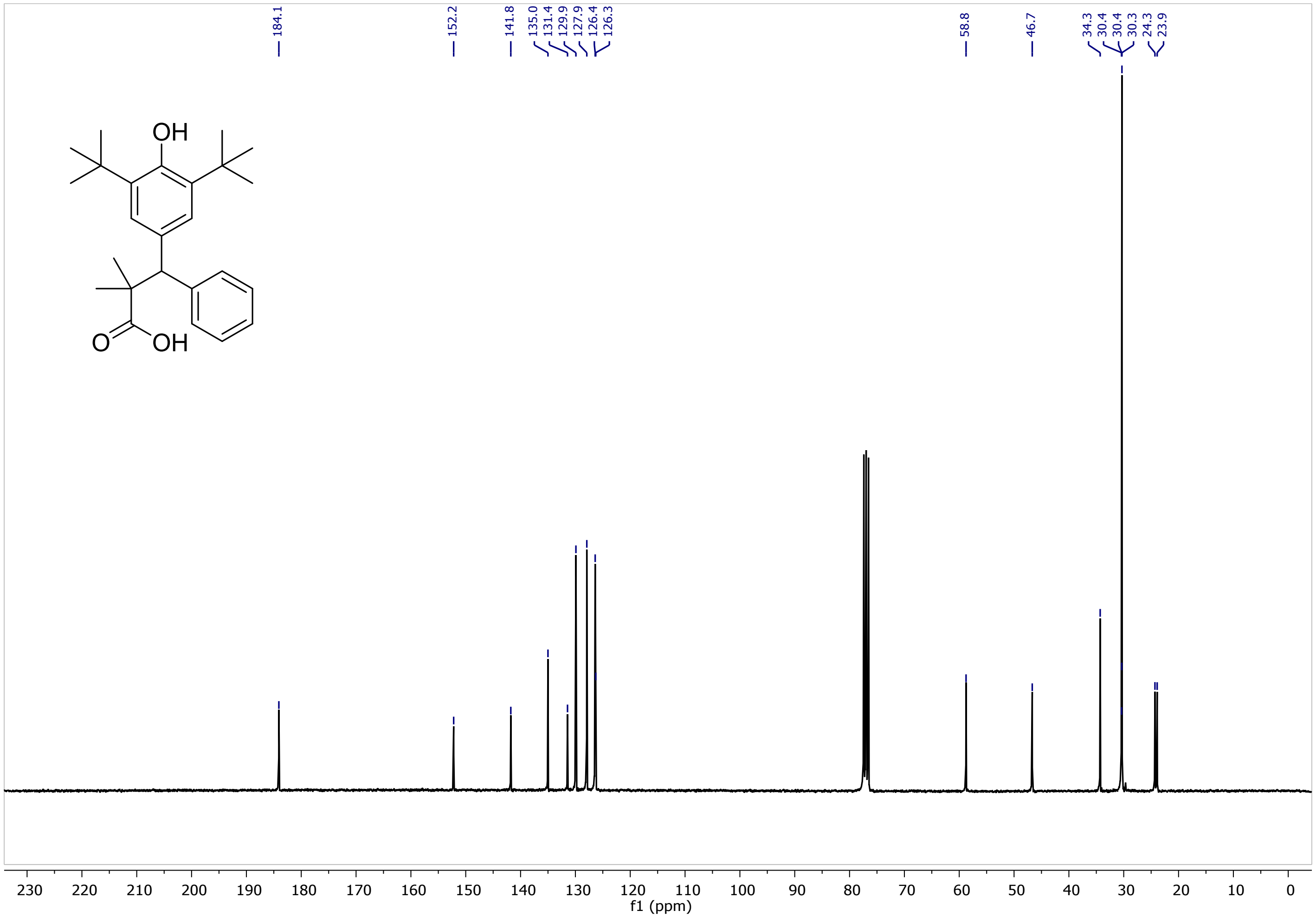

Figure S2. $^{13}C\{^{1}H\}$**-NMR, 75 MHz,** $CDCl_3$ **for 3aa**

INSTITUTO DE QUIMICA, UNAM
LABORATORIO DE ESPECTROMETRIA DE MASAS

Data:1196 A-H
Sample Name:Dr. Alvarez Cecilio / Operador Javier Perez
Description:
Ionization Mode:ESI+
History:Determine m/z[Peak Detect[Centroid,30,Area];Correct Base[];Smooth[5]];Correct Base[5.0%];Average(MS[...

Acquired:5/3/2023 9:20:09 AM
Operator:AccuTOF
Mass Calibration data:cal-PEG-600-nuevo
Created:10/4/2023 3:55:35 PM
Created by:AccuTOF

Charge number:1
Tolerance:40.00(ppm), 5.00 .. 15.00(mmu)
Unsaturation Number:0.0 .. 100.0 (Fraction:Both)
Element:^{12}C:0 .. 25, ^{1}H:0 .. 40, ^{14}N:0 .. 1, ^{16}O:0 .. 4

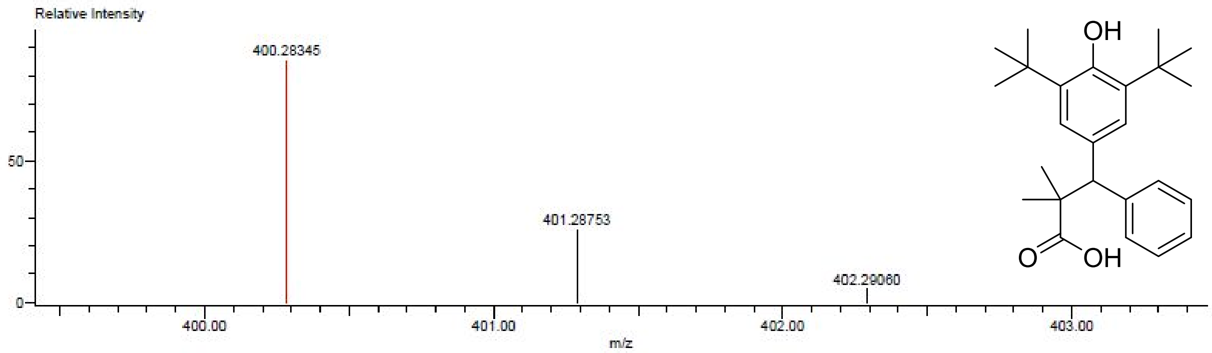

Mass	Intensity	Calc. Mass	Mass Difference (mmu)	Mass Difference (ppm)	Possible Formula	Unsaturation Number
400.28345	157264.77	400.28517	-1.72	-4.29	$^{12}C_{25}{}^{1}H_{38}{}^{14}N_{1}{}^{16}O_{3}$	7.5

Figure S3. HRMS, DART-ESI^{+} (TOF) for 3aa

3-(3,5-di-*tert*-butyl-4-hydroxyphenyl)-2,2-dimethyl-3-(4-methylphenyl)propanoic acid (3ba): 195 mg, 98% yield (White solid) ^{1}H NMR (300 MHz, Chloroform-*d*) δ 7.33 – 7.25 (m, 2H), 7.15 (s, 2H), 7.10 (d, *J* = 7.9 Hz, 2H), 4.33 (s, 1H), 2.34 (s, 3H), 1.43 (s, 17H), 1.28 (d, *J* = 2.7 Hz, 6H); $^{13}C\{^{1}H\}$ NMR (75 MHz, $CDCl_3$) δ 184.5, 152.1, 138.7, 135.7, 134.9, 131.6, 129.8, 128.6, 126.3, 58.4, 46.8, 34.3, 30.3, 24.3, 23.8, 20.9; HRMS (DART-ESI$^+$) m/z:$[M+NH_4]^+$ Calculated for $C_{26}H_{40}NO_{3,}$ 414.3003 , Found, 414.2991

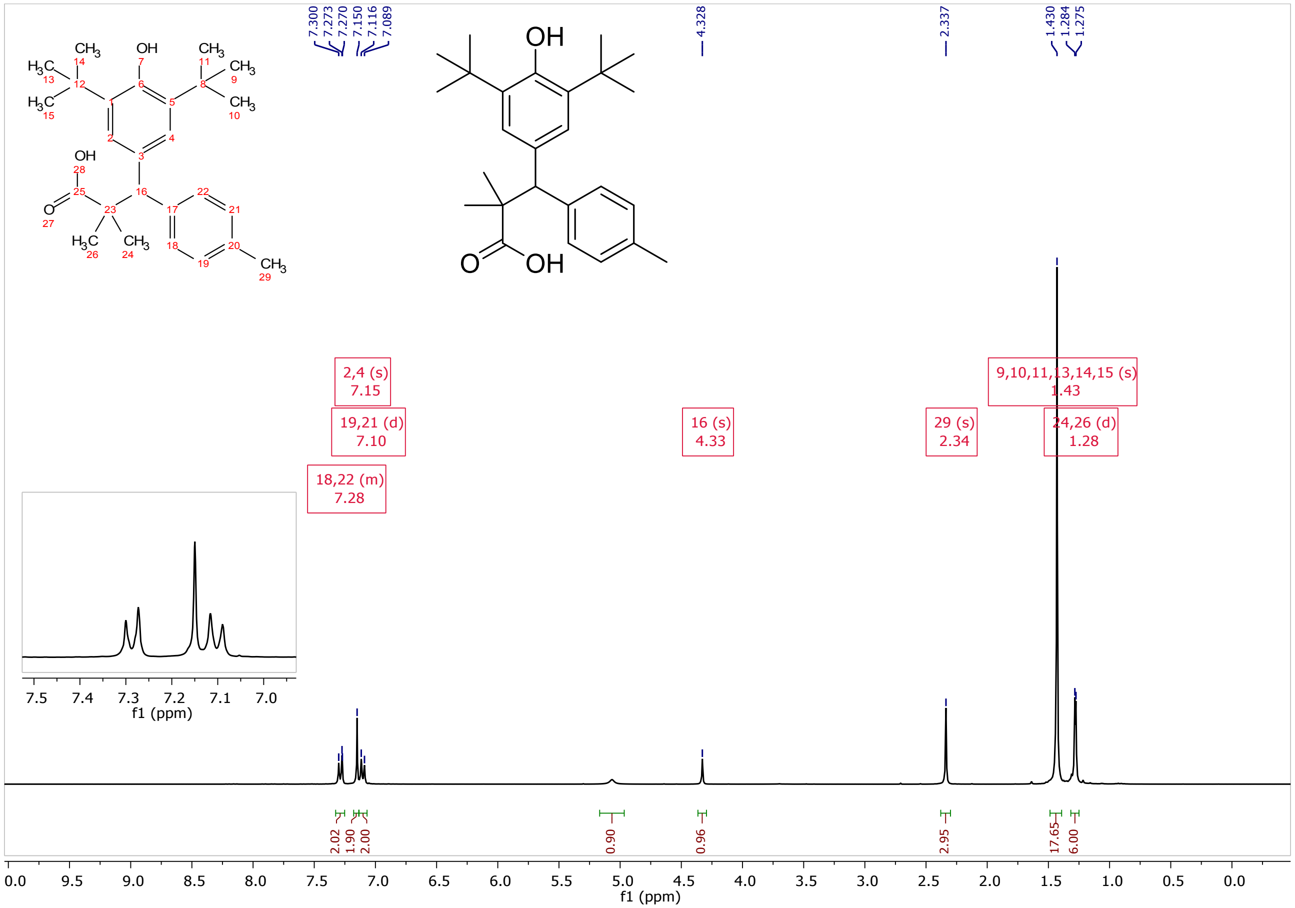

Figure S4. ^{1}H**-NMR, 300 MHz,** $CDCl_3$ **for 3ba**

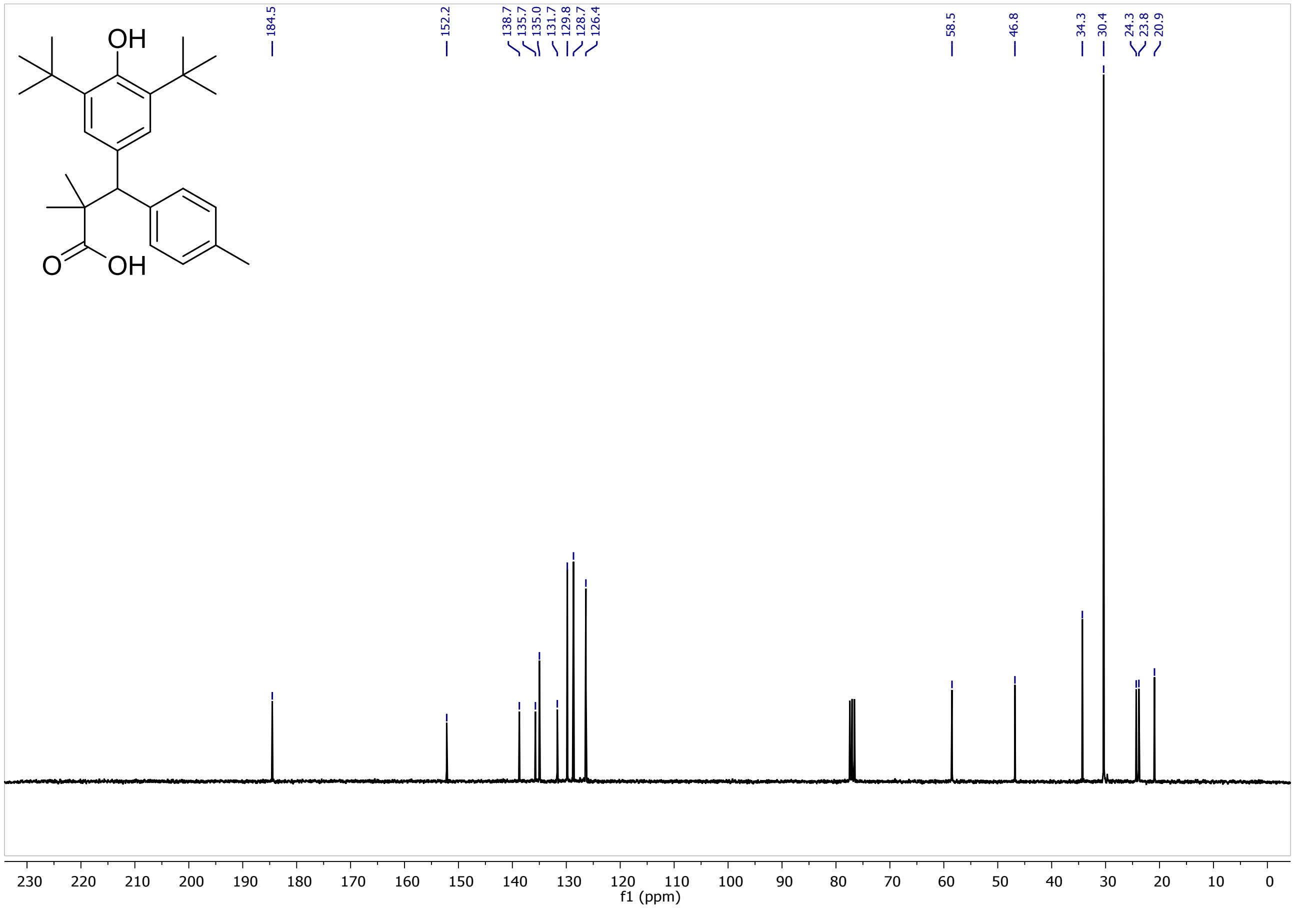

Figure S5. $^{13}C\{^{1}H\}$**-NMR, 75 MHz,** $CDCl_3$ **for 3ba**

Data:938 A-meti-
Sample Name:Dr Alvarez Cecilio / Operador: Javier Perez
Description:
Ionization Mode:ESI+
History:Determine m/z[Peak Detect[Centroid,30,Area];Correct Base[];Smooth[5]];Correct Base[5.0%];Average(MS[...

Acquired:3/30/2023 2:52:31 PM
Operator:AccuTOF
Mass Calibration data:24enero2023
Created:3/30/2023 3:18:20 PM
Created by:AccuTOF

Charge number:1
Element:^{12}C:0 .. 30, ^{1}H:0 .. 60, ^{14}N:0 .. 1, ^{16}O:0 .. 4

Tolerance:3.00(mmu)

Unsaturation Number:-1.0 .. 50.0 (Fraction:.5)

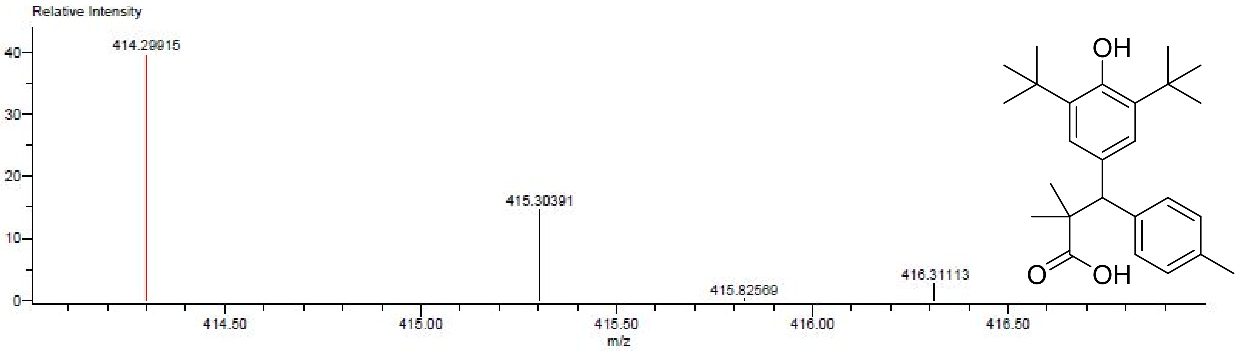

Mass	Intensity	Calc. Mass	Mass Difference (mmu)	Mass Difference (ppm)	Possible Formula	Unsaturation Number
414.29915	19559.93	414.30082	-1.67	-4.04	$^{12}C_{26}{}^{1}H_{40}{}^{14}N_{1}{}^{16}O_{3}$	7.5

Figure S6. HRMS, DART-ESI^{+} (TOF) for 3ba

3-(3,5-di-tert-butyl-4-hydroxyphenyl)-3-(4-methoxyphenyl)-2,2-dimethylpropanoic acid (3ca): 145 mg, 70% yield (White solid), ^{1}H NMR (400 MHz, Chloroform-*d*) δ 7.30 (d, *J* = 8.7 Hz, 2H), 7.13 (s, 2H), 6.94 – 6.74 (m, 2H), 5.07 (s, 1H), 4.31 (s, 1H), 3.79 (d, *J* = 0.8 Hz, 3H), 1.42 (d, *J* = 0.9 Hz, 23H), 1.26 (d, *J* = 3.8 Hz, 8H); $^{13}C\{^{1}H\}$ NMR (75 MHz, $CDCl_3$) δ 184.7, 158.0, 152.2, 135.0, 134.0, 131.8, 130.9, 126.3, 113.4, 58.0, 55.1, 46.9, 34.3, 30.4, 24.4, 23.8; HRMS (DART-ESI^{+}) m/z: $[M+NH_4]^+$ Calcd for $C_{26}H_{40}NO_4$, 430.2953, Found, 430.2962.

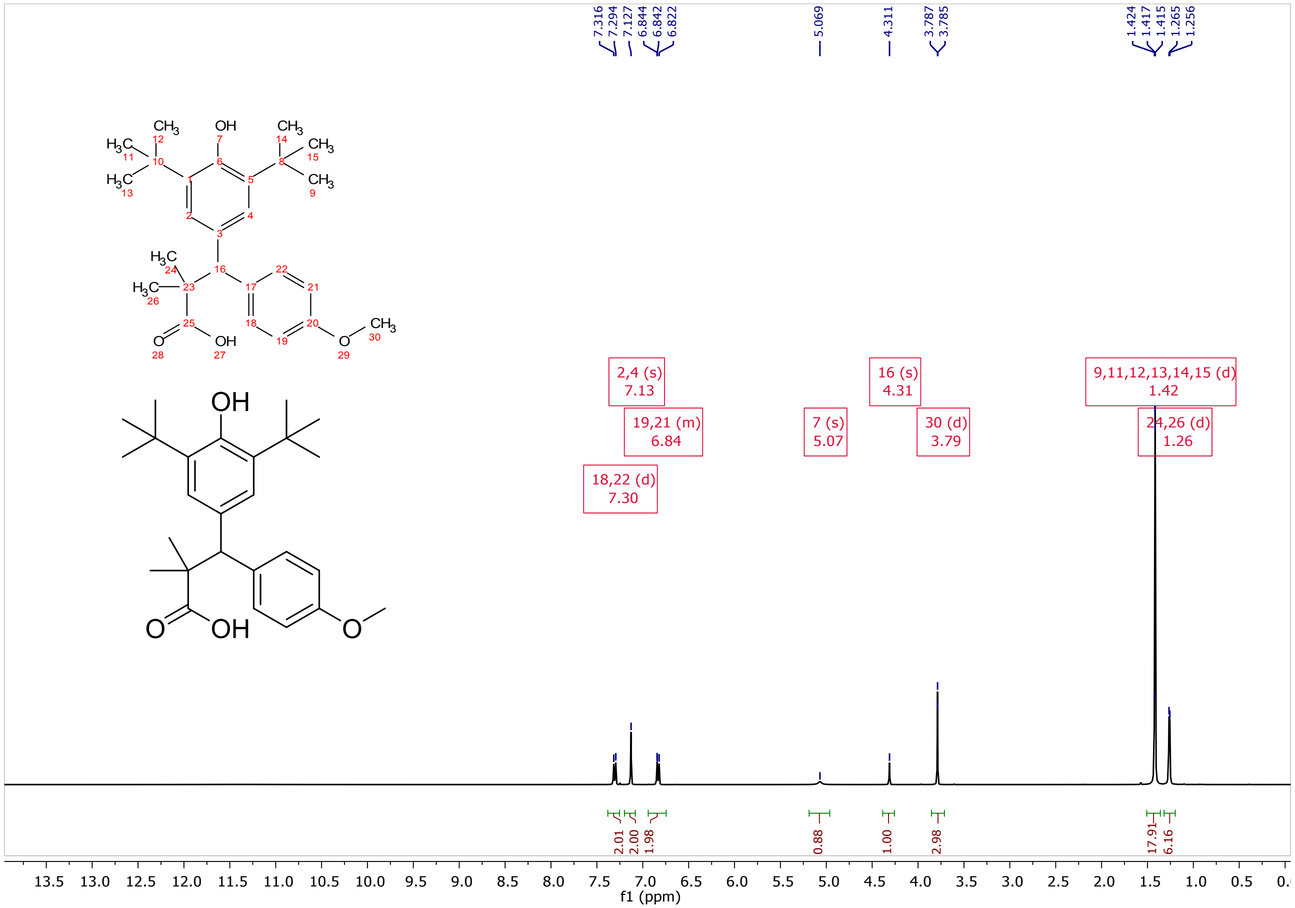

Figure S7. ^{1}**H-NMR, 300 MHz,** $CDCl_3$ **for 3ca**

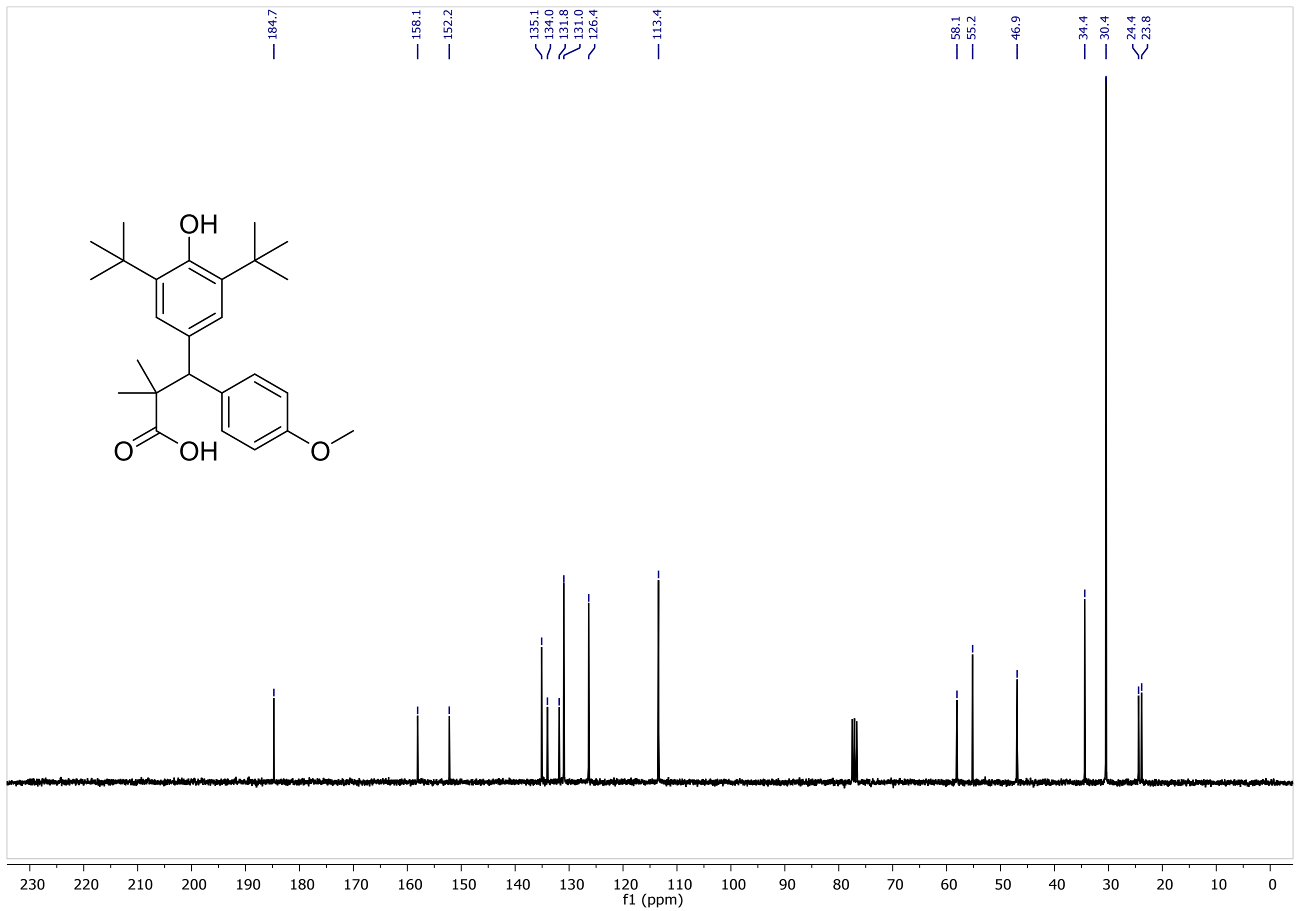

Figure S8. $^{13}C\{^{1}H\}$-NMR, 75 MHz, $CDCl_3$ for 3ca

Data:432 A-oMe
Sample Name:Dr. Alvarez Cecilio / Operador Javier Perez
Description:
Ionization Mode:ESI+
History:Determine m/z[Peak Detect[Centroid,30,Area];Correct Base[];Smooth[5]];Correct Base[5.0%];Average(MS[...

Acquired:2/21/2023 8:13:43 AM
Operator:AccuTOF
Mass Calibration data:24enero2023
Created:3/21/2023 3:35:14 PM
Created by:AccuTOF

Charge number:1
Tolerance:2.50(mmu)
Unsaturation Number:0.0 .. 11.0 (Fraction:Both)
Element:^{12}C:0 .. 30, ^{1}H:0 .. 60, ^{14}N:1 .. 3, ^{16}O:0 .. 5

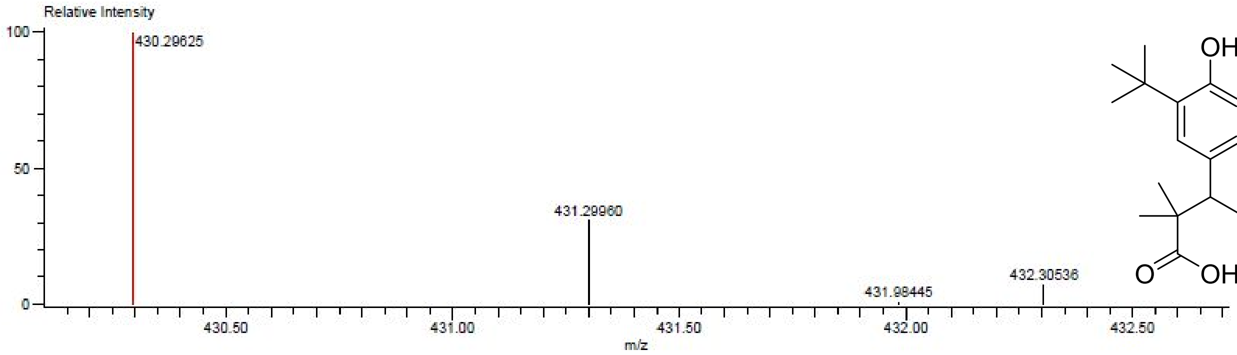

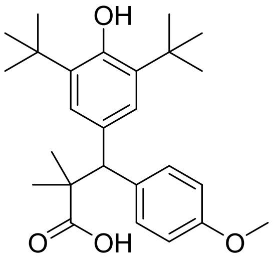

Mass	Intensity	Calc. Mass	Mass Difference (mmu)	Mass Difference (ppm)	Possible Formula	Unsaturation Number
430.29625	14482.11	430.29573	0.52	1.20	$^{12}C_{25}{}^{1}H_{40}{}^{14}N_{1}{}^{16}O_{4}$	7.5

Figure S9. **HRMS, DART-ESI^{+} (TOF) for 3ca**

3-(3,5-di-*tert*-butyl-4-hydroxyphenyl)-3-(3-methoxyphenyl)-2,2-dimethylpropanoic acid (3da): 145 mg, 70% Yield (White solid), ^{1}H NMR (300 MHz, Chloroform-*d*) δ 7.17 (t, *J* = 7.9 Hz, 1H), 7.11 (s, 2H), 6.99 – 6.90 (m, 2H), 6.74 (ddd, *J* = 8.2, 2.6, 0.9 Hz, 1H), 5.04 (s, 1H), 4.29 (s, 1H), 3.77 (s, 3H), 1.39 (s, 18H), 1.26 (s, 6H); ^{13}C{^{1}H} NMR (75 MHz, $CDCl_3$) δ 181.8, 158.3, 151.4, 142.5, 134.1, 130.4, 127.9, 125.5, 121.4, 115.1, 110.7, 58.0, 54.2, 45.8, 33.4, 29.5, 23.4, 23.2; HRMS (DART-ESI$^+$) m/z: $[M+NH_4]^+$ Calcd for $C_{26}H_{36}O_4$, 430.2953, Found, 430.2955.

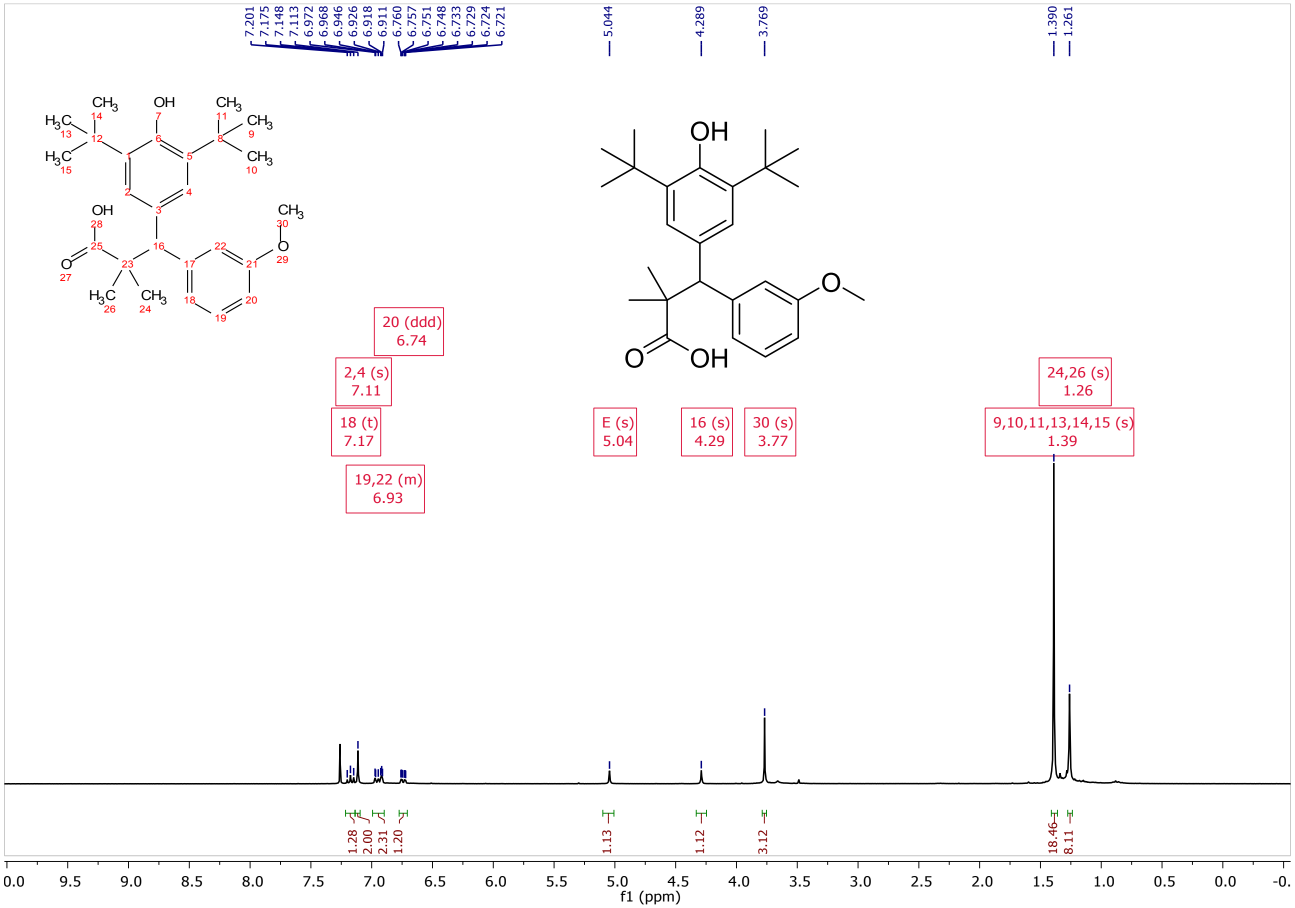

Figure S10. ^{1}H-NMR, 300 MHz, $CDCl_3$ for 3da

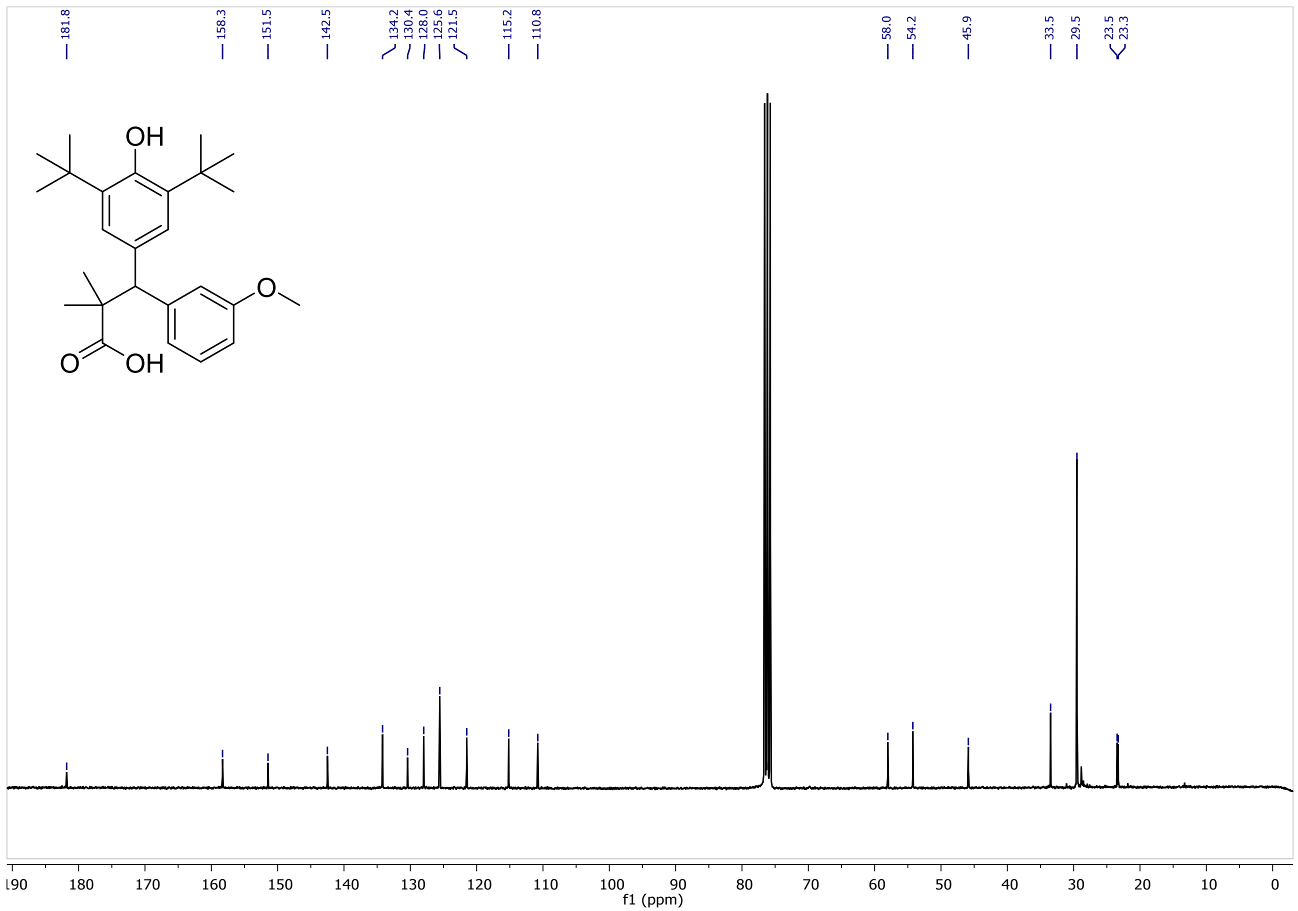

Figure S11. $^{13}C\{^1H\}$-NMR, 75 MHz, $CDCl_3$ for 3da

INSTITUTO DE QUIMICA, UNAM
LABORATORIO DE ESPECTROMETRIA DE MASAS

Data:774 A-m-OMeF3
Sample Name:Dr Alvarez Cecilio/ Operador Javier Perez
Description:
Ionization Mode:ESI+
History:Determine m/z[Peak Detect[Centroid,30,Area];Correct Base[];Smooth[5]];Correct Base[5.0%];Average(MS[...

Acquired:3/14/2023 5:59:02 PM
Operator:AccuTOF
Mass Calibration data:Cal_PEG_600
Created:3/29/2023 11:47:36 AM
Created by:AccuTOF

Charge number:1
Tolerance:3.00(ppm), 5.00 .. 15.00(mmu)
Unsaturation Number:-1.0 .. 50.0 (Fraction:Both)
Element:^{12}C:0 .. 30, ^{1}H:0 .. 40, ^{14}N:0 .. 3, ^{16}O:4 .. 4

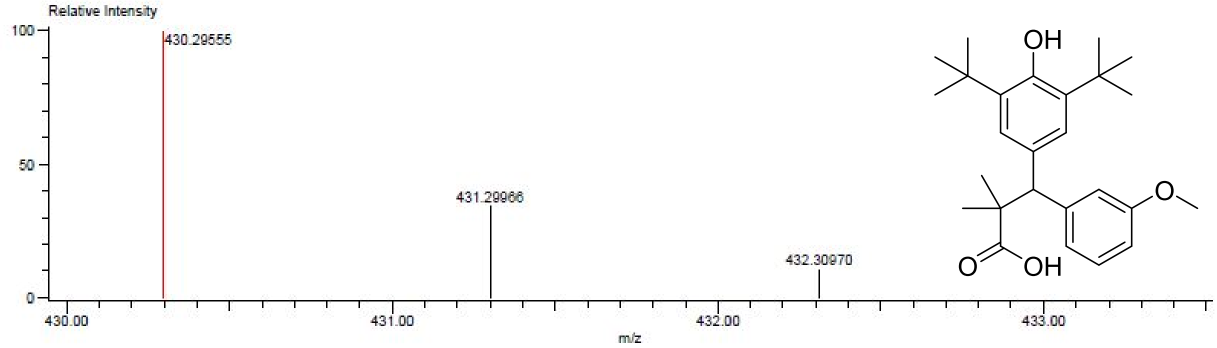

Mass	Intensity	Calc. Mass	Mass Difference (mmu)	Mass Difference (ppm)	Possible Formula	Unsaturation Number
430.29555	42605.02	430.29573	-0.18	-0.42	$^{12}C_{26}{}^{1}H_{40}{}^{14}N_{1}{}^{16}O_{4}$	7.5

Figure S12. HRMS, DART-ESI^{+} (TOF) for 3da

3-(3,5-di-*tert*-butyl-4-hydroxyphenyl)-3-(2-methoxyphenyl)-2,2-dimethylpropanoic acid (3ea): 15 mg, 7% Yield (White solid), ^{1}H NMR (300 MHz, Chloroform-*d*) δ 7.44 (dd, *J* = 7.7, 1.7 Hz, 1H), 7.16 (ddd, *J* = 8.3, 7.4, 1.6 Hz, 1H), 7.10 (s, 2H), 6.93 – 6.80 (m, 2H), 4.99 (s, 1H), 3.72 (s, 2H), 1.38 (s, 18H), 1.25 (d, *J* = 19.0 Hz, 6H); $^{13}C\{^{1}H\}$ NMR (75 MHz, $CDCl_3$) δ 184.7, 157.5, 152.0, 134.7, 131.7, 130.8, 129.9, 127.2, 126.7, 120.0, 110.9, 55.5, 48.7, 46.6, 34.3, 30.4, 24.8, 24.0; HRMS (DART-ESI$^+$) m/z: $[M+H]^+$ Calcd for $C_{26}H_{40}NO_{4,}$ 413.2686, Found, 413.2712.

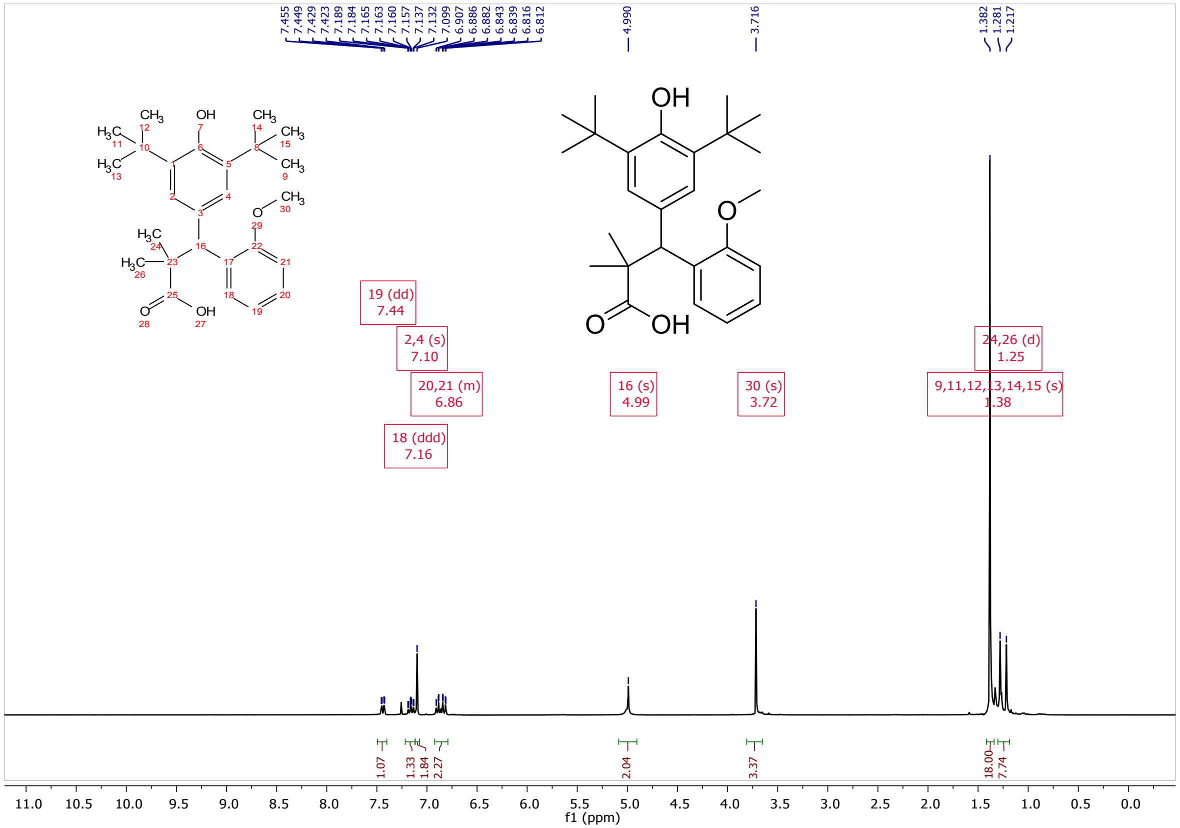

Figure S13. ^{1}**H-NMR, 300 MHz,** $CDCl_3$ **for 3ea**

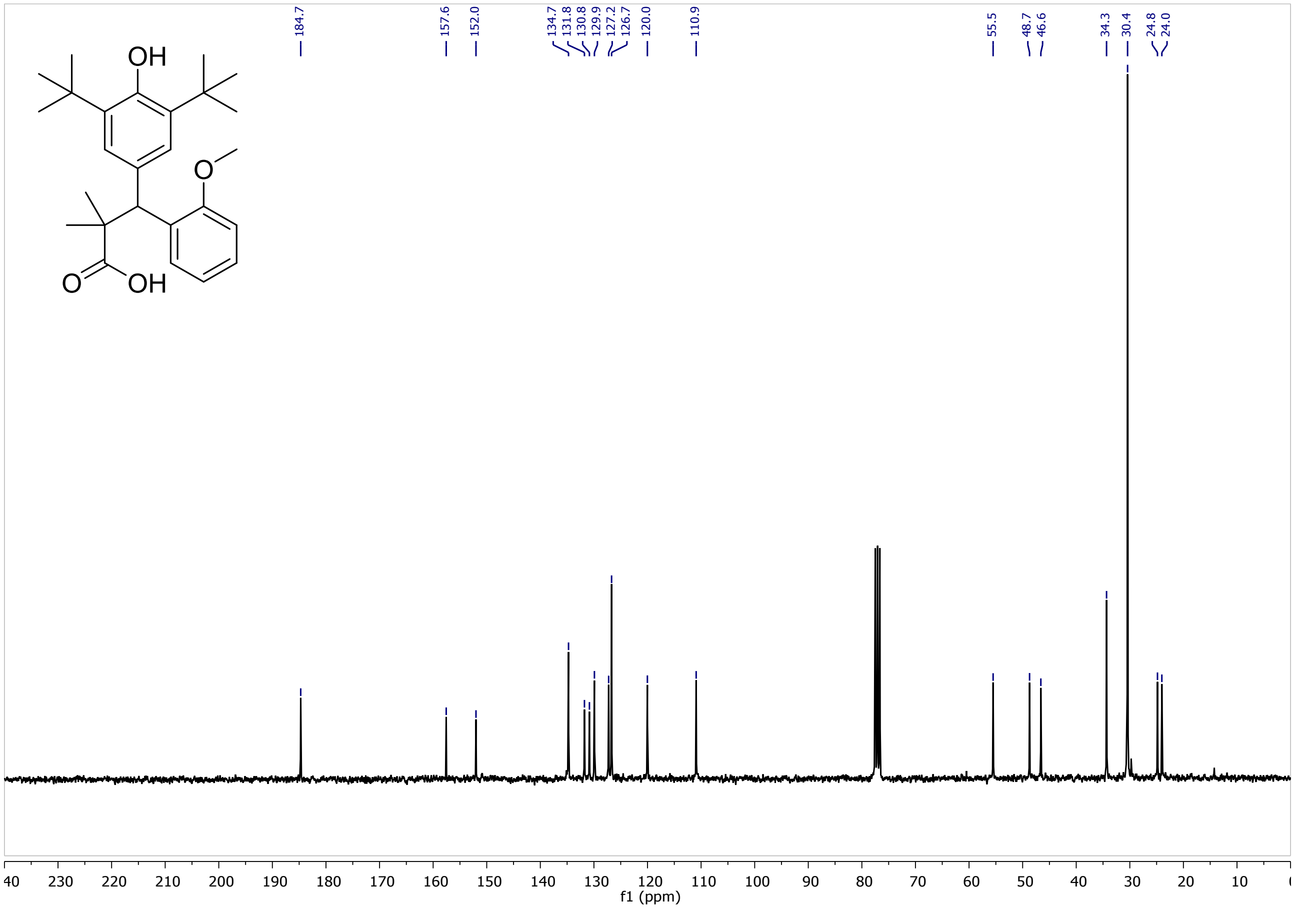

Figure S14. $^{13}C\{^{1}H\}$**-NMR, 75 MHz,** $CDCl_3$ **for 3ea**

INSTITUTO DE QUIMICA, UNAM
LABORATORIO DE ESPECTROMETRIA DE MASAS

Data:778 A-o-OMe
Sample Name:Dr Alvarez Cecilio/ Operador Javier Perez
Description:
Ionization Mode:ESI+
History:Determine m/z[Peak Detect[Centroid,30,Area];Correct Base[];Smooth[5]];Correct Base[5.0%];Average(MS[...

Acquired:3/14/2023 6:07:03 PM
Operator:AccuTOF
Mass Calibration data:Cal_PEG_600
Created:3/29/2023 11:41:30 AM
Created by:AccuTOF

Charge number:1
Element:^{12}C:0 .. 30, ^{1}H:0 .. 40, ^{14}N:0 .. 3, ^{16}O:4 .. 4

Tolerance:5.00(ppm), 5.00 .. 15.00(mmu)

Unsaturation Number:-1.0 .. 50.0 (Fraction:Both)

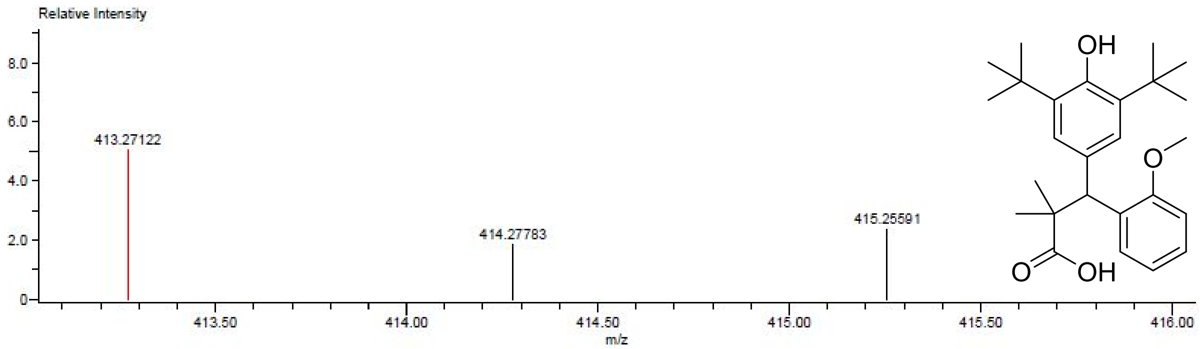

Mass	Intensity	Calc. Mass	Mass Difference (mmu)	Mass Difference (ppm)	Possible Formula	Unsaturation Number
413.27122	9087.41	413.26918	2.03	4.92	$^{12}C_{25}{}^{1}H_{37}{}^{16}O_4$	8.5

Figure S15. **HRMS, DART-ESI⁺ (TOF) for 3ea**

3-(2-bromophenyl)-3-(3,5-di-*tert*-butyl-4-hydroxyphenyl)-2,2-dimethylpropanoic acid (3fa): 70 mg, 31% Yield (White solid), ^{1}H NMR (300 MHz, Chloroform-*d*) δ 7.70 – 7.44 (m, 2H), 7.31 – 7.18 (m, 1H), 7.11 – 6.94 (m, 2H), 5.03 (d, *J* = 10.7 Hz, 2H), 1.44 – 1.23 (m, 24H); $^{13}C\{^{1}H\}$ NMR (75 MHz, $CDCl_3$) δ 183.9, 152.2, 141.6, 134.9, 133.3, 130.1, 129.8, 127.8, 126.9, 126.9, 126.7, 55.8, 46.8, 34.3, 30.3, 24.9, 24.2; HRMS (DART-ESI^{+}) m/z: $[M+NH_4]^{+}$ Calcd for $C_{25}H_{37}BrNO_3$, 478.1951, Found, 478.1949.

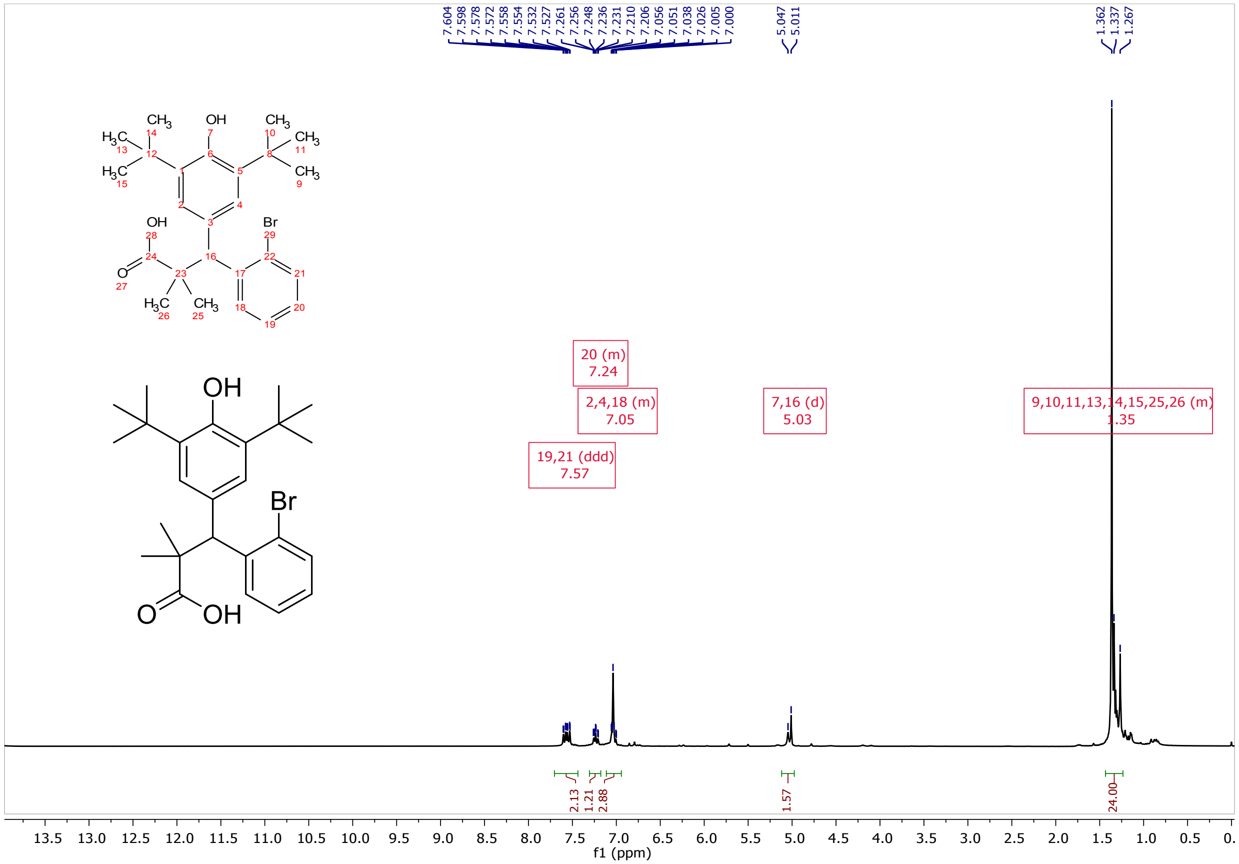

Figure S16. **^{1}H-NMR, 300 MHz, $CDCl_3$ for 3fa**

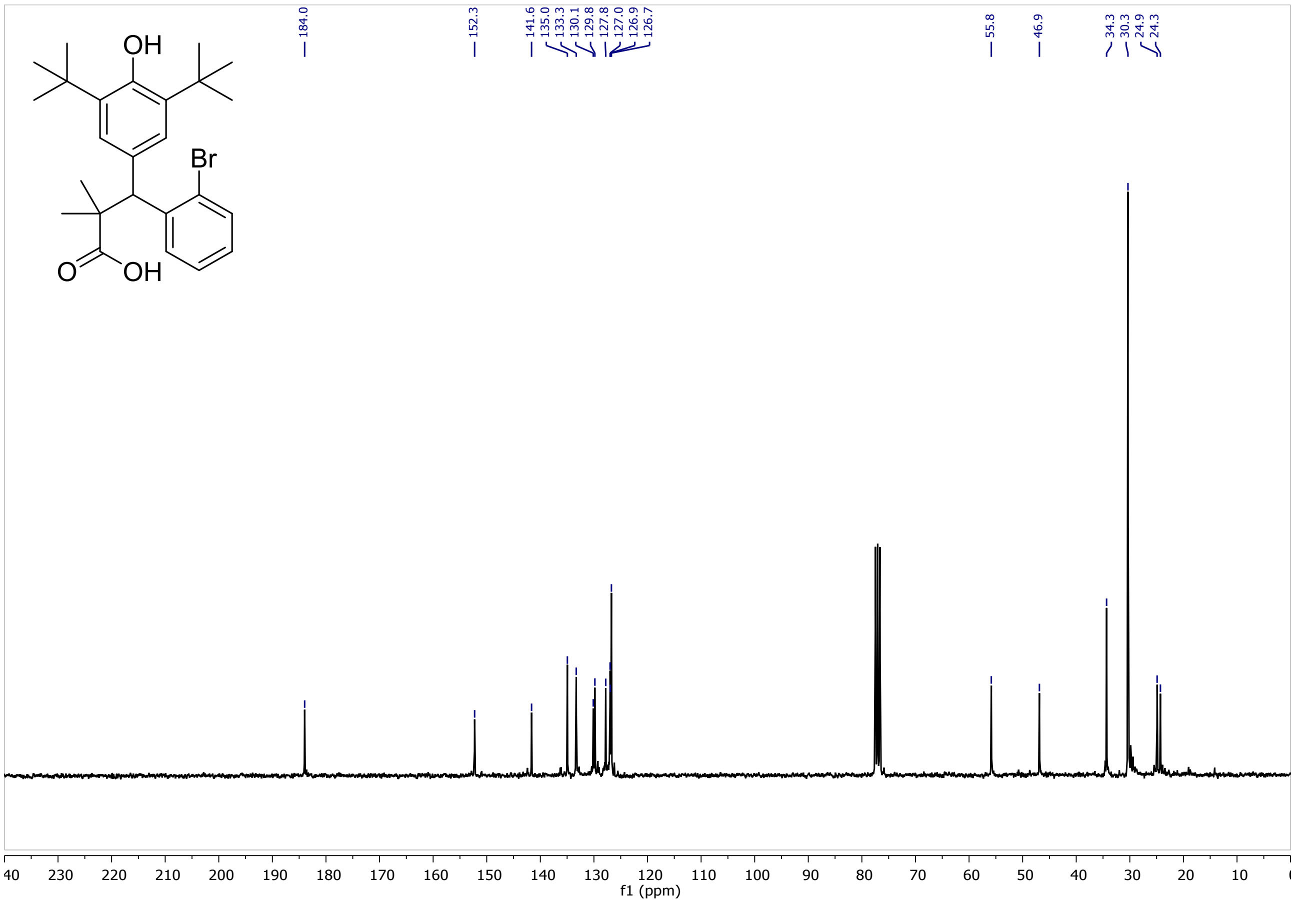

Figure S17. $^{13}C\{^{1}H\}$**-NMR, 75 MHz,** $CDCl_3$ **for 3fa**

3-(4-bromophenyl)-3-(3,5-di-*tert*-butyl-4-hydroxyphenyl)-2,2-dimethylpropanoic acid (3ga): 185 mg, 82% Yield (White solid), ^{1}H NMR (300 MHz, Chloroform-*d*) δ 7.40 (d, *J* = 8.4 Hz, 2H), 7.23 (d, *J* = 8.5 Hz, 2H), 7.06 (s, 2H), 4.30 (s, 1H), 1.41 (d, *J* = 3.0 Hz, 18H), 1.25 (d, *J* = 1.4 Hz, 6H); ^{13}C{^{1}H} NMR (75 MHz, $CDCl_3$) δ 184.4, 152.5, 141.1, 135.3, 131.7, 131.1, 130.9, 126.4, 120.4, 58.2, 46.6, 34.4, 30.4, 24.3, 24.1; HRMS (DART-ESI^{+}) m/z: $[M+NH_4]^{+}$ Calcd for $C_{25}H_{37}BrNO_3$, 478.1951, Found, 478.1949.

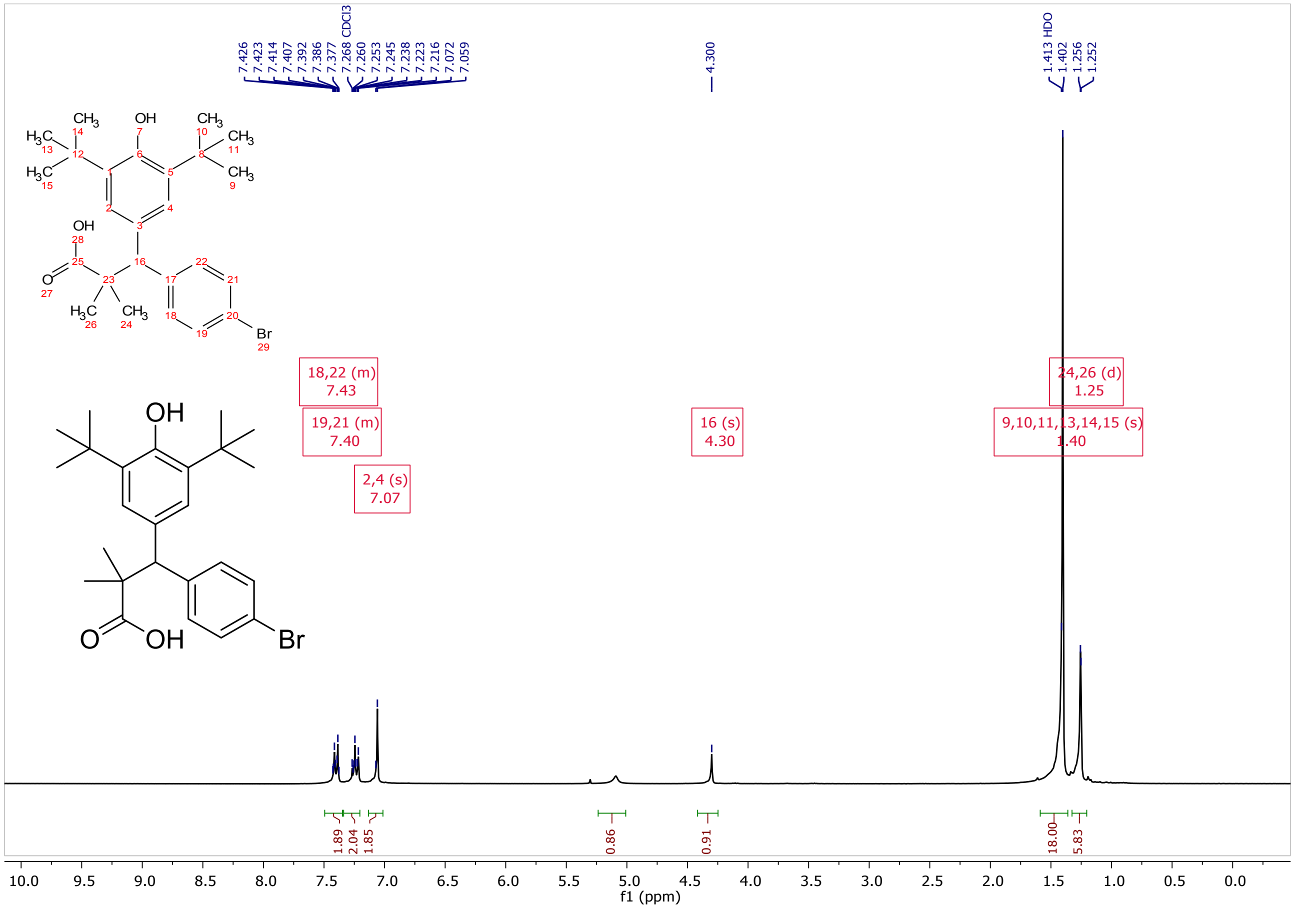

Figure S18. ^{1}H-NMR, 300 MHz, $CDCl_3$ for 3ga

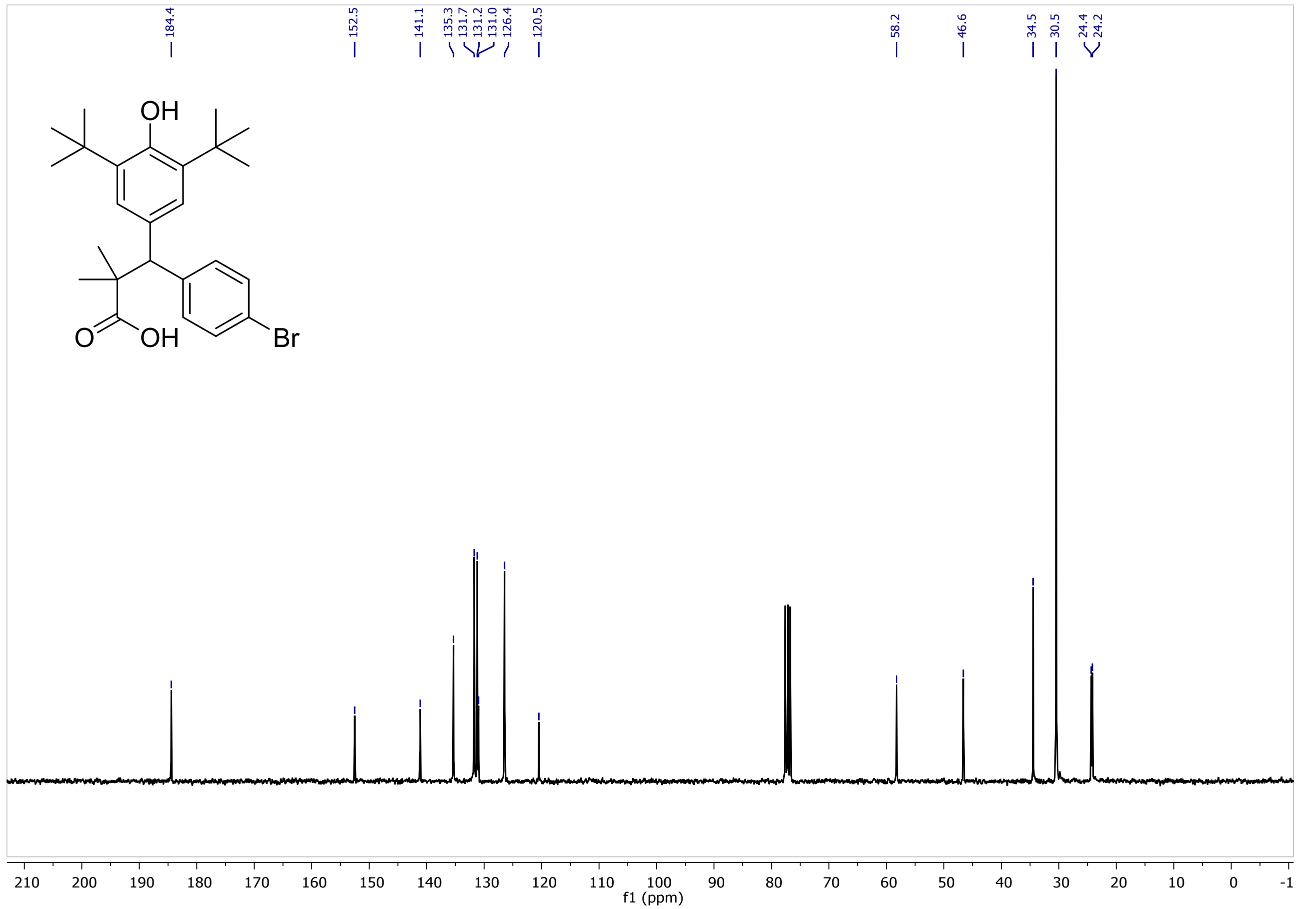

Figure S19. $^{13}C\{^{1}H\}$**-NMR, 75 MHz, $CDCl_3$ for 3ga**

Data:433 A-pBr
Sample Name:Dr. Alvarez Cecilio / Operador Javier Perez
Description:
Ionization Mode:ESI+
History:Determine m/z[Peak Detect[Centroid,30,Area];Correct Base[];Smooth[5]];Correct Base[5.0%];Average(MS[...

Acquired:2/21/2023 8:17:16 AM
Operator:AccuTOF
Mass Calibration data:24enero2023
Created:3/21/2023 3:57:21 PM
Created by:AccuTOF

Charge number:1 Tolerance:2.50(mmu)
Unsaturation Number:0.0 .. 11.0 (Fraction:Both)
Element:^{12}C:0 .. 30, ^{1}H:0 .. 60, ^{79}Br:1 .. 1, ^{14}N:1 .. 2, ^{16}O:0 .. 5

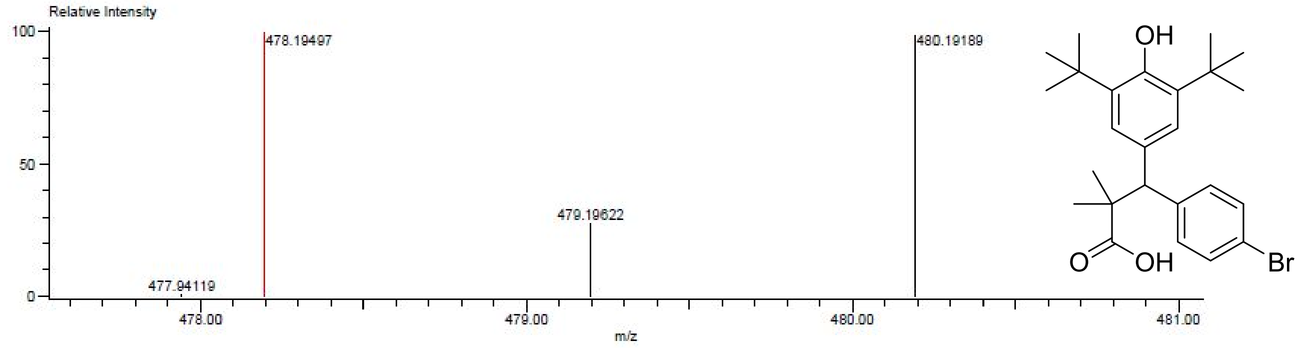

Mass	Intensity	Calc. Mass	Mass Difference (mmu)	Mass Difference (ppm)	Possible Formula	Unsaturation Number
478.19497	15776.09	478.19568	-0.71	-1.49	$^{12}C_{25}{}^{1}H_{37}{}^{79}Br_{1}{}^{14}N_{1}{}^{16}O_{3}$	7.5

Figure S20. **HRMS, DART-ESI⁺ (TOF) for 3ga**

3-(3,5-di-*tert*-butyl-4-hydroxyphenyl)-3-(4-fluorophenyl)-2,2-dimethylpropanoic acid (3ha): 181 mg, 90% Yield (White solid), ^{1}H NMR (300 MHz, Chloroform-*d*) δ 7.32 (dd, *J* = 8.6, 5.5 Hz, 2H), 7.08 (s, 2H), 6.96 (t, *J* = 8.7 Hz, 2H), 4.34 (s, 1H), 1.41 (s, 18H), 1.26 (d, *J* = 3.5 Hz, 6H); $^{13}C\{^{1}H\}$ NMR (101 MHz, CHLOROFORM-*D*) δ 184.5, (*d*, 162.8, 160.4 *J* = 244.7 Hz), 152.4, 137.8, 137.7, 135.3, (*d*, 131.5, 131.4, *J* = 8 Hz), 131.3, 126.4, (*d*, 114.9, 114.7 *J* = 21.1 Hz), 58.0, 46.7, 34.4, 30.4, 24.2, 24.2; HRMS (DART-ESI^{+}) m/z: $[M+NH_4]^+$ Calcd for $C_{25}H_{37}FNO_3$, 418.2752, Found, 418.2763.

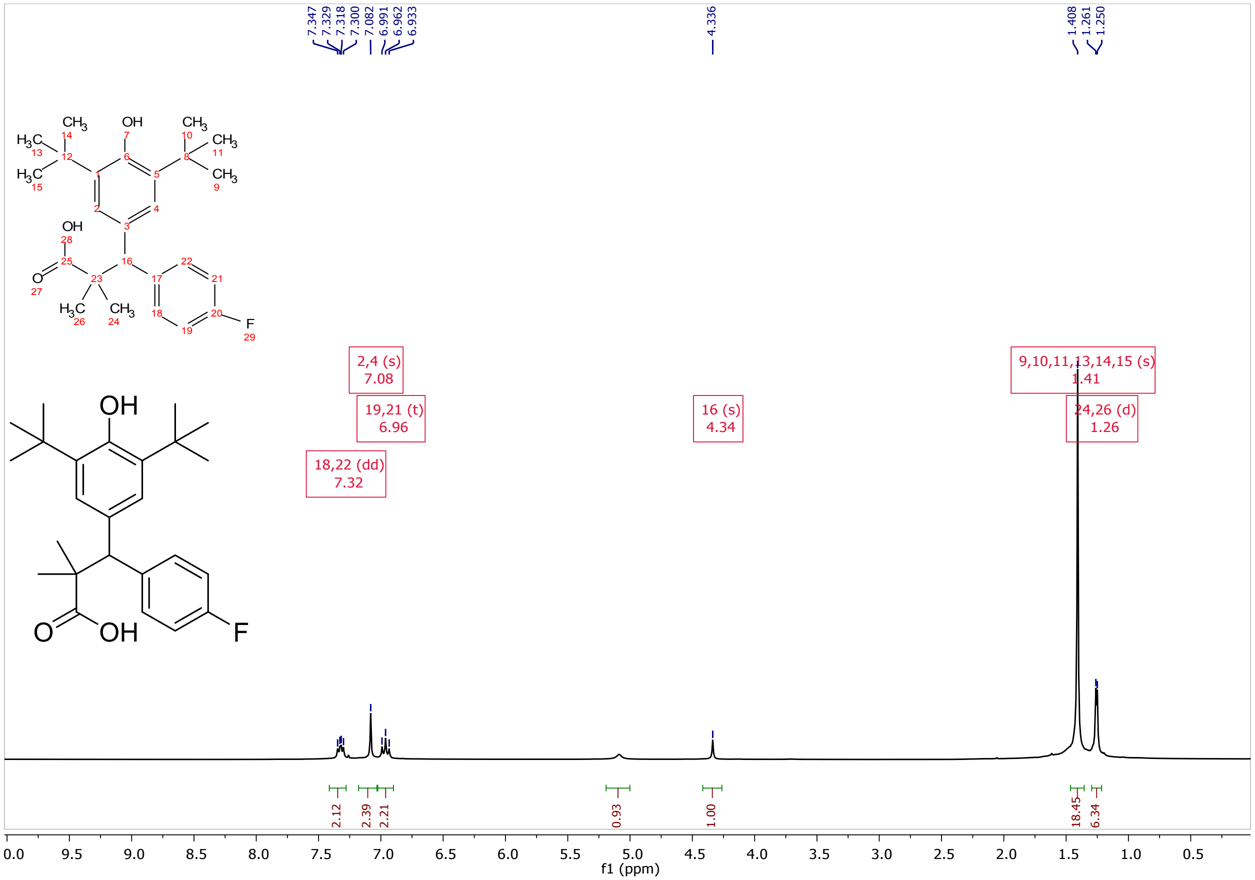

Figure S21. ^{1}H-NMR, 300 MHz, $CDCl_3$ for 3ha

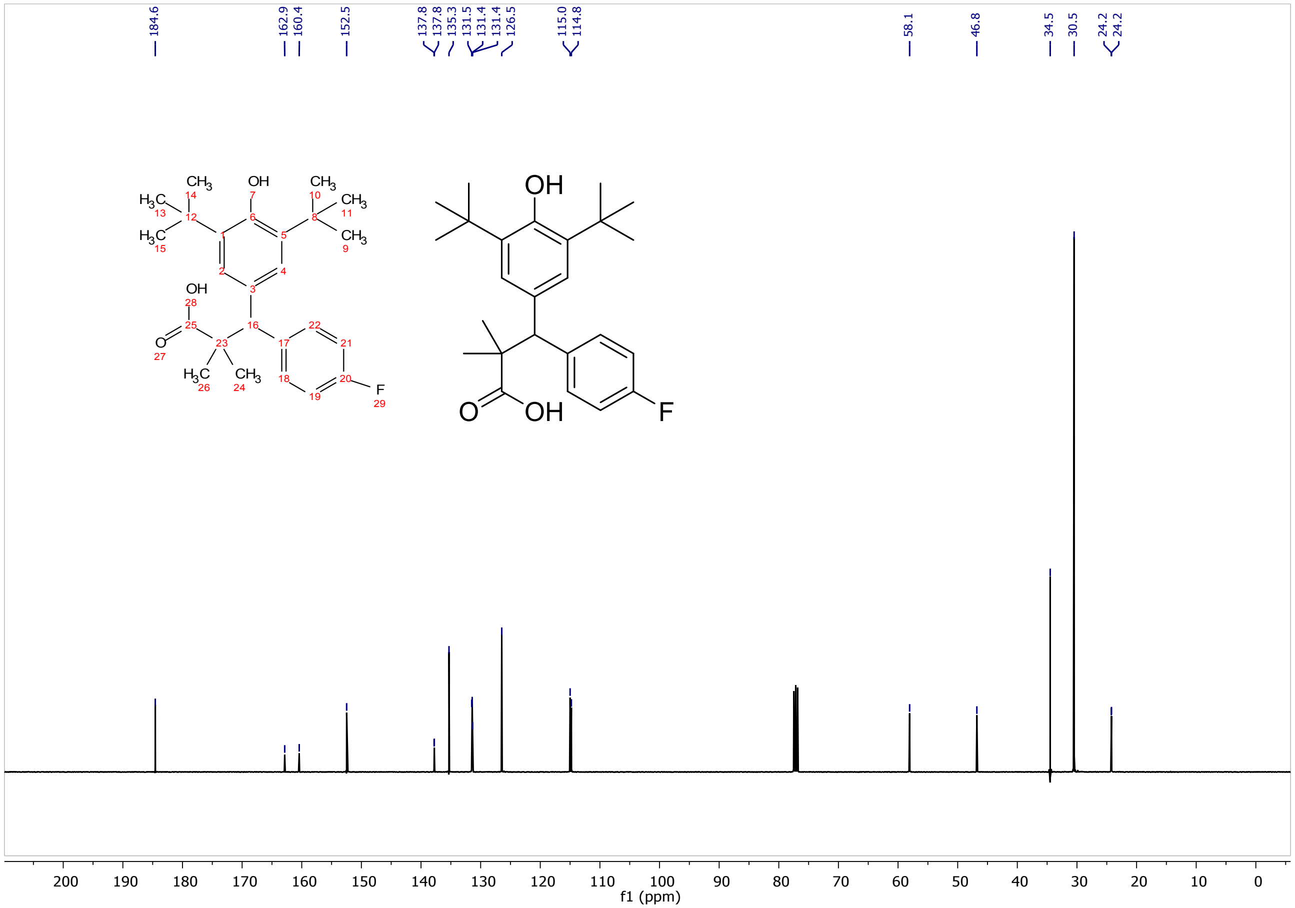

Figure S22. $^{13}C\{^{1}H\}$**-NMR, 75 MHz,** $CDCl_3$ **for 3ha**

Data:456 A-pF
Sample Name:Dr Alvarez Cecilio / Operador Javier Perez
Description:
Ionization Mode:ESI+
History:Determine m/z[Peak Detect[Centroid,30,Area];Correct Base[];Smooth[5]];Correct Base[5.0%];Average(MS[...

Acquired:2/22/2023 7:30:44 AM
Operator:AccuTOF
Mass Calibration data:24enero2023
Created:3/21/2023 3:20:39 PM
Created by:AccuTOF

Charge number:1
Tolerance:2.50(mmu)
Unsaturation Number:0.0 .. 11.0 (Fraction:Both)
Element:^{12}C:0 .. 30, ^{1}H:0 .. 60, ^{19}F:1 .. 1, ^{14}N:1 .. 3, ^{16}O:0 .. 5

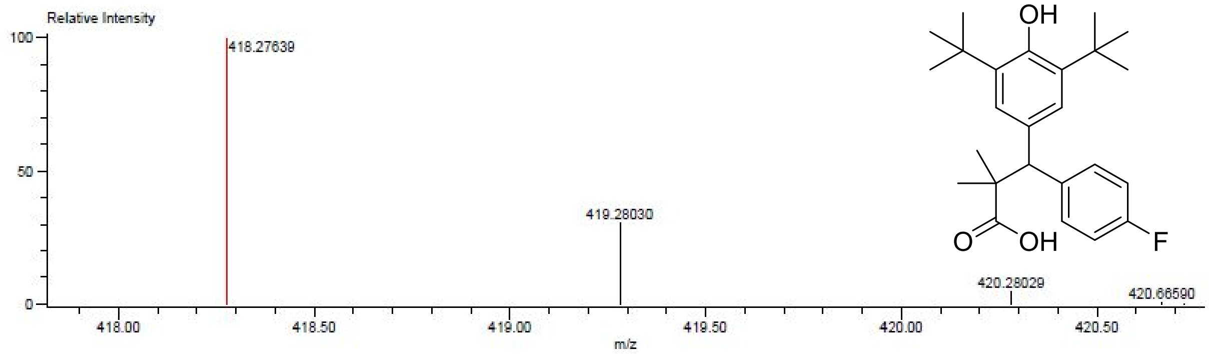

Mass	Intensity	Calc. Mass	Mass Difference (mmu)	Mass Difference (ppm)	Possible Formula	Unsaturation Number
418.27639	7539.80	418.27575	0.64	1.53	$^{12}C_{25}{}^{1}H_{37}{}^{19}F_{1}{}^{14}N_{1}{}^{16}O_{3}$	7.5

Figure S23. HRMS, DART-ESI^{+} (TOF) for 3ha

3-(3,5-di-*tert*-butyl-4-hydroxyphenyl)-2,2-dimethyl-3-(4-(trifluoromethyl)phenyl)propanoic acid (3ia): 202 mg, 90% Yield (White solid), ^{1}H NMR (300 MHz, Chloroform-*d*) δ 7.50 (q, *J* = 8.4 Hz, 4H), 7.07 (d, *J* = 1.1 Hz, 2H), 1.40 (d, *J* = 1.1 Hz, 19H), 1.27 (s, 6H); $^{13}C\{^{1}H\}$ NMR (75 MHz, $CDCl_3$) δ 184.1, 152.6, 152.6, 146.2, 135.5, 130.6, 130.2, (*q,* 129.8, 126.2, 122.6, 119.0, *J* = 271.6 Hz), (*q*, 129.4, 128.9, 128.5, 128.1, *J* = 32.4 Hz) , 126.5, 126.4, (*q,* 125.1, 125.0, 125.0, 124.9, *J* = 3.8 Hz), 58.7, 46.7, 34.5, 30.4, 24.6, 24.1; HRMS (DART-ESI^{+}) m/z: $[M+NH_4]^{+}$ Calcd for $C_{26}H_{37}F_3NO_3$, 468.2720, Found, 468.2726.

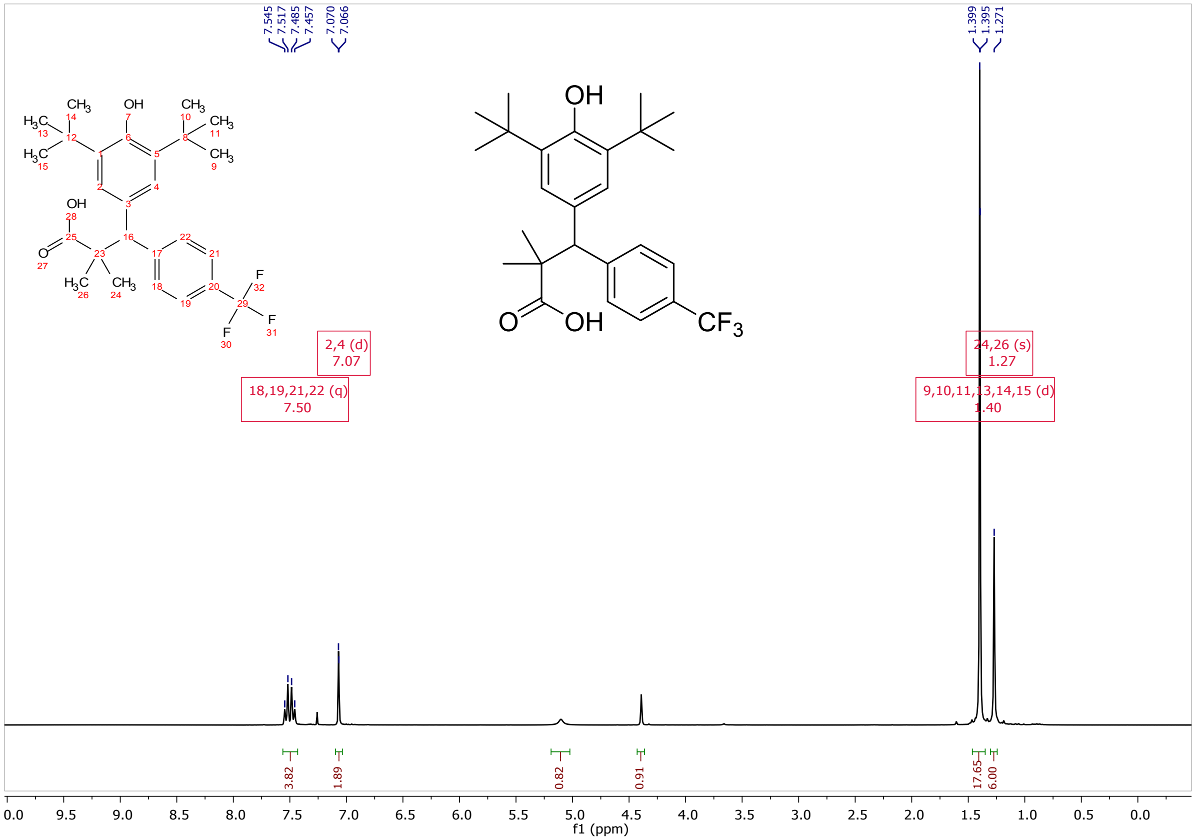

Figure S24. ^{1}H-NMR, 300 MHz, $CDCl_3$ for 3ia

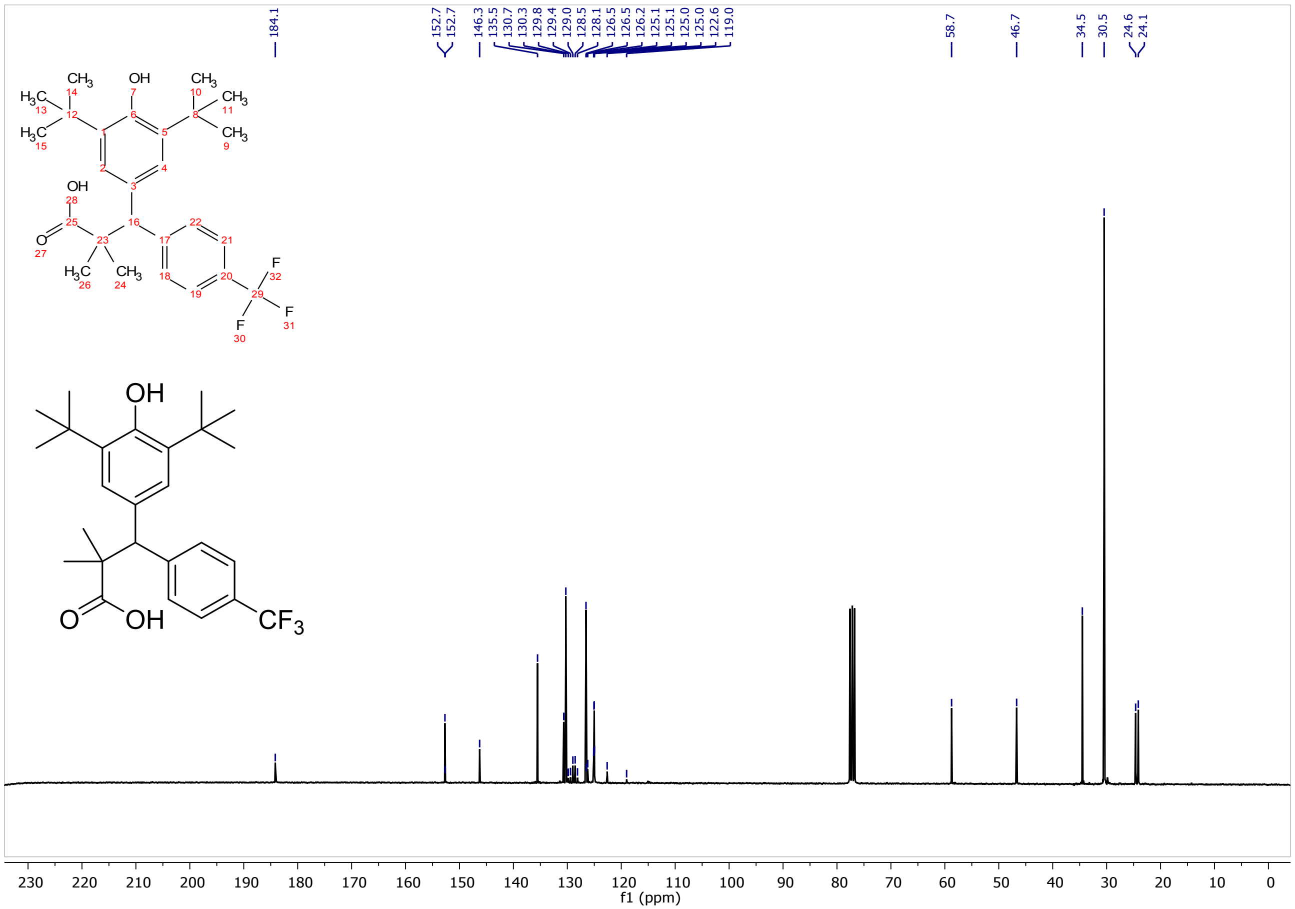

Figure S25. $^{13}C\{^{1}H\}$**-NMR, 75 MHz,** $CDCl_3$ **for 3ia**

Data:457 A-CF3
Sample Name:Dr Alvarez Cecilio / Operador Javier Perez
Description:
Ionization Mode:ESI+
History:Determine m/z[Peak Detect[Centroid,30,Area];Correct Base[];Smooth[5]];Correct Base[5.0%];Average(MS[...

Acquired:2/22/2023 7:34:42 AM
Operator:AccuTOF
Mass Calibration data:24enero2023
Created:3/21/2023 3:12:19 PM
Created by:AccuTOF

Charge number:1
Tolerance:2.50(mmu)
Unsaturation Number:0.0 .. 50.0 (Fraction:Both)
Element:^{12}C:0 .. 30, ^{1}H:0 .. 60, ^{19}F:3 .. 3, ^{14}N:1 .. 3, ^{16}O:0 .. 5

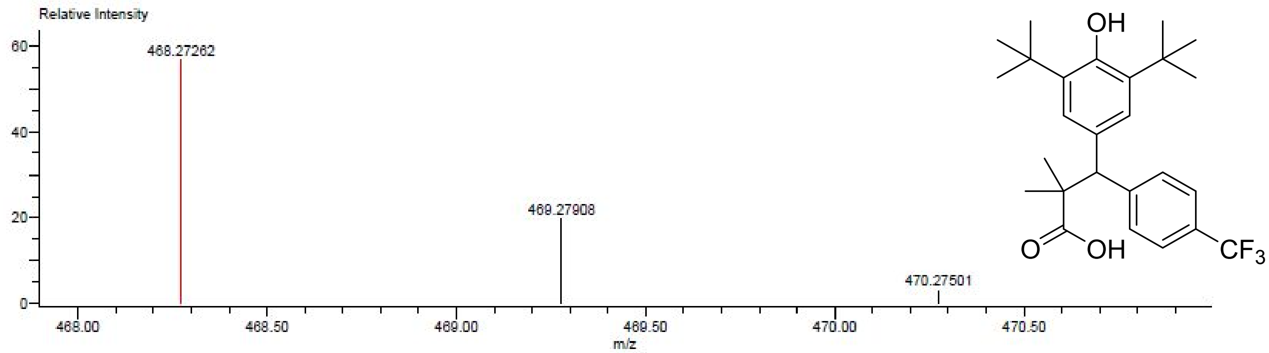

Mass	Intensity	Calc. Mass	Mass Difference (mmu)	Mass Difference (ppm)	Possible Formula	Unsaturation Number
468.27262	21025.36	468.27255	0.06	0.14	$^{12}C_{26}{}^{1}H_{37}{}^{19}F_{3}{}^{14}N_{1}{}^{16}O_{3}$	7.5

Figure S26. HRMS, DART-ESI^{+} (TOF) for 3ia

3-(3,5-di-*tert*-butyl-4-hydroxyphenyl)-2,2-dimethyl-3-(naphthalen-1-yl)propanoic acid (3ja): 137 mg, 63% Yield (White solid), ^{1}H NMR (300 MHz, DMSO-d_6) δ 8.00 – 7.76 (m, 4H), 7.60 – 7.38 (m, 3H), 7.21 (s, 2H), 6.85 (s, 1H), 4.51 (s, 1H), 1.39 (s, 19H), 1.26 (d, J = 5.8 Hz, 6H); ^{13}C{^{1}H} NMR (75 MHz, DMSO) δ 179.0, 152.6, 140.4, 138.8, 133.2, 132.7, 132.0, 128.9, 128.4, 128.1, 127.7, 127.5, 126.4, 126.0, 58.9, 46.2, 35.0, 30.9, 25.3, 24.6; HRMS (DART-ESI$^+$) m/z: $[M+NH_4]^+$ Calcd for $C_{29}H_{40}NO_3$, 450.3003, Found, 450.3008.

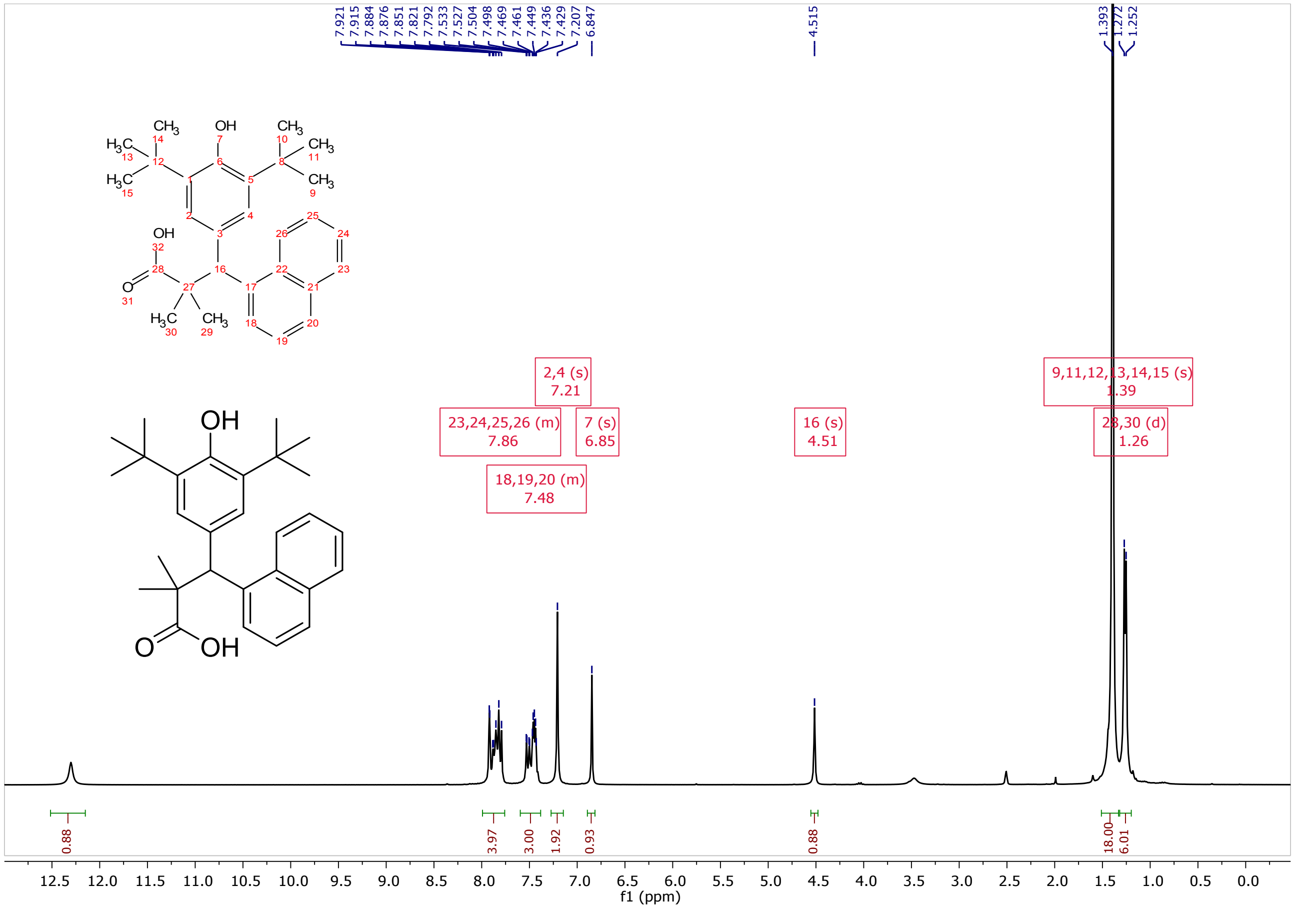

Figure S27. ^{1}**H-NMR, 300 MHz,** $CDCl_3$ **for 3ja**

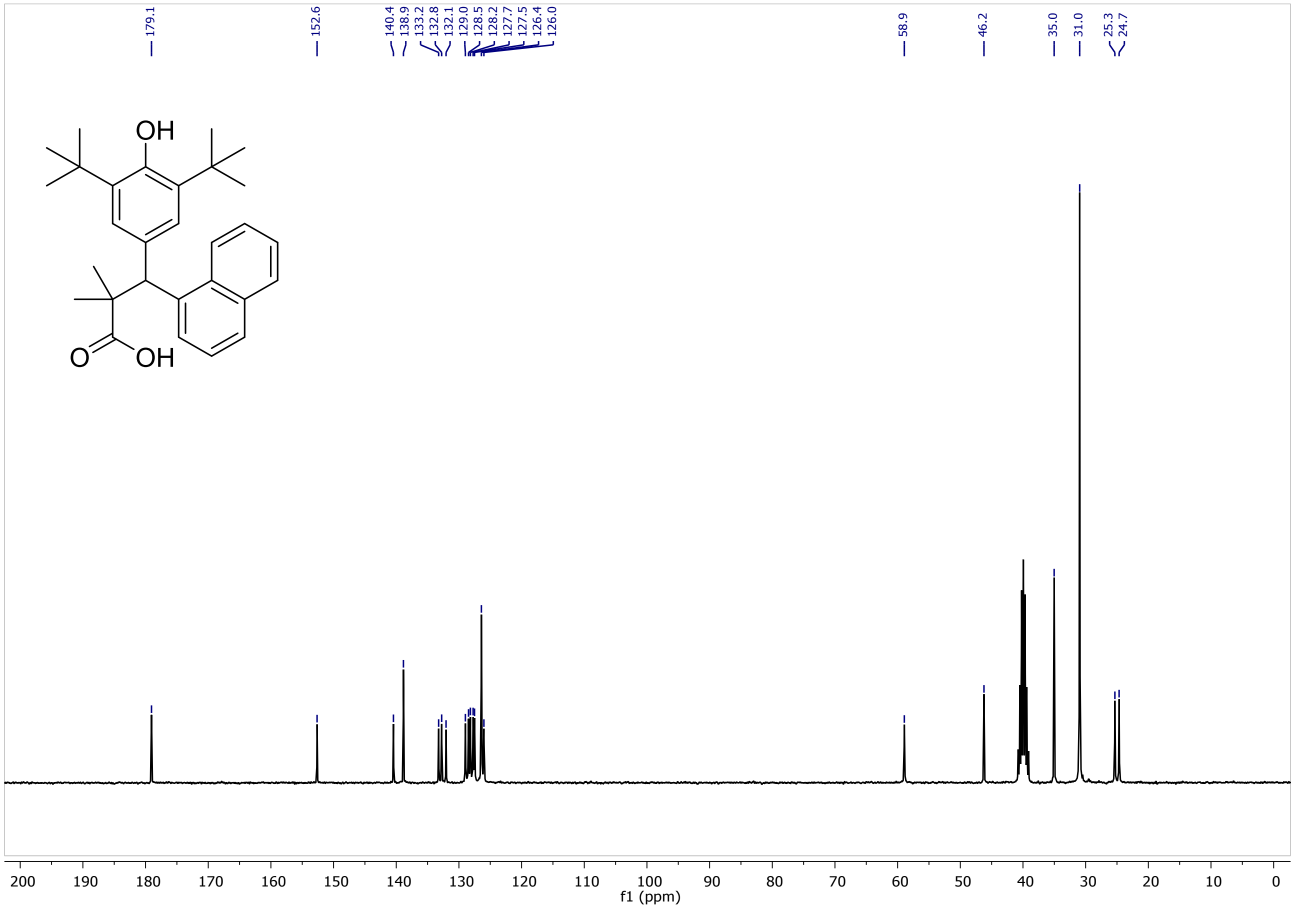

Figure S28. $^{13}C\{^1H\}$**-NMR, 75 MHz,** $CDCl_3$ **for 3ja**

INSTITUTO DE QUIMICA, UNAM
LABORATORIO DE ESPECTROMETRIA DE MASAS

Data:767 A-Naft
Sample Name:Dr Alvarez Cecilio/ Operador Javier Perez
Description:
Ionization Mode:ESI+
History:Determine m/z[Peak Detect[Centroid,30,Area];Correct Base[];Smooth[5]];Correct Base[5.0%];Average(MS[...

Acquired:3/14/2023 5:34:02 PM
Operator:AccuTOF
Mass Calibration data:Cal_PEG_600
Created:3/29/2023 12:17:07 PM
Created by:AccuTOF

Charge number:1
Tolerance:3.00(ppm), 5.00 .. 15.00(mmu)
Unsaturation Number:-1.0 .. 50.0 (Fraction:Both)
Element:^{12}C:0 .. 30, ^{1}H:0 .. 50, ^{14}N:0 .. 3, ^{16}O:0 .. 4

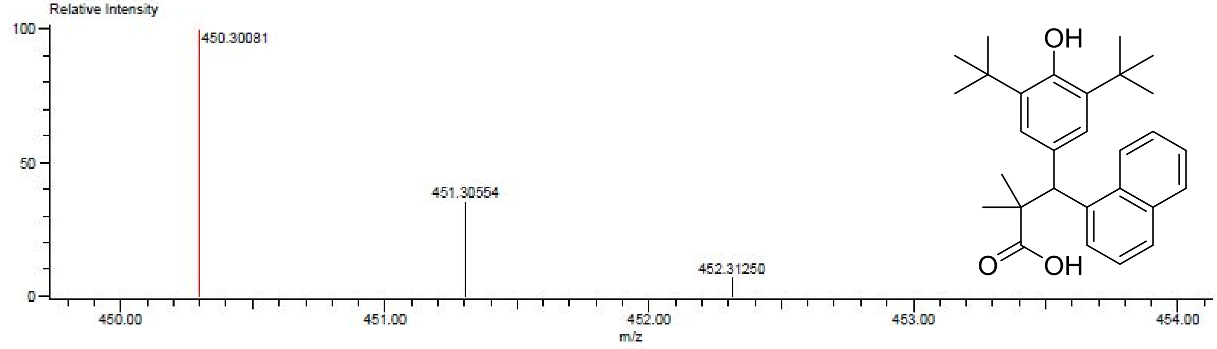

Mass	Intensity	Calc. Mass	Mass Difference (mmu)	Mass Difference (ppm)	Possible Formula	Unsaturation Number
450.30081	238444.63	450.30082	-0.01	-0.03	$^{12}C_{29}{}^{1}H_{40}{}^{14}N_{1}{}^{16}O_{3}$	10.5

Figure S29. **HRMS, DART-ESI⁺ (TOF) for 3ja**

3-(anthracen-9-yl)-3-(3,5-di-*tert*-butyl-4-hydroxyphenyl)-2,2-dimethylpropanoic acid (3la): 236 mg, 98% Yield (White solid), ^{1}H NMR (300 MHz, Chloroform-*d*) δ 7.82 –7.00 (m, 10H), 6.97 (s, 1H), 4.41 (s, 1H), 1.37 (s, 18H), 1.20 (d, *J* = 2.3 Hz, 6H); ^{13}C{^{1}H} NMR (75 MHz, $CDCl_3$) δ 183.6, 153.1, 141.0, 137.1, 136.5, 135.5, 134.8, 134.6, 129.9, 129.4, 128.8, 128.0, 127.0, 126.7, 126.5, 126.4, 126.3, 125.9, 125.5, 123.6, 53.6, 50.9, 34.3, 30.2, 23.2, 22.5; HRMS (DART-ESI$^+$) m/z: $[M+H]^+$ Calcd for $C_{33}H_{39}NO_3$, 483.2894, Found, 483.2918.

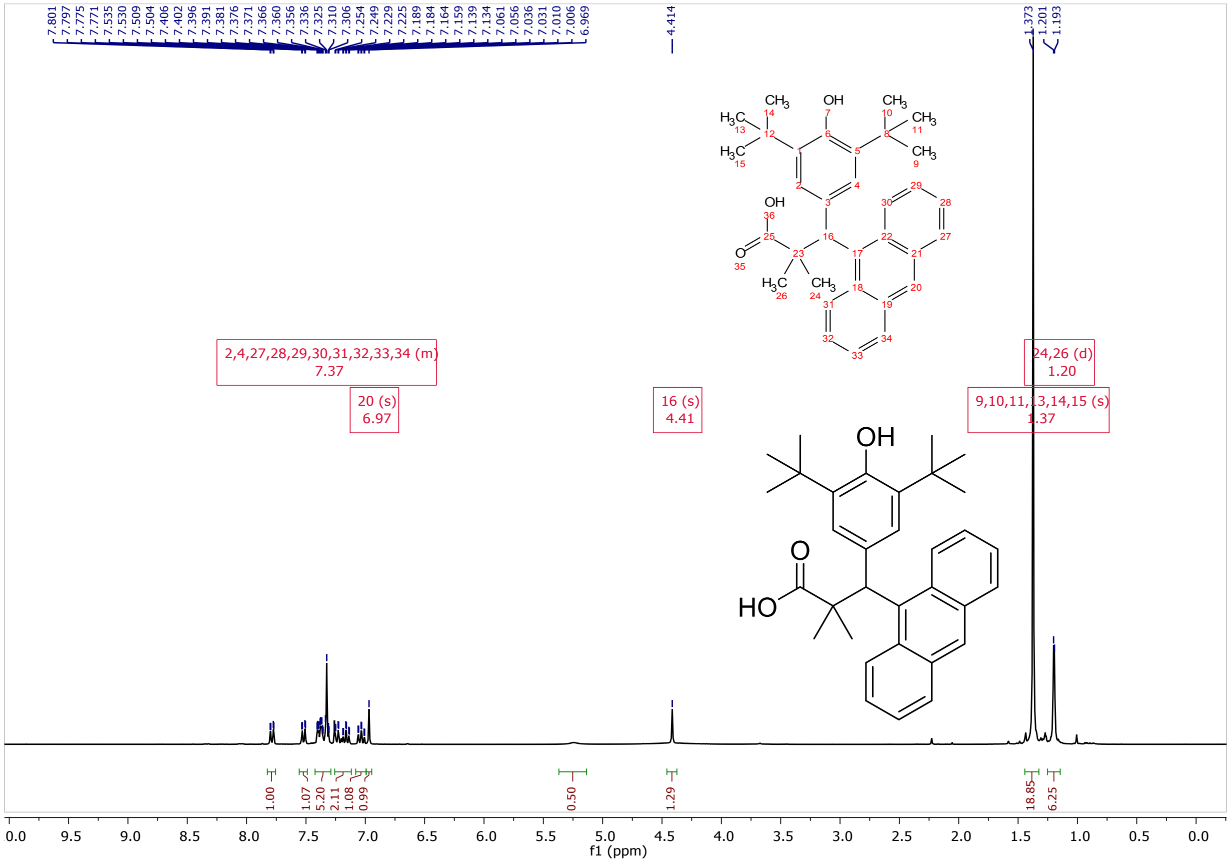

Figure S30. ^{1}**H-NMR, 300 MHz,** $CDCl_3$ **for 3la**

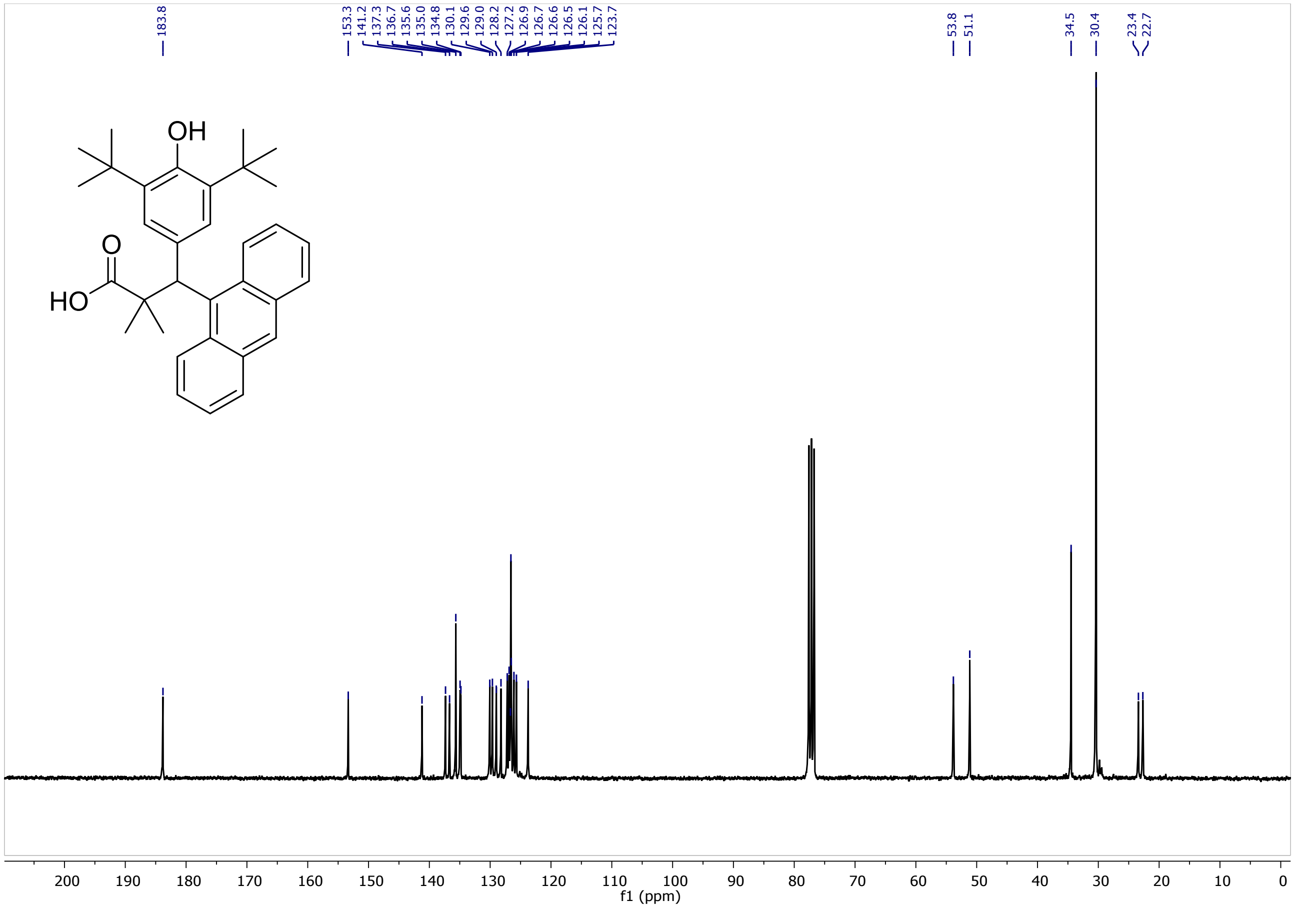

Figure S31. $^{13}C\{^{1}H\}$**-NMR, 75 MHz,** $CDCl_3$ **for 3la**

INSTITUTO DE QUIMICA, UNAM
LABORATORIO DE ESPECTROMETRIA DE MASAS

Data:1018 A-antra
Sample Name:Dr Alvarez Cecilio / Operador Javier Perez
Description:
Ionization Mode:ESI+
History:Determine m/z[Peak Detect[Centroid,30,Area];Correct Base[];Smooth[5]];Correct Base[5.0%];Average(MS[...

Acquired:4/14/2023 12:00:43 AM
Operator:AccuTOF
Mass Calibration data:Cal_PEG_600
Created:4/28/2023 3:52:11 PM
Created by:AccuTOF

Charge number:1
Element:^{12}C:0 .. 40, ^{1}H:0 .. 40, ^{16}O:0 .. 10

Tolerance:3.00(ppm), 5.00 .. 15.00(mmu)

Unsaturation Number:-1.0 .. 50.0 (Fraction:Both)

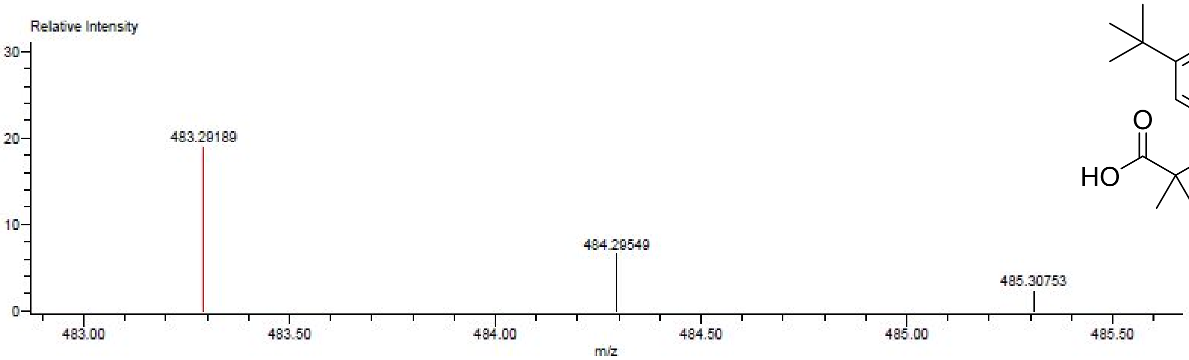

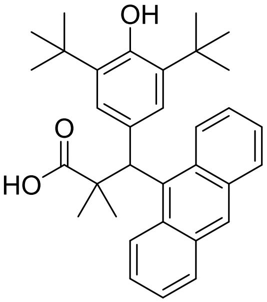

Mass	Intensity	Calc. Mass	Mass Difference (mmu)	Mass Difference (ppm)	Possible Formula	Unsaturation Number
483.29189	28338.65	483.28992	1.97	4.09	$^{12}C_{33}{}^{1}H_{39}{}^{16}O_3$	14.5

Figure S32. HRMS, DART-ESI$^+$ (TOF) for 3la

3-(3,5-di-tert-butyl-4-hydroxyphenyl)-2,2-dimethyl-3-(thiophen-2-yl)propanoic acid (3ma): 128 mg, 66% Yield (White solid), ^{1}H NMR (300 MHz, Chloroform-*d*) δ 7.28 (d, *J* = 2.1 Hz, 2H), 7.21 (dd, *J* = 5.2, 1.3 Hz, 1H), 7.05 (dt, *J* = 3.5, 1.6 Hz, 1H), 6.96 (dd, *J* = 5.1, 3.5 Hz, 1H), 5.15 (s, 1H), 4.75 (s, 1H), 1.48 (d, *J* = 2.1 Hz, 18H), 1.40 – 1.22 (m, 6H); ^{13}C{^{1}H} NMR (75 MHz, $CDCl_3$) δ 184.1, 152.6, 143.8, 135.1, 130.1, 126.9, 126.5, 126.0, 124.1, 54.5, 47.9, 34.3, 30.3, 23.3, 22.9; HRMS (DART-ESI^{+}) m/z: $[M+H]^{+}$ Calcd for $C_{23}H_{33}O_3S$, 389.2145, Found, 389.2148.

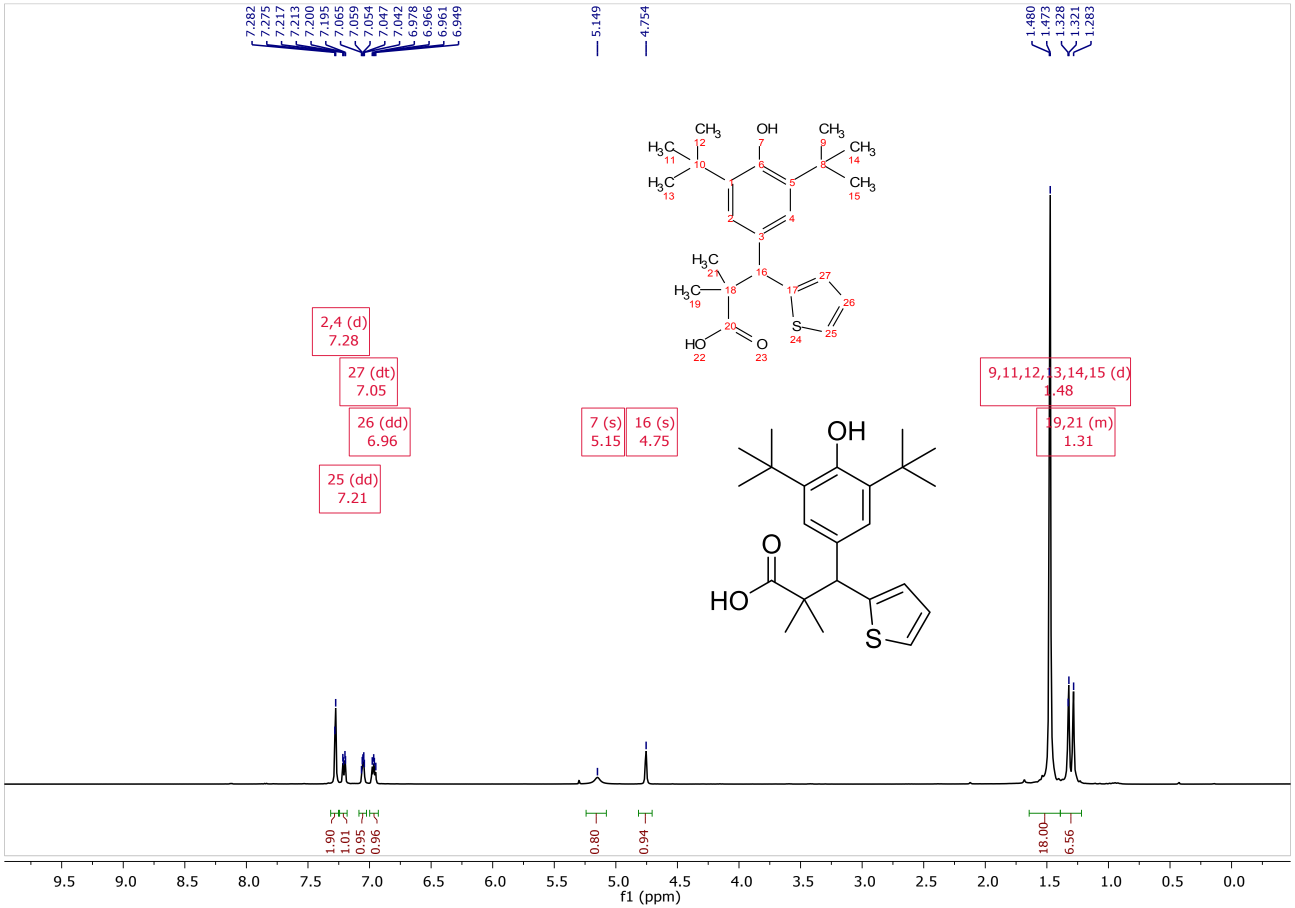

Figure S33. ^{1}H-NMR, 300 MHz, $CDCl_3$ for 3ma

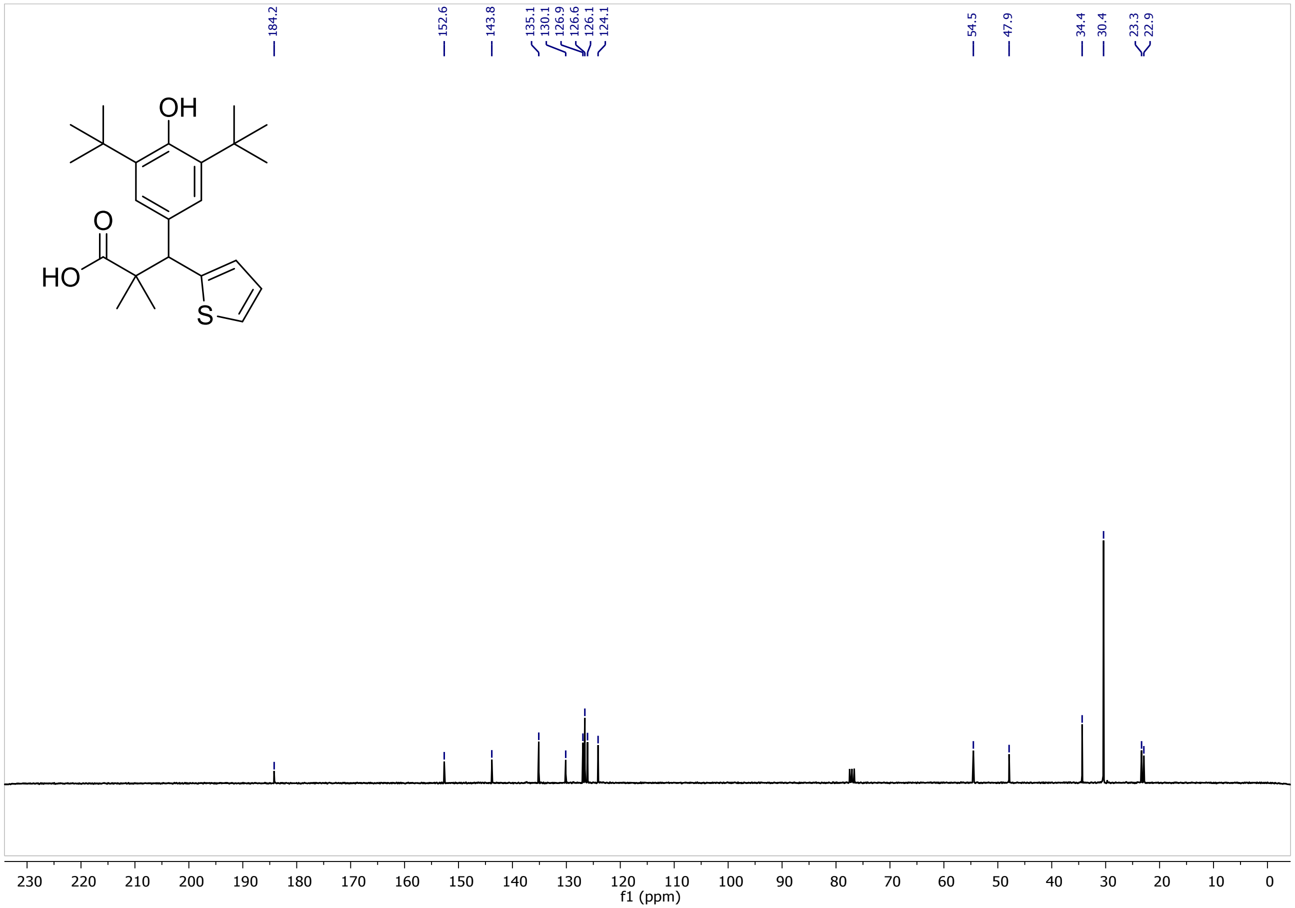

Figure S34. $^{13}C\{^{1}H\}$**-NMR, 75 MHz,** $CDCl_3$ **for 3ma**

INSTITUTO DE QUIMICA, UNAM
LABORATORIO DE ESPECTROMETRIA DE MASAS

Data:769 A-Tiol
Sample Name:Dr Alvarez Cecilio/ Operador Javier Perez
Description:
Ionization Mode:ESI+
History:Determine m/z[Peak Detect[Centroid,30,Area];Correct Base[];Smooth[5]];Correct Base[5.0%];Average(MS[...

Acquired:3/14/2023 5:40:05 PM
Operator:AccuTOF
Mass Calibration data:Cal_PEG_600
Created:3/29/2023 12:09:55 PM
Created by:AccuTOF

Charge number:1
Element:^{12}C:0 .. 40, ^{1}H:0 .. 50, ^{16}O:0 .. 4, ^{32}S:1 .. 1

Tolerance:30.00(ppm), 5.00 .. 15.00(mmu)

Unsaturation Number:-1.0 .. 50.0 (Fraction:Both)

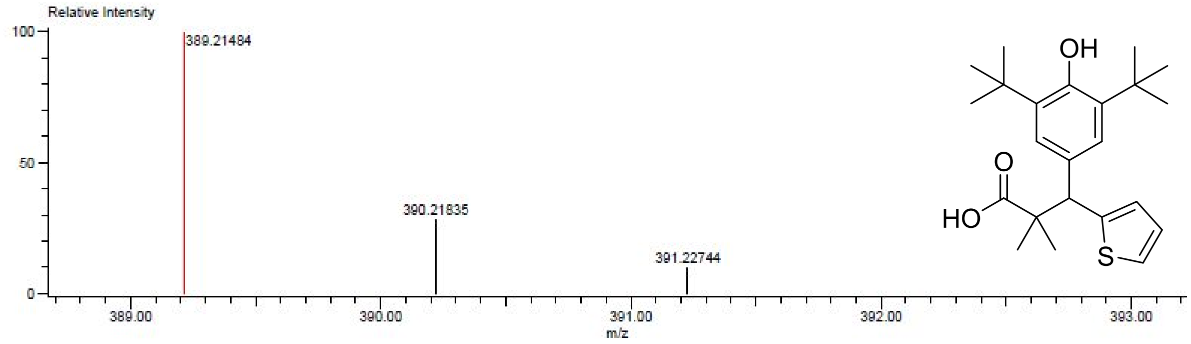

Mass	Intensity	Calc. Mass	Mass Difference (mmu)	Mass Difference (ppm)	Possible Formula	Unsaturation Number
389.21484	93834.56	389.21504	-0.20	-0.51	$^{12}C_{23}{}^{1}H_{33}{}^{16}O_{3}{}^{32}S_{1}$	8.5

Figure S35. HRMS, DART-ESI⁺ (TOF) for 3ma

3-(1,3-benzodioxol-5-yl)-3-(3,5-di-*tert*-butyl-4-hydroxyphenyl)-2,2-dimethylpropanoic acid (3na): 211 mg, 99% Yield (White solid), ^{1}H NMR (300 MHz, Chloroform-*d*) δ 7.09 (s, 2H), 6.87 – 6.79 (m, 2H), 6.71 (d, *J* = 7.9 Hz, 1H), 5.91 (s, 2H), 4.25 (s, 1H), 1.40 (s, 18H), 1.25 (d, *J* = 1.3 Hz, 6H); $^{13}C\{^{1}H\}$ NMR (76 MHz, CHLOROFORM-*D*) δ 184.3, 152.3, 147.3, 146.0, 135.8, 135.2, 131.6, 126.3, 123.1, 110.5, 107.8, 100.9, 58.5, 46.8, 34.4, 30.4, 24.4, 24.0; HRMS (DART-ESI^{+}) m/z: $[M+NH_4]^+$ Calcd for $C_{26}H_{38}NO_5$, 444.2744, Found, 444.2745.

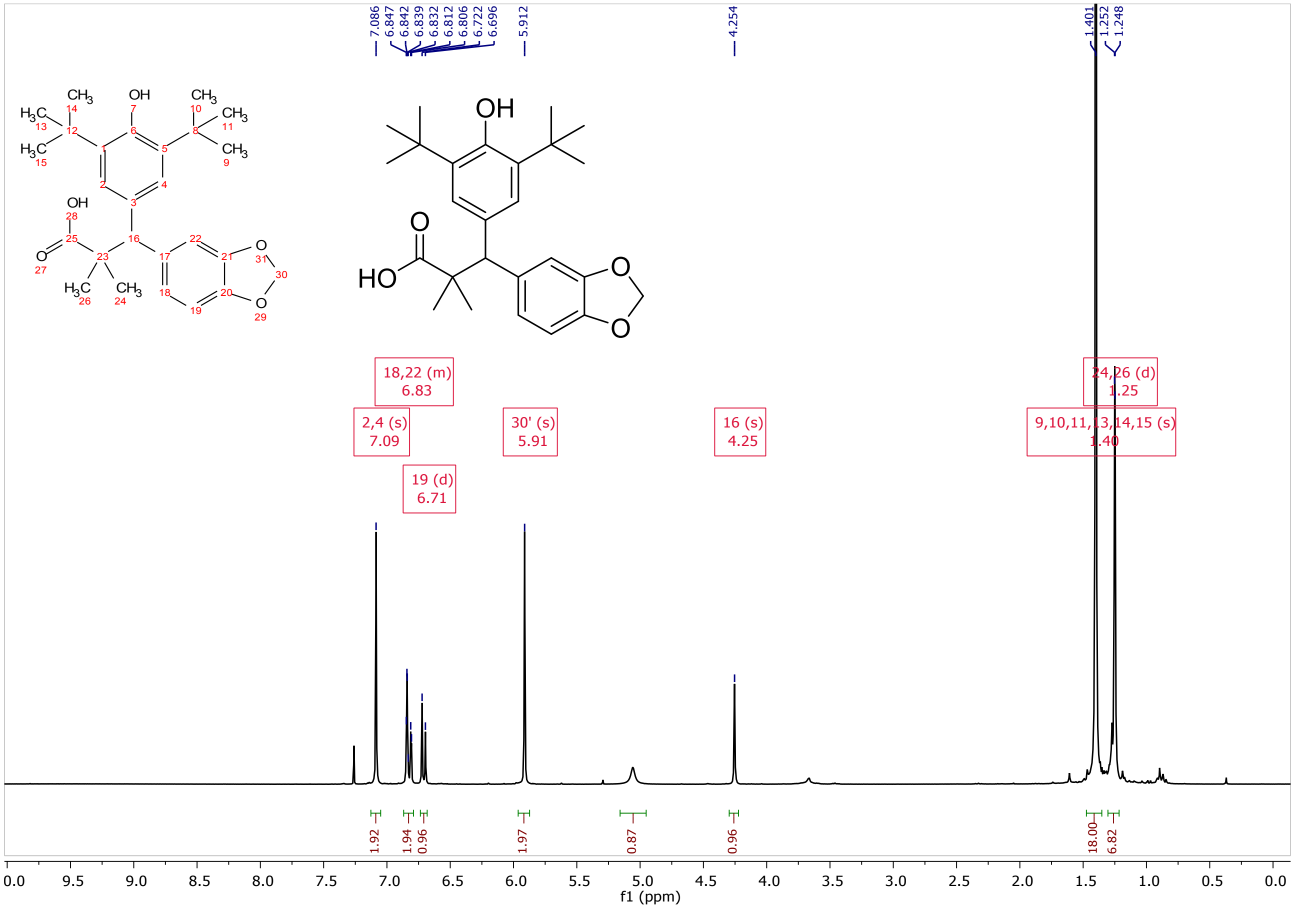

Figure S36. ^{1}H-NMR, 300 MHz, $CDCl_3$ for 3na

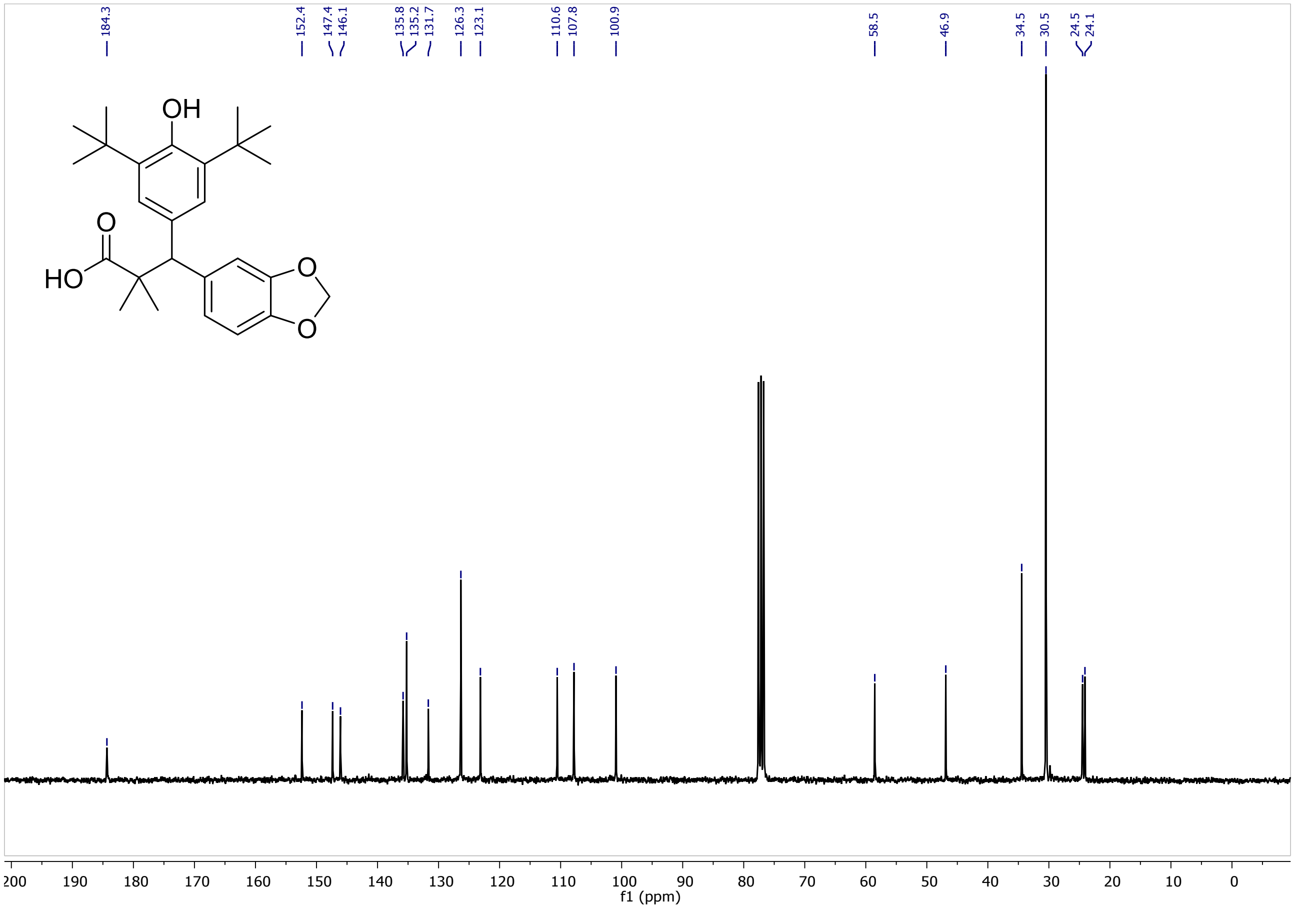

Figure S37. $^{13}C\{^{1}H\}$-NMR, 75 MHz, $CDCl_3$ for 3na

INSTITUTO DE QUIMICA, UNAM
LABORATORIO DE ESPECTROMETRIA DE MASAS

Data:771 A-dioxo
Sample Name:Dr Alvarez Cecilio/ Operador Javier Perez
Description:
Ionization Mode:ESI+
History:Determine m/z[Peak Detect[Centroid,30,Area];Correct Base[];Smooth[5]];Correct Base[5.0%];Average(MS[...

Acquired:3/14/2023 5:47:03 PM
Operator:AccuTOF
Mass Calibration data:Cal_PEG_600
Created:3/29/2023 12:01:50 PM
Created by:AccuTOF

Charge number:1
Element:^{12}C:0 .. 30, ^{1}H:0 .. 40, ^{14}N:0 .. 3, ^{16}O:4 .. 5

Tolerance:3.00(ppm), 5.00 .. 15.00(mmu)

Unsaturation Number:-1.0 .. 50.0 (Fraction:Both)

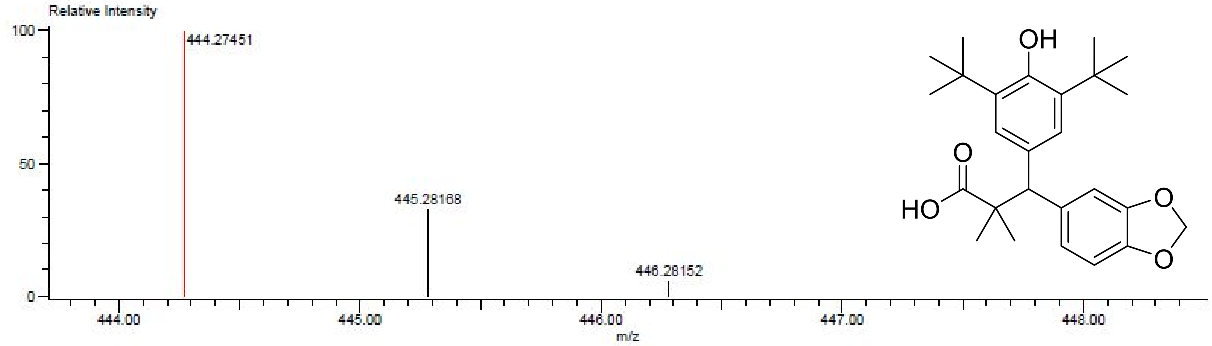

Mass	Intensity	Calc. Mass	Mass Difference (mmu)	Mass Difference (ppm)	Possible Formula	Unsaturation Number
444.27451	33928.25	444.27500	-0.49	-1.09	$^{12}C_{25}{}^{1}H_{38}{}^{14}N_{1}{}^{16}O_{5}$	8.5

Figure S38. HRMS, DART-ESI⁺ (TOF) for 3na

3-(ferrocenyl)-3-(3,5-di-*tert*-butyl-4-hydroxyphenyl)-2,2-dimethylpropanoic acid (3oa): 110 mg, 45% Yield (red solid), ^{1}H NMR (300 MHz, Chloroform-*d*) δ 7.20 (s, 2H), 5.10 (s, 1H), 4.25 – 3.99 (m, 4H), 3.87 (s, 1H), 3.66 (s, 4H), 1.49 (s, 18H), 1.17 – 1.01 (m, 6H); $^{13}C\{^{1}H\}$ NMR (75 MHz, $CDCl_3$) δ 152.2, 134.8, 132.3, 126.5, 88.8, 71.3, 68.7, 68.4, 66.3, 54.0, 47.9, 34.3, 30.5, 29.7, 28.8, 23.8, 22.3; HRMS (DART-ESI^{+}) m/z: $[M+NH_4]^+$ Calcd for $C_{29}H_{39}FeO_3$, 491.2243, Found, 491.2238.

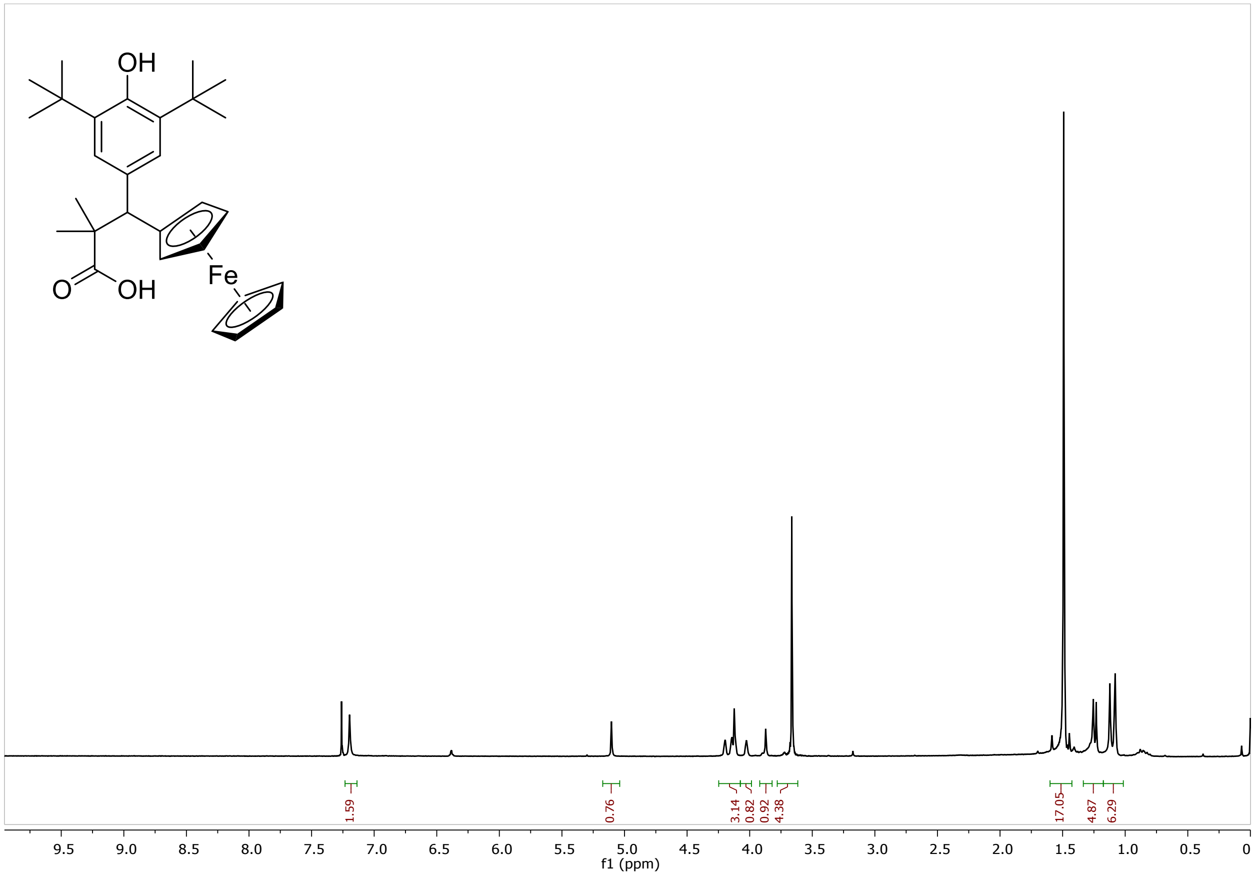

Figure S39. ^{1}H-NMR, 300 MHz, $CDCl_3$ for 3oa

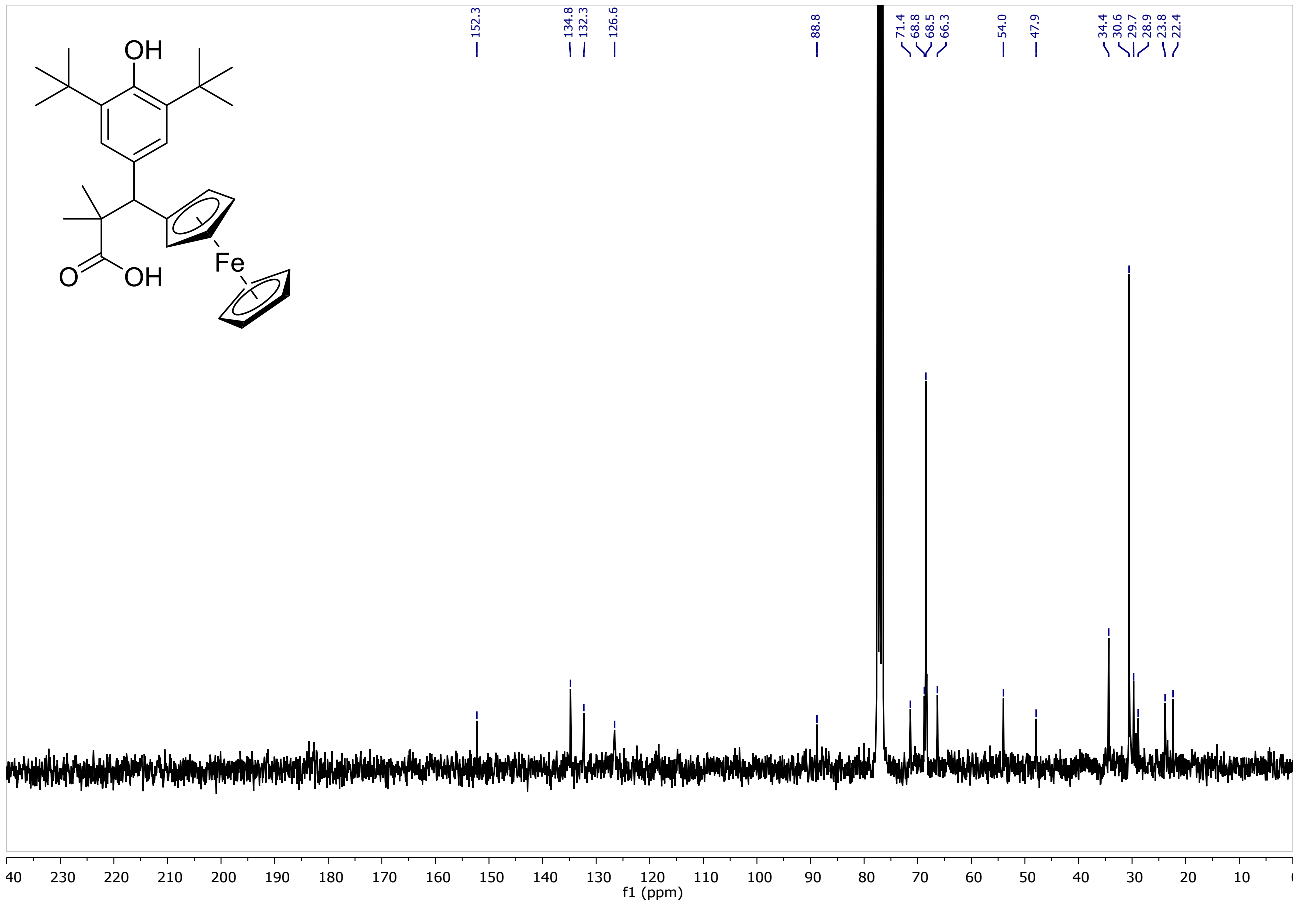

Figure S40. $^{13}C\{^{1}H\}$-NMR, 75 MHz, $CDCl_3$ for 3oa

Data:780 A-Fe
Sample Name:Dr Alvarez Cecilio/ Operador Javier Perez
Description:
Ionization Mode:ESI+
History:Determine m/z[Peak Detect[Centroid,30,Area];Correct Base[];Smooth[5]];Correct Base[5.0%];Average(MS[...

Acquired:3/14/2023 6:16:05 PM
Operator:AccuTOF
Mass Calibration data:24enero2023
Created:3/30/2023 1:38:30 PM
Created by:AccuTOF

Charge number:1
Tolerance:5.00(ppm), 5.00 .. 15.00(mmu)
Unsaturation Number:-1.0 .. 50.0 (Fraction:.5)
Element:^{12}C:0 .. 30, ^{1}H:0 .. 60, ^{56}Fe:1 .. 1, ^{14}N:0 .. 3, ^{16}O:0 .. 4

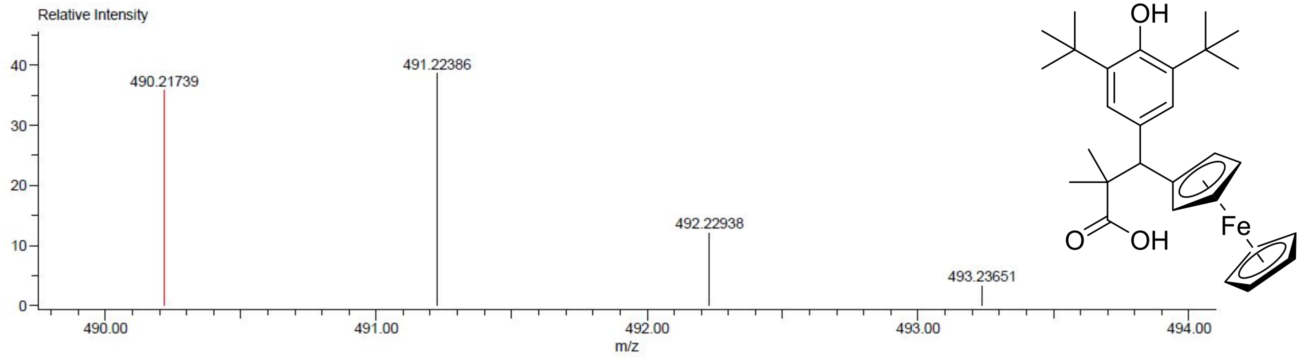

Mass	Intensity	Calc. Mass	Mass Difference (mmu)	Mass Difference (ppm)	Possible Formula	Unsaturation Number
490.21739	44526.47	490.21704	0.35	0.72	$^{12}C_{29}{}^{1}H_{38}{}^{56}Fe_{1}{}^{16}O_{3}$	11.5

Figure S41. HRMS, DART-ESI^{+} (TOF) for 3oa

1-((3,5-di-*tert*-butyl-4-hydroxyphenyl)(4-methoxyphenyl)methyl)cyclobutanecarboxylic acid (3cb): 127 mg, 60% Yield (White solid), ^{1}H NMR (300 MHz, Chloroform-*d*) δ 7.10 (d, *J* = 8.3 Hz, 2H), 6.97 (s, 2H), 6.77 (d, *J* = 8.2 Hz, 2H), 4.30 (s, 1H), 3.74 (s, 3H), 2.57 – 1.59 (m, 6H), 1.38 (s, 18H), $^{13}C\{^{1}H\}$ NMR (75 MHz, $CDCl_3$) δ 183.1, 157.8, 152.0, 135.1, 134.9, 132.6, 130.4, 126.1, 113.3, 55.1, 54.3, 34.3, 30.3, 28.5, 28.0, 16.1; HRMS (DART-ESI^{+}) m/z: $[M+NH_4]^{+}$ Calcd for $C_{27}H_{40}NO_4$, 442.2952, Found, 442.2970.

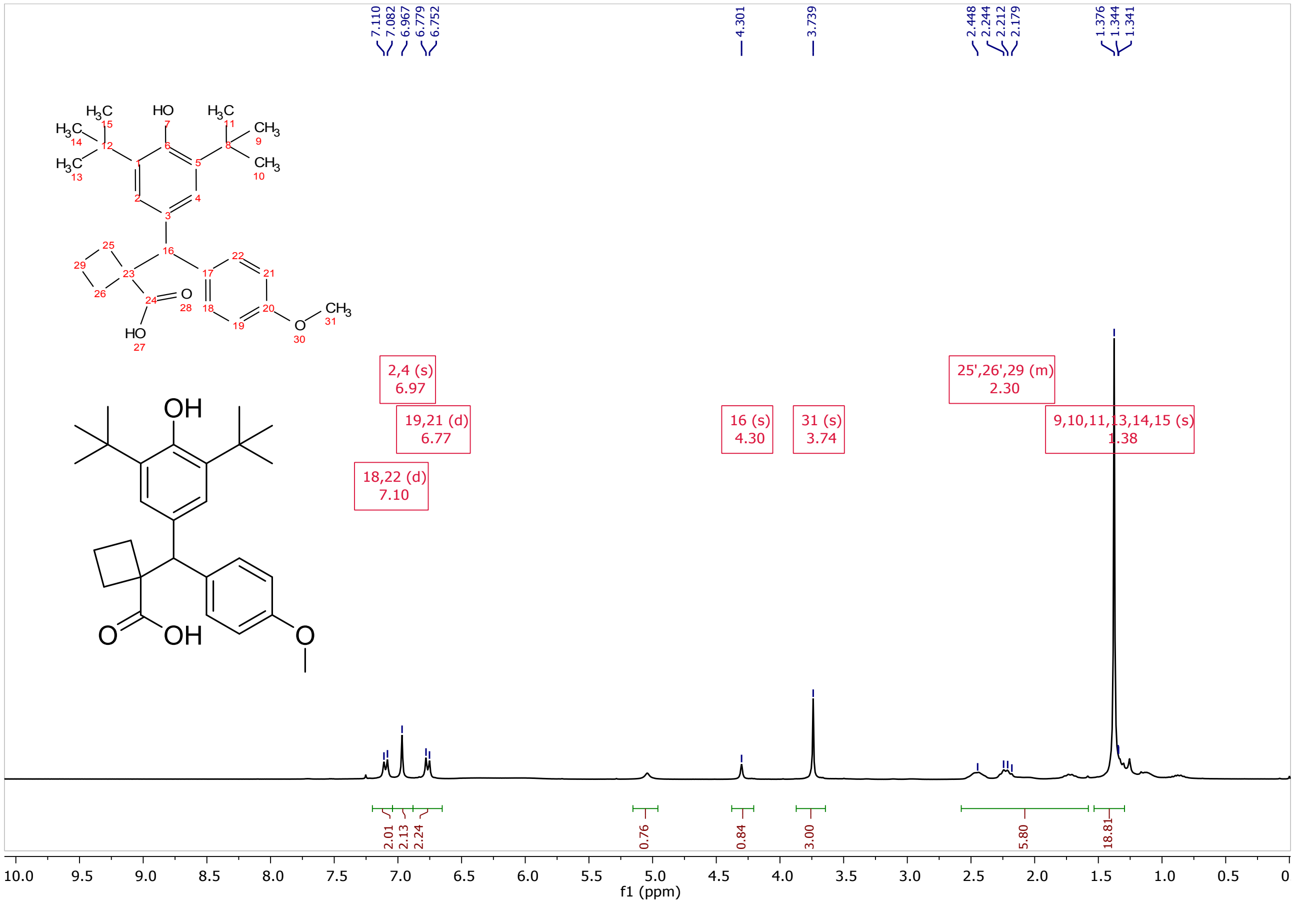

Figure S42. ^{1}**H-NMR, 300 MHz,** $CDCl_3$ **for 3cb**

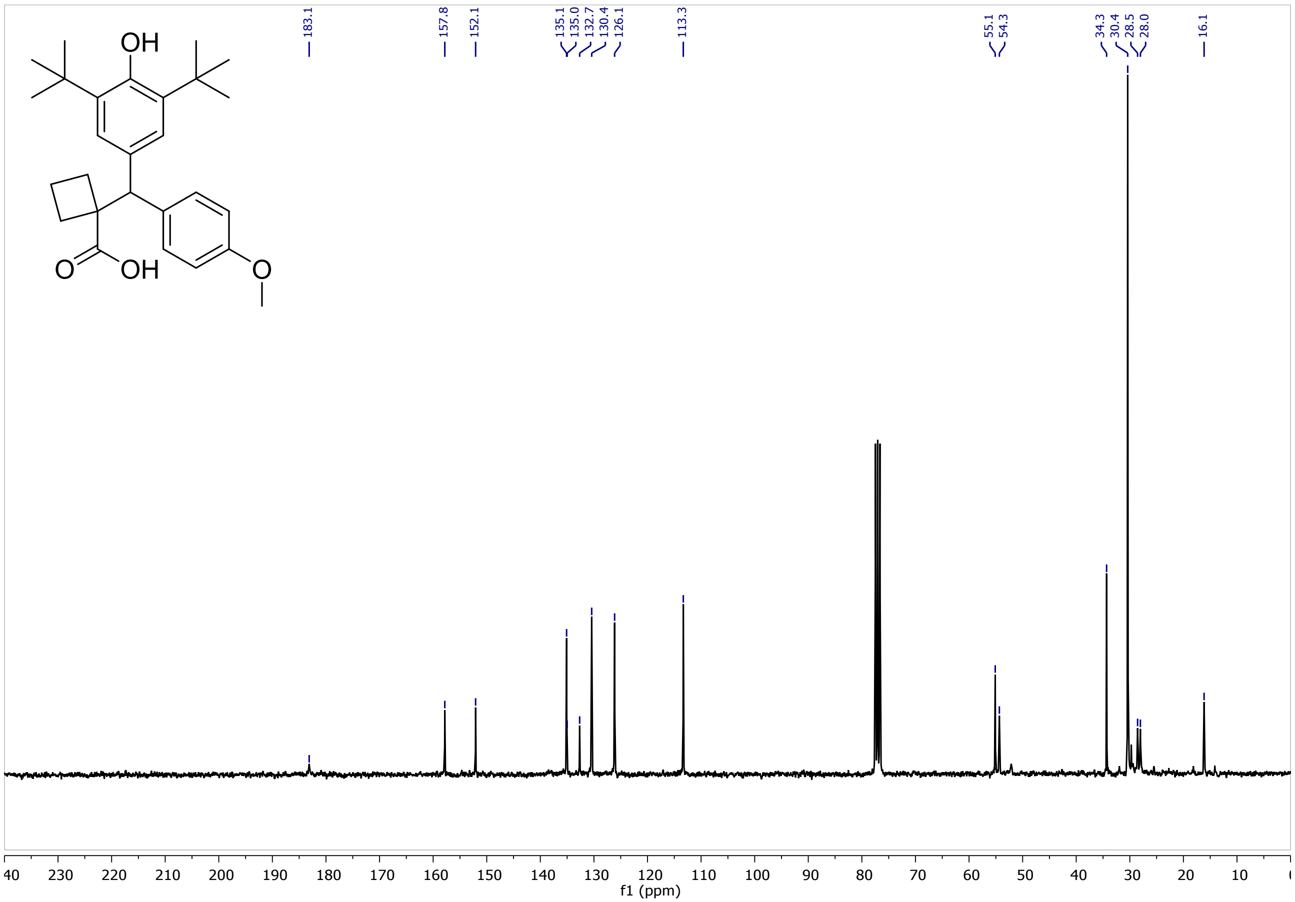

Figure S43. $^{13}C\{^{1}H\}$**-NMR, 75 MHz,** $CDCl_3$ **for 3cb**

Data:U-913 EN-CButOMe
Sample Name:M. en C. Saulo C. Rosales
Description:
Ionization Mode:ESI+
History:Determine m/z[Peak Detect[Centroid,30,Area];Correct Base[5.0%]];Correct Base[5.0%];Average(MS[1] 1..1)

Acquired:8/6/2024 12:09:26 PM
Operator:AccuTOF
Mass Calibration data:CAL_PEG_600_ALUMNOS_2024
Created:8/7/2024 12:22:06 PM
Created by:AccuTOF

Charge number:1
Tolerance:3.00(ppm), 5.00 .. 15.00(mmu)
Unsaturation Number:-1.5 .. 100.0 (Fraction:Both)
Element:^{12}C:0 .. 28, ^{1}H:0 .. 50, ^{14}N:0 .. 1, ^{16}O:0 .. 4

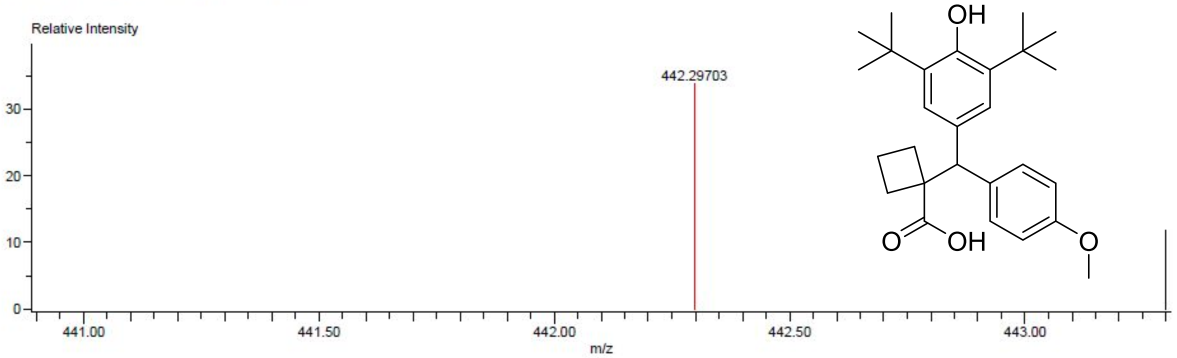

Mass	Intensity	Calc. Mass	Mass Difference (mmu)	Mass Difference (ppm)	Possible Formula	Unsaturation Number
442.29703	28604.92	442.29573	1.30	2.94	$^{12}C_{27}{}^{1}H_{40}{}^{14}N_{1}{}^{16}O_{4}$	8.5

Figure S44. HRMS, DART-ESI^{+} (TOF) for 3cb

1-((3,5-di-*tert*-butyl-4-hydroxyphenyl)(4-methoxyphenyl)methyl)cyclopentanecarboxylic acid (3cc): 122 mg, 56% Yield (White solid), ^{1}H NMR (300 MHz, Chloroform-*d*) δ 7.32 – 7.13 (m, 2H), 7.03 (s, 2H), 6.77 (d, *J* = 8.7 Hz, 2H), 4.43 (s, 1H), 3.75 (s, 3H), 2.40 – 1.65 (m, 4H), 1.37 (s, 22H); ^{13}C{^{1}H} NMR (75 MHz, $CDCl_3$) δ 183.9, 157.9, 152.0, 135.0, 134.5, 132.4, 130.9, 126.3, 113.2, 58.5, 56.2, 55.1, 34.3, 34.2, 33.8, 30.4, 24.5, 24.4; HRMS (DART-ESI$^+$) m/z: $[M+NH_4]^+$ Calcd for $C_{28}H_{42}NO_4$, 456.3108, Found, 456.3101.

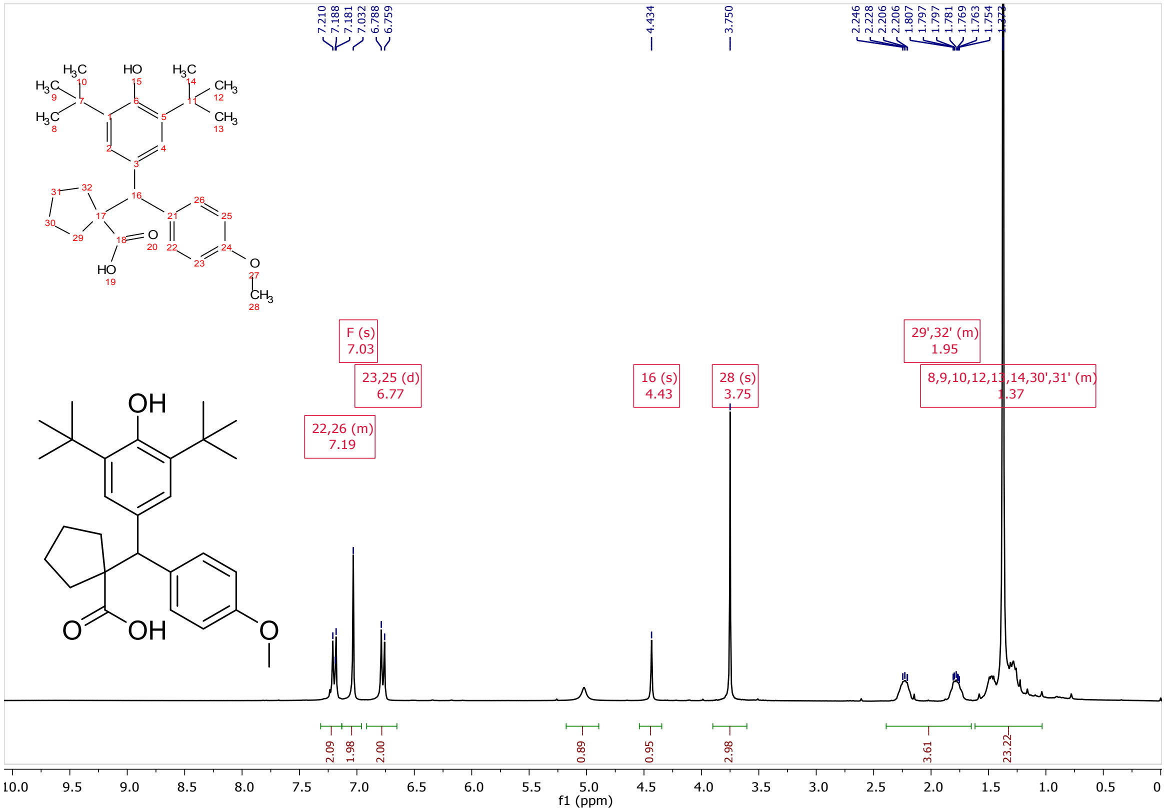

Figure S45. ^{1}**H-NMR, 300 MHz,** $CDCl_3$ **for 3cc**

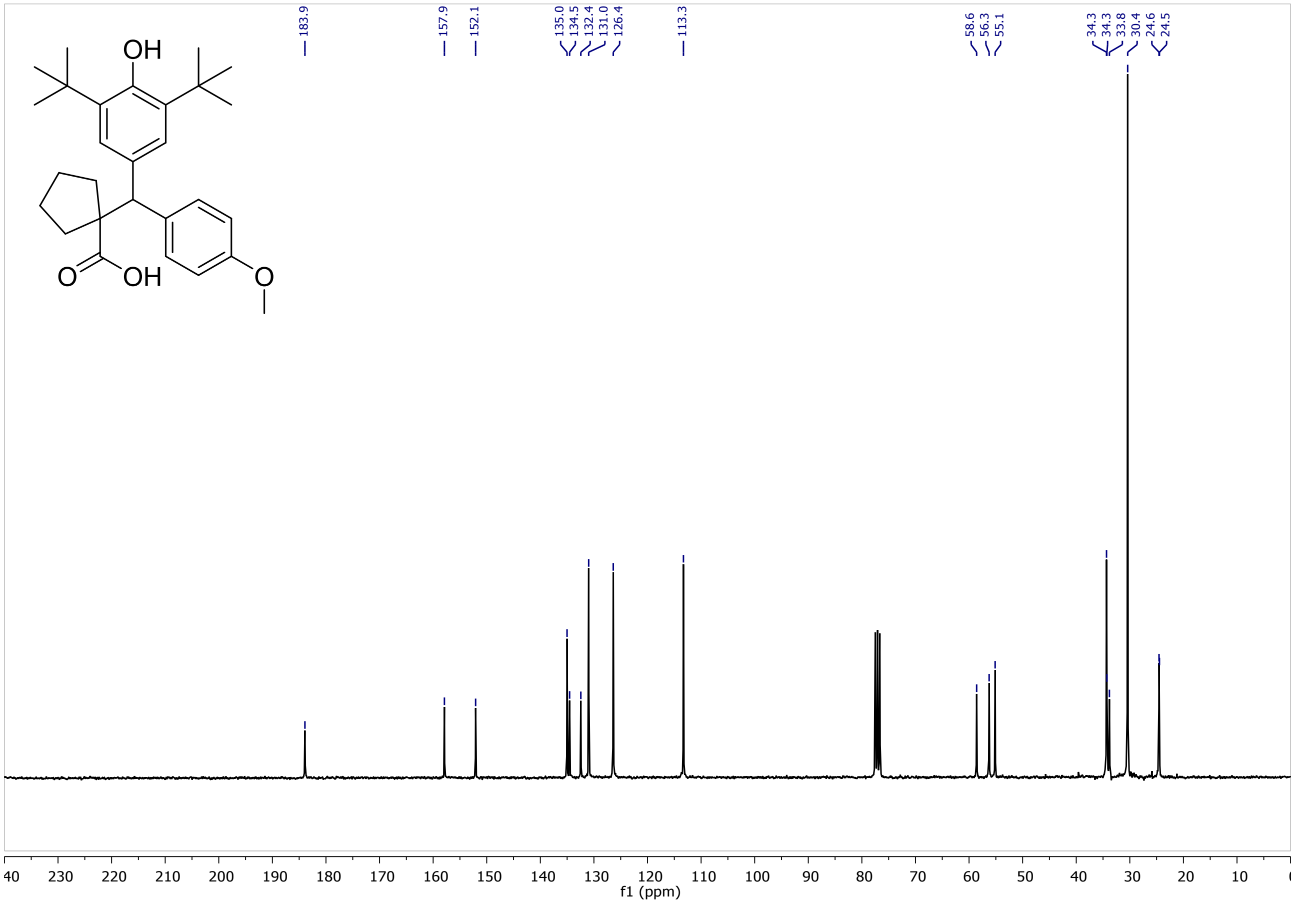

Figure S46. $^{13}C\{^{1}H\}$-NMR, 75 MHz, $CDCl_3$ for 3cc

Data:U-916 EN-CPenOMe
Sample Name:M. en C. Saulo C. Rosales
Description:
Ionization Mode:ESI+
History:Determine m/z[Peak Detect[Centroid,30,Area];Correct Base[5.0%]];Correct Base[5.0%];Average(MS[1] 1..1)

Acquired:8/6/2024 12:19:22 PM
Operator:AccuTOF
Mass Calibration data:CAL_PEG_600_ALUMNOS_2024
Created:8/7/2024 12:30:03 PM
Created by:AccuTOF

Charge number:1
Element:^{12}C:0 .. 30, ^{1}H:0 .. 43, ^{14}N:0 .. 1, ^{16}O:0 .. 4

Tolerance:3.00(ppm), 5.00 .. 15.00(mmu)

Unsaturation Number:-1.5 .. 100.0 (Fraction:Both)

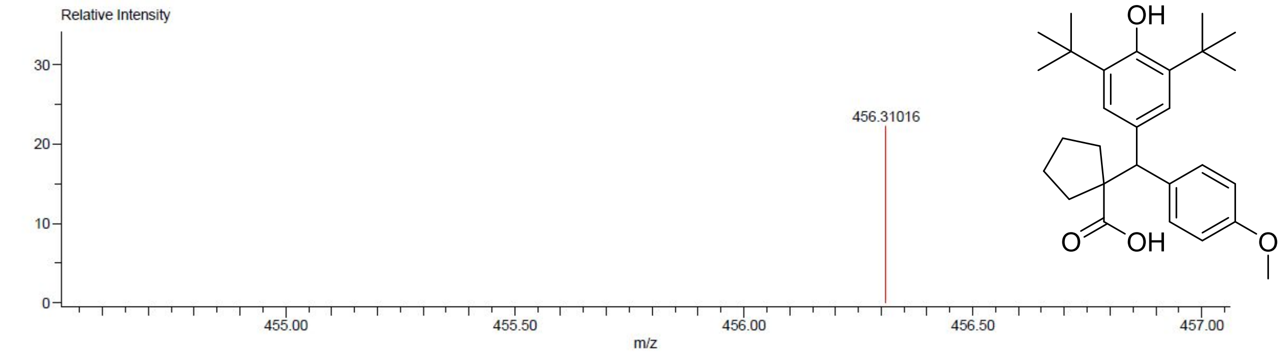

Mass	Intensity	Calc. Mass	Mass Difference (mmu)	Mass Difference (ppm)	Possible Formula	Unsaturation Number
456.31016	19404.49	456.31138	-1.22	-2.67	$^{12}C_{28}{}^{1}H_{42}{}^{14}N_{1}{}^{16}O_{4}$	8.5

Figure S47. HRMS, DART-ESI^{+} (TOF) for 3cc

3-(3,5-di-*tert*-butyl-4-hydroxyphenyl)-2,2-dimethyl-3-(1-methyl-1*H*-indol-3-yl)propanoic acid (3pa): 80 mg, 37% Yield (White solid), ^{1}H NMR (300 MHz, Chloroform-*d*) δ 7.52 (d, *J* = 7.9 Hz, 1H), 7.33 – 7.10 (m, 5H), 7.02 (ddd, *J* = 8.1, 6.8, 1.2 Hz, 1H), 4.68 (s, 1H), 3.76 (s, 3H), 1.32 (s, 18H), 1.32 – 1.20 (m, 6H); ^{13}C{^{1}H} NMR (75 MHz, $CDCl_3$) δ 183.3, 152.1, 136.1, 134.7, 131.2, 129.0, 126.5, 126.4, 121.4, 119.5, 118.6, 114.9, 108.8, 53.7, 49.5, 47.2, 34.2, 32.8, 31.7, 30.3, 29.7, 29.2, 24.3, 23.1; HRMS (DART-ESI$^+$) m/z: $[M+NH_4]^+$ Calcd for $C_{28}H_{38}NO_3$, 436.2846, Found, 436.2819.

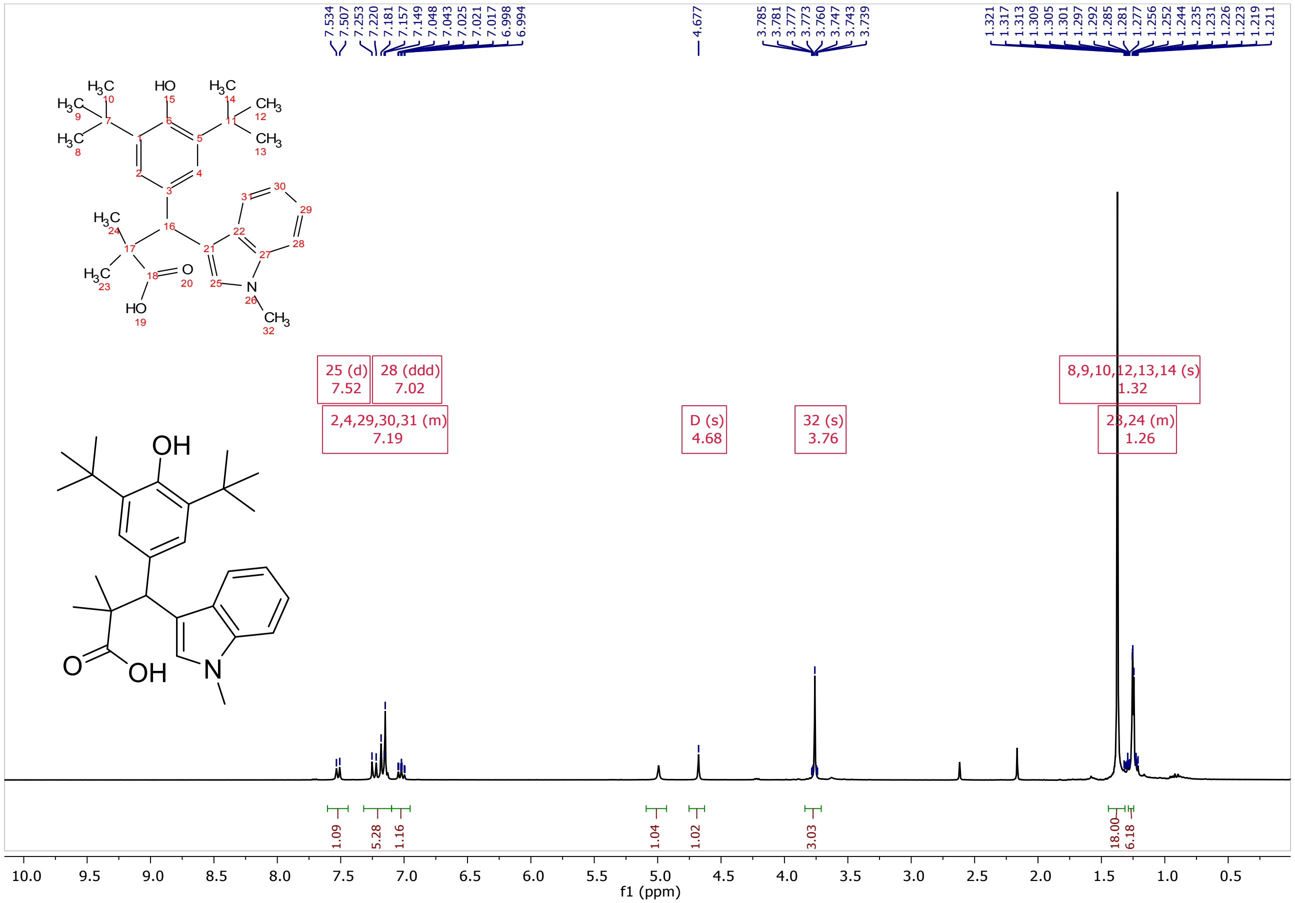

Figure S48. ^{1}**H-NMR, 300 MHz,** $CDCl_3$ **for 3pa**

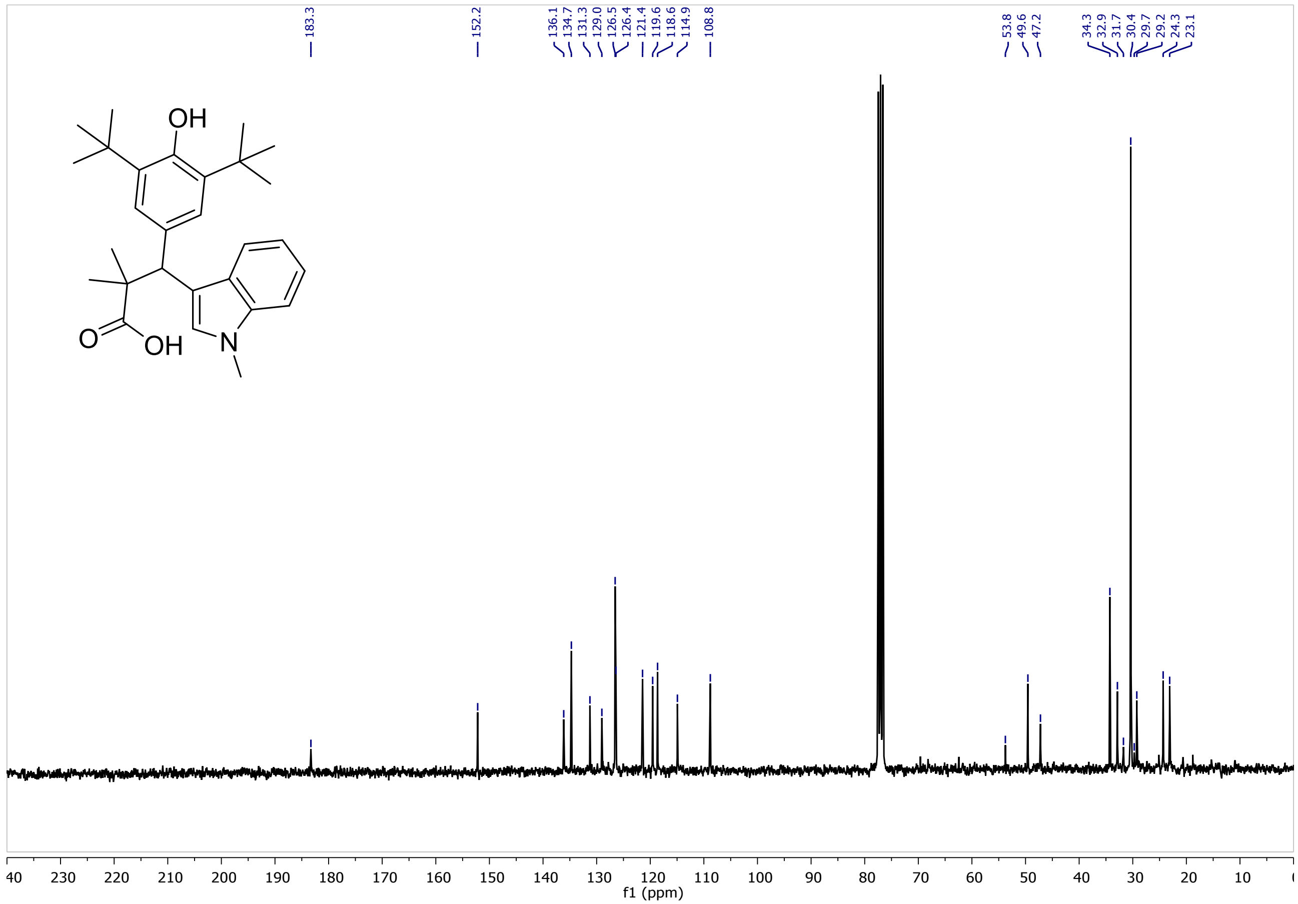

Figure S49. $^{13}C\{^{1}H\}$-NMR, 75 MHz, $CDCl_3$ for 3pa

Data:U-943 EN-Indol
Sample Name:M. en C. Saulo C. Rosales
Description:
Ionization Mode:ESI+
History:Determine m/z[Peak Detect[Centroid,30,Area];Correct Base[5.0%]];Correct Base[5.0%];Average(MS[1] 1..1)

Acquired:8/8/2024 12:22:51 PM
Operator:AccuTOF
Mass Calibration data:CAL_PEG_600_ALUMNOS_2024
Created:8/14/2024 11:22:17 AM
Created by:AccuTOF

Charge number:1
Tolerance:5.00(mmu)
Unsaturation Number:-1.5 .. 40.0 (Fraction:Both)
Element:^{12}C:26 .. 28, ^{1}H:0 .. 50, ^{14}N:0 .. 2, ^{16}O:0 .. 5

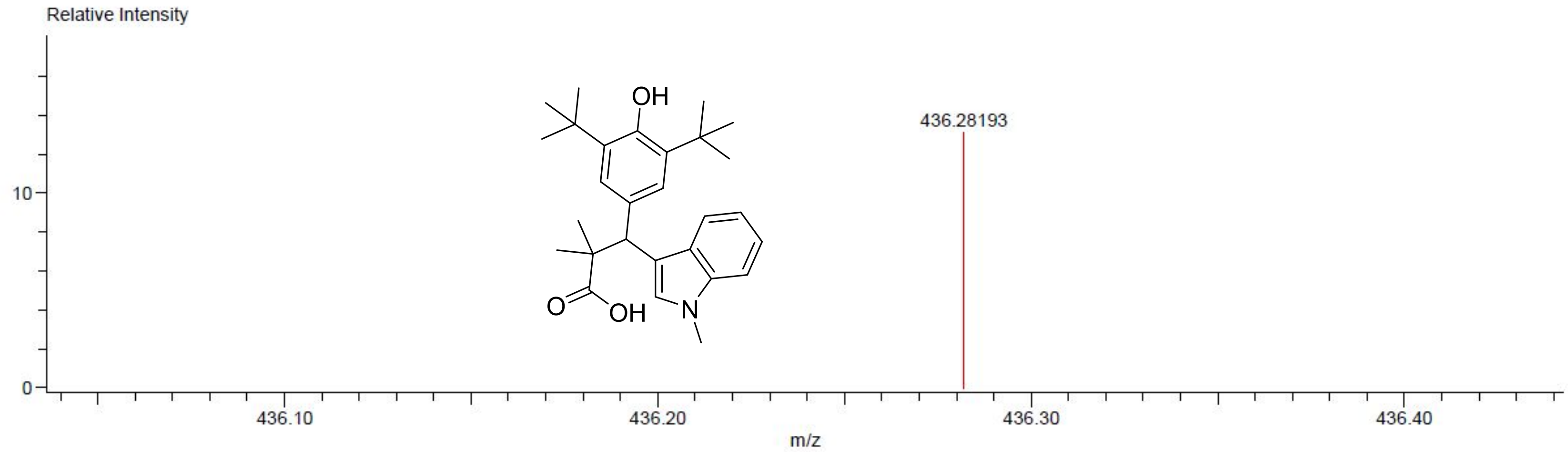

Mass	Intensity	Calc. Mass	Mass Difference (mmu)	Mass Difference (ppm)	Possible Formula	Unsaturation Number
436.28193	4964.44	436.28517	-3.23	-7.41	$^{12}C_{28}{}^{1}H_{38}{}^{14}N_{1}{}^{16}O_{3}$	10.5

Figure S50. HRMS, DART-ESI⁺ (TOF) for 3pa

1-((3,5-di-*tert*-butyl-4-hydroxyphenyl)(4-methylphenyl)methyl)cyclohexanecarboxylic acid (3bd): 70 mg, 32% Yield (White solid), ^{1}H NMR (300 MHz, Chloroform-*d*) δ 7.30 (d, *J* = 8.0 Hz, 2H), 7.16 (s, 2H), 7.05 (d, *J* = 7.8 Hz, 2H), 5.01 (s, 1H), 3.92 (s, 1H), 2.28 (s, 3H), 2.23 – 2.00 (m, 2H), 1.74 – 1.14 (m, 26H); $^{13}C\{^{1}H\}$ NMR (75 MHz, $CDCl_3$) δ 181.7, 152.3, 138.3, 135.9, 134.8, 131.1, 129.9, 128.6, 126.4, 62.1, 52.5, 34.3, 33.1, 32.9, 30.3, 25.5, 23.6, 20.9; HRMS (DART-ESI^{+}) m/z: $[M+NH_4]^{+}$ Calcd for $C_{29}H_{44}NO_3$, 454.3316, Found, 454.3311.

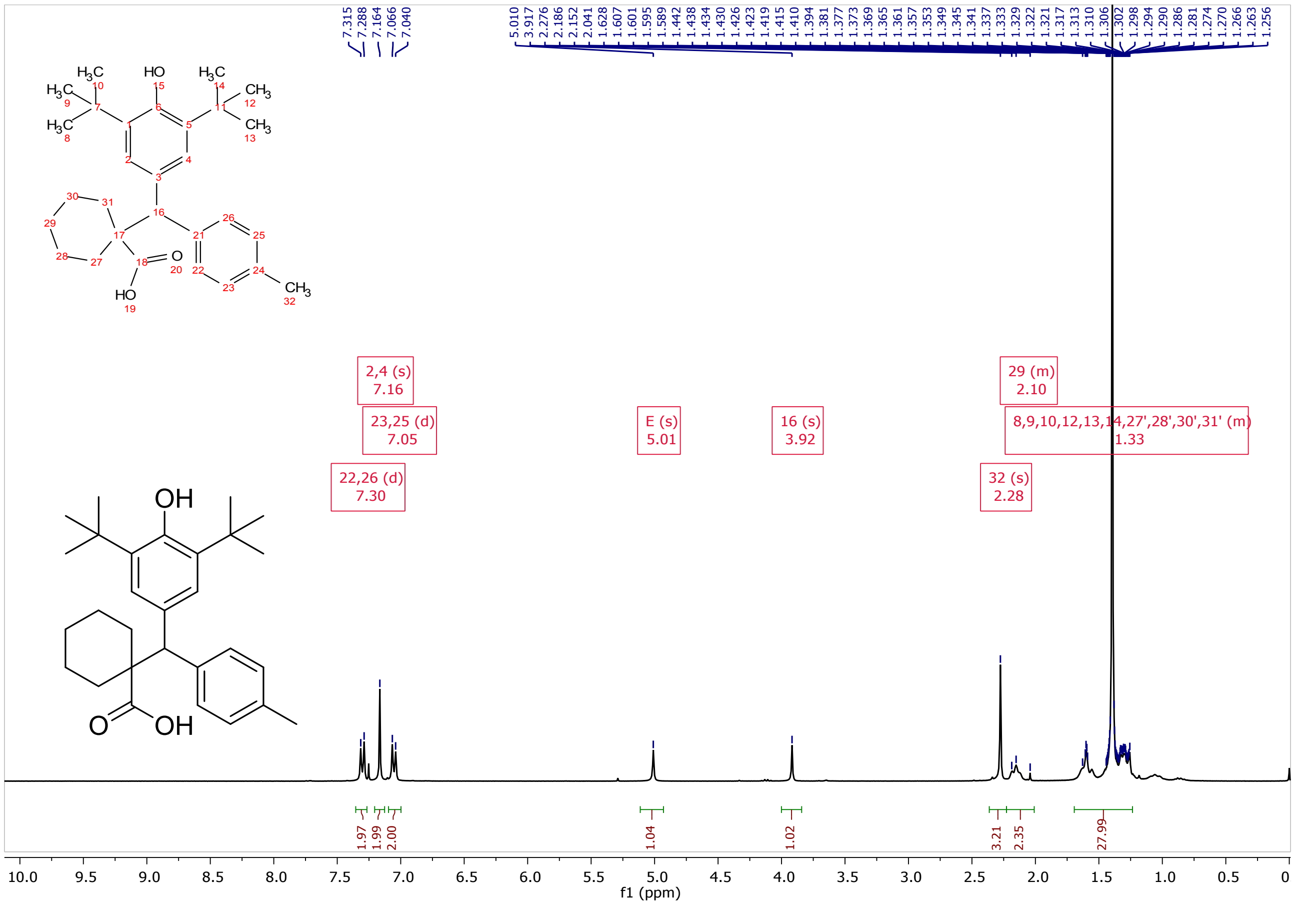

Figure S51. ^{1}H-NMR, 300 MHz, $CDCl_3$ for 3bd

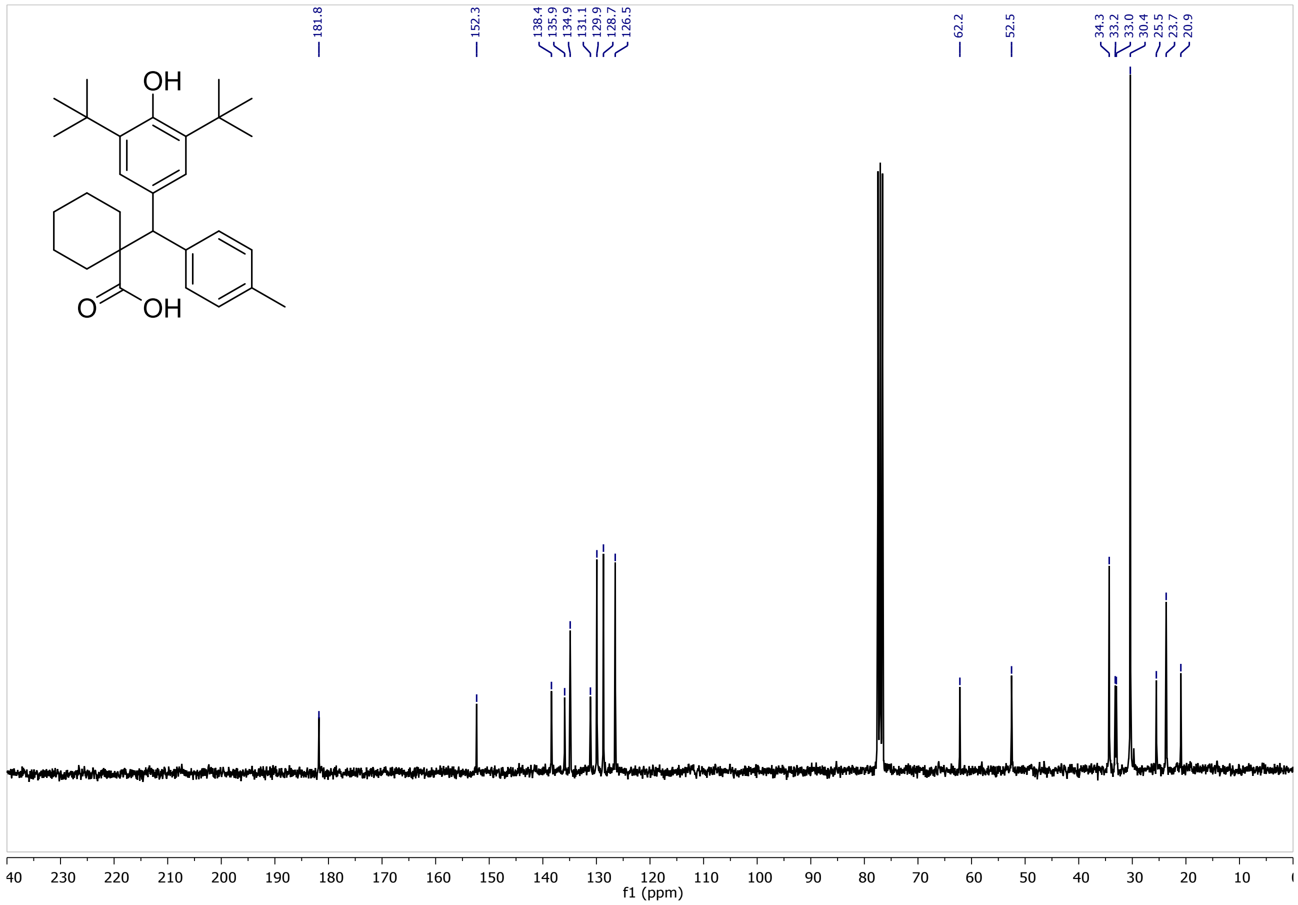

Figure S52. $^{13}C\{^{1}H\}$-NMR, 75 MHz, $CDCl_3$ for 3bd

Data:U-915 EN-CHexTol
Sample Name:M. en C. Saulo C. Rosales
Description:
Ionization Mode:ESI+
History:Determine m/z[Peak Detect[Centroid,30,Area];Correct Base[5.0%]];Correct Base[5.0%];Average(MS[1] 0..0)

Acquired:8/6/2024 12:15:51 PM
Operator:AccuTOF
Mass Calibration data:CAL_PEG_600_ALUMNOS_2024
Created:8/7/2024 12:27:03 PM
Created by:AccuTOF

Charge number:1
Tolerance:3.00(ppm), 5.00 .. 15.00(mmu)
Unsaturation Number:-1.5 .. 100.0 (Fraction:Both)
Element:^{12}C:0 .. 30, ^{1}H:0 .. 45, ^{14}N:0 .. 1, ^{16}O:0 .. 3

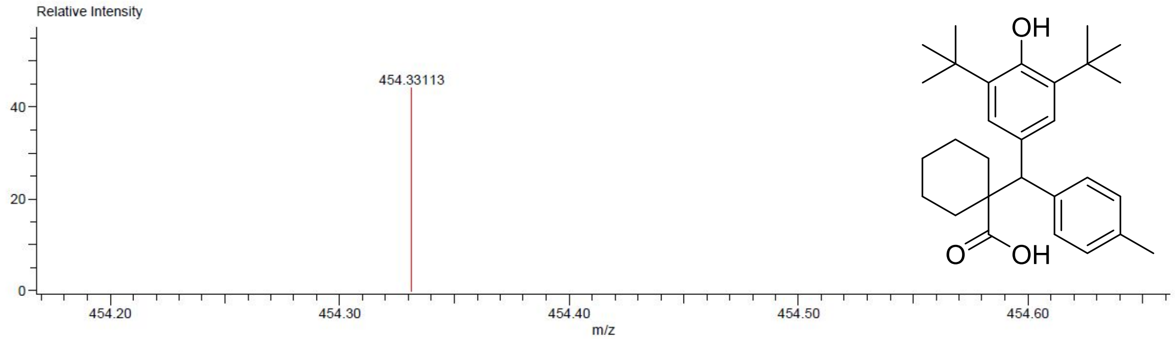

Mass	Intensity	Calc. Mass	Mass Difference (mmu)	Mass Difference (ppm)	Possible Formula	Unsaturation Number
454.33113	5040.81	454.33212	-0.99	-2.17	$^{12}C_{29}{}^{1}H_{44}{}^{14}N_{1}{}^{16}O_{3}$	8.5

Figure S53. HRMS, DART-ESI^{+} (TOF) for 3bd

3-(3,5-di-*tert*-butyl-4-hydroxyphenyl)-2,2-dimethyl-3-(2-methylphenyl)propanoic acid (3qa): 111 mg, 56% Yield (White solid), ^{1}H NMR (300 MHz, Chloroform-*d*) δ 7.53 (d, *J* = 7.6 Hz, 1H), 7.18 – 7.05 (m, 3H), 6.99 (s, 2H), 5.02 (s, 1H), 4.58 (s, 1H), 2.28 (s, 3H), 1.35 (s, 18H), 1.31 (s, 3H), 1.24 (s, 3H); $^{13}C\{^{1}H\}$ NMR (75 MHz, $CDCl_3$) δ 184.4, 152.1, 140.9, 137.1, 134.8, 130.7, 130.4, 127.4, 126.9, 126.0, 125.5, 52.8, 46.8, 34.2, 30.3, 25.5, 23.8, 20.8; HRMS (DART-ESI$^+$) m/z: $[M+NH_4]^+$ Calcd for $C_{26}H_{40}NO_3$, 414.3003, Found, 414.3015.

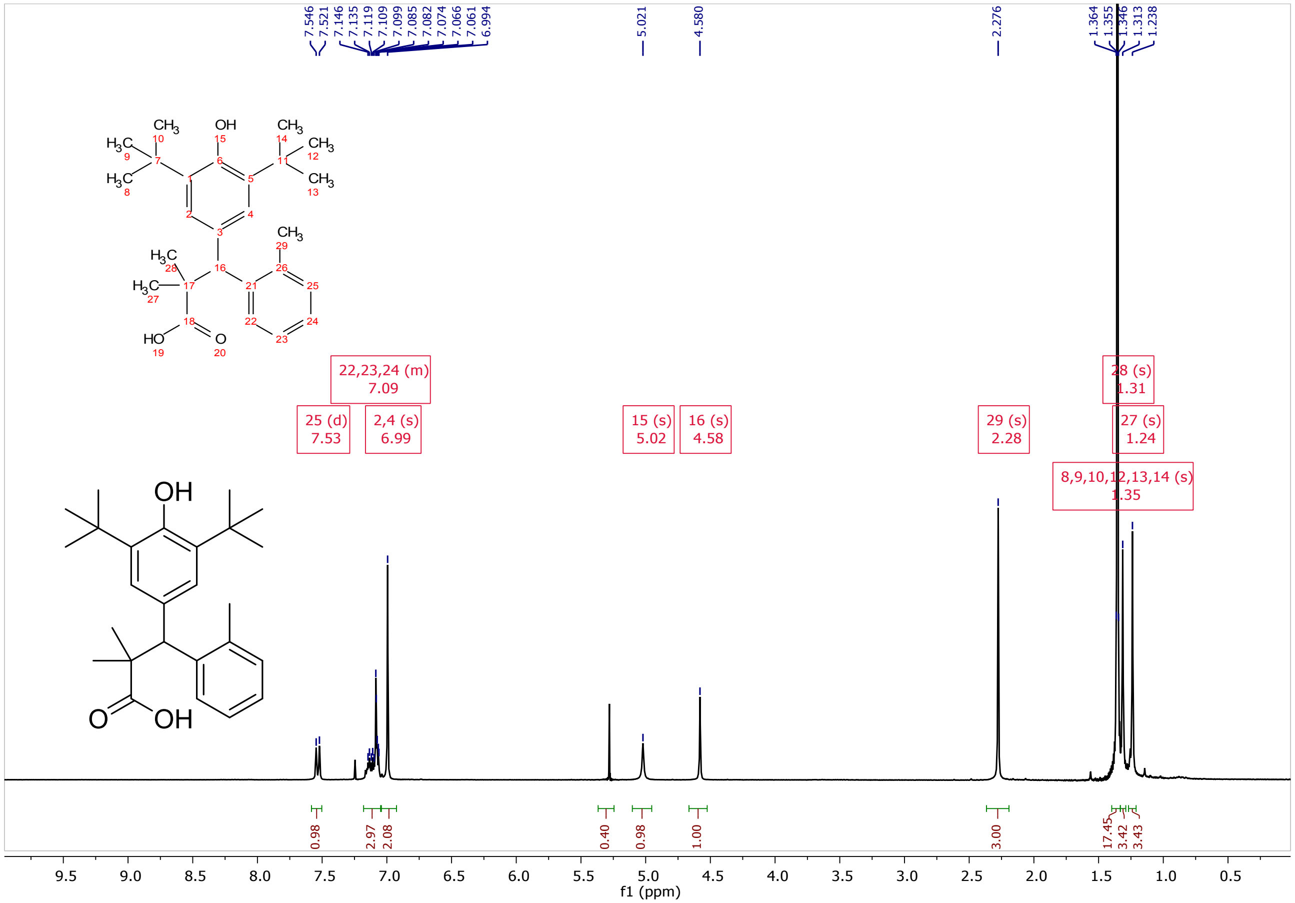

Figure S54. ^{1}H-NMR, 300 MHz, $CDCl_3$ for 3qa

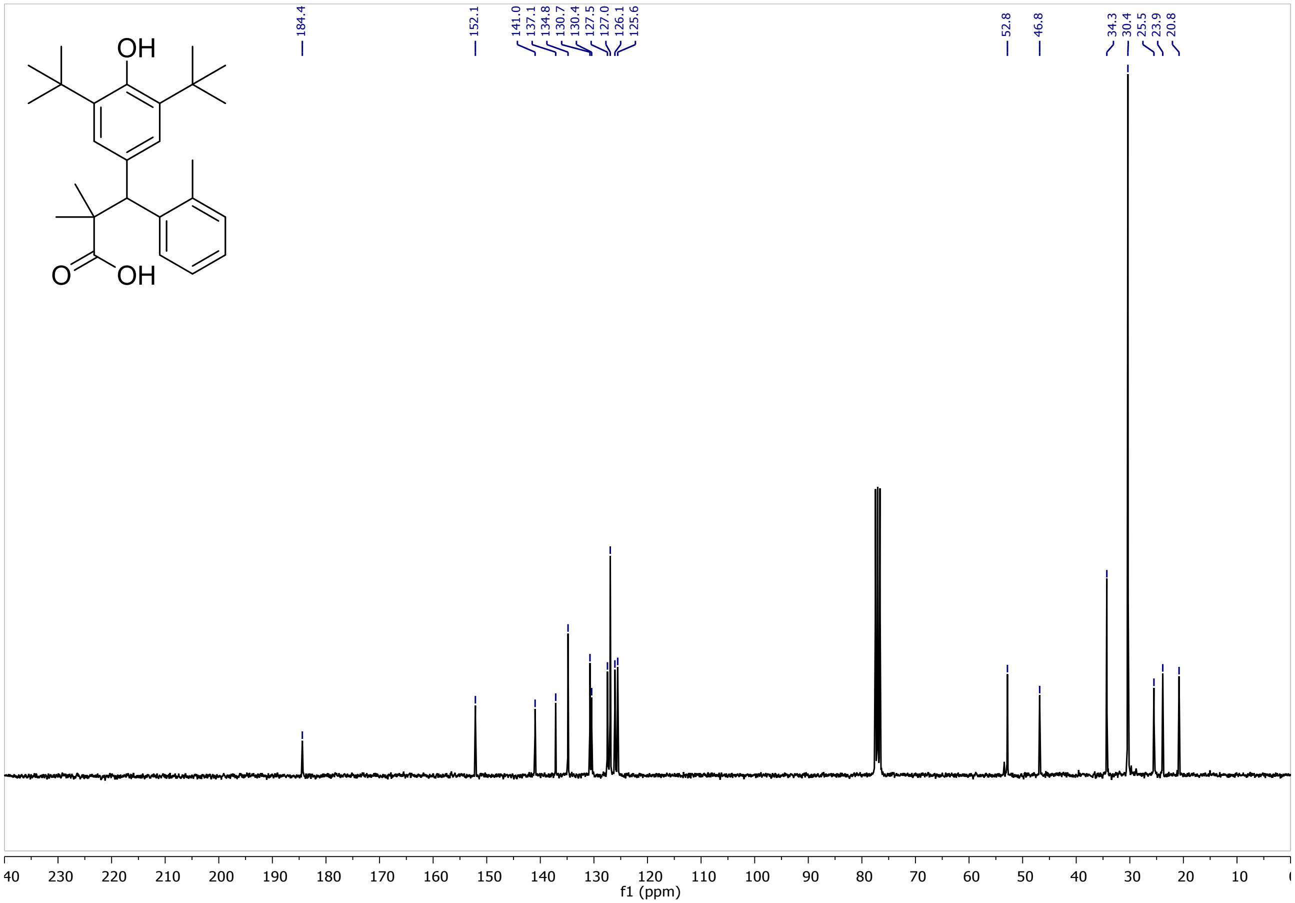

Figure S55. $^{13}C\{^1H\}$**-NMR, 75 MHz,** $CDCl_3$ **for 3qa**

Data:U-940 EN-2Tol
Sample Name:M. en C. Saulo C. Rosales
Description:
Ionization Mode:ESI+
History:Determine m/z[Peak Detect[Centroid,30,Area];Correct Base[5.0%]];Correct Base[5.0%];Average(MS[1] 0..0)

Acquired:8/8/2024 12:11:57 PM
Operator:AccuTOF
Mass Calibration data:CAL_PEG_600_ALUMNOS_2024
Created:8/9/2024 10:30:30 AM
Created by:AccuTOF

Charge number:1
Tolerance:5.00(mmu)
Unsaturation Number:-1.5 .. 30.0 (Fraction:Both)
Element:^{12}C:0 .. 26, ^{1}H:0 .. 53, ^{14}N:0 .. 2, ^{16}O:0 .. 3

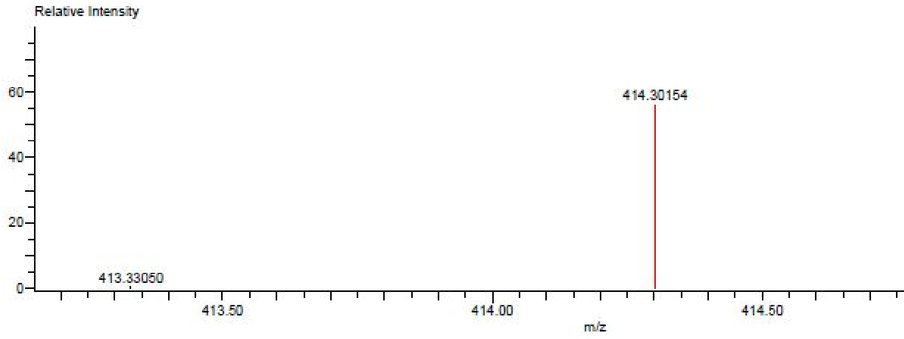

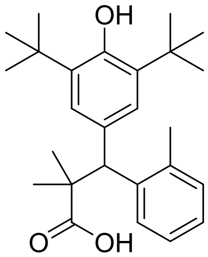

Mass	Intensity	Calc. Mass	Mass Difference (mmu)	Mass Difference (ppm)	Possible Formula	Unsaturation Number
414.30154	3058.25	414.30082	0.72	1.74	$^{12}C_{26}{}^{1}H_{40}{}^{14}N_{1}{}^{16}O_{3}$	7.5

Figure S56. HRMS, DART-ESI^{+} (TOF) for 3qa

3-(2-chlorophenyl)-3-(3,5-di-*tert*-butyl-4-hydroxyphenyl)-2,2-dimethylpropanoic acid (3ra): 102 mg, 49% Yield (White solid), ^{1}H NMR (300 MHz, Chloroform-*d*) δ 7.58 (dd, *J* = 7.8, 1.8 Hz, 1H), 7.34 (dd, *J* = 7.8, 1.6 Hz, 1H), 7.24 – 7.07 (m, 2H), 7.03 (s, 2H), 5.04 (d, *J* = 6.4 Hz, 2H), 1.37 (d, *J* = 1.5 Hz, 16H), 1.33 (s, 3H), 1.26 (s, 3H); $^{13}C\{^{1}H\}$ NMR (75 MHz, $CDCl_3$) δ 183.9, 152.2, 140.0, 135.4, 134.9, 130.2, 129.8, 127.4, 126.6, 126.2, 52.9, 46.7, 34.3, 30.3, 24.8, 24.3, 18.7; HRMS (DART-ESI^{+}) m/z: $[M+NH_4]^{+}$ Calcd for $C_{25}H_{37Cl}NO_3$, 434.2456, Found, 434.2646.

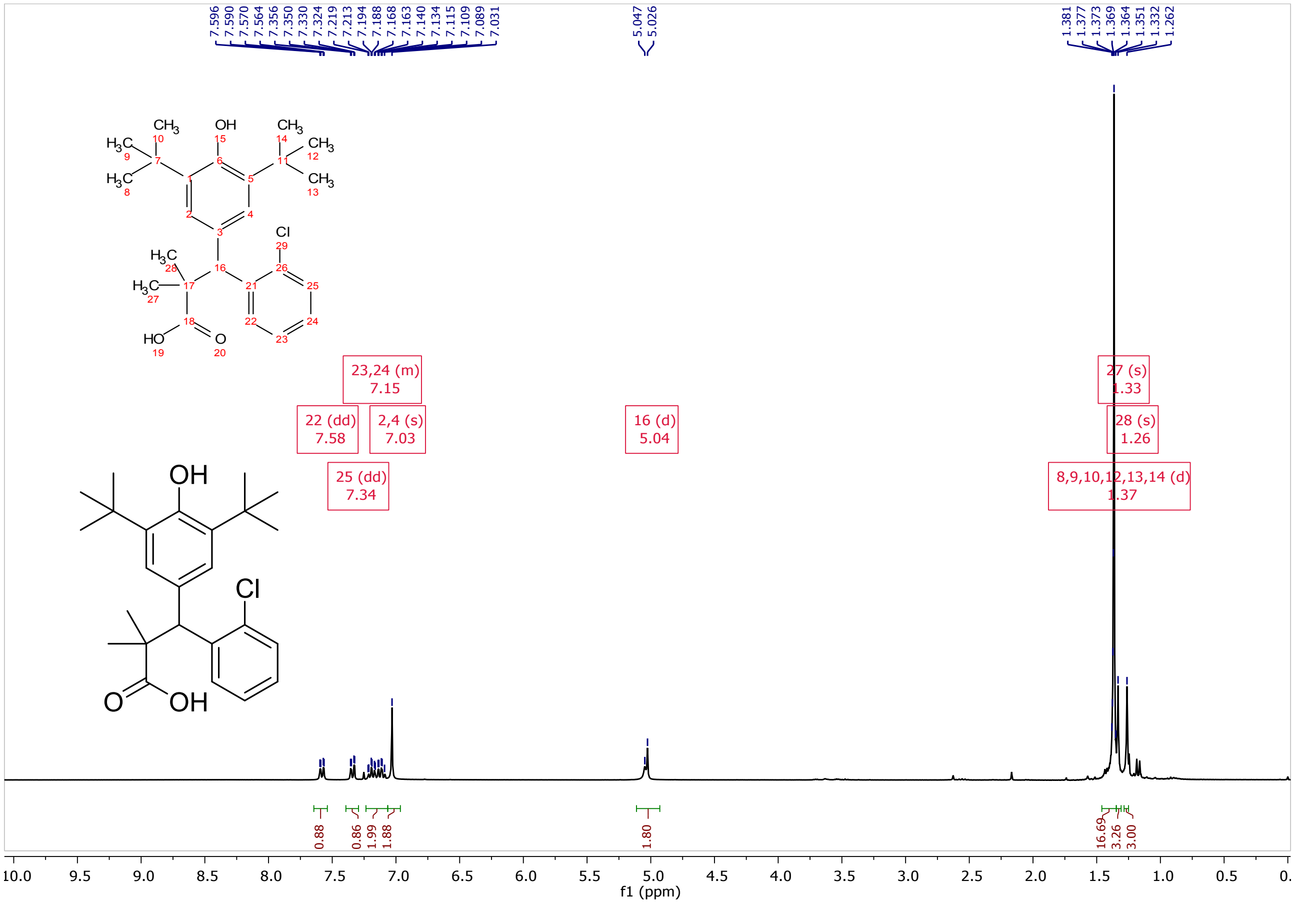

Figure S57. ^{1}H-NMR, 300 MHz, $CDCl_3$ for 3ra

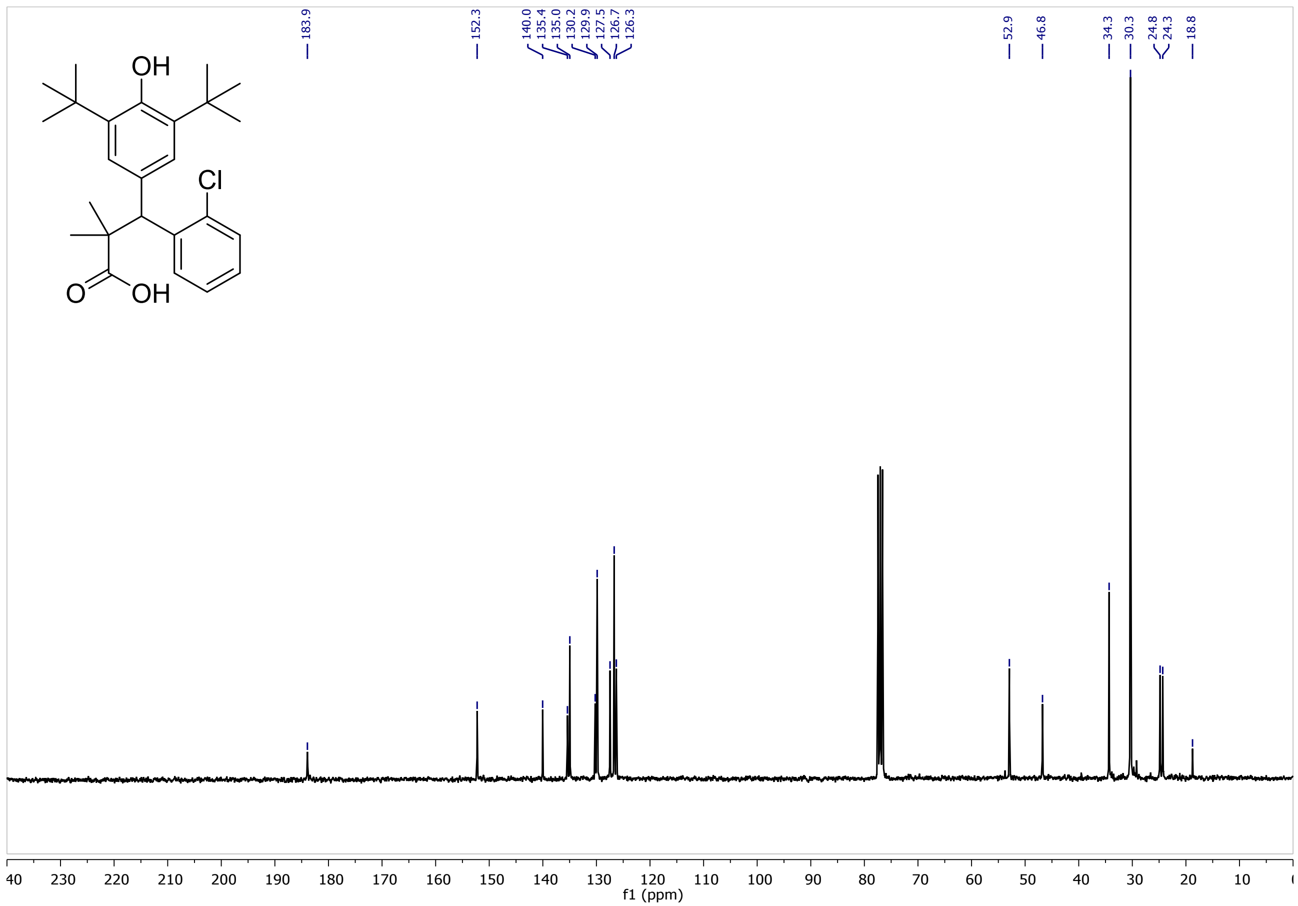

Figure S58. $^{13}C\{^{1}H\}$-NMR, 75 MHz, $CDCl_3$ for 3ra

1-(3,5-di-*tert*-butyl-4-hydroxyphenyl)-4,4-dimethyl-7-((trifluoromethyl)sulfonyl)-1,4,4a,7-tetrahydro-3*H*-pyrano[3,4-*c*]pyridin-3-one (4sa): 142 mg, 55% Yield (White solid), ^1H NMR (300 MHz, Chloroform-*d*) δ 7.08 (s, 2H), 6.67 (ddt, *J* = 8.4, 1.3, 0.6 Hz, 1H), 6.04 (tt, *J* = 1.8, 0.8 Hz, 1H), 5.78 – 5.64 (m, 1H), 5.39 (s, 1H), 5.17 (dd, *J* = 8.4, 4.1 Hz, 1H), 3.46 (dq, *J* = 3.1, 1.0 Hz, 1H), 1.43 (d, *J* = 6.6 Hz, 22H), 1.32 (s, 3H); $^{13}C\{^1H\}$ NMR (75 MHz, $CDCl_3$) δ 175.3, 154.8, 136.4, 125.2, 124.7, 123.6, 121.1, 118.9, 106.9, 83.9, 44.1, 40.0, 34.3, 30.0, 22.9, 22.2; HRMS (DART-ESI$^+$) m/z: $[M+H]^+$ Calcd for $C_{25}H_{33}F_3NO_5S$, 516.1894, Found, 516.2020.

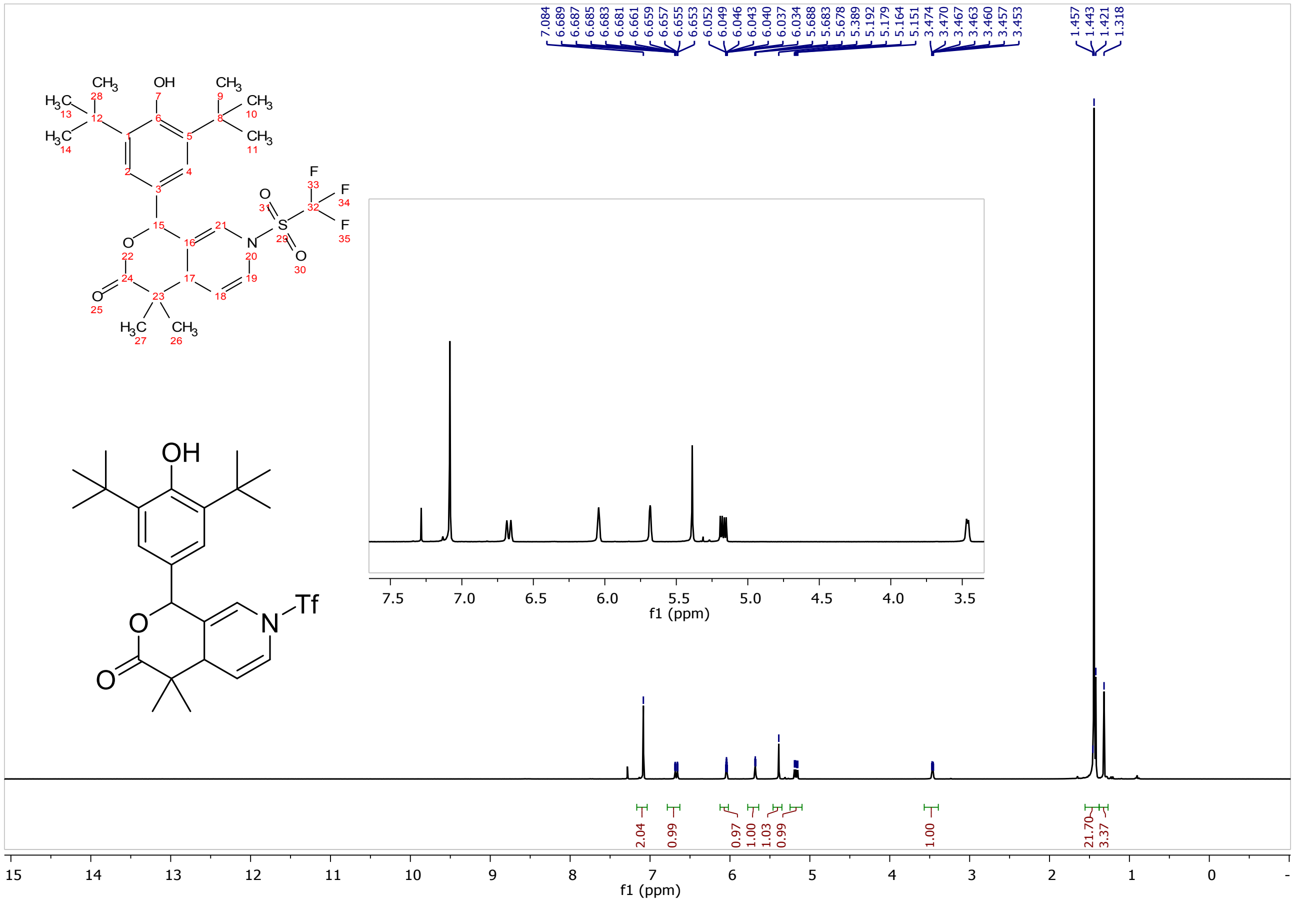

Figure S59. ^1H-NMR, 300 MHz, $CDCl_3$ for 4sa

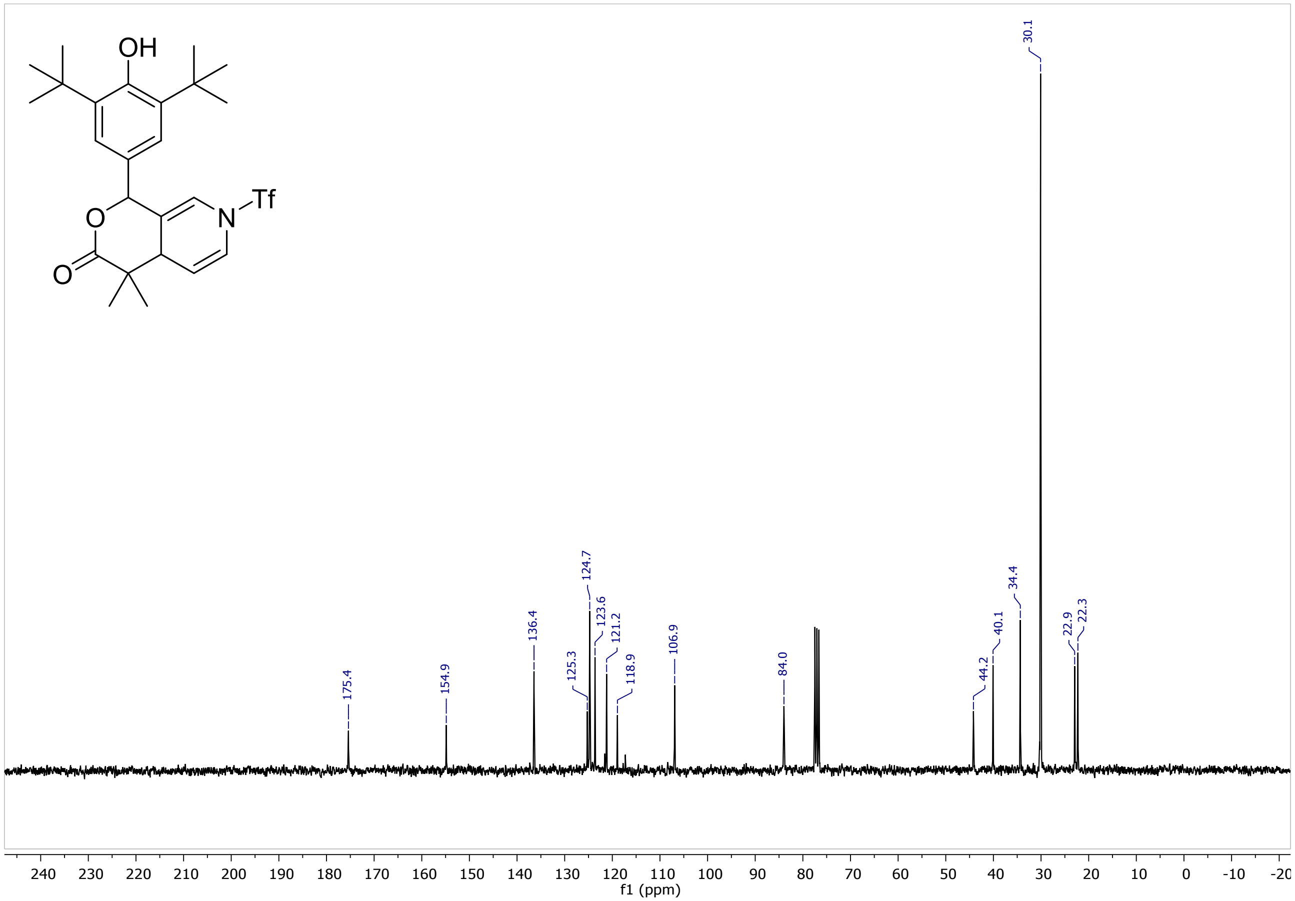

Figure S60. $^{13}C\{^{1}H\}$**-NMR, 75 MHz,** $CDCl_3$ **for 4sa**

INSTITUTO DE QUIMICA, UNAM
LABORATORIO DE ESPECTROMETRIA DE MASAS

Data:836 LJBP-40
Sample Name:Dr. Alvarez Cecilio/ Operador Javier Perez
Description:
Ionization Mode:ESI+
History:Determine m/z[Peak Detect[Centroid,30,Area];Correct Base[];Smooth[5]];Correct Base[5.0%];Average(MS[...

Acquired:3/16/2023 5:49:39 PM
Operator:AccuTOF
Mass Calibration data:Cal_PEG_600
Created:3/31/2023 1:44:37 PM
Created by:AccuTOF

Charge number:1 Tolerance:3.00(mmu) Unsaturation Number:-1.0 .. 50.0 (Fraction:.5)
Element:^{12}C:0 .. 25, ^{1}H:0 .. 40, ^{19}F:1 .. 3, ^{14}N:0 .. 3, ^{16}O:0 .. 5, ^{32}S:0 .. 1

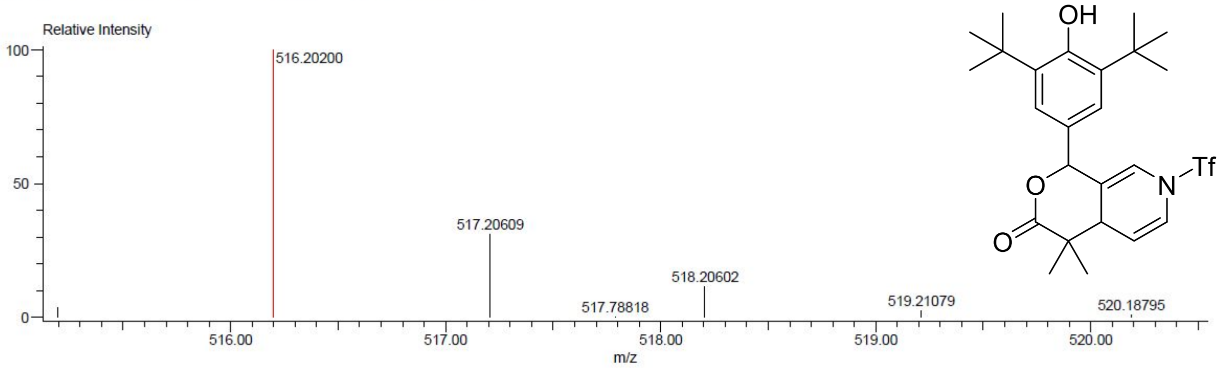

Mass	Intensity	Calc. Mass	Mass Difference (mmu)	Mass Difference (ppm)	Possible Formula	Unsaturation Number
516.20200	337947.42	516.20315	-1.15	-2.23	$^{12}C_{25}{}^{1}H_{33}{}^{19}F_{3}{}^{14}N_{1}{}^{16}O_{5}{}^{32}S_{1}$	9.5

Figure S61. HRMS, DART-ESI^{+} (TOF) for 4sa

1-(3,5-di-*tert*-butyl-4-hydroxyphenyl)-4,4-dimethyl-7-((trifluoromethyl)sulfonyl)-1,4,4a,7-tetrahydro-3*H*-pyrano[3,4-*c*]pyridin-3-one (4sb): 120 mg, 45% Yield (White solid), ^{1}H NMR (300 MHz, Chloroform-*d*) δ 7.23 (s, 1H), 7.17 (d, *J* = 0.8 Hz, 1H), 6.84 – 6.58 (m, 1H), 5.56 – 5.40 (m, 1H), 5.14 – 5.01 (m, 1H), 3.21 – 2.98 (m, 1H), 2.56 – 2.12 (m, 5H), 2.09 – 1.91 (m, 1H), 1.60 – 1.41 (m, 18H); $^{13}C\{^{1}H\}$ NMR (75 MHz, $CDCl_3$) δ 154.6, 136.5, 136.0, 132.2, 126.2, 125.8, 124.5, 124.1, 106.1, 70.7, 48.7, 44.3, 34.3, 32.1, 30.2, 30.1, 23.9, 16.1**;** HRMS (DART-ESI$^+$) m/z: $[M+H]^+$ Calcd for $C_{26}H_{33}F_3NO_5S$, 528.1889, Found, 528.2040.

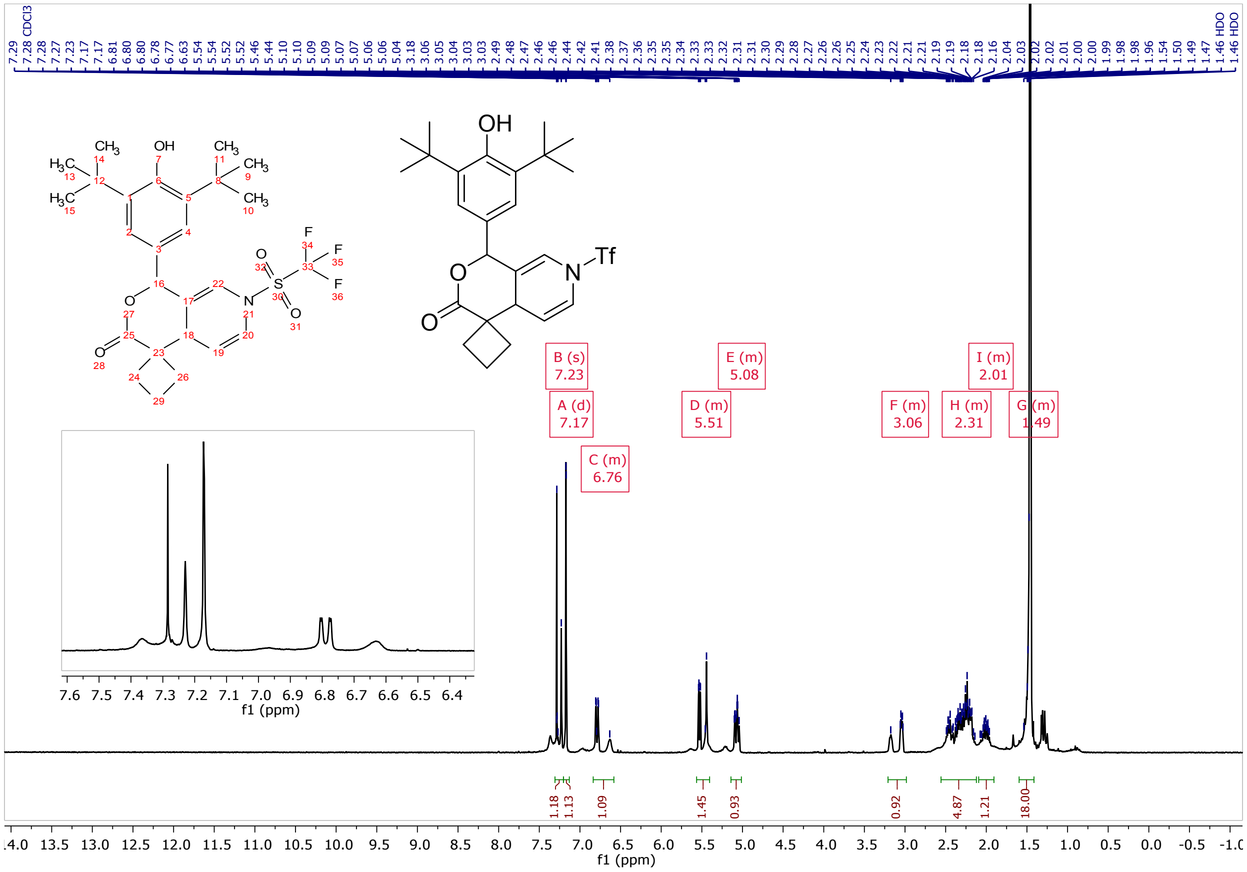

Figure S62. ^{1}H-NMR, 300 MHz, $CDCl_3$ for 4sb

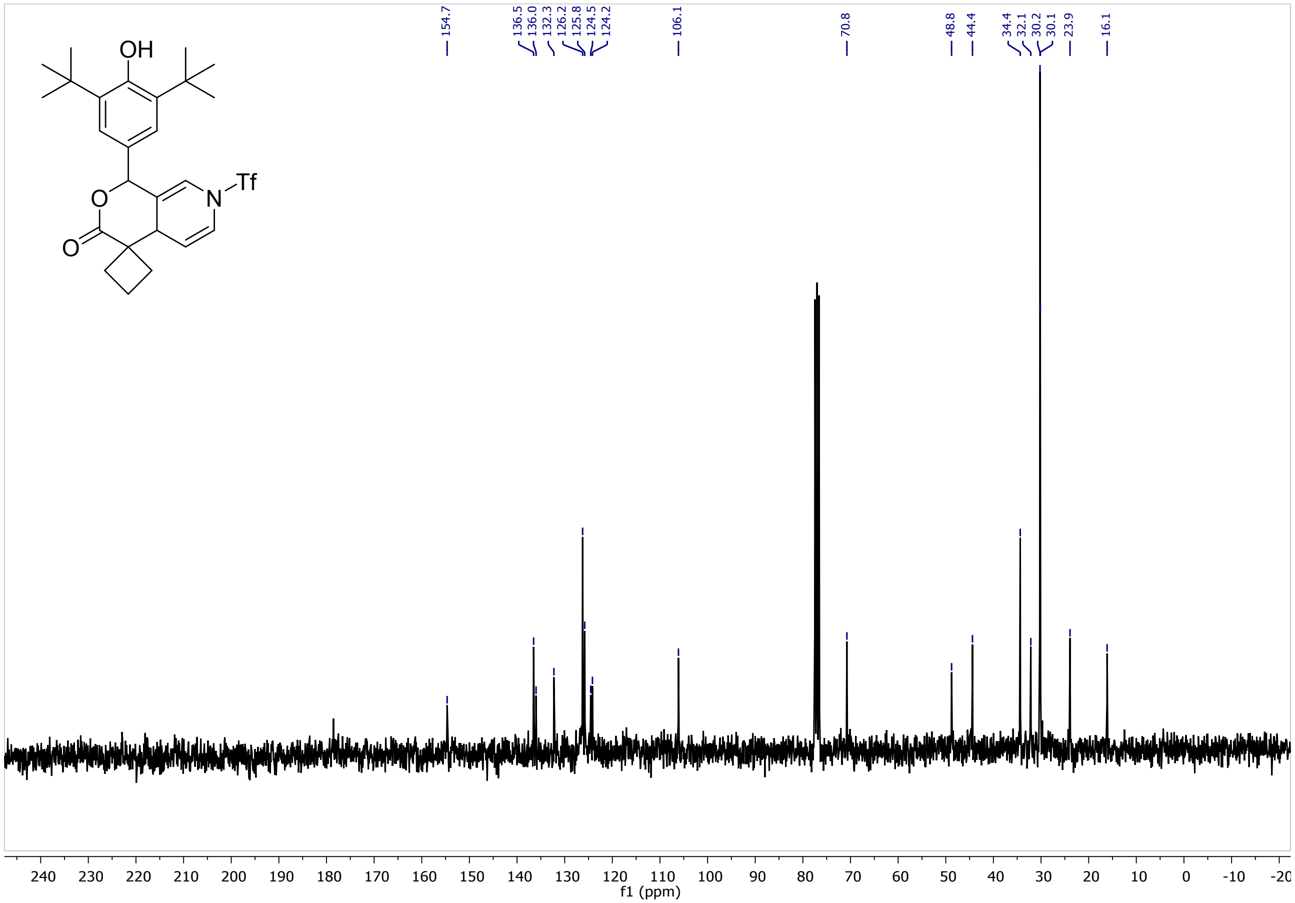

Figure S63. $^{13}C\{^1H\}$**-NMR, 75 MHz,** $CDCl_3$ **for 4sb**

INSTITUTO DE QUIMICA, UNAM
LABORATORIO DE ESPECTROMETRIA DE MASAS

Data:838 LJBP-57
Sample Name:Dr. Alvarez Cecilio/ Operador Javier Perez
Description:
Ionization Mode:ESI+
History:Determine m/z[Peak Detect[Centroid,30,Area];Correct Base[];Smooth[5]];Correct Base[5.0%];Average(MS[...

Acquired:3/16/2023 5:55:49 PM
Operator:AccuTOF
Mass Calibration data:Cal_PEG_600
Created:4/3/2023 9:13:38 AM
Created by:AccuTOF

Charge number:1 Tolerance:15.00(mmu) Unsaturation Number:-1.0 .. 50.0 (Fraction:.5)
Element:^{12}C:0 .. 27, ^{1}H:0 .. 40, ^{19}F:1 .. 3, ^{14}N:0 .. 2, ^{16}O:0 .. 6, ^{32}S:0 .. 1

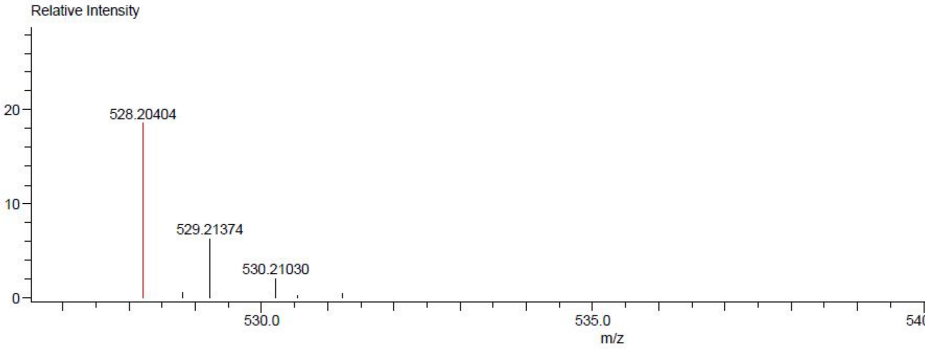

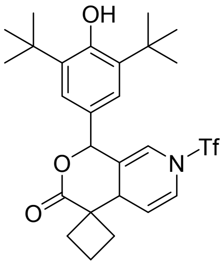

Mass	Intensity	Calc. Mass	Mass Difference (mmu)	Mass Difference (ppm)	Possible Formula	Unsaturation Number
528.20404	16598.51	528.20315	0.89	1.68	$^{12}C_{26}{}^{1}H_{33}{}^{19}F_{3}{}^{14}N_{1}{}^{16}O_{5}{}^{32}S_{1}$	10.5

Figure S64. HRMS, DART-ESI^{+} (TOF) for 4sc

1-(3,5-di-*tert*-butyl-4-hydroxyphenyl)-4-spiro[4.5]-7-((trifluoromethyl)sulfonyl)-1,4,4a,7-tetrahydro-3*H*-pyrano[3,4-*c*]pyridin-3-one (4sc): 122 mg, 45% Yield (White solid), ^{1}H NMR (300 MHz, Chloroform-*d*) δ 7.24 – 7.09 (m, 1H), 7.03 (d, *J* = 11.9 Hz, 1H), 6.84 – 6.73 (m, 1H), 5.68 (d, *J* = 3.0 Hz, 1H), 5.48 – 5.30 (m, 2H), 3.62 – 3.28 (m, 1H), 3.02 – 2.74 (m, 0H), 2.56 – 1.80 (m, 1H), 1.58 – 1.36 (m, 18H); ^{13}C{^{1}H} NMR (75 MHz, $CDCl_3$) δ 174.6, 174.4, 154.8, 154.2, 136.3, 136.3, 127.7, 125.8, 125.7, 124.8, 124.2, 123.9, 123.1, 122.3, 121.5, 121.1, 119.3, 118.7, 107.3, 107.2, 83.5, 80.8, 48.3, 47.9, 37.3, 34.8, 34.4, 34.3, 30.2, 30.1, 30.0, 27.6, 27.3, 24.1, 23.9, 15.3, 15.2; HRMS (DART-ESI$^+$) m/z: $[M+H]^+$ Calcd for $C_{27}H_{34}F_3NO_5S$, 542.2040, Found, 542.2168.

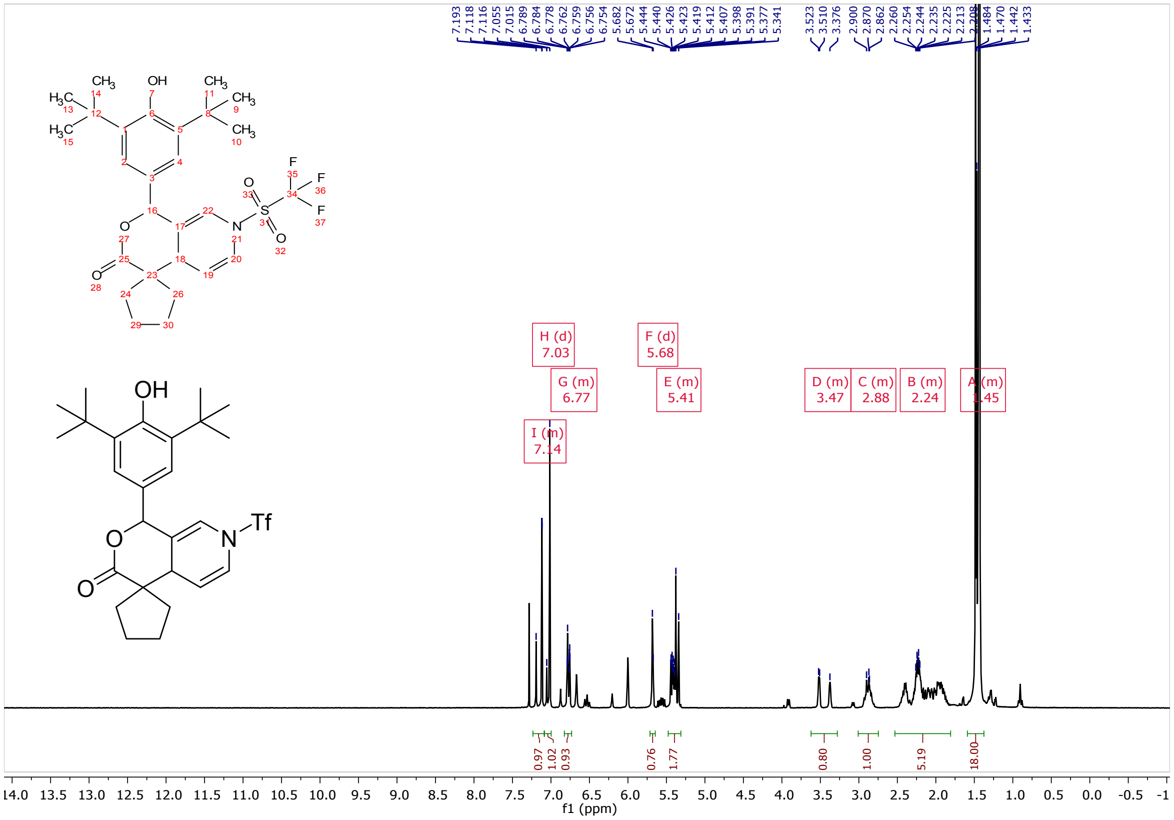

Figure S65. **^{1}H-NMR, 300 MHz, $CDCl_3$ for 4sc**

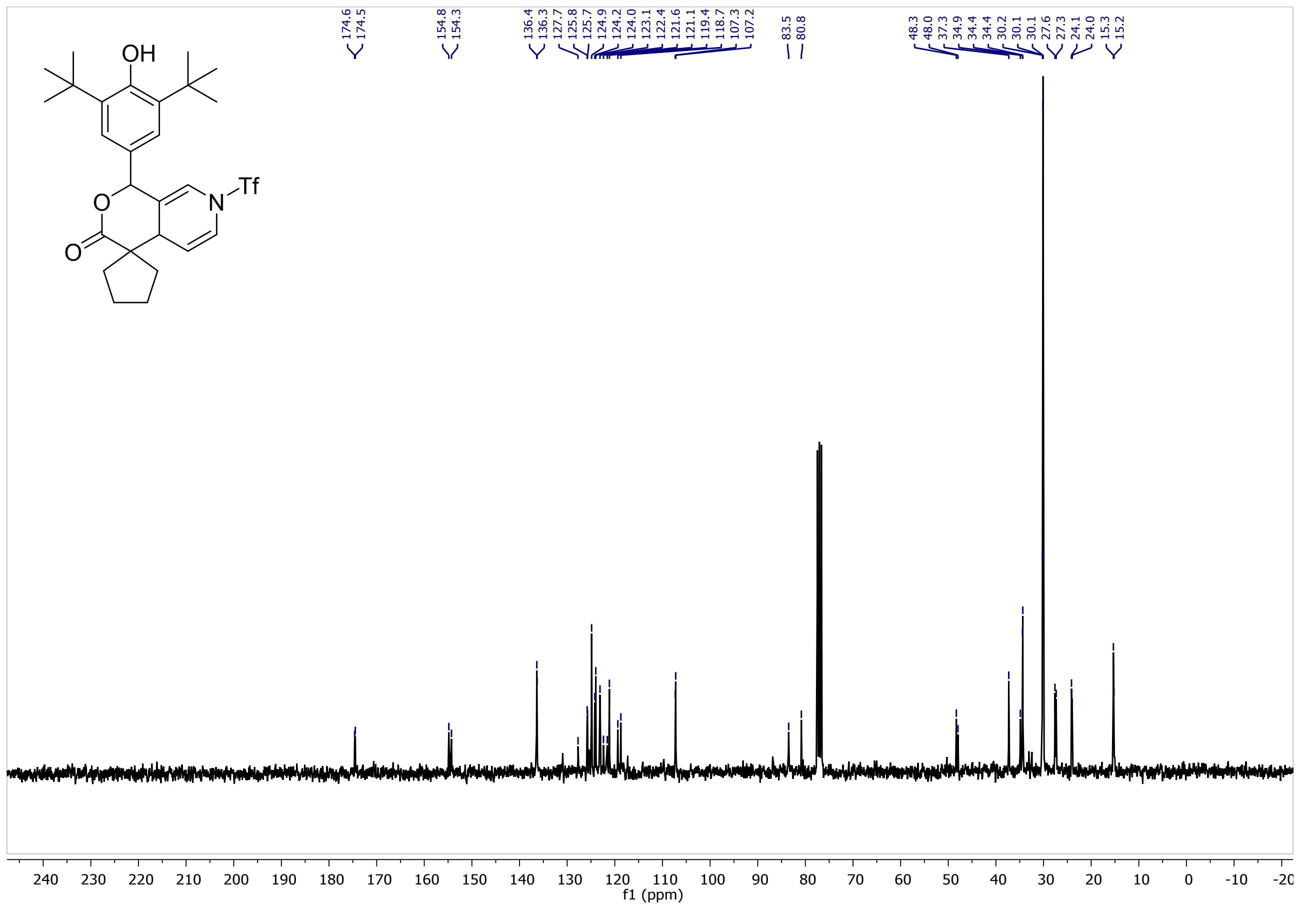

Figure S66. $^{13}C\{^{1}H\}$**-NMR, 75 MHz,** $CDCl_3$ **for 4sc**

INSTITUTO DE QUIMICA, UNAM
LABORATORIO DE ESPECTROMETRIA DE MASAS

Data:837 LJBP-30b
Sample Name:Dr. Alvarez Cecilio/ Operador Javier Perez
Description:
Ionization Mode:ESI+
History:Determine m/z[Peak Detect[Centroid,30,Area];Correct Base[];Smooth[5]];Correct Base[5.0%];Average(MS[...

Acquired:3/16/2023 5:52:24 PM
Operator:AccuTOF
Mass Calibration data:Cal_PEG_600
Created:3/31/2023 1:49:39 PM
Created by:AccuTOF

Charge number:1
Tolerance:3.00(mmu)
Unsaturation Number:-1.0 .. 50.0 (Fraction:.5)
Element:^{12}C:0 .. 27, ^{1}H:0 .. 40, ^{19}F:1 .. 3, ^{14}N:0 .. 3, ^{16}O:0 .. 5, ^{32}S:0 .. 1

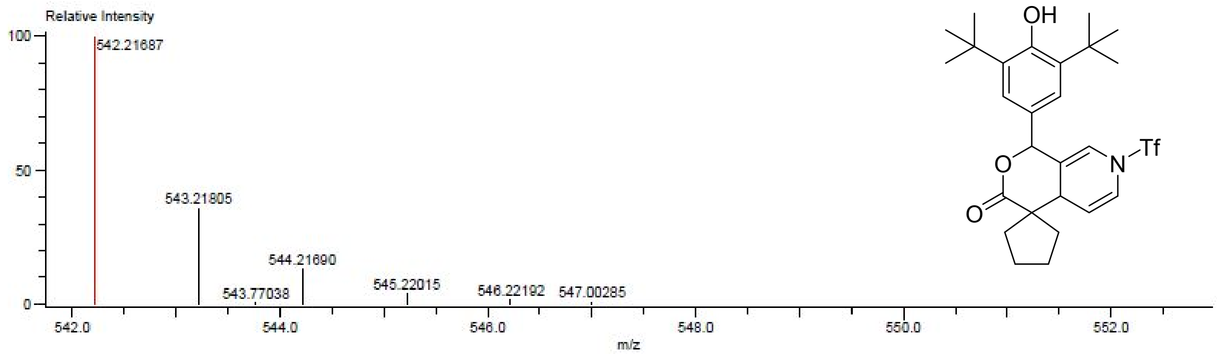

Mass	Intensity	Calc. Mass	Mass Difference (mmu)	Mass Difference (ppm)	Possible Formula	Unsaturation Number
542.21687	920967.45	542.21880	-1.93	-3.56	$^{12}C_{27}{}^{1}H_{35}{}^{19}F_{3}{}^{14}N_{1}{}^{16}O_{5}{}^{32}S_{1}$	10.5

Figure S67. HRMS, DART-ESI⁺ (TOF) for 4sc

(7*Z*)-7-(3,5-di-*tert*-butyl-4-hydroxybenzylidene)-3,3-dimethyl-6-((trifluoromethyl)sulfonyl)-3a,6,7,7a-tetrahydrofuro[2,3-*c*]pyridin-2(3*H*)-one (4ta): 150 mg, 58% Yield (White solid), ^{1}H NMR (300 MHz, Chloroform-*d*) δ 7.24 (s, 1H), 7.20 (d, *J* = 0.7 Hz, 2H), 6.82 (dd, *J* = 8.4, 1.6 Hz, 1H), 5.72 (dd, *J* = 6.0, 1.2 Hz, 1H), 5.45 (s, 1H), 5.15 (ddd, *J* = 8.4, 3.1, 1.2 Hz, 1H), 1.32 (d, *J* = 11.9 Hz, 8H); $^{13}C\{^{1}H\}$ NMR (75 MHz, $CDCl_3$) δ 179.5, 154.7, 136.56, 133.0, 126.2, 125.5, 124.5, 124.1, 108.4, 71.4, 44.7, 44.5, 34.3, 30.2, 25.0, 20.5; HRMS (DART-ESI$^+$) m/z: $[M+H]^+$ Calcd for $C_{25}H_{32}F_3NO_5S$, 516.2026, Found, 516.2014.

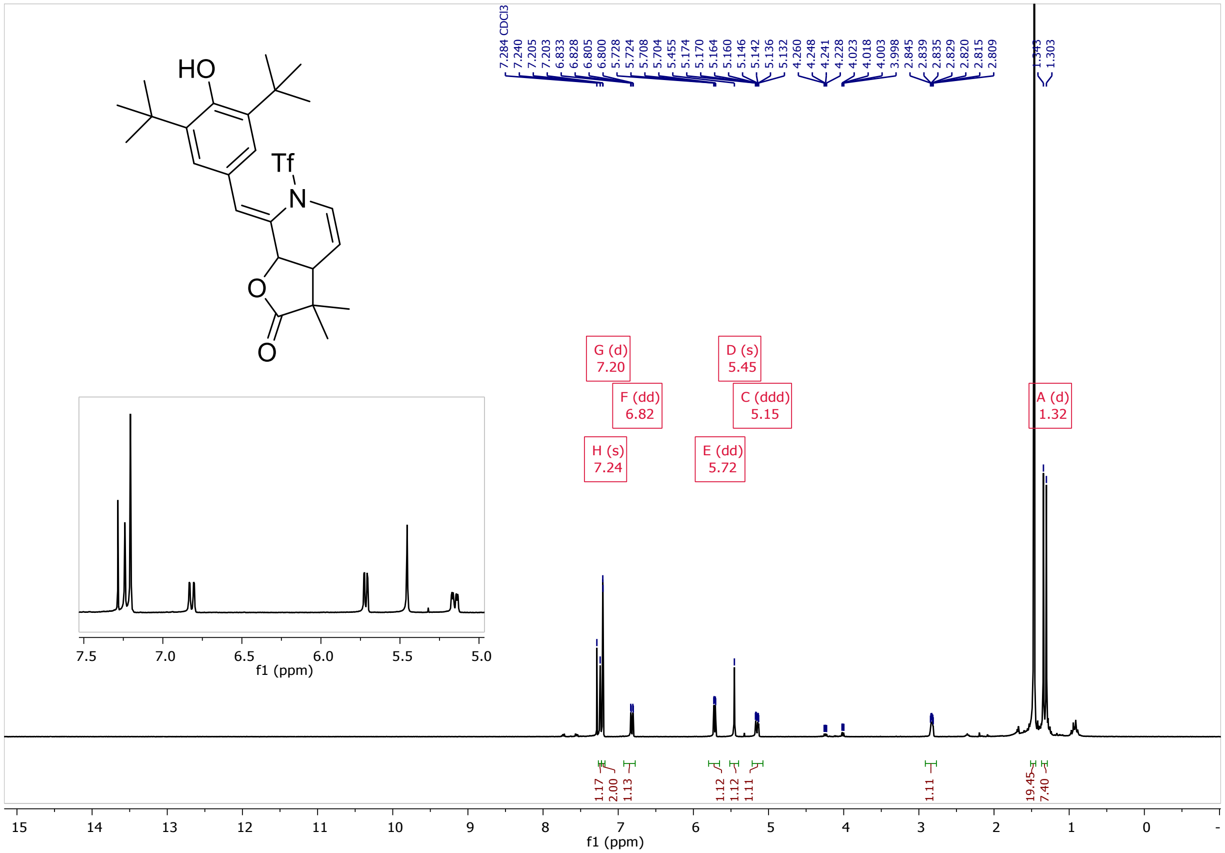

Figure S68. ^{1}H-NMR, 300 MHz, $CDCl_3$ for 4ta

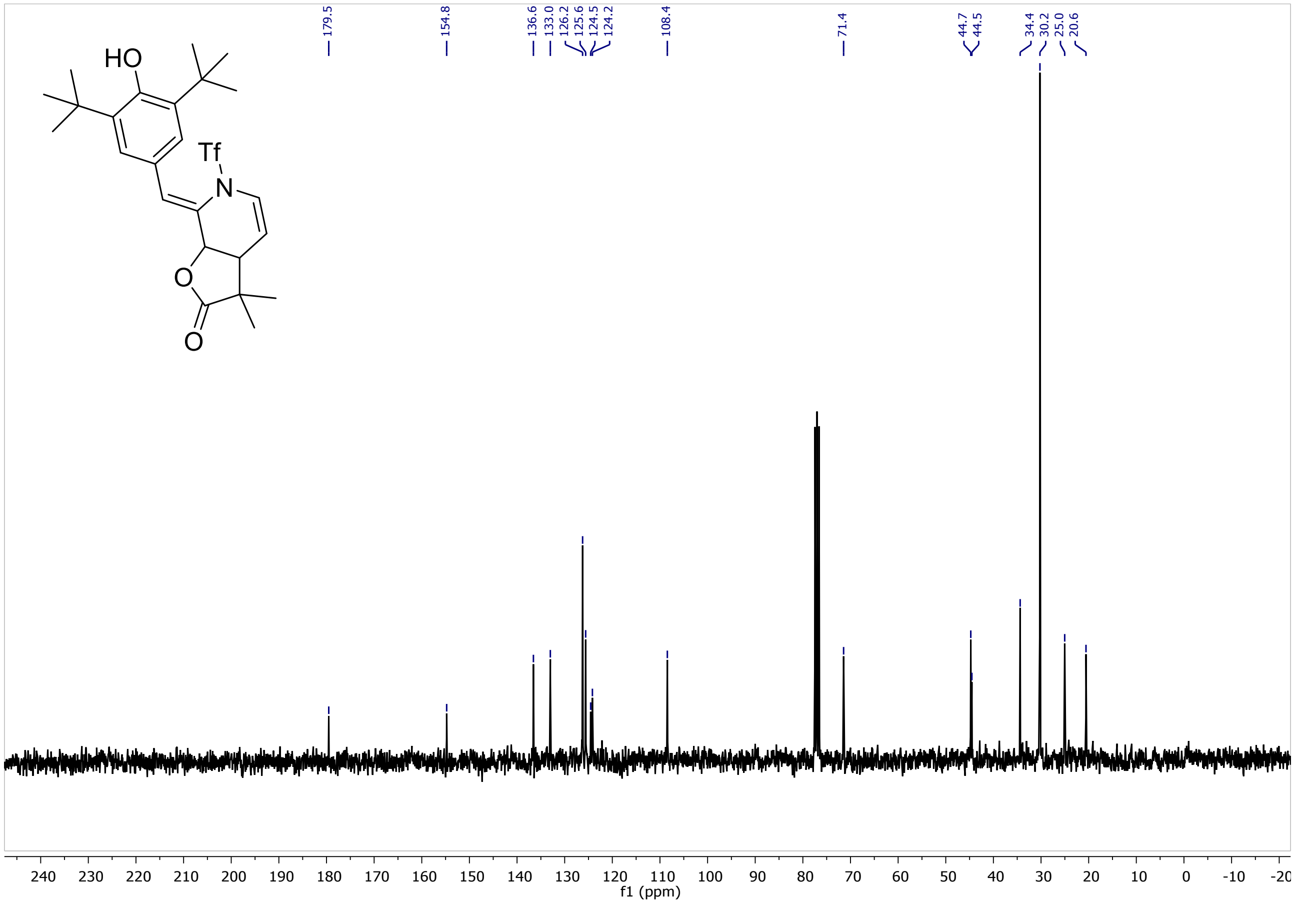

Figure S69. $^{13}C\{^{1}H\}$-NMR, 75 MHz, $CDCl_3$ for 4ta

Sample Name:M. en C. Saulo C. Rosales
Description:
Ionization Mode:ESI+
History:Determine m/z[Peak Detect[Centroid,30,Area];Correct Base[5.0%]];Correct Base[5.0%];Average(MS[1] 1..1)

Operator:AccuTOF
Mass Calibration data:CAL_PEG_600-ENERO_2025_cg
Created:1/22/2025 12:31:53 PM
Created by:AccuTOF

Charge number:1
Tolerance:100.00(ppm), 5.00 .. 15.00(mmu)
Unsaturation Number:-2.5 .. 200.0 (Fraction:Both)
Element:^{12}C:25 .. 25, ^{1}H:0 .. 50, ^{19}F:0 .. 3, ^{14}N:0 .. 1, ^{16}O:0 .. 5, ^{32}S:0 .. 1

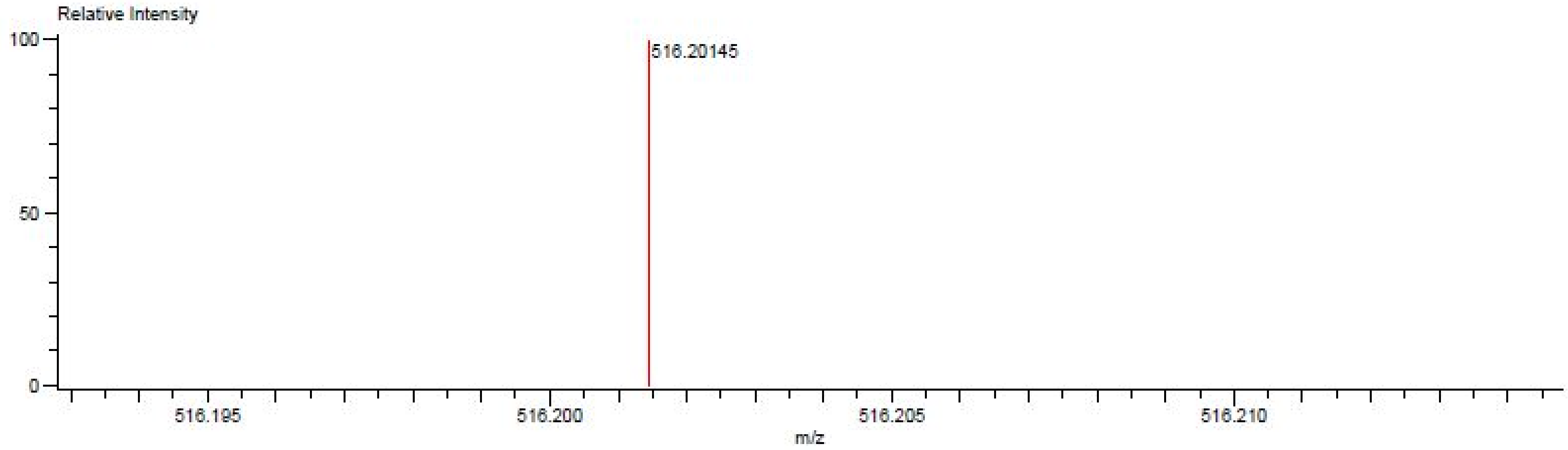

Mass	Intensity	Calc. Mass	Mass Difference (mmu)	Mass Difference (ppm)	Possible Formula	Unsaturation Number
516.20145	4153.37	516.20315	-1.70	-3.29	$^{12}C_{25}{}^{1}H_{33}{}^{19}F_{3}{}^{14}N_{1}{}^{16}O_{5}{}^{32}S_{1}$	8.5

Figure S70. HRMS, DART-ESI^{+} (TOF) for 4ta

(7Z)-7-(3,5-di-tert-butyl-4-hydroxybenzylidene)-3-spiro[4.4]-6-((trifluoromethyl)sulfonyl)-3a,6,7,7a-tetrahydrofuro[2,3-c]pyridin-2(3H)-one (4tc): 110 mg, 40% Yield (White solid), ^{1}H NMR (300 MHz, Chloroform-*d*) δ 7.13 (s, 1H), 7.08 (d, *J* = 0.7 Hz, 2H), 6.69 (dd, *J* = 8.4, 1.6 Hz, 1H), 5.55 (dd, *J* = 5.6, 1.3 Hz, 1H), 5.34 (s, 1H), 5.01 (ddd, *J* = 8.4, 2.7, 1.3 Hz, 1H), 2.69 (dt, *J* = 4.9, 2.2 Hz, 1H), 2.06 – 1.52 (m, 8H), 1.36 (s, 18H); ^{13}C{^{1}H} NMR (75 MHz, CDCl$_3$) δ 179.6, 154.7, 136.5, 132.3, 126.2, 125.5, 124.7, 124.1, 108.0, 71.8, 54.7, 44.3, 37.2, 34.3, 30.7, 30.2, 25.1, 24.9; HRMS (DART-ESI$^+$) m/z: [M+H]$^+$ Calcd for $C_{27}H_{35}F_3NO_5S$, 542.2183, Found, 542.2128.

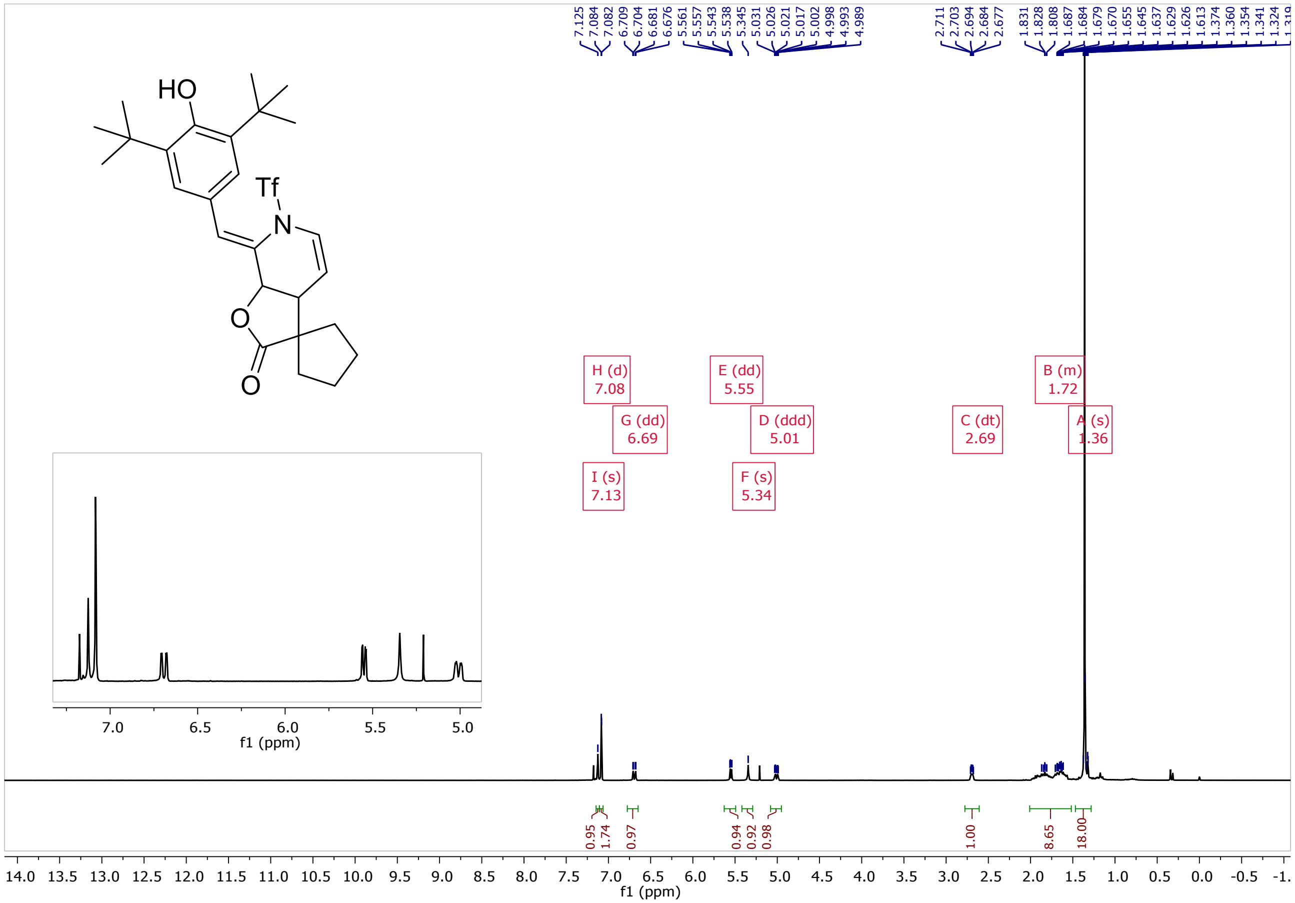

Figure S71. HRMS, DART-ESI$^+$ (TOF) for 4tc

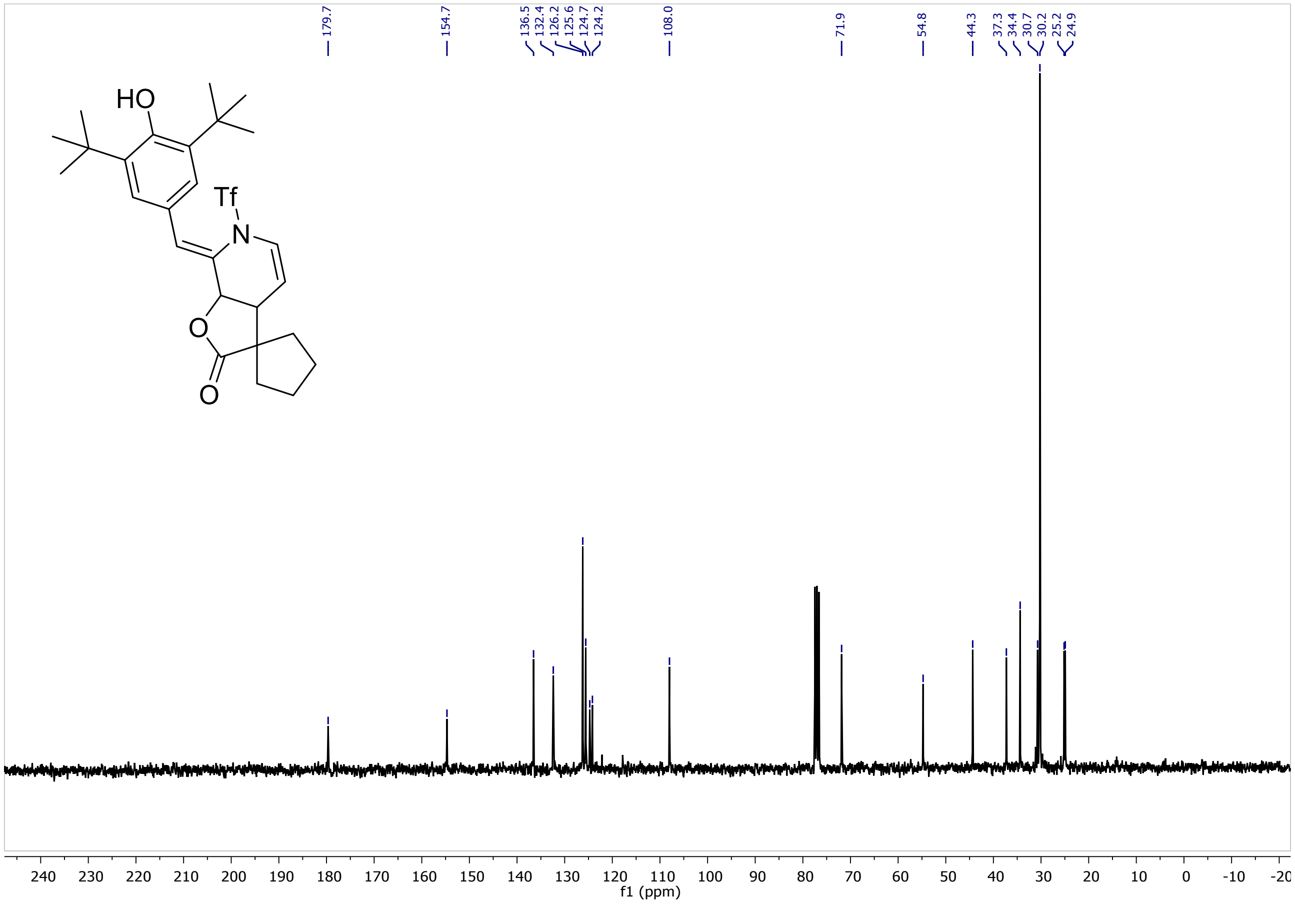

Figure S72. $^{13}C\{^{1}H\}$-NMR, 75 MHz, $CDCl_3$ for 4tc

(7Z)-7-(3,5-di-tert-butyl-4-hydroxybenzylidene)-3-spiro[4.3]-6-((trifluoromethyl)sulfonyl)-3a,6,7,7a-tetrahydrofuro[2,3-c]pyridin-2(3H)-one (4tb): 85 mg, 32% Yield (White solid), ^{1}H NMR (300 MHz, Chloroform-*d*) δ 7.26 – 7.15 (m, 2H), 5.53 (dd, *J* = 5.4, 1.4 Hz, 1H), 5.16 – 4.99 (m, 1H), 3.24 – 2.96 (m, 1H), 2.62 – 1.87 (m, 6H), 1.46 (d, *J* = 1.5 Hz, 18H); ^{13}C{^{1}H} NMR (75 MHz, $CDCl_3$) δ 154.6, 136.5, 136.0, 132.2, 126.2, 125.8, 124.5, 124.1, 106.1, 70.7, 48.7, 44.3, 34.3, 32.1, 30.2, 30.1, 23.9, 16.1; HRMS (DART-ESI^{+}) m/z: $[M+H]^{+}$ Calcd for $C_{26}H_{33}F_{3}NO_{5}S$, 528.2026, Found, 528.2054.

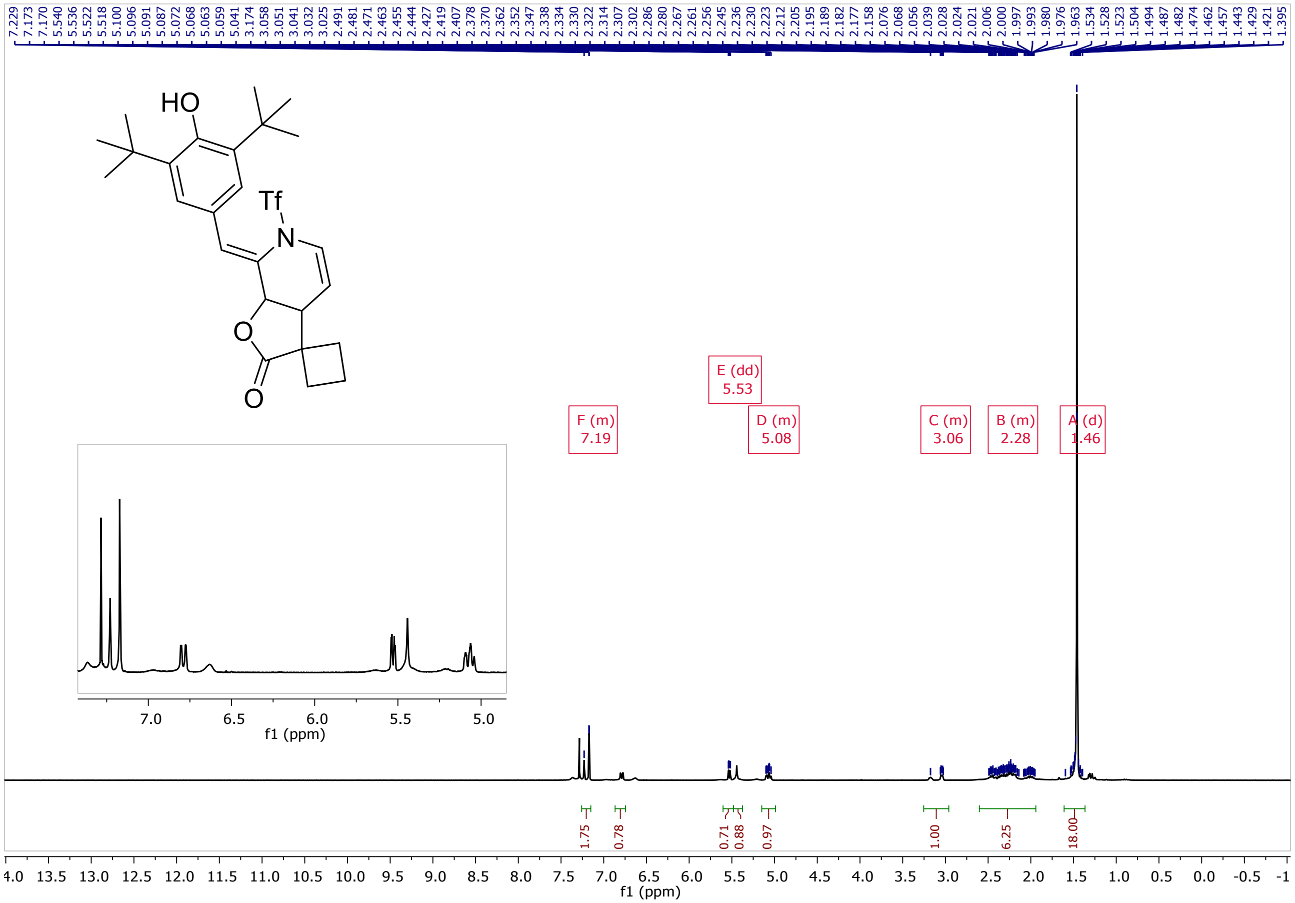

Figure S73. ^{1}H-NMR, 300 MHz, $CDCl_3$ for 4tb

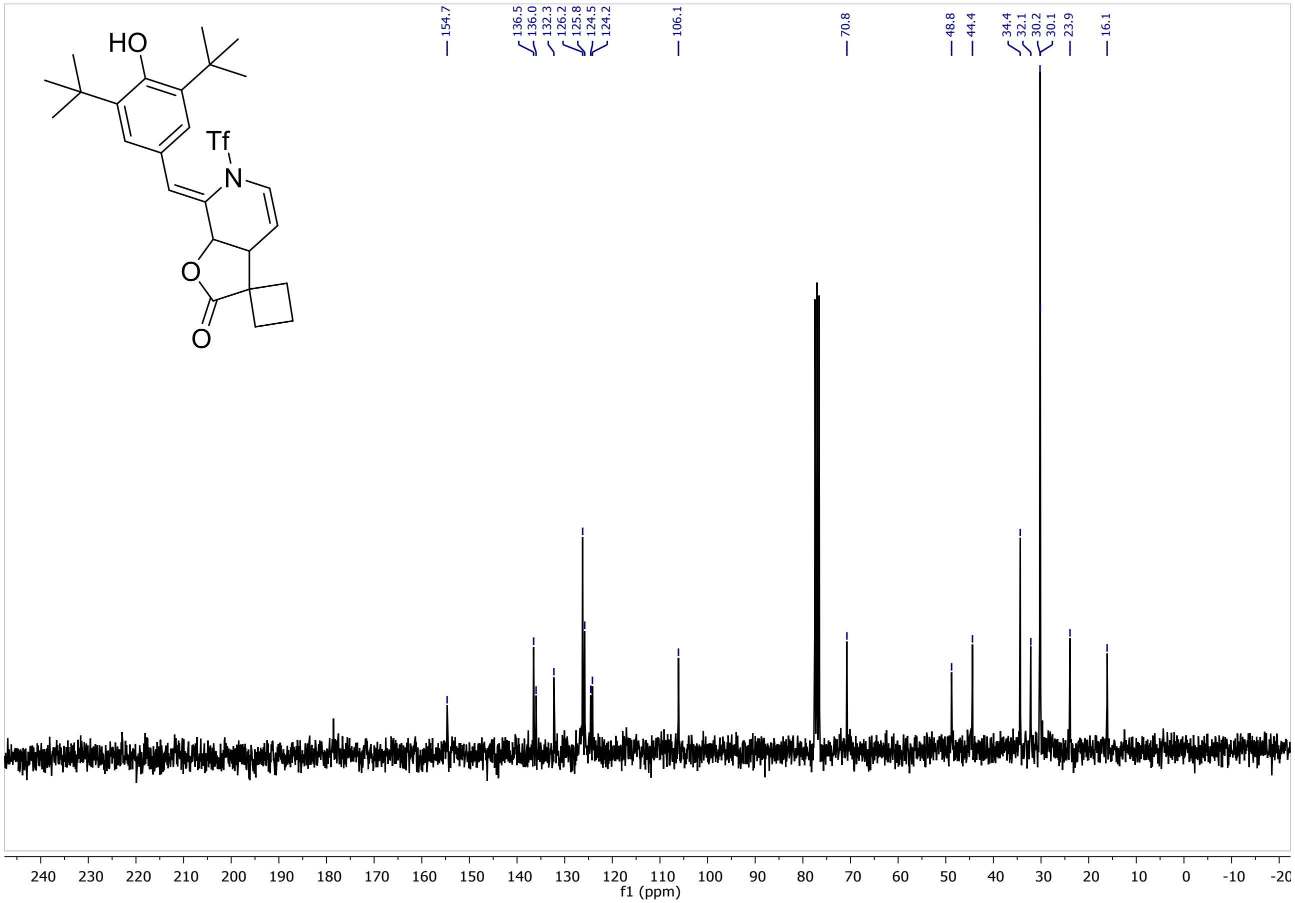

Figure S74. $^{13}C\{^{1}H\}$**-NMR, 75 MHz,** $CDCl_3$ **for 4tb**

(7Z)-7-(3,5-di-tert-butyl-4-hydroxybenzylidene)-3-spiro[5.4]-6-((trifluoromethyl)sulfonyl)-3a,6,7,7a-tetrahydrofuro[2,3-c]pyridin-2(3H)-one (4ud): 145mg, 45% Yield (White solid), ^{1}H NMR (300 MHz, Chloroform-*d*) δ 7.16 (d, *J* = 5.0 Hz, 3H), 5.74 (d, *J* = 6.9 Hz, 1H), 5.46 (s, 1H), 3.15 (d, *J* = 6.8 Hz, 1H), 2.19 – 1.19 (m, 28H); ^{13}C{^{1}H} NMR (75 MHz, $CDCl_3$) δ 177.3, 154.9, 136.6, 136.1, 133.7, 128.5, 126.9, 126.5, 126.3, 126.1, 124.4, 123.9, 108.3, 77.0, 76.6, 74.4, 60.4, 51.7, 48.8, 35.1, 34.3, 30.2, 30.1, 29.7, 25.0, 22.3, 21.8, 21.0, 14.2; HRMS (DART-ESI$^+$) m/z: [M+H]$^+$ Calcd for $C_{28}H_{36}BrF_3NO_5S$, 634.1444, Found, 634.1456.

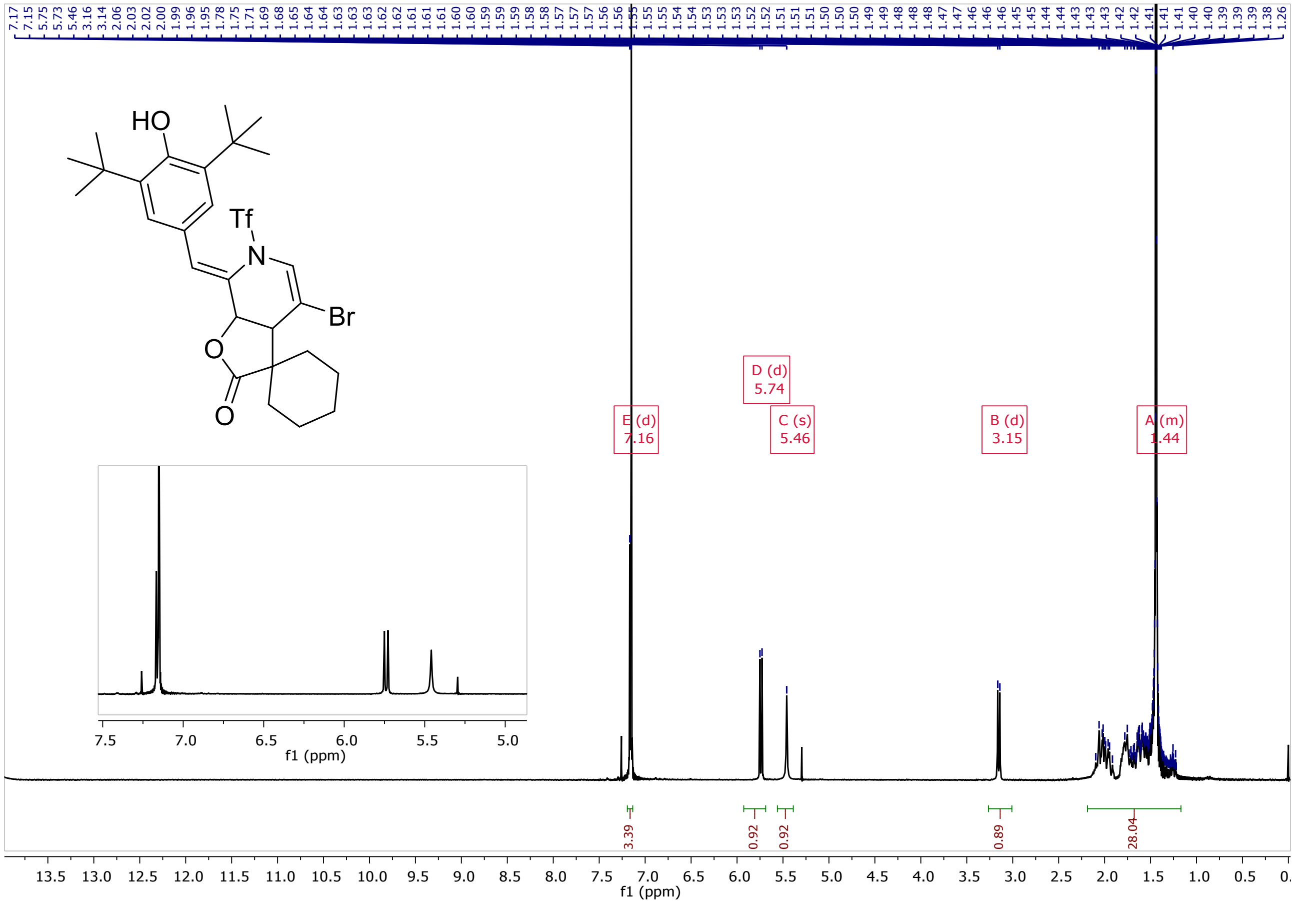

Figure S75. ^{1}H-NMR, 300 MHz, $CDCl_3$ for 4ud

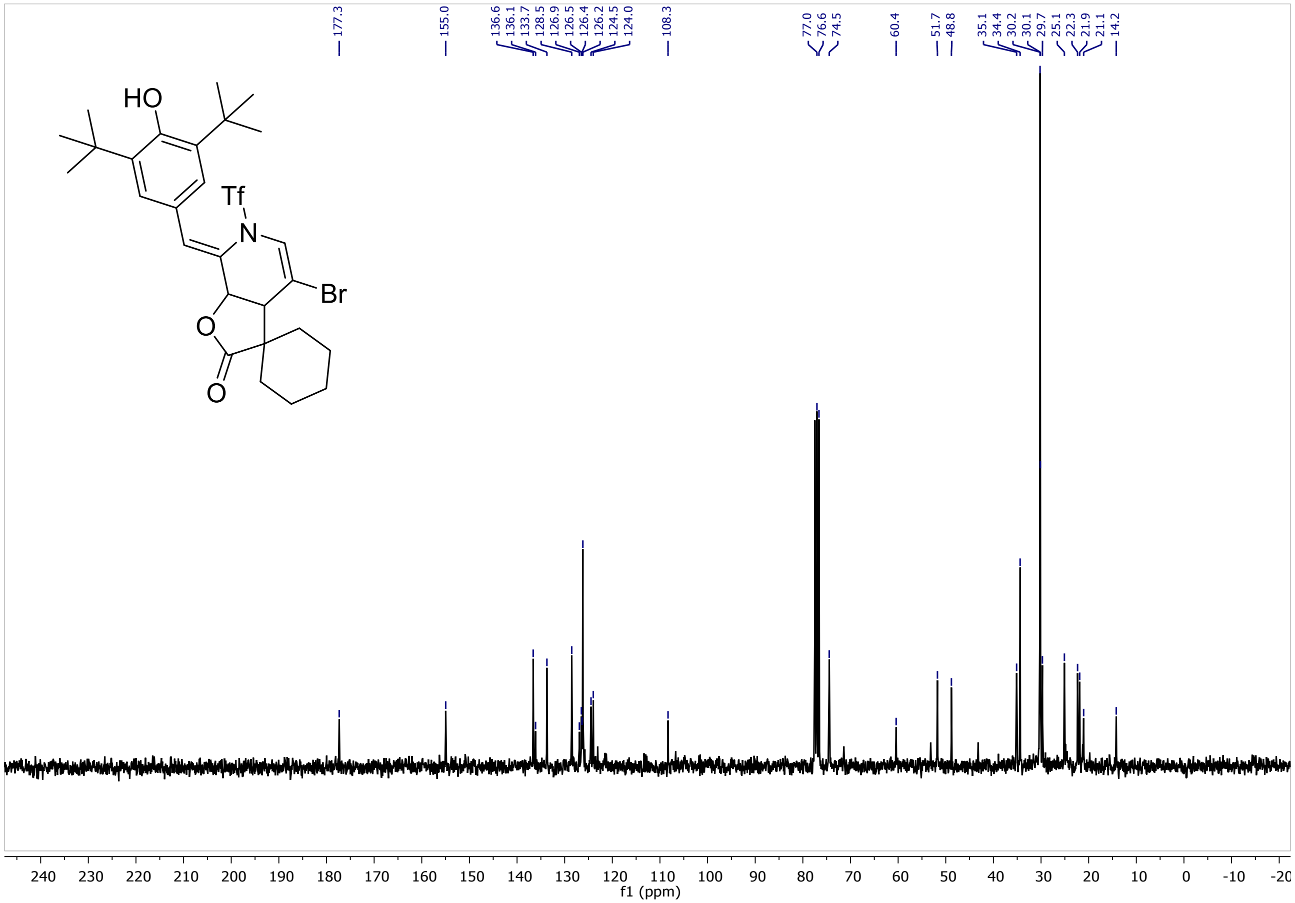

Figure S76. $^{13}C\{^{1}H\}$**-NMR, 75 MHz,** $CDCl_3$ **for 4ud**

Data:U833 LJBPBr2Py6Cy
Sample Name:M. en C. Saulo C. Rosales
Description:
Ionization Mode:ESI+
History:Determine m/z[Peak Detect[Centroid,30,Area];Correct Base[5.0%]];Correct Base[5.0%];Average(MS[1] 1..1)

Acquired:6/26/2024 1:41:25 PM
Operator:AccuTOF
Mass Calibration data:CAL_PEG_600_ALUMNOS_2024
Created:6/26/2024 1:56:51 PM
Created by:AccuTOF

Charge number:1
Tolerance:100.00(ppm), 5.00 .. 15.00(mmu)
Unsaturation Number:-1.5 .. 1000.0 (Fraction:Both)
Element:^{12}C:28 .. 28, ^{1}H:36 .. 36, ^{79}Br:0 .. 1, ^{81}Br:0 .. 1, ^{19}F:3 .. 3, ^{14}N:1 .. 1, ^{16}O:5 .. 5, ^{32}S:1 .. 1

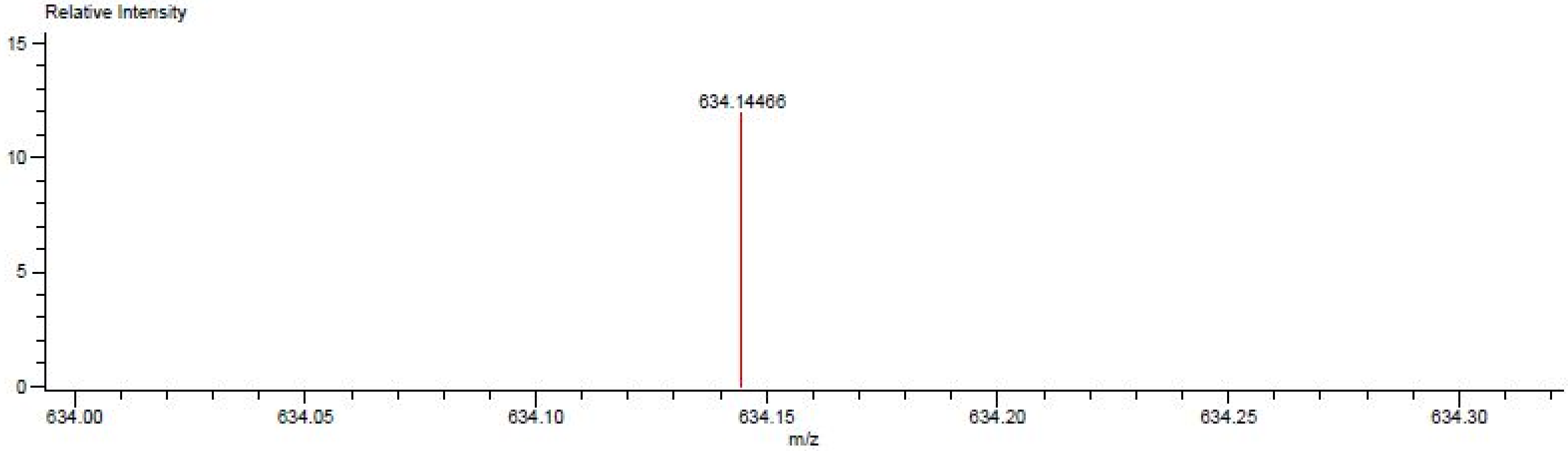

Mass	Intensity	Calc. Mass	Mass Difference (mmu)	Mass Difference (ppm)	Possible Formula	Unsaturation Number
634.14466	9109.32	634.14497	-0.31	-0.48	$^{12}C_{28}{}^{1}H_{36}{}^{79}Br_{1}{}^{19}F_{3}{}^{14}N_{1}{}^{16}O_{5}{}^{32}S_{1}$	10.5

Figure S77. HRMS, DART-ESI⁺ (TOF) for 4ud

7-(3,5-di-tert-butyl-4-hydroxyphenyl)-6,6-dimethyl-6,7-dihydro-5H-indeno[5,6-d][1,3]dioxol-5-one (5na): ^{1}H NMR for 5na (300 MHz, Chloroform-*d*) δ 7.17 (s, 1H), 6.76 (d, *J* = 5.0 Hz, 3H), 6.08 (dd, *J* = 11.3, 1.2 Hz, 2H), 5.13 (s, 1H), 4.10 (s, 1H), 1.39 (s, 18H), 1.29 (s, 3H), 0.64 (s, 3H); ^{13}C{^{1}H} NMR (75 MHz, $CDCl_3$) δ 209.2, 154.3, 152.7, 152.5, 148.5, 135.6, 130.3, 129.9, 125.5, 106.1, 102.4, 102.1, 57.4, 51.2, 34.3, 30.3, 25.3, 22.9.

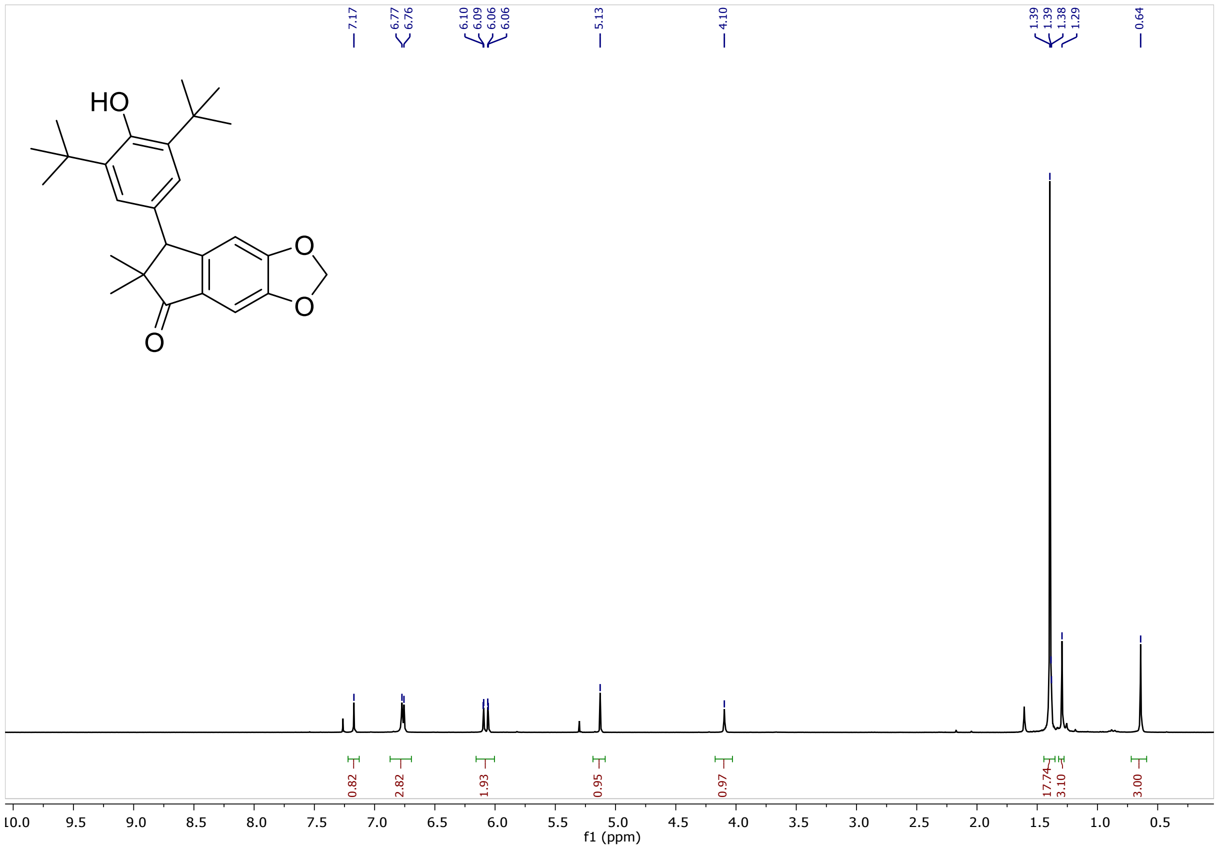

Figure S78. ^{1}H-NMR, 300 MHz, $CDCl_3$ for 5na

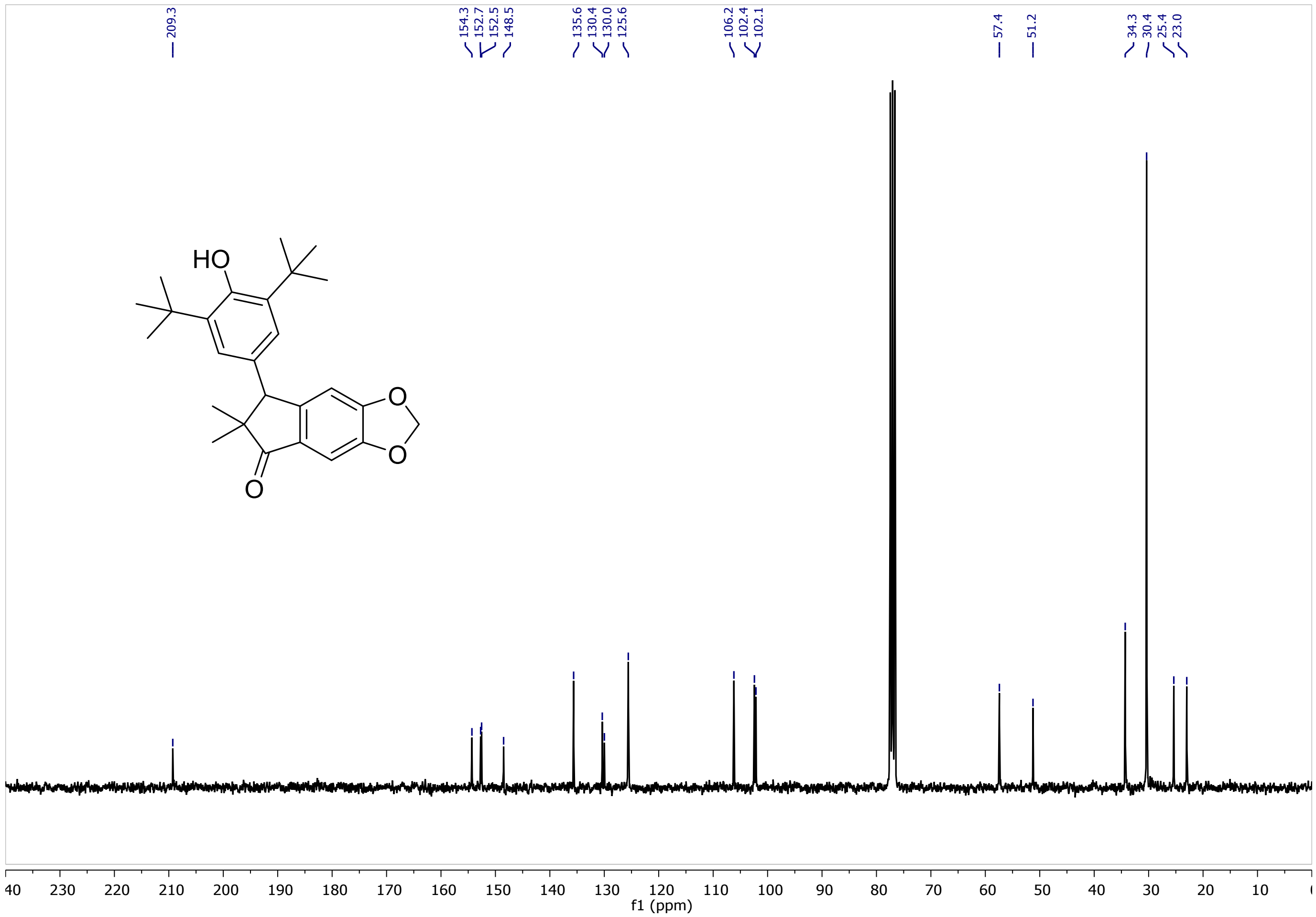

Figure S79. $^{13}C\{^{1}H\}$**-NMR, 75 MHz,** $CDCl_3$ **for 5na**

Data:U-1318 IndDoxA
Sample Name:M. en C. Saulo C. Rosales
Description:
Ionization Mode:ESI+
History:Determine m/z[Peak Detect[Centroid,30,Area];Correct Base[5.0%]];Correct Base[5.0%];Average(MS[1] 0..0)

Acquired:10/22/2024 1:36:23 PM
Operator:AccuTOF
Mass Calibration data:CAL_PEG_600_JEOL_8-OCT-2...
Created:10/22/2024 1:59:11 PM
Created by:AccuTOF

Charge number:1
Tolerance:100.00(ppm), 5.00 .. 15.00(mmu)
Unsaturation Number:-1.5 .. 100.0 (Fraction:.5)
Element:^{12}C:0 .. 50, ^{1}H:0 .. 50, ^{14}N:0 .. 0, ^{16}O:0 .. 5

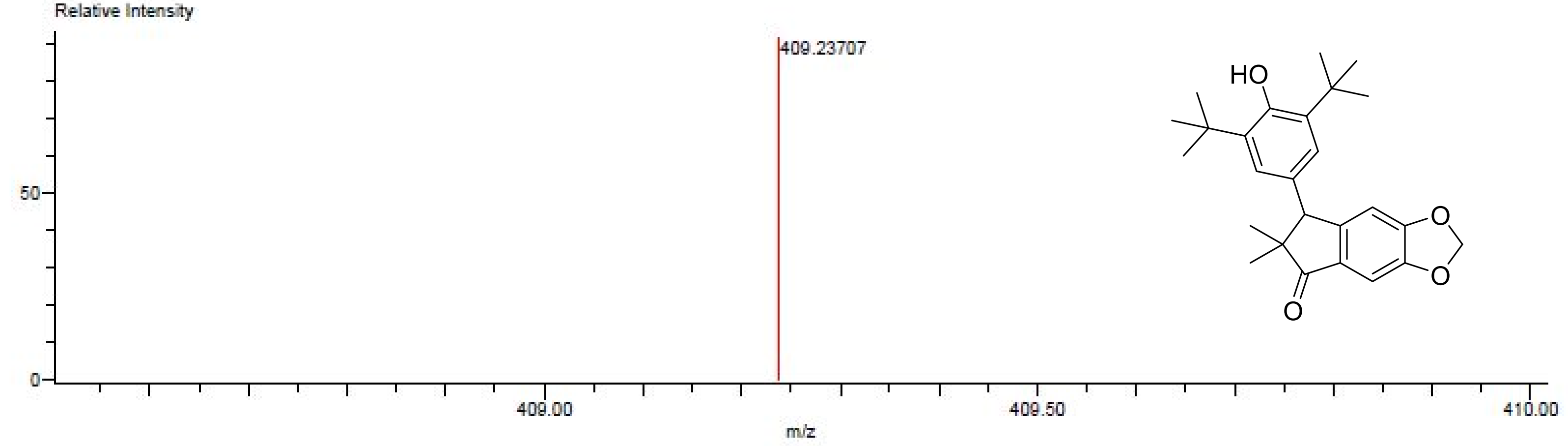

Mass	Intensity	Calc. Mass	Mass Difference (mmu)	Mass Difference (ppm)	Possible Formula	Unsaturation Number
409.23707	15689.88	409.23788	-0.81	-1.98	$^{12}C_{26}{}^{1}H_{33}{}^{16}O_4$	10.5

Figure S80. HRMS, DART-ESI$^+$ (TOF) for 5na

X-Ray data

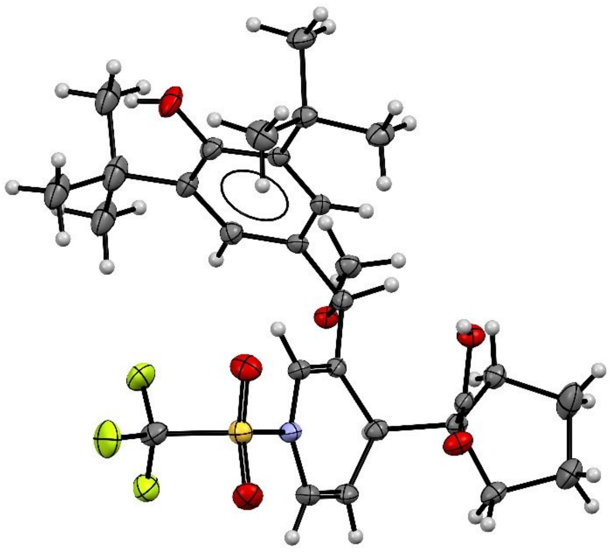

Table 1. Crystal data and structure refinement for **4vI**, **CCDC: 2366600.**

Identification code	shelx	
Empirical formula	C28 H38 F3 N O6 S	
Formula weight	573.65	
Temperature	130(2) K	
Wavelength	0.71073 Å	
Crystal system	Monoclinic	
Space group	P 21/c	
Unit cell dimensions	a = 14.1811(15) Å	α= 90°.
	b = 10.7927(9) Å	β= 106.832(12)°.
	c = 19.776(2) Å	γ = 90°.
Volume	2897.0(5) $Å^3$	
Z	4	
Density (calculated)	1.315 Mg/m^3	
Absorption coefficient	0.172 mm^{-1}	
F(000)	1216	
Crystal size	0.550 x 0.410 x 0.180 mm^3	
Theta range for data collection	3.666 to 29.511°.	

Index ranges	-19<=h<=15, -9<=k<=14, -27<=l<=22
Reflections collected	15365
Independent reflections	6904 [R(int) = 0.0469]
Completeness to theta = 25.242°	99.7 %
Refinement method	Full-matrix least-squares on F^2
Data / restraints / parameters	6904 / 3 / 356
Goodness-of-fit on F^2	1.026
Final R indices [I>2sigma(I)]	R1 = 0.0585, wR2 = 0.1150
R indices (all data)	R1 = 0.1037, wR2 = 0.1412
Extinction coefficient	n/a
Largest diff. peak and hole	0.644 and -0.434 e.Å^{-3}

Table 2. Atomic coordinates (x 10^4) and equivalent isotropic displacement parameters ($Å^2$x 10^3) for **4vI**. U(eq) is defined as one third of the trace of the orthogonalized U^{ij} tensor.

	x	y	z	U(eq)
C(1)	3295(2)	8850(2)	3853(1)	19(1)
C(2)	2645(2)	9350(2)	3256(1)	22(1)
C(3)	1631(2)	9401(2)	3162(1)	23(1)
C(4)	1289(2)	8871(2)	3697(1)	23(1)
C(5)	1922(2)	8359(2)	4323(1)	18(1)
C(6)	2927(2)	8379(2)	4383(1)	19(1)
C(7)	956(2)	10032(3)	2491(2)	39(1)
C(8)	1538(2)	7788(2)	4906(1)	23(1)
C(9)	404(3)	11141(4)	2709(2)	39(1)
C(9P)	-115(8)	10262(12)	2426(7)	39(1)
C(10)	206(3)	9118(4)	2032(2)	39(1)
C(10P)	1066(9)	9260(11)	1847(6)	39(1)
C(11)	1539(3)	10574(4)	2014(2)	39(1)
C(11P)	1433(9)	11295(11)	2412(7)	39(1)
C(12)	948(2)	8747(3)	5199(2)	32(1)
C(13)	882(2)	6657(3)	4614(2)	34(1)
C(14)	2386(2)	7332(2)	5533(1)	28(1)
C(15)	4380(2)	8706(2)	3915(1)	19(1)
C(16)	3946(2)	6576(2)	3479(1)	20(1)
C(17)	4632(2)	7451(2)	3665(1)	18(1)
C(18)	5694(2)	7216(2)	3665(1)	20(1)
C(19)	5714(2)	6129(2)	3191(1)	23(1)
C(20)	5009(2)	5285(2)	2997(1)	23(1)
C(21)	6432(2)	7046(2)	4430(1)	19(1)
C(22)	7441(2)	6536(2)	4408(1)	25(1)
C(23)	8138(2)	6841(3)	5136(2)	40(1)
C(24)	7726(2)	8001(3)	5394(2)	44(1)
C(25)	6730(2)	8265(2)	4849(1)	23(1)
C(26)	5927(2)	6128(2)	4797(1)	19(1)
C(27)	2540(2)	4976(2)	2093(2)	28(1)
C(28)	4816(2)	10816(2)	3806(1)	25(1)

F(1)	3109(1)	5080(2)	1668(1)	36(1)
F(2)	2139(1)	6073(2)	2140(1)	40(1)
F(3)	1812(1)	4184(2)	1794(1)	46(1)
N(1)	4136(1)	5399(2)	3217(1)	22(1)
O(1)	292(1)	8817(2)	3643(1)	33(1)
O(2)	4723(1)	9602(1)	3506(1)	21(1)
O(3)	5417(1)	6621(2)	5187(1)	25(1)
O(4)	5931(1)	5003(2)	4685(1)	27(1)
O(5)	2605(1)	4520(2)	3395(1)	31(1)
O(6)	3642(1)	3250(2)	2826(1)	31(1)
S(1)	3247(1)	4402(1)	2962(1)	24(1)

Table 3. Bond lengths [Å] and angles [°] for **4vI**.

C(1)-C(2)	1.380(3)
C(1)-C(6)	1.394(3)
C(1)-C(15)	1.516(3)
C(2)-C(3)	1.396(3)
C(2)-H(2)	0.9500
C(3)-C(4)	1.407(4)
C(3)-C(7)	1.551(4)
C(4)-O(1)	1.388(3)
C(4)-C(5)	1.414(3)
C(5)-C(6)	1.395(3)
C(5)-C(8)	1.539(3)
C(6)-H(6)	0.9500
C(7)-C(9P)	1.507(11)
C(7)-C(11)	1.537(5)
C(7)-C(10)	1.538(5)
C(7)-C(11P)	1.549(11)
C(7)-C(9)	1.559(5)
C(7)-C(10P)	1.566(11)
C(8)-C(14)	1.537(4)
C(8)-C(13)	1.543(4)
C(8)-C(12)	1.545(3)
C(9)-H(9A)	0.9800
C(9)-H(9B)	0.9800
C(9)-H(9C)	0.9800
C(9P)-H(9PA)	0.9800
C(9P)-H(9PB)	0.9800
C(9P)-H(9PC)	0.9800
C(10)-H(10A)	0.9800
C(10)-H(10B)	0.9800
C(10)-H(10C)	0.9800
C(10P)-H(10D)	0.9800
C(10P)-H(10E)	0.9800
C(10P)-H(10F)	0.9800
C(11)-H(11A)	0.9800

C(11)-H(11B)	0.9800
C(11)-H(11C)	0.9800
C(11P)-H(11D)	0.9800
C(11P)-H(11E)	0.9800
C(11P)-H(11F)	0.9800
C(12)-H(12A)	0.9800
C(12)-H(12B)	0.9800
C(12)-H(12C)	0.9800
C(13)-H(13A)	0.9800
C(13)-H(13B)	0.9800
C(13)-H(13C)	0.9800
C(14)-H(14A)	0.9800
C(14)-H(14B)	0.9800
C(14)-H(14C)	0.9800
C(15)-O(2)	1.432(3)
C(15)-C(17)	1.521(3)
C(15)-H(15)	1.0000
C(16)-C(17)	1.330(3)
C(16)-N(1)	1.427(3)
C(16)-H(16)	0.9500
C(17)-C(18)	1.527(3)
C(18)-C(19)	1.507(3)
C(18)-C(21)	1.582(3)
C(18)-H(18)	1.0000
C(19)-C(20)	1.325(3)
C(19)-H(19)	0.9500
C(20)-N(1)	1.431(3)
C(20)-H(20)	0.9500
C(21)-C(26)	1.523(3)
C(21)-C(22)	1.545(3)
C(21)-C(25)	1.547(3)
C(22)-C(23)	1.528(4)
C(22)-H(22A)	0.9900
C(22)-H(22B)	0.9900
C(23)-C(24)	1.530(4)
C(23)-H(23A)	0.9900

C(23)-H(23B)	0.9900
C(24)-C(25)	1.534(4)
C(24)-H(24A)	0.9900
C(24)-H(24B)	0.9900
C(25)-H(25A)	0.9900
C(25)-H(25B)	0.9900
C(26)-O(4)	1.235(3)
C(26)-O(3)	1.313(3)
C(27)-F(2)	1.328(3)
C(27)-F(1)	1.328(3)
C(27)-F(3)	1.339(3)
C(27)-S(1)	1.830(3)
C(28)-O(2)	1.428(3)
C(28)-H(28A)	0.9800
C(28)-H(28B)	0.9800
C(28)-H(28C)	0.9800
N(1)-S(1)	1.622(2)
O(1)-H(1)	0.8400
O(3)-H(3)	0.8400
O(5)-S(1)	1.4241(19)
O(6)-S(1)	1.4209(18)

C(2)-C(1)-C(6)	118.8(2)
C(2)-C(1)-C(15)	121.3(2)
C(6)-C(1)-C(15)	119.7(2)
C(1)-C(2)-C(3)	122.5(2)
C(1)-C(2)-H(2)	118.8
C(3)-C(2)-H(2)	118.8
C(2)-C(3)-C(4)	116.7(2)
C(2)-C(3)-C(7)	119.2(2)
C(4)-C(3)-C(7)	124.1(2)
O(1)-C(4)-C(3)	121.7(2)
O(1)-C(4)-C(5)	115.2(2)
C(3)-C(4)-C(5)	123.2(2)
C(6)-C(5)-C(4)	116.2(2)
C(6)-C(5)-C(8)	121.1(2)

C(4)-C(5)-C(8)	122.7(2)
C(1)-C(6)-C(5)	122.5(2)
C(1)-C(6)-H(6)	118.7
C(5)-C(6)-H(6)	118.7
C(11)-C(7)-C(10)	106.6(3)
C(9P)-C(7)-C(11P)	107.7(7)
C(9P)-C(7)-C(3)	120.1(5)
C(11)-C(7)-C(3)	112.6(2)
C(10)-C(7)-C(3)	111.9(3)
C(11P)-C(7)-C(3)	107.1(5)
C(11)-C(7)-C(9)	106.3(3)
C(10)-C(7)-C(9)	109.8(3)
C(3)-C(7)-C(9)	109.5(3)
C(9P)-C(7)-C(10P)	110.7(7)
C(11P)-C(7)-C(10P)	103.8(7)
C(3)-C(7)-C(10P)	106.2(5)
C(14)-C(8)-C(5)	111.7(2)
C(14)-C(8)-C(13)	107.2(2)
C(5)-C(8)-C(13)	110.0(2)
C(14)-C(8)-C(12)	107.1(2)
C(5)-C(8)-C(12)	111.1(2)
C(13)-C(8)-C(12)	109.7(2)
C(7)-C(9)-H(9A)	109.5
C(7)-C(9)-H(9B)	109.5
H(9A)-C(9)-H(9B)	109.5
C(7)-C(9)-H(9C)	109.5
H(9A)-C(9)-H(9C)	109.5
H(9B)-C(9)-H(9C)	109.5
C(7)-C(9P)-H(9PA)	109.5
C(7)-C(9P)-H(9PB)	109.5
H(9PA)-C(9P)-H(9PB)	109.5
C(7)-C(9P)-H(9PC)	109.5
H(9PA)-C(9P)-H(9PC)	109.5
H(9PB)-C(9P)-H(9PC)	109.5
C(7)-C(10)-H(10A)	109.5
C(7)-C(10)-H(10B)	109.5

H(10A)-C(10)-H(10B)	109.5
C(7)-C(10)-H(10C)	109.5
H(10A)-C(10)-H(10C)	109.5
H(10B)-C(10)-H(10C)	109.5
C(7)-C(10P)-H(10D)	109.5
C(7)-C(10P)-H(10E)	109.5
H(10D)-C(10P)-H(10E)	109.5
C(7)-C(10P)-H(10F)	109.5
H(10D)-C(10P)-H(10F)	109.5
H(10E)-C(10P)-H(10F)	109.5
C(7)-C(11)-H(11A)	109.5
C(7)-C(11)-H(11B)	109.5
H(11A)-C(11)-H(11B)	109.5
C(7)-C(11)-H(11C)	109.5
H(11A)-C(11)-H(11C)	109.5
H(11B)-C(11)-H(11C)	109.5
C(7)-C(11P)-H(11D)	109.5
C(7)-C(11P)-H(11E)	109.5
H(11D)-C(11P)-H(11E)	109.5
C(7)-C(11P)-H(11F)	109.5
H(11D)-C(11P)-H(11F)	109.5
H(11E)-C(11P)-H(11F)	109.5
C(8)-C(12)-H(12A)	109.5
C(8)-C(12)-H(12B)	109.5
H(12A)-C(12)-H(12B)	109.5
C(8)-C(12)-H(12C)	109.5
H(12A)-C(12)-H(12C)	109.5
H(12B)-C(12)-H(12C)	109.5
C(8)-C(13)-H(13A)	109.5
C(8)-C(13)-H(13B)	109.5
H(13A)-C(13)-H(13B)	109.5
C(8)-C(13)-H(13C)	109.5
H(13A)-C(13)-H(13C)	109.5
H(13B)-C(13)-H(13C)	109.5
C(8)-C(14)-H(14A)	109.5
C(8)-C(14)-H(14B)	109.5

H(14A)-C(14)-H(14B)	109.5
C(8)-C(14)-H(14C)	109.5
H(14A)-C(14)-H(14C)	109.5
H(14B)-C(14)-H(14C)	109.5
O(2)-C(15)-C(1)	112.85(18)
O(2)-C(15)-C(17)	105.53(18)
C(1)-C(15)-C(17)	113.19(19)
O(2)-C(15)-H(15)	108.4
C(1)-C(15)-H(15)	108.4
C(17)-C(15)-H(15)	108.4
C(17)-C(16)-N(1)	122.2(2)
C(17)-C(16)-H(16)	118.9
N(1)-C(16)-H(16)	118.9
C(16)-C(17)-C(15)	120.0(2)
C(16)-C(17)-C(18)	121.7(2)
C(15)-C(17)-C(18)	118.23(19)
C(19)-C(18)-C(17)	109.0(2)
C(19)-C(18)-C(21)	112.24(19)
C(17)-C(18)-C(21)	113.49(19)
C(19)-C(18)-H(18)	107.3
C(17)-C(18)-H(18)	107.3
C(21)-C(18)-H(18)	107.3
C(20)-C(19)-C(18)	124.5(2)
C(20)-C(19)-H(19)	117.7
C(18)-C(19)-H(19)	117.7
C(19)-C(20)-N(1)	120.0(2)
C(19)-C(20)-H(20)	120.0
N(1)-C(20)-H(20)	120.0
C(26)-C(21)-C(22)	110.46(19)
C(26)-C(21)-C(25)	113.0(2)
C(22)-C(21)-C(25)	101.91(19)
C(26)-C(21)-C(18)	104.98(18)
C(22)-C(21)-C(18)	112.1(2)
C(25)-C(21)-C(18)	114.58(19)
C(23)-C(22)-C(21)	104.3(2)
C(23)-C(22)-H(22A)	110.9

C(21)-C(22)-H(22A)	110.9
C(23)-C(22)-H(22B)	110.9
C(21)-C(22)-H(22B)	110.9
H(22A)-C(22)-H(22B)	108.9
C(22)-C(23)-C(24)	106.6(2)
C(22)-C(23)-H(23A)	110.4
C(24)-C(23)-H(23A)	110.4
C(22)-C(23)-H(23B)	110.4
C(24)-C(23)-H(23B)	110.4
H(23A)-C(23)-H(23B)	108.6
C(23)-C(24)-C(25)	106.3(2)
C(23)-C(24)-H(24A)	110.5
C(25)-C(24)-H(24A)	110.5
C(23)-C(24)-H(24B)	110.5
C(25)-C(24)-H(24B)	110.5
H(24A)-C(24)-H(24B)	108.7
C(24)-C(25)-C(21)	105.7(2)
C(24)-C(25)-H(25A)	110.6
C(21)-C(25)-H(25A)	110.6
C(24)-C(25)-H(25B)	110.6
C(21)-C(25)-H(25B)	110.6
H(25A)-C(25)-H(25B)	108.7
O(4)-C(26)-O(3)	122.4(2)
O(4)-C(26)-C(21)	121.8(2)
O(3)-C(26)-C(21)	115.5(2)
F(2)-C(27)-F(1)	108.7(2)
F(2)-C(27)-F(3)	108.0(2)
F(1)-C(27)-F(3)	107.8(2)
F(2)-C(27)-S(1)	111.39(19)
F(1)-C(27)-S(1)	110.78(17)
F(3)-C(27)-S(1)	109.97(19)
O(2)-C(28)-H(28A)	109.5
O(2)-C(28)-H(28B)	109.5
H(28A)-C(28)-H(28B)	109.5
O(2)-C(28)-H(28C)	109.5
H(28A)-C(28)-H(28C)	109.5

H(28B)-C(28)-H(28C)	109.5
C(16)-N(1)-C(20)	117.2(2)
C(16)-N(1)-S(1)	119.44(16)
C(20)-N(1)-S(1)	120.78(16)
C(4)-O(1)-H(1)	109.5
C(28)-O(2)-C(15)	113.03(18)
C(26)-O(3)-H(3)	109.5
O(6)-S(1)-O(5)	123.23(11)
O(6)-S(1)-N(1)	108.97(11)
O(5)-S(1)-N(1)	109.24(11)
O(6)-S(1)-C(27)	104.89(12)
O(5)-S(1)-C(27)	105.20(12)
N(1)-S(1)-C(27)	103.40(11)

Symmetry transformations used to generate equivalent atoms:

Table 4. Anisotropic displacement parameters (Å^{2}x 10^3)for **4vI**. The anisotropic displacement factor exponent takes the form: $-2\pi^2[h^2a^{*2}U^{11} + ... + 2 h k a^* b^* U^{12}]$

	U11	U22	U33	U23	U13	U12
C(1)	18(1)	18(1)	20(1)	-3(1)	7(1)	2(1)
C(2)	20(1)	24(1)	22(1)	0(1)	6(1)	0(1)
C(3)	18(1)	26(1)	24(1)	1(1)	3(1)	3(1)
C(4)	15(1)	26(1)	27(1)	-1(1)	6(1)	1(1)
C(5)	19(1)	17(1)	21(1)	-3(1)	7(1)	1(1)
C(6)	17(1)	20(1)	18(1)	-3(1)	3(1)	2(1)
C(7)	25(1)	53(1)	35(1)	17(1)	2(1)	4(1)
C(8)	20(1)	25(1)	26(1)	-1(1)	10(1)	0(1)
C(9)	25(1)	53(1)	35(1)	17(1)	2(1)	4(1)
C(9P)	25(1)	53(1)	35(1)	17(1)	2(1)	4(1)
C(10)	25(1)	53(1)	35(1)	17(1)	2(1)	4(1)
C(10P)	25(1)	53(1)	35(1)	17(1)	2(1)	4(1)
C(11)	25(1)	53(1)	35(1)	17(1)	2(1)	4(1)
C(11P)	25(1)	53(1)	35(1)	17(1)	2(1)	4(1)
C(12)	29(2)	40(2)	31(2)	2(1)	16(1)	10(1)
C(13)	35(2)	33(2)	38(2)	-2(1)	16(1)	-9(1)
C(14)	27(1)	33(1)	29(1)	8(1)	14(1)	5(1)
C(15)	18(1)	20(1)	18(1)	0(1)	4(1)	0(1)
C(16)	18(1)	22(1)	22(1)	-2(1)	6(1)	4(1)
C(17)	17(1)	24(1)	14(1)	2(1)	6(1)	2(1)
C(18)	19(1)	21(1)	21(1)	1(1)	8(1)	0(1)
C(19)	20(1)	30(1)	21(1)	0(1)	10(1)	4(1)
C(20)	22(1)	26(1)	23(1)	-4(1)	8(1)	3(1)
C(21)	19(1)	19(1)	19(1)	1(1)	7(1)	1(1)
C(22)	18(1)	27(1)	31(1)	4(1)	10(1)	4(1)
C(23)	23(2)	51(2)	40(2)	-2(2)	1(1)	7(1)
C(24)	30(2)	36(2)	52(2)	-5(2)	-10(2)	1(1)
C(25)	20(1)	24(1)	24(1)	1(1)	6(1)	-1(1)
C(26)	15(1)	24(1)	18(1)	3(1)	3(1)	5(1)
C(27)	21(1)	28(1)	35(2)	0(1)	10(1)	2(1)
C(28)	26(1)	21(1)	28(1)	-2(1)	8(1)	1(1)

F(1)	31(1)	51(1)	26(1)	4(1)	9(1)	6(1)
F(2)	36(1)	39(1)	39(1)	0(1)	3(1)	16(1)
F(3)	29(1)	54(1)	45(1)	-12(1)	-2(1)	-12(1)
N(1)	18(1)	24(1)	24(1)	-5(1)	7(1)	1(1)
O(1)	14(1)	49(1)	34(1)	10(1)	4(1)	3(1)
O(2)	21(1)	20(1)	22(1)	0(1)	8(1)	-1(1)
O(3)	26(1)	26(1)	27(1)	2(1)	13(1)	-1(1)
O(4)	26(1)	22(1)	36(1)	5(1)	14(1)	2(1)
O(5)	31(1)	32(1)	35(1)	-2(1)	18(1)	-8(1)
O(6)	34(1)	20(1)	37(1)	-1(1)	10(1)	0(1)
S(1)	23(1)	22(1)	25(1)	-2(1)	7(1)	-2(1)

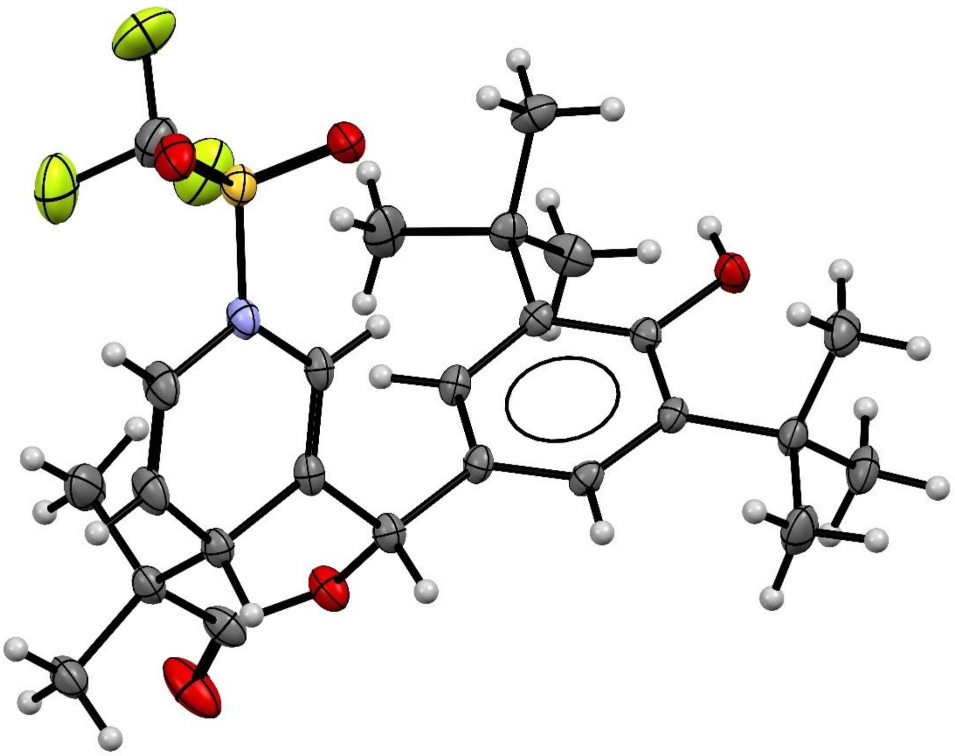

Table 1. Crystal data and structure refinement for **4v**, **CCDC: 2366599.**

Identification code	shelx	
Empirical formula	C_{25} H32 F3 N O5 S	
Formula weight	515.57	
Temperature	130(2) K	
Wavelength	0.71073 Å	
Crystal system	Monoclinic	
Space group	P 21/n	
Unit cell dimensions	a = 13.4604(8) Å	α= 90°.
	b = 8.3982(6) Å	β= 91.176(5)°.
	c = 24.0097(19) Å	γ = 90°.
Volume	2713.6(3) $Å^3$	
Z	4	
Density (calculated)	1.262 Mg/m^3	

Absorption coefficient	0.173 mm^{-1}
F(000)	1088
Crystal size	0.420 x 0.400 x 0.160 mm^3
Theta range for data collection	3.441 to 29.454°.
Index ranges	-17<=h<=15, -11<=k<=11, -26<=l<=32
Reflections collected	14244
Independent reflections	6339 [R(int) = 0.0221]
Completeness to theta = 25.242°	99.7 %
Refinement method	Full-matrix least-squares on F^2
Data / restraints / parameters	6339 / 0 / 324
Goodness-of-fit on F^2	1.026
Final R indices [I>2sigma(I)]	R1 = 0.0420, wR2 = 0.0988
R indices (all data)	R1 = 0.0575, wR2 = 0.1083
Extinction coefficient	n/a
Largest diff. peak and hole	0.355 and -0.383 e.Å^{-3}

Table 2. Atomic coordinates (x 10^4) and equivalent isotropic displacement parameters ($Å^2$x 10^3) for **4v**. U(eq) is defined as one third of the trace of the orthogonalized U^{ij} tensor.

	x	y	z	U(eq)
C(1)	1320(1)	10088(2)	1318(1)	19(1)
C(2)	2589(1)	6987(2)	927(1)	20(1)
C(3)	2781(1)	7057(2)	1469(1)	19(1)
C(4)	1221(1)	8708(2)	1629(1)	18(1)
C(5)	636(1)	12384(2)	728(1)	21(1)
C(6)	282(1)	8084(2)	1710(1)	18(1)
C(7)	-438(1)	10171(2)	1163(1)	18(1)
C(8)	3692(1)	6363(2)	1747(1)	20(1)
C(9)	-571(1)	8791(2)	1483(1)	17(1)
C(10)	504(1)	10855(2)	1074(1)	18(1)
C(11)	4314(1)	7737(2)	2007(1)	22(1)
C(12)	4056(1)	5309(2)	804(1)	28(1)
C(13)	-1608(1)	8109(2)	1600(1)	22(1)
C(14)	2108(1)	7846(2)	1872(1)	20(1)
C(15)	3653(1)	8832(2)	2344(1)	26(1)
C(16)	4258(1)	5367(2)	1344(1)	29(1)
C(17)	-2230(1)	9354(2)	1906(1)	29(1)
C(18)	293(1)	12120(2)	118(1)	28(1)
C(19)	-2143(1)	7595(2)	1057(1)	30(1)
C(20)	1735(1)	12874(2)	705(1)	34(1)
C(21)	-1541(1)	6633(2)	1974(1)	33(1)
C(22)	5136(1)	7077(2)	2390(1)	29(1)
C(23)	85(1)	13799(2)	990(1)	28(1)
C(24)	3793(1)	8272(2)	-223(1)	37(1)
C(25)	4766(1)	8795(2)	1554(1)	36(1)
F(1)	3371(1)	9459(1)	43(1)	52(1)
F(2)	4732(1)	8149(2)	-46(1)	54(1)
F(3)	3787(1)	8609(2)	-761(1)	61(1)
N(1)	3238(1)	6165(2)	565(1)	22(1)
O(1)	2129(1)	6705(2)	-245(1)	29(1)
O(2)	2678(1)	8999(1)	2215(1)	26(1)

O(3)	-1291(1)	10820(1)	937(1)	24(1)
O(4)	3708(1)	5214(2)	-369(1)	33(1)
O(5)	3986(1)	9672(2)	2706(1)	46(1)
S(1)	3140(1)	6402(1)	-101(1)	24(1)

Table 3. Bond lengths [Å] and angles [°] for **4v**.

C(1)-C(4)	1.388(2)
C(1)-C(10)	1.392(2)
C(1)-H(1)	0.9500
C(2)-C(3)	1.321(2)
C(2)-N(1)	1.4241(18)
C(2)-H(2)	0.9500
C(3)-C(14)	1.495(2)
C(3)-C(8)	1.502(2)
C(4)-C(6)	1.385(2)
C(4)-C(14)	1.503(2)
C(5)-C(20)	1.537(2)
C(5)-C(10)	1.542(2)
C(5)-C(23)	1.542(2)
C(5)-C(18)	1.543(2)
C(6)-C(9)	1.393(2)
C(6)-H(6)	0.9500
C(7)-O(3)	1.3727(17)
C(7)-C(9)	1.405(2)
C(7)-C(10)	1.412(2)
C(8)-C(16)	1.497(2)
C(8)-C(11)	1.549(2)
C(8)-H(8)	1.0000
C(9)-C(13)	1.540(2)
C(11)-C(15)	1.523(2)
C(11)-C(22)	1.530(2)
C(11)-C(25)	1.540(2)
C(12)-C(16)	1.320(2)
C(12)-N(1)	1.426(2)
C(12)-H(12)	0.9500
C(13)-C(21)	1.532(2)
C(13)-C(17)	1.536(2)
C(13)-C(19)	1.537(2)
C(14)-O(2)	1.4766(18)
C(14)-H(14)	1.0000

C(15)-O(5)	1.201(2)
C(15)-O(2)	1.3487(18)
C(16)-H(16)	0.9500
C(17)-H(17A)	0.9800
C(17)-H(17B)	0.9800
C(17)-H(17C)	0.9800
C(18)-H(18A)	0.9800
C(18)-H(18B)	0.9800
C(18)-H(18C)	0.9800
C(19)-H(19A)	0.9800
C(19)-H(19B)	0.9800
C(19)-H(19C)	0.9800
C(20)-H(20A)	0.9800
C(20)-H(20B)	0.9800
C(20)-H(20C)	0.9800
C(21)-H(21A)	0.9800
C(21)-H(21B)	0.9800
C(21)-H(21C)	0.9800
C(22)-H(22A)	0.9800
C(22)-H(22B)	0.9800
C(22)-H(22C)	0.9800
C(23)-H(23A)	0.9800
C(23)-H(23B)	0.9800
C(23)-H(23C)	0.9800
C(24)-F(1)	1.320(2)
C(24)-F(3)	1.321(2)
C(24)-F(2)	1.330(2)
C(24)-S(1)	1.826(2)
C(25)-H(25A)	0.9800
C(25)-H(25B)	0.9800
C(25)-H(25C)	0.9800
N(1)-S(1)	1.6154(13)
O(1)-S(1)	1.4198(11)
O(3)-H(3)	0.8200
O(4)-S(1)	1.4182(12)

C(4)-C(1)-C(10)	121.96(14)
C(4)-C(1)-H(1)	119.0
C(10)-C(1)-H(1)	119.0
C(3)-C(2)-N(1)	120.78(13)
C(3)-C(2)-H(2)	119.6
N(1)-C(2)-H(2)	119.6
C(2)-C(3)-C(14)	123.24(13)
C(2)-C(3)-C(8)	124.16(13)
C(14)-C(3)-C(8)	112.60(12)
C(6)-C(4)-C(1)	119.26(13)
C(6)-C(4)-C(14)	118.92(13)
C(1)-C(4)-C(14)	121.80(13)
C(20)-C(5)-C(10)	111.39(13)
C(20)-C(5)-C(23)	106.26(13)
C(10)-C(5)-C(23)	111.08(12)
C(20)-C(5)-C(18)	105.87(13)
C(10)-C(5)-C(18)	110.86(12)
C(23)-C(5)-C(18)	111.18(13)
C(4)-C(6)-C(9)	122.11(14)
C(4)-C(6)-H(6)	118.9
C(9)-C(6)-H(6)	118.9
O(3)-C(7)-C(9)	115.45(13)
O(3)-C(7)-C(10)	121.68(13)
C(9)-C(7)-C(10)	122.87(13)
C(16)-C(8)-C(3)	110.58(12)
C(16)-C(8)-C(11)	113.49(13)
C(3)-C(8)-C(11)	108.64(12)
C(16)-C(8)-H(8)	108.0
C(3)-C(8)-H(8)	108.0
C(11)-C(8)-H(8)	108.0
C(6)-C(9)-C(7)	116.91(13)
C(6)-C(9)-C(13)	120.92(13)
C(7)-C(9)-C(13)	122.14(13)
C(1)-C(10)-C(7)	116.87(13)
C(1)-C(10)-C(5)	120.83(13)
C(7)-C(10)-C(5)	122.29(13)

C(15)-C(11)-C(22)	108.85(13)
C(15)-C(11)-C(25)	105.64(14)
C(22)-C(11)-C(25)	110.03(13)
C(15)-C(11)-C(8)	110.34(12)
C(22)-C(11)-C(8)	110.58(13)
C(25)-C(11)-C(8)	111.27(13)
C(16)-C(12)-N(1)	121.20(14)
C(16)-C(12)-H(12)	119.4
N(1)-C(12)-H(12)	119.4
C(21)-C(13)-C(17)	107.22(13)
C(21)-C(13)-C(19)	106.93(14)
C(17)-C(13)-C(19)	110.34(13)
C(21)-C(13)-C(9)	111.34(13)
C(17)-C(13)-C(9)	109.73(13)
C(19)-C(13)-C(9)	111.17(12)
O(2)-C(14)-C(3)	109.73(11)
O(2)-C(14)-C(4)	107.49(12)
C(3)-C(14)-C(4)	116.71(12)
O(2)-C(14)-H(14)	107.5
C(3)-C(14)-H(14)	107.5
C(4)-C(14)-H(14)	107.5
O(5)-C(15)-O(2)	116.89(15)
O(5)-C(15)-C(11)	121.72(15)
O(2)-C(15)-C(11)	121.17(13)
C(12)-C(16)-C(8)	123.89(14)
C(12)-C(16)-H(16)	118.1
C(8)-C(16)-H(16)	118.1
C(13)-C(17)-H(17A)	109.5
C(13)-C(17)-H(17B)	109.5
H(17A)-C(17)-H(17B)	109.5
C(13)-C(17)-H(17C)	109.5
H(17A)-C(17)-H(17C)	109.5
H(17B)-C(17)-H(17C)	109.5
C(5)-C(18)-H(18A)	109.5
C(5)-C(18)-H(18B)	109.5
H(18A)-C(18)-H(18B)	109.5

C(5)-C(18)-H(18C)	109.5
H(18A)-C(18)-H(18C)	109.5
H(18B)-C(18)-H(18C)	109.5
C(13)-C(19)-H(19A)	109.5
C(13)-C(19)-H(19B)	109.5
H(19A)-C(19)-H(19B)	109.5
C(13)-C(19)-H(19C)	109.5
H(19A)-C(19)-H(19C)	109.5
H(19B)-C(19)-H(19C)	109.5
C(5)-C(20)-H(20A)	109.5
C(5)-C(20)-H(20B)	109.5
H(20A)-C(20)-H(20B)	109.5
C(5)-C(20)-H(20C)	109.5
H(20A)-C(20)-H(20C)	109.5
H(20B)-C(20)-H(20C)	109.5
C(13)-C(21)-H(21A)	109.5
C(13)-C(21)-H(21B)	109.5
H(21A)-C(21)-H(21B)	109.5
C(13)-C(21)-H(21C)	109.5
H(21A)-C(21)-H(21C)	109.5
H(21B)-C(21)-H(21C)	109.5
C(11)-C(22)-H(22A)	109.5
C(11)-C(22)-H(22B)	109.5
H(22A)-C(22)-H(22B)	109.5
C(11)-C(22)-H(22C)	109.5
H(22A)-C(22)-H(22C)	109.5
H(22B)-C(22)-H(22C)	109.5
C(5)-C(23)-H(23A)	109.5
C(5)-C(23)-H(23B)	109.5
H(23A)-C(23)-H(23B)	109.5
C(5)-C(23)-H(23C)	109.5
H(23A)-C(23)-H(23C)	109.5
H(23B)-C(23)-H(23C)	109.5
F(1)-C(24)-F(3)	108.55(16)
F(1)-C(24)-F(2)	108.59(16)
F(3)-C(24)-F(2)	108.46(15)

F(1)-C(24)-S(1)	111.01(12)
F(3)-C(24)-S(1)	110.35(14)
F(2)-C(24)-S(1)	109.83(13)
C(11)-C(25)-H(25A)	109.5
C(11)-C(25)-H(25B)	109.5
H(25A)-C(25)-H(25B)	109.5
C(11)-C(25)-H(25C)	109.5
H(25A)-C(25)-H(25C)	109.5
H(25B)-C(25)-H(25C)	109.5
C(2)-N(1)-C(12)	118.50(12)
C(2)-N(1)-S(1)	120.41(10)
C(12)-N(1)-S(1)	120.53(11)
C(15)-O(2)-C(14)	123.48(12)
C(7)-O(3)-H(3)	109.3
O(4)-S(1)-O(1)	122.64(7)
O(4)-S(1)-N(1)	109.12(7)
O(1)-S(1)-N(1)	108.73(7)
O(4)-S(1)-C(24)	105.46(8)
O(1)-S(1)-C(24)	105.59(8)
N(1)-S(1)-C(24)	103.59(8)

Symmetry transformations used to generate equivalent atoms:

Table 4. Anisotropic displacement parameters ($Å^2$x 10^3)for **4v**. The anisotropic displacement factor exponent takes the form: $-2\pi^2[h^2a^{*2}U^{11} + ... + 2 h k a^* b^* U^{12}]$

	U^{11}	U^{22}	U^{33}	U^{23}	U^{13}	U^{12}
C(1)	16(1)	18(1)	24(1)	-2(1)	4(1)	-1(1)
C(2)	13(1)	17(1)	29(1)	-2(1)	1(1)	2(1)
C(3)	14(1)	15(1)	28(1)	-1(1)	0(1)	0(1)
C(4)	15(1)	18(1)	22(1)	-1(1)	3(1)	2(1)
C(5)	22(1)	16(1)	25(1)	3(1)	2(1)	-2(1)
C(6)	19(1)	16(1)	20(1)	1(1)	5(1)	2(1)
C(7)	16(1)	19(1)	18(1)	-2(1)	1(1)	3(1)
C(8)	17(1)	19(1)	24(1)	1(1)	-1(1)	3(1)
C(9)	16(1)	19(1)	18(1)	-2(1)	4(1)	0(1)
C(10)	19(1)	15(1)	19(1)	-2(1)	3(1)	1(1)
C(11)	18(1)	22(1)	26(1)	3(1)	-4(1)	0(1)
C(12)	20(1)	30(1)	34(1)	-7(1)	0(1)	10(1)
C(13)	15(1)	26(1)	24(1)	1(1)	6(1)	-2(1)
C(14)	17(1)	19(1)	24(1)	-1(1)	-1(1)	1(1)
C(15)	28(1)	20(1)	29(1)	0(1)	-9(1)	4(1)
C(16)	20(1)	30(1)	36(1)	-4(1)	-4(1)	11(1)
C(17)	19(1)	36(1)	34(1)	-4(1)	9(1)	1(1)
C(18)	35(1)	26(1)	23(1)	4(1)	6(1)	1(1)
C(19)	22(1)	35(1)	33(1)	-4(1)	2(1)	-8(1)
C(20)	29(1)	24(1)	49(1)	13(1)	2(1)	-8(1)
C(21)	24(1)	34(1)	41(1)	11(1)	9(1)	-5(1)
C(22)	22(1)	35(1)	30(1)	-1(1)	-7(1)	4(1)
C(23)	36(1)	19(1)	30(1)	-1(1)	-4(1)	2(1)
C(24)	32(1)	38(1)	42(1)	5(1)	8(1)	-4(1)
C(25)	28(1)	41(1)	39(1)	12(1)	-4(1)	-11(1)
F(1)	59(1)	27(1)	70(1)	1(1)	15(1)	-4(1)
F(2)	32(1)	53(1)	78(1)	4(1)	4(1)	-16(1)
F(3)	66(1)	69(1)	49(1)	21(1)	17(1)	-11(1)
N(1)	17(1)	24(1)	26(1)	-3(1)	0(1)	5(1)
O(1)	22(1)	39(1)	26(1)	1(1)	-2(1)	3(1)
O(2)	24(1)	26(1)	29(1)	-9(1)	-5(1)	7(1)

O(3)	18(1)	25(1)	30(1)	7(1)	-1(1)	3(1)
O(4)	27(1)	40(1)	31(1)	-11(1)	3(1)	6(1)
O(5)	45(1)	37(1)	56(1)	-22(1)	-26(1)	13(1)
S(1)	20(1)	28(1)	26(1)	-2(1)	2(1)	1(1)

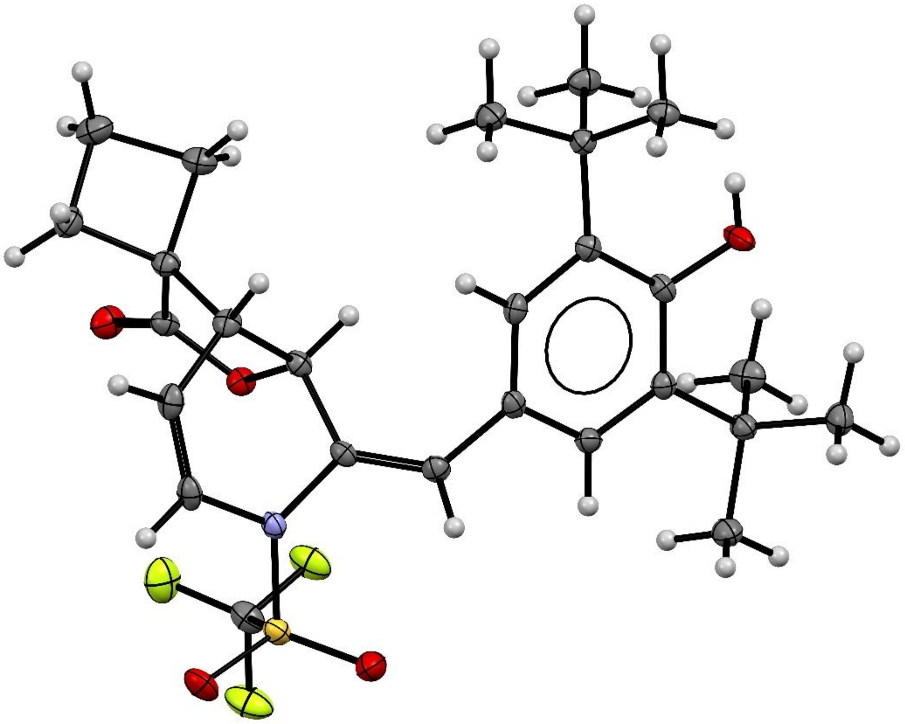

Table 1. Crystal data and structure refinement for **4w**, **CCDC: 2366601.**

Identification code	shelx	
Empirical formula	C26 H32 F3 N O5 S	
Formula weight	527.58	
Temperature	130(2) K	
Wavelength	0.71073 Å	
Crystal system	Monoclinic	
Space group	P 21/c	
Unit cell dimensions	a = 10.5288(10) Å	α= 90°.
	b = 12.175(2) Å	β= 104.149(12)°.
	c = 20.504(4) Å	γ = 90°.
Volume	2548.6(7) $Å^3$	
Z	4	
Density (calculated)	1.375 Mg/m^3	
Absorption coefficient	0.186 mm^{-1}	
F(000)	1112	

Crystal size	0.560 x 0.350 x 0.240 mm^3
Theta range for data collection	3.500 to 29.579°.
Index ranges	-14<=h<=14, -12<=k<=15, -28<=l<=20
Reflections collected	13182
Independent reflections	6017 [R(int) = 0.0276]
Completeness to theta = 25.242°	99.7 %
Refinement method	Full-matrix least-squares on F^2
Data / restraints / parameters	6017 / 0 / 331
Goodness-of-fit on F^2	1.021
Final R indices [I>2sigma(I)]	R1 = 0.0412, wR2 = 0.0925
R indices (all data)	R1 = 0.0580, wR2 = 0.1026
Extinction coefficient	n/a
Largest diff. peak and hole	0.406 and -0.415 e.$Å^{-3}$

Table 2. Atomic coordinates (x 10^4) and equivalent isotropic displacement parameters ($Å^2$x 10^3) for **4w**. U(eq) is defined as one third of the trace of the orthogonalized U^{ij} tensor.

	x	y	z	U(eq)
S(1)	3488(1)	6419(1)	2090(1)	16(1)
F(1)	3454(1)	4288(1)	1896(1)	28(1)
F(2)	2063(1)	5211(1)	1150(1)	33(1)
F(3)	4119(1)	5235(1)	1151(1)	32(1)
O(1)	6314(1)	4361(1)	2314(1)	16(1)
O(2)	3382(1)	7343(1)	1653(1)	23(1)
O(3)	2591(1)	6252(1)	2497(1)	24(1)
O(4)	7448(1)	3941(1)	1552(1)	22(1)
O(5)	5622(1)	1996(1)	5501(1)	23(1)
N(1)	4991(1)	6352(1)	2547(1)	15(1)
C(1)	4799(2)	5345(1)	3547(1)	16(1)
C(2)	5406(2)	5503(1)	3054(1)	14(1)
C(3)	3280(2)	5208(1)	1538(1)	21(1)
C(4)	3056(2)	2335(1)	4759(1)	16(1)
C(5)	4050(2)	3867(1)	4187(1)	15(1)
C(6)	6595(2)	4907(1)	2973(1)	14(1)
C(7)	5523(2)	2814(1)	5037(1)	15(1)
C(8)	7752(2)	5668(1)	2950(1)	16(1)
C(9)	4224(2)	3023(1)	4658(1)	14(1)
C(10)	7253(2)	6688(1)	2556(1)	19(1)
C(11)	6607(2)	3410(1)	4935(1)	14(1)
C(12)	5091(2)	4483(1)	4071(1)	15(1)
C(13)	2883(2)	2466(2)	5482(1)	22(1)
C(14)	8035(2)	3149(1)	5310(1)	15(1)
C(15)	7410(2)	4366(1)	2076(1)	16(1)
C(16)	6349(2)	4249(1)	4454(1)	16(1)
C(17)	1763(2)	2707(2)	4280(1)	22(1)
C(18)	8376(2)	1948(1)	5183(1)	20(1)
C(19)	8510(2)	4942(1)	2569(1)	16(1)
C(20)	9017(2)	3865(2)	5059(1)	23(1)
C(21)	8268(2)	3390(1)	6070(1)	19(1)

C(22)	9618(2)	5365(2)	2258(1)	21(1)
C(23)	3247(2)	1117(1)	4600(1)	23(1)
C(24)	9488(2)	4069(2)	2971(1)	22(1)
C(25)	5999(2)	6952(1)	2345(1)	18(1)
C(26)	10385(2)	4287(2)	2491(1)	26(1)

Table 3. Bond lengths [Å] and angles [°] for **4w**.

S(1)-O(3)	1.4199(13)
S(1)-O(2)	1.4245(12)
S(1)-N(1)	1.6320(14)
S(1)-C(3)	1.8396(18)
F(1)-C(3)	1.3282(19)
F(2)-C(3)	1.3326(19)
F(3)-C(3)	1.323(2)
O(1)-C(15)	1.3571(19)
O(1)-C(6)	1.4696(18)
O(4)-C(15)	1.2034(19)
O(5)-C(7)	1.3635(19)
O(5)-H(5)	0.8200
N(1)-C(25)	1.430(2)
N(1)-C(2)	1.4565(19)
C(1)-C(2)	1.335(2)
C(1)-C(12)	1.480(2)
C(1)-H(1)	0.9500
C(2)-C(6)	1.492(2)
C(4)-C(17)	1.539(2)
C(4)-C(9)	1.542(2)
C(4)-C(23)	1.543(2)
C(4)-C(13)	1.546(2)
C(5)-C(9)	1.391(2)
C(5)-C(12)	1.397(2)
C(5)-H(5A)	0.9500
C(6)-C(8)	1.540(2)
C(6)-H(6)	1.0000
C(7)-C(11)	1.411(2)
C(7)-C(9)	1.422(2)
C(8)-C(10)	1.504(2)
C(8)-C(19)	1.527(2)
C(8)-H(8)	1.0000
C(10)-C(25)	1.325(2)
C(10)-H(10)	0.9500

C(11)-C(16)	1.400(2)
C(11)-C(14)	1.545(2)
C(12)-C(16)	1.394(2)
C(13)-H(13A)	0.9800
C(13)-H(13B)	0.9800
C(13)-H(13C)	0.9800
C(14)-C(20)	1.533(2)
C(14)-C(18)	1.543(2)
C(14)-C(21)	1.545(2)
C(15)-C(19)	1.510(2)
C(16)-H(16)	0.9500
C(17)-H(17A)	0.9800
C(17)-H(17B)	0.9800
C(17)-H(17C)	0.9800
C(18)-H(18A)	0.9800
C(18)-H(18B)	0.9800
C(18)-H(18C)	0.9800
C(19)-C(22)	1.549(2)
C(19)-C(24)	1.568(2)
C(20)-H(20A)	0.9800
C(20)-H(20B)	0.9800
C(20)-H(20C)	0.9800
C(21)-H(21A)	0.9800
C(21)-H(21B)	0.9800
C(21)-H(21C)	0.9800
C(22)-C(26)	1.554(2)
C(22)-H(22A)	0.9900
C(22)-H(22B)	0.9900
C(23)-H(23A)	0.9800
C(23)-H(23B)	0.9800
C(23)-H(23C)	0.9800
C(24)-C(26)	1.546(3)
C(24)-H(24A)	0.9900
C(24)-H(24B)	0.9900
C(25)-H(25)	0.9500
C(26)-H(26A)	0.9900

C(26)-H(26B)	0.9900
O(3)-S(1)-O(2)	121.45(8)
O(3)-S(1)-N(1)	110.31(7)
O(2)-S(1)-N(1)	108.43(7)
O(3)-S(1)-C(3)	104.50(8)
O(2)-S(1)-C(3)	105.44(7)
N(1)-S(1)-C(3)	105.41(7)
C(15)-O(1)-C(6)	109.28(11)
C(7)-O(5)-H(5)	109.7
C(25)-N(1)-C(2)	117.11(12)
C(25)-N(1)-S(1)	119.48(10)
C(2)-N(1)-S(1)	121.65(11)
C(2)-C(1)-C(12)	126.78(15)
C(2)-C(1)-H(1)	116.6
C(12)-C(1)-H(1)	116.6
C(1)-C(2)-N(1)	121.84(14)
C(1)-C(2)-C(6)	125.82(14)
N(1)-C(2)-C(6)	112.25(13)
F(3)-C(3)-F(1)	109.30(14)
F(3)-C(3)-F(2)	109.11(13)
F(1)-C(3)-F(2)	108.38(14)
F(3)-C(3)-S(1)	110.64(12)
F(1)-C(3)-S(1)	110.84(11)
F(2)-C(3)-S(1)	108.53(12)
C(17)-C(4)-C(9)	111.43(13)
C(17)-C(4)-C(23)	106.80(13)
C(9)-C(4)-C(23)	110.17(13)
C(17)-C(4)-C(13)	106.72(13)
C(9)-C(4)-C(13)	110.93(13)
C(23)-C(4)-C(13)	110.68(14)
C(9)-C(5)-C(12)	122.68(14)
C(9)-C(5)-H(5A)	118.7
C(12)-C(5)-H(5A)	118.7
O(1)-C(6)-C(2)	109.69(12)
O(1)-C(6)-C(8)	103.13(12)

C(2)-C(6)-C(8)	113.73(13)
O(1)-C(6)-H(6)	110.0
C(2)-C(6)-H(6)	110.0
C(8)-C(6)-H(6)	110.0
O(5)-C(7)-C(11)	123.56(13)
O(5)-C(7)-C(9)	114.29(14)
C(11)-C(7)-C(9)	122.15(14)
C(10)-C(8)-C(19)	111.08(13)
C(10)-C(8)-C(6)	109.76(13)
C(19)-C(8)-C(6)	100.55(12)
C(10)-C(8)-H(8)	111.7
C(19)-C(8)-H(8)	111.7
C(6)-C(8)-H(8)	111.7
C(5)-C(9)-C(7)	117.21(14)
C(5)-C(9)-C(4)	121.30(13)
C(7)-C(9)-C(4)	121.48(13)
C(25)-C(10)-C(8)	124.44(15)
C(25)-C(10)-H(10)	117.8
C(8)-C(10)-H(10)	117.8
C(16)-C(11)-C(7)	117.10(14)
C(16)-C(11)-C(14)	119.89(14)
C(7)-C(11)-C(14)	122.99(13)
C(16)-C(12)-C(5)	118.12(14)
C(16)-C(12)-C(1)	123.64(15)
C(5)-C(12)-C(1)	118.24(13)
C(4)-C(13)-H(13A)	109.5
C(4)-C(13)-H(13B)	109.5
H(13A)-C(13)-H(13B)	109.5
C(4)-C(13)-H(13C)	109.5
H(13A)-C(13)-H(13C)	109.5
H(13B)-C(13)-H(13C)	109.5
C(20)-C(14)-C(18)	106.16(13)
C(20)-C(14)-C(21)	106.08(13)
C(18)-C(14)-C(21)	111.31(13)
C(20)-C(14)-C(11)	111.71(13)
C(18)-C(14)-C(11)	110.42(13)

C(21)-C(14)-C(11)	110.98(13)
O(4)-C(15)-O(1)	122.37(14)
O(4)-C(15)-C(19)	127.63(15)
O(1)-C(15)-C(19)	109.99(13)
C(12)-C(16)-C(11)	122.70(15)
C(12)-C(16)-H(16)	118.6
C(11)-C(16)-H(16)	118.6
C(4)-C(17)-H(17A)	109.5
C(4)-C(17)-H(17B)	109.5
H(17A)-C(17)-H(17B)	109.5
C(4)-C(17)-H(17C)	109.5
H(17A)-C(17)-H(17C)	109.5
H(17B)-C(17)-H(17C)	109.5
C(14)-C(18)-H(18A)	109.5
C(14)-C(18)-H(18B)	109.5
H(18A)-C(18)-H(18B)	109.5
C(14)-C(18)-H(18C)	109.5
H(18A)-C(18)-H(18C)	109.5
H(18B)-C(18)-H(18C)	109.5
C(15)-C(19)-C(8)	101.56(12)
C(15)-C(19)-C(22)	114.21(13)
C(8)-C(19)-C(22)	124.01(14)
C(15)-C(19)-C(24)	109.51(13)
C(8)-C(19)-C(24)	118.79(13)
C(22)-C(19)-C(24)	88.72(12)
C(14)-C(20)-H(20A)	109.5
C(14)-C(20)-H(20B)	109.5
H(20A)-C(20)-H(20B)	109.5
C(14)-C(20)-H(20C)	109.5
H(20A)-C(20)-H(20C)	109.5
H(20B)-C(20)-H(20C)	109.5
C(14)-C(21)-H(21A)	109.5
C(14)-C(21)-H(21B)	109.5
H(21A)-C(21)-H(21B)	109.5
C(14)-C(21)-H(21C)	109.5
H(21A)-C(21)-H(21C)	109.5

H(21B)-C(21)-H(21C)	109.5
C(19)-C(22)-C(26)	88.86(13)
C(19)-C(22)-H(22A)	113.8
C(26)-C(22)-H(22A)	113.8
C(19)-C(22)-H(22B)	113.8
C(26)-C(22)-H(22B)	113.8
H(22A)-C(22)-H(22B)	111.1
C(4)-C(23)-H(23A)	109.5
C(4)-C(23)-H(23B)	109.5
H(23A)-C(23)-H(23B)	109.5
C(4)-C(23)-H(23C)	109.5
H(23A)-C(23)-H(23C)	109.5
H(23B)-C(23)-H(23C)	109.5
C(26)-C(24)-C(19)	88.47(12)
C(26)-C(24)-H(24A)	113.9
C(19)-C(24)-H(24A)	113.9
C(26)-C(24)-H(24B)	113.9
C(19)-C(24)-H(24B)	113.9
H(24A)-C(24)-H(24B)	111.1
C(10)-C(25)-N(1)	122.11(15)
C(10)-C(25)-H(25)	118.9
N(1)-C(25)-H(25)	118.9
C(24)-C(26)-C(22)	89.36(13)
C(24)-C(26)-H(26A)	113.8
C(22)-C(26)-H(26A)	113.8
C(24)-C(26)-H(26B)	113.8
C(22)-C(26)-H(26B)	113.8
H(26A)-C(26)-H(26B)	111.0

Symmetry transformations used to generate equivalent atoms:

Table 4. Anisotropic displacement parameters ($Å^2$x 10^3)for **4w**. The anisotropic displacement factor exponent takes the form: $-2\pi^2[h^2a^{*2}U^{11} + ... + 2 h k a^* b^* U^{12}]$

	U^{11}	U^{22}	U^{33}	U^{23}	U^{13}	U^{12}
S(1)	19(1)	15(1)	15(1)	2(1)	3(1)	3(1)
F(1)	33(1)	15(1)	30(1)	3(1)	-1(1)	-1(1)
F(2)	31(1)	28(1)	31(1)	-1(1)	-10(1)	-2(1)
F(3)	43(1)	31(1)	24(1)	-8(1)	14(1)	-1(1)
O(1)	16(1)	15(1)	15(1)	-3(1)	3(1)	-1(1)
O(2)	30(1)	17(1)	20(1)	4(1)	2(1)	4(1)
O(3)	19(1)	35(1)	20(1)	2(1)	5(1)	3(1)
O(4)	21(1)	26(1)	19(1)	-8(1)	4(1)	1(1)
O(5)	16(1)	25(1)	25(1)	13(1)	-2(1)	-2(1)
N(1)	18(1)	14(1)	14(1)	3(1)	5(1)	1(1)
C(1)	18(1)	14(1)	16(1)	0(1)	5(1)	2(1)
C(2)	17(1)	12(1)	14(1)	1(1)	2(1)	0(1)
C(3)	24(1)	18(1)	19(1)	1(1)	0(1)	-1(1)
C(4)	15(1)	16(1)	17(1)	3(1)	3(1)	-1(1)
C(5)	14(1)	17(1)	14(1)	1(1)	3(1)	2(1)
C(6)	17(1)	14(1)	11(1)	-1(1)	2(1)	0(1)
C(7)	18(1)	13(1)	13(1)	1(1)	3(1)	0(1)
C(8)	15(1)	17(1)	15(1)	-3(1)	3(1)	-3(1)
C(9)	16(1)	14(1)	13(1)	-1(1)	4(1)	0(1)
C(10)	24(1)	14(1)	21(1)	-2(1)	10(1)	-4(1)
C(11)	16(1)	14(1)	13(1)	-2(1)	3(1)	0(1)
C(12)	19(1)	13(1)	13(1)	0(1)	6(1)	2(1)
C(13)	22(1)	23(1)	21(1)	4(1)	7(1)	-3(1)
C(14)	14(1)	17(1)	14(1)	1(1)	3(1)	0(1)
C(15)	16(1)	15(1)	16(1)	0(1)	2(1)	3(1)
C(16)	18(1)	16(1)	15(1)	-2(1)	7(1)	-2(1)
C(17)	16(1)	25(1)	23(1)	4(1)	3(1)	-1(1)
C(18)	20(1)	20(1)	21(1)	-3(1)	4(1)	3(1)
C(19)	14(1)	17(1)	15(1)	-1(1)	2(1)	-1(1)
C(20)	16(1)	26(1)	26(1)	5(1)	4(1)	-1(1)
C(21)	18(1)	19(1)	18(1)	-2(1)	1(1)	0(1)

C(22)	18(1)	25(1)	21(1)	0(1)	7(1)	-2(1)
C(23)	23(1)	17(1)	28(1)	0(1)	3(1)	-3(1)
C(24)	18(1)	23(1)	21(1)	1(1)	0(1)	2(1)
C(25)	26(1)	12(1)	18(1)	2(1)	10(1)	-1(1)
C(26)	17(1)	30(1)	32(1)	0(1)	6(1)	3(1)

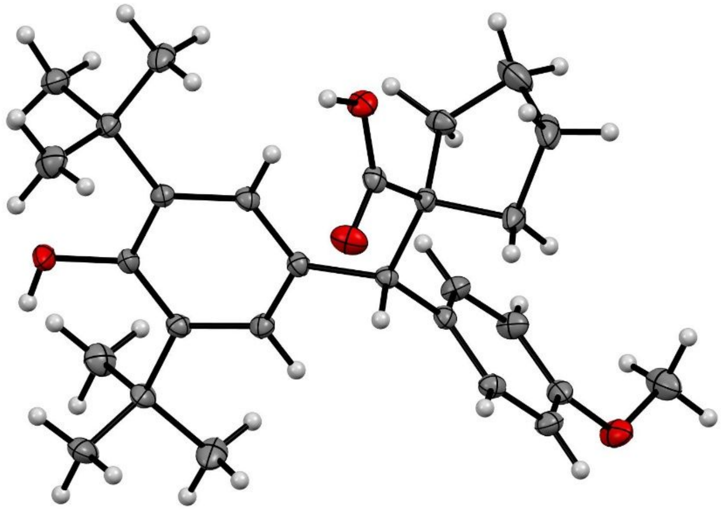

Table 1. Crystal data and structure refinement for pqm-ac.

Identification code	shelx	
Empirical formula	C28 H38 O4	
Formula weight	438.58	
Temperature	130(2) K	
Wavelength	0.71073 Å	
Crystal system	Trigonal	
Space group	P -3	
Unit cell dimensions	a = 20.4182(8) Å	α= 90°.
	b = 20.4182(8) Å	β= 90°.
	c = 20.4703(8) Å	γ = 120°.
Volume	7390.8(6) $Å^3$	
Z	12	
Density (calculated)	1.182 Mg/m^3	
Absorption coefficient	0.077 mm^{-1}	
F(000)	2856	
Crystal size	0.460 x 0.210 x 0.180 mm^3	
Theta range for data collection	3.456 to 29.595°.	

Index ranges	-28<=h<=27, -27<=k<=26, -24<=l<=27
Reflections collected	85169
Independent reflections	12758 [R(int) = 0.0405]
Completeness to theta = 25.242°	99.7 %
Refinement method	Full-matrix least-squares on F^2
Data / restraints / parameters	12758 / 0 / 591
Goodness-of-fit on F^2	1.023
Final R indices [I>2sigma(I)]	R1 = 0.0555, wR2 = 0.1373
R indices (all data)	R1 = 0.0802, wR2 = 0.1572
Extinction coefficient	n/a
Largest diff. peak and hole	0.990 and -0.462 e.Å^{-3}

Table 2. Atomic coordinates (x 10^4) and equivalent isotropic displacement parameters ($Å^2$x 10^3) for pqm-ac. U(eq) is defined as one third of the trace of the orthogonalized U^{ij} tensor.

	x	y	z	U(eq)
O(1)	4921(1)	5205(1)	3294(1)	22(1)
O(3)	3705(1)	1536(1)	3943(1)	24(1)
O(4)	11(1)	3316(1)	3910(1)	28(1)
O(5)	4182(1)	4486(1)	2480(1)	26(1)
C(1)	4062(1)	2823(1)	3875(1)	16(1)
C(3)	4275(1)	4777(1)	3045(1)	17(1)
C(4)	3863(1)	3373(1)	3751(1)	16(1)
C(5)	2161(1)	3638(1)	3506(1)	16(1)
C(6)	3482(1)	2062(1)	3845(1)	16(1)
C(10)	2575(1)	2440(1)	3589(1)	17(1)
C(11)	3134(1)	3202(1)	3583(1)	16(1)
C(12)	2942(1)	3786(1)	3315(1)	14(1)
C(13)	2722(1)	1858(1)	3729(1)	16(1)
C(16)	1890(1)	3505(1)	4145(1)	20(1)
C(19)	3571(1)	4631(1)	3419(1)	16(1)
C(21)	4898(1)	3054(1)	3989(1)	18(1)
C(22)	1681(1)	3638(1)	3019(1)	18(1)
C(24)	728(1)	3427(1)	3804(1)	20(1)
C(25)	2075(1)	1030(1)	3727(1)	20(1)
C(32)	3760(1)	4914(1)	4128(1)	22(1)
C(33)	1181(1)	3403(1)	4298(1)	22(1)
C(34)	974(1)	3536(1)	3161(1)	21(1)
C(39)	5009(1)	2701(1)	4614(1)	24(1)
C(42)	2167(1)	600(1)	3146(1)	27(1)
C(43)	3355(1)	5194(1)	3115(1)	25(1)
C(44)	2040(1)	639(1)	4383(1)	27(1)
C(46)	5404(1)	3915(1)	4059(1)	27(1)
C(48)	5186(1)	2811(1)	3395(1)	28(1)
C(49)	1299(1)	971(1)	3643(1)	31(1)
C(51)	-100(1)	3623(1)	4491(1)	34(1)
C(54)	3938(1)	5959(1)	3404(1)	33(1)

C(56)	4043(1)	5770(1)	4110(1)	38(1)
O(2)	1578(1)	-2051(1)	1086(1)	24(1)
O(6)	4521(1)	619(1)	2594(1)	28(1)
O(7)	5279(1)	613(1)	1809(1)	31(1)
O(8)	3188(1)	3293(1)	696(1)	31(1)
C(2)	1838(1)	-776(1)	1345(1)	16(1)
C(7)	2395(1)	-36(1)	1490(1)	17(1)
C(8)	2075(1)	-1306(1)	1227(1)	16(1)
C(9)	3546(1)	1591(1)	1456(1)	16(1)
C(14)	3161(1)	195(1)	1517(1)	16(1)
C(15)	4794(1)	777(1)	2022(1)	19(1)
C(17)	3313(1)	1582(1)	812(1)	19(1)
C(18)	3364(1)	-349(1)	1395(1)	17(1)
C(20)	2843(1)	-1103(1)	1253(1)	16(1)
C(23)	3731(1)	1005(1)	1723(1)	16(1)
C(26)	4567(1)	1224(1)	1580(1)	17(1)
C(27)	3633(1)	2184(1)	1857(1)	21(1)
C(28)	1000(1)	-991(1)	1331(1)	20(1)
C(29)	3327(1)	2751(1)	973(1)	22(1)
C(30)	3202(1)	2155(1)	571(1)	20(1)
C(31)	3112(1)	-1674(1)	1125(1)	20(1)
C(35)	3536(1)	2768(1)	1617(1)	24(1)
C(36)	644(1)	-1278(1)	652(1)	25(1)
C(37)	557(1)	-1580(1)	1866(1)	26(1)
C(38)	4724(1)	1149(1)	847(1)	22(1)
C(40)	5146(1)	2072(1)	1749(1)	24(1)
C(41)	2758(1)	-2327(1)	1618(1)	27(1)
C(45)	902(1)	-306(1)	1477(1)	31(1)
C(47)	3973(1)	-1310(1)	1197(1)	30(1)
C(50)	2912(1)	-1980(1)	426(1)	29(1)
C(52)	5451(1)	1872(1)	671(1)	32(1)
C(53)	3695(1)	4054(1)	874(1)	32(1)
C(55)	5409(1)	2467(1)	1089(1)	32(1)

Table 3. Bond lengths [Å] and angles [°] for pqm-ac.

O(1)-C(3)	1.2704(18)
O(1)-H(1)	0.9660
O(3)-C(6)	1.3745(17)
O(3)-H(3)	0.8201
O(4)-C(24)	1.3818(18)
O(4)-C(51)	1.415(2)
O(5)-C(3)	1.2705(18)
C(1)-C(4)	1.395(2)
C(1)-C(6)	1.407(2)
C(1)-C(21)	1.545(2)
C(3)-C(19)	1.519(2)
C(4)-C(11)	1.392(2)
C(4)-H(4)	0.9500
C(5)-C(16)	1.395(2)
C(5)-C(22)	1.398(2)
C(5)-C(12)	1.5173(19)
C(6)-C(13)	1.412(2)
C(10)-C(13)	1.393(2)
C(10)-C(11)	1.395(2)
C(10)-H(10)	0.9500
C(11)-C(12)	1.5328(19)
C(12)-C(19)	1.5664(19)
C(12)-H(12)	1.0000
C(13)-C(25)	1.541(2)
C(16)-C(33)	1.391(2)
C(16)-H(16)	0.9500
C(19)-C(32)	1.539(2)
C(19)-C(43)	1.554(2)
C(21)-C(46)	1.536(2)
C(21)-C(48)	1.538(2)
C(21)-C(39)	1.540(2)
C(22)-C(34)	1.382(2)
C(22)-H(22)	0.9500
C(24)-C(34)	1.386(2)

C(24)-C(33)	1.388(2)
C(25)-C(49)	1.538(2)
C(25)-C(42)	1.543(2)
C(25)-C(44)	1.545(2)
C(32)-C(56)	1.543(2)
C(32)-H(32A)	0.9900
C(32)-H(32B)	0.9900
C(33)-H(33)	0.9500
C(34)-H(34)	0.9500
C(39)-H(39A)	0.9800
C(39)-H(39B)	0.9800
C(39)-H(39C)	0.9800
C(42)-H(42A)	0.9800
C(42)-H(42B)	0.9800
C(42)-H(42C)	0.9800
C(43)-C(54)	1.532(2)
C(43)-H(43A)	0.9900
C(43)-H(43B)	0.9900
C(44)-H(44A)	0.9800
C(44)-H(44B)	0.9800
C(44)-H(44C)	0.9800
C(46)-H(46A)	0.9800
C(46)-H(46B)	0.9800
C(46)-H(46C)	0.9800
C(48)-H(48A)	0.9800
C(48)-H(48B)	0.9800
C(48)-H(48C)	0.9800
C(49)-H(49A)	0.9800
C(49)-H(49B)	0.9800
C(49)-H(49C)	0.9800
C(51)-H(51A)	0.9800
C(51)-H(51B)	0.9800
C(51)-H(51C)	0.9800
C(54)-C(56)	1.539(3)
C(54)-H(54A)	0.9900
C(54)-H(54B)	0.9900

C(56)-H(56A)	0.9900
C(56)-H(56B)	0.9900
O(2)-C(8)	1.3740(17)
O(2)-H(2)	0.8200
O(6)-C(15)	1.2676(19)
O(7)-C(15)	1.2708(19)
O(7)-H(7)	1.1275
O(8)-C(29)	1.3911(19)
O(8)-C(53)	1.418(2)
C(2)-C(7)	1.395(2)
C(2)-C(8)	1.411(2)
C(2)-C(28)	1.539(2)
C(7)-C(14)	1.392(2)
C(7)-H(7A)	0.9500
C(8)-C(20)	1.407(2)
C(9)-C(17)	1.398(2)
C(9)-C(27)	1.399(2)
C(9)-C(23)	1.523(2)
C(14)-C(18)	1.388(2)
C(14)-C(23)	1.531(2)
C(15)-C(26)	1.514(2)
C(17)-C(30)	1.388(2)
C(17)-H(17)	0.9500
C(18)-C(20)	1.395(2)
C(18)-H(18)	0.9500
C(20)-C(31)	1.541(2)
C(23)-C(26)	1.562(2)
C(23)-H(23)	1.0000
C(26)-C(38)	1.557(2)
C(26)-C(40)	1.571(2)
C(27)-C(35)	1.392(2)
C(27)-H(27)	0.9500
C(28)-C(45)	1.538(2)
C(28)-C(37)	1.540(2)
C(28)-C(36)	1.542(2)
C(29)-C(35)	1.381(2)

C(29)-C(30)	1.384(2)
C(30)-H(30)	0.9500
C(31)-C(50)	1.533(2)
C(31)-C(41)	1.534(2)
C(31)-C(47)	1.536(2)
C(35)-H(35)	0.9500
C(36)-H(36A)	0.9800
C(36)-H(36B)	0.9800
C(36)-H(36C)	0.9800
C(37)-H(37A)	0.9800
C(37)-H(37B)	0.9800
C(37)-H(37C)	0.9800
C(38)-C(52)	1.523(2)
C(38)-H(38A)	0.9900
C(38)-H(38B)	0.9900
C(40)-C(55)	1.527(2)
C(40)-H(40A)	0.9900
C(40)-H(40B)	0.9900
C(41)-H(41A)	0.9800
C(41)-H(41B)	0.9800
C(41)-H(41C)	0.9800
C(45)-H(45A)	0.9800
C(45)-H(45B)	0.9800
C(45)-H(45C)	0.9800
C(47)-H(47A)	0.9800
C(47)-H(47B)	0.9800
C(47)-H(47C)	0.9800
C(50)-H(50A)	0.9800
C(50)-H(50B)	0.9800
C(50)-H(50C)	0.9800
C(52)-C(55)	1.523(3)
C(52)-H(52A)	0.9900
C(52)-H(52B)	0.9900
C(53)-H(53A)	0.9800
C(53)-H(53B)	0.9800
C(53)-H(53C)	0.9800

C(55)-H(55A)	0.9900
C(55)-H(55B)	0.9900

C(3)-O(1)-H(1)	111.6
C(6)-O(3)-H(3)	109.5
C(24)-O(4)-C(51)	116.76(13)
C(4)-C(1)-C(6)	117.30(13)
C(4)-C(1)-C(21)	120.19(13)
C(6)-C(1)-C(21)	122.36(13)
O(1)-C(3)-O(5)	123.17(14)
O(1)-C(3)-C(19)	119.25(13)
O(5)-C(3)-C(19)	117.51(13)
C(11)-C(4)-C(1)	123.21(13)
C(11)-C(4)-H(4)	118.4
C(1)-C(4)-H(4)	118.4
C(16)-C(5)-C(22)	117.33(13)
C(16)-C(5)-C(12)	123.69(13)
C(22)-C(5)-C(12)	118.97(13)
O(3)-C(6)-C(1)	115.60(13)
O(3)-C(6)-C(13)	122.63(13)
C(1)-C(6)-C(13)	121.77(13)
C(13)-C(10)-C(11)	123.17(13)
C(13)-C(10)-H(10)	118.4
C(11)-C(10)-H(10)	118.4
C(4)-C(11)-C(10)	117.05(13)
C(4)-C(11)-C(12)	123.44(13)
C(10)-C(11)-C(12)	119.04(13)
C(5)-C(12)-C(11)	113.87(12)
C(5)-C(12)-C(19)	112.80(11)
C(11)-C(12)-C(19)	114.86(11)
C(5)-C(12)-H(12)	104.6
C(11)-C(12)-H(12)	104.6
C(19)-C(12)-H(12)	104.6
C(10)-C(13)-C(6)	117.21(13)
C(10)-C(13)-C(25)	120.29(13)
C(6)-C(13)-C(25)	122.46(13)

C(33)-C(16)-C(5)	121.45(14)
C(33)-C(16)-H(16)	119.3
C(5)-C(16)-H(16)	119.3
C(3)-C(19)-C(32)	111.53(12)
C(3)-C(19)-C(43)	104.13(12)
C(32)-C(19)-C(43)	102.15(12)
C(3)-C(19)-C(12)	108.82(11)
C(32)-C(19)-C(12)	117.03(12)
C(43)-C(19)-C(12)	112.34(12)
C(46)-C(21)-C(48)	107.25(13)
C(46)-C(21)-C(39)	106.63(13)
C(48)-C(21)-C(39)	109.59(13)
C(46)-C(21)-C(1)	111.85(12)
C(48)-C(21)-C(1)	108.94(12)
C(39)-C(21)-C(1)	112.42(12)
C(34)-C(22)-C(5)	121.99(14)
C(34)-C(22)-H(22)	119.0
C(5)-C(22)-H(22)	119.0
O(4)-C(24)-C(34)	116.29(14)
O(4)-C(24)-C(33)	123.41(14)
C(34)-C(24)-C(33)	120.25(14)
C(49)-C(25)-C(13)	111.70(13)
C(49)-C(25)-C(42)	106.49(13)
C(13)-C(25)-C(42)	110.18(13)
C(49)-C(25)-C(44)	106.06(14)
C(13)-C(25)-C(44)	110.93(12)
C(42)-C(25)-C(44)	111.33(13)
C(19)-C(32)-C(56)	106.53(14)
C(19)-C(32)-H(32A)	110.4
C(56)-C(32)-H(32A)	110.4
C(19)-C(32)-H(32B)	110.4
C(56)-C(32)-H(32B)	110.4
H(32A)-C(32)-H(32B)	108.6
C(24)-C(33)-C(16)	119.55(14)
C(24)-C(33)-H(33)	120.2
C(16)-C(33)-H(33)	120.2

C(22)-C(34)-C(24)	119.38(14)
C(22)-C(34)-H(34)	120.3
C(24)-C(34)-H(34)	120.3
C(21)-C(39)-H(39A)	109.5
C(21)-C(39)-H(39B)	109.5
H(39A)-C(39)-H(39B)	109.5
C(21)-C(39)-H(39C)	109.5
H(39A)-C(39)-H(39C)	109.5
H(39B)-C(39)-H(39C)	109.5
C(25)-C(42)-H(42A)	109.5
C(25)-C(42)-H(42B)	109.5
H(42A)-C(42)-H(42B)	109.5
C(25)-C(42)-H(42C)	109.5
H(42A)-C(42)-H(42C)	109.5
H(42B)-C(42)-H(42C)	109.5
C(54)-C(43)-C(19)	103.60(13)
C(54)-C(43)-H(43A)	111.0
C(19)-C(43)-H(43A)	111.0
C(54)-C(43)-H(43B)	111.0
C(19)-C(43)-H(43B)	111.0
H(43A)-C(43)-H(43B)	109.0
C(25)-C(44)-H(44A)	109.5
C(25)-C(44)-H(44B)	109.5
H(44A)-C(44)-H(44B)	109.5
C(25)-C(44)-H(44C)	109.5
H(44A)-C(44)-H(44C)	109.5
H(44B)-C(44)-H(44C)	109.5
C(21)-C(46)-H(46A)	109.5
C(21)-C(46)-H(46B)	109.5
H(46A)-C(46)-H(46B)	109.5
C(21)-C(46)-H(46C)	109.5
H(46A)-C(46)-H(46C)	109.5
H(46B)-C(46)-H(46C)	109.5
C(21)-C(48)-H(48A)	109.5
C(21)-C(48)-H(48B)	109.5
H(48A)-C(48)-H(48B)	109.5

C(21)-C(48)-H(48C)	109.5
H(48A)-C(48)-H(48C)	109.5
H(48B)-C(48)-H(48C)	109.5
C(25)-C(49)-H(49A)	109.5
C(25)-C(49)-H(49B)	109.5
H(49A)-C(49)-H(49B)	109.5
C(25)-C(49)-H(49C)	109.5
H(49A)-C(49)-H(49C)	109.5
H(49B)-C(49)-H(49C)	109.5
O(4)-C(51)-H(51A)	109.5
O(4)-C(51)-H(51B)	109.5
H(51A)-C(51)-H(51B)	109.5
O(4)-C(51)-H(51C)	109.5
H(51A)-C(51)-H(51C)	109.5
H(51B)-C(51)-H(51C)	109.5
C(43)-C(54)-C(56)	104.07(14)
C(43)-C(54)-H(54A)	110.9
C(56)-C(54)-H(54A)	110.9
C(43)-C(54)-H(54B)	110.9
C(56)-C(54)-H(54B)	110.9
H(54A)-C(54)-H(54B)	109.0
C(54)-C(56)-C(32)	106.59(14)
C(54)-C(56)-H(56A)	110.4
C(32)-C(56)-H(56A)	110.4
C(54)-C(56)-H(56B)	110.4
C(32)-C(56)-H(56B)	110.4
H(56A)-C(56)-H(56B)	108.6
C(8)-O(2)-H(2)	109.5
C(15)-O(7)-H(7)	111.6
C(29)-O(8)-C(53)	116.02(13)
C(7)-C(2)-C(8)	117.39(13)
C(7)-C(2)-C(28)	120.12(13)
C(8)-C(2)-C(28)	122.48(13)
C(14)-C(7)-C(2)	123.02(13)
C(14)-C(7)-H(7A)	118.5
C(2)-C(7)-H(7A)	118.5

O(2)-C(8)-C(20)	115.55(12)
O(2)-C(8)-C(2)	122.64(13)
C(20)-C(8)-C(2)	121.81(13)
C(17)-C(9)-C(27)	117.28(14)
C(17)-C(9)-C(23)	122.99(13)
C(27)-C(9)-C(23)	119.69(13)
C(18)-C(14)-C(7)	117.18(13)
C(18)-C(14)-C(23)	123.05(13)
C(7)-C(14)-C(23)	119.63(13)
O(6)-C(15)-O(7)	123.44(14)
O(6)-C(15)-C(26)	118.60(13)
O(7)-C(15)-C(26)	117.86(13)
C(30)-C(17)-C(9)	121.52(14)
C(30)-C(17)-H(17)	119.2
C(9)-C(17)-H(17)	119.2
C(14)-C(18)-C(20)	123.50(13)
C(14)-C(18)-H(18)	118.3
C(20)-C(18)-H(18)	118.3
C(18)-C(20)-C(8)	117.11(13)
C(18)-C(20)-C(31)	120.41(13)
C(8)-C(20)-C(31)	122.47(13)
C(9)-C(23)-C(14)	113.39(12)
C(9)-C(23)-C(26)	111.85(11)
C(14)-C(23)-C(26)	112.87(12)
C(9)-C(23)-H(23)	106.0
C(14)-C(23)-H(23)	106.0
C(26)-C(23)-H(23)	106.0
C(15)-C(26)-C(38)	112.13(12)
C(15)-C(26)-C(23)	109.58(12)
C(38)-C(26)-C(23)	113.73(12)
C(15)-C(26)-C(40)	104.16(12)
C(38)-C(26)-C(40)	103.99(12)
C(23)-C(26)-C(40)	112.78(12)
C(35)-C(27)-C(9)	121.54(15)
C(35)-C(27)-H(27)	119.2
C(9)-C(27)-H(27)	119.2

C(45)-C(28)-C(2)	111.56(12)
C(45)-C(28)-C(37)	106.23(13)
C(2)-C(28)-C(37)	110.15(12)
C(45)-C(28)-C(36)	105.65(13)
C(2)-C(28)-C(36)	111.70(13)
C(37)-C(28)-C(36)	111.34(13)
C(35)-C(29)-C(30)	120.43(15)
C(35)-C(29)-O(8)	123.59(14)
C(30)-C(29)-O(8)	115.89(15)
C(29)-C(30)-C(17)	119.64(14)
C(29)-C(30)-H(30)	120.2
C(17)-C(30)-H(30)	120.2
C(50)-C(31)-C(41)	110.26(14)
C(50)-C(31)-C(47)	107.11(14)
C(41)-C(31)-C(47)	106.51(13)
C(50)-C(31)-C(20)	110.14(13)
C(41)-C(31)-C(20)	110.80(13)
C(47)-C(31)-C(20)	111.91(13)
C(29)-C(35)-C(27)	119.47(14)
C(29)-C(35)-H(35)	120.3
C(27)-C(35)-H(35)	120.3
C(28)-C(36)-H(36A)	109.5
C(28)-C(36)-H(36B)	109.5
H(36A)-C(36)-H(36B)	109.5
C(28)-C(36)-H(36C)	109.5
H(36A)-C(36)-H(36C)	109.5
H(36B)-C(36)-H(36C)	109.5
C(28)-C(37)-H(37A)	109.5
C(28)-C(37)-H(37B)	109.5
H(37A)-C(37)-H(37B)	109.5
C(28)-C(37)-H(37C)	109.5
H(37A)-C(37)-H(37C)	109.5
H(37B)-C(37)-H(37C)	109.5
C(52)-C(38)-C(26)	106.36(13)
C(52)-C(38)-H(38A)	110.5
C(26)-C(38)-H(38A)	110.5

C(52)-C(38)-H(38B)	110.5
C(26)-C(38)-H(38B)	110.5
H(38A)-C(38)-H(38B)	108.6
C(55)-C(40)-C(26)	105.03(13)
C(55)-C(40)-H(40A)	110.7
C(26)-C(40)-H(40A)	110.7
C(55)-C(40)-H(40B)	110.7
C(26)-C(40)-H(40B)	110.7
H(40A)-C(40)-H(40B)	108.8
C(31)-C(41)-H(41A)	109.5
C(31)-C(41)-H(41B)	109.5
H(41A)-C(41)-H(41B)	109.5
C(31)-C(41)-H(41C)	109.5
H(41A)-C(41)-H(41C)	109.5
H(41B)-C(41)-H(41C)	109.5
C(28)-C(45)-H(45A)	109.5
C(28)-C(45)-H(45B)	109.5
H(45A)-C(45)-H(45B)	109.5
C(28)-C(45)-H(45C)	109.5
H(45A)-C(45)-H(45C)	109.5
H(45B)-C(45)-H(45C)	109.5
C(31)-C(47)-H(47A)	109.5
C(31)-C(47)-H(47B)	109.5
H(47A)-C(47)-H(47B)	109.5
C(31)-C(47)-H(47C)	109.5
H(47A)-C(47)-H(47C)	109.5
H(47B)-C(47)-H(47C)	109.5
C(31)-C(50)-H(50A)	109.5
C(31)-C(50)-H(50B)	109.5
H(50A)-C(50)-H(50B)	109.5
C(31)-C(50)-H(50C)	109.5
H(50A)-C(50)-H(50C)	109.5
H(50B)-C(50)-H(50C)	109.5
C(55)-C(52)-C(38)	102.96(13)
C(55)-C(52)-H(52A)	111.2
C(38)-C(52)-H(52A)	111.2

C(55)-C(52)-H(52B)	111.2
C(38)-C(52)-H(52B)	111.2
H(52A)-C(52)-H(52B)	109.1
O(8)-C(53)-H(53A)	109.5
O(8)-C(53)-H(53B)	109.5
H(53A)-C(53)-H(53B)	109.5
O(8)-C(53)-H(53C)	109.5
H(53A)-C(53)-H(53C)	109.5
H(53B)-C(53)-H(53C)	109.5
C(52)-C(55)-C(40)	102.73(14)
C(52)-C(55)-H(55A)	111.2
C(40)-C(55)-H(55A)	111.2
C(52)-C(55)-H(55B)	111.2
C(40)-C(55)-H(55B)	111.2
H(55A)-C(55)-H(55B)	109.1

Symmetry transformations used to generate equivalent atoms:

Table 4. Anisotropic displacement parameters ($Å^2 x 10^3$)for pqm-ac. The anisotropic displacement factor exponent takes the form: $-2\pi^2[h^2a^{*2}U^{11} + ... + 2 h k a^* b^* U^{12}]$

	U^{11}	U^{22}	U^{33}	U^{23}	U^{13}	U^{12}
O(1)	14(1)	21(1)	25(1)	0(1)	2(1)	6(1)
O(3)	20(1)	12(1)	41(1)	-3(1)	-8(1)	8(1)
O(4)	16(1)	37(1)	31(1)	-9(1)	-1(1)	13(1)
O(5)	24(1)	27(1)	20(1)	-3(1)	5(1)	7(1)
C(1)	14(1)	15(1)	17(1)	-1(1)	0(1)	7(1)
C(3)	17(1)	12(1)	20(1)	3(1)	2(1)	6(1)
C(4)	14(1)	12(1)	19(1)	1(1)	1(1)	5(1)
C(5)	14(1)	12(1)	21(1)	0(1)	0(1)	6(1)
C(6)	19(1)	13(1)	18(1)	-1(1)	-2(1)	9(1)
C(10)	14(1)	15(1)	21(1)	1(1)	0(1)	7(1)
C(11)	15(1)	14(1)	17(1)	0(1)	1(1)	7(1)
C(12)	13(1)	12(1)	16(1)	0(1)	0(1)	6(1)
C(13)	16(1)	13(1)	18(1)	0(1)	-1(1)	5(1)
C(16)	16(1)	23(1)	19(1)	0(1)	-2(1)	9(1)
C(19)	14(1)	13(1)	20(1)	1(1)	0(1)	7(1)
C(21)	15(1)	17(1)	24(1)	0(1)	-1(1)	8(1)
C(22)	18(1)	16(1)	18(1)	0(1)	-1(1)	6(1)
C(24)	13(1)	19(1)	27(1)	-4(1)	0(1)	7(1)
C(25)	17(1)	12(1)	28(1)	0(1)	-3(1)	5(1)
C(32)	18(1)	23(1)	21(1)	-6(1)	1(1)	8(1)
C(33)	18(1)	26(1)	19(1)	-1(1)	2(1)	9(1)
C(34)	17(1)	21(1)	22(1)	-1(1)	-4(1)	8(1)
C(39)	22(1)	21(1)	28(1)	-1(1)	-7(1)	10(1)
C(42)	27(1)	17(1)	31(1)	-5(1)	-8(1)	7(1)
C(43)	20(1)	14(1)	41(1)	3(1)	-4(1)	9(1)
C(44)	28(1)	19(1)	29(1)	6(1)	2(1)	8(1)
C(46)	15(1)	19(1)	44(1)	1(1)	-5(1)	7(1)
C(48)	23(1)	35(1)	29(1)	-2(1)	2(1)	17(1)
C(49)	16(1)	17(1)	52(1)	6(1)	-2(1)	3(1)
C(51)	21(1)	35(1)	42(1)	-15(1)	4(1)	11(1)
C(54)	22(1)	16(1)	60(1)	-1(1)	-2(1)	10(1)

C(56)	38(1)	26(1)	45(1)	-16(1)	0(1)	13(1)
O(2)	13(1)	14(1)	41(1)	-5(1)	-2(1)	6(1)
O(6)	32(1)	31(1)	22(1)	4(1)	-2(1)	18(1)
O(7)	29(1)	41(1)	35(1)	3(1)	-3(1)	26(1)
O(8)	29(1)	18(1)	48(1)	-3(1)	-12(1)	14(1)
C(2)	12(1)	16(1)	19(1)	1(1)	0(1)	6(1)
C(7)	16(1)	16(1)	20(1)	1(1)	0(1)	9(1)
C(8)	14(1)	13(1)	19(1)	0(1)	0(1)	5(1)
C(9)	11(1)	14(1)	22(1)	0(1)	1(1)	5(1)
C(14)	15(1)	14(1)	17(1)	1(1)	0(1)	6(1)
C(15)	15(1)	18(1)	20(1)	0(1)	-3(1)	7(1)
C(17)	18(1)	18(1)	21(1)	-2(1)	1(1)	10(1)
C(18)	13(1)	16(1)	22(1)	2(1)	-1(1)	7(1)
C(20)	15(1)	16(1)	19(1)	2(1)	0(1)	8(1)
C(23)	15(1)	14(1)	18(1)	0(1)	0(1)	7(1)
C(26)	14(1)	14(1)	21(1)	-1(1)	-2(1)	6(1)
C(27)	17(1)	22(1)	24(1)	-5(1)	-2(1)	9(1)
C(28)	13(1)	17(1)	29(1)	-3(1)	-2(1)	8(1)
C(29)	14(1)	16(1)	36(1)	1(1)	0(1)	8(1)
C(30)	18(1)	22(1)	23(1)	2(1)	1(1)	11(1)
C(31)	16(1)	17(1)	28(1)	0(1)	-1(1)	10(1)
C(35)	19(1)	18(1)	37(1)	-8(1)	-2(1)	10(1)
C(36)	18(1)	25(1)	32(1)	-2(1)	-6(1)	10(1)
C(37)	18(1)	28(1)	28(1)	-1(1)	5(1)	9(1)
C(38)	19(1)	25(1)	22(1)	5(1)	3(1)	12(1)
C(40)	15(1)	18(1)	37(1)	-4(1)	-7(1)	6(1)
C(41)	30(1)	20(1)	35(1)	4(1)	2(1)	15(1)
C(45)	17(1)	23(1)	55(1)	-9(1)	-5(1)	12(1)
C(47)	18(1)	21(1)	53(1)	-2(1)	-1(1)	12(1)
C(50)	30(1)	37(1)	30(1)	-7(1)	0(1)	23(1)
C(52)	24(1)	30(1)	41(1)	12(1)	13(1)	14(1)
C(53)	27(1)	20(1)	46(1)	4(1)	-8(1)	9(1)
C(55)	20(1)	21(1)	52(1)	9(1)	6(1)	8(1)

Computational details.

Computational details

All electronic structure calculations were performed using the ORCA program package, version 5.0.3.[2,3] We employed the ωB97X exchange-correlation functional[4] in combination with the D3 dispersion correction.[5] This combination is particularly well-suited to describe long-range dispersion interactions, a critical aspect in the accurate modeling of the systems studied here.[6] The calculations were conducted using the Def2-TZVP[7] basis set and the corresponding auxiliary counterparts,[8] which provides a good balance between computational efficiency and accuracy. All calculations used the resolution of the identity approximation for the formation of the Coulomb matrix[9] together with an improved chain of spheres algorithm to calculate the exchange contribution.[10–12] For all geometry optimizations, tight convergence criteria in ORCA were applied, and vibrational frequency analyses were performed to confirm the nature of the stationary points as minima or transition states on the corresponding potential energy surface. Solvent effects were modeled using the SMD solvation model,[13] which approximates the electrostatic influence of the solvent environment on the electronic structure of the molecules. We consider zero-point energy corrections, and we report Gibbs free energies at 298 K. Finally, the analysis of the electron density of selected molecules was carried out using the AIMAll package.[14]

Coordinates

1s

O	2.83588745	8.8103686	-3.06900998
O	4.50863818	9.74934951	-1.89578608
O	7.33588046	6.20253529	5.8154789
F	0.1611207	3.91909708	2.25669782
C	6.13939557	6.13377993	0.49832083
H	6.94509816	6.46167312	-0.16983581
C	3.48544038	9.04066799	-2.02342713
C	2.9621536	8.36203099	-0.75636982
C	3.49509905	5.61015742	-2.16970151
H	4.50422577	4.07134129	1.22588178
C	2.71926231	4.64630711	-1.61543976
H	3.23124126	5.97084653	-3.16598441
N	3.07118706	4.13045889	-0.32480741
C	4.2144187	4.62668909	0.33570398
F	-0.01100595	4.92336282	0.35247321
C	4.93490357	5.65328326	-0.18220701
C	4.54522291	6.24441463	-1.45623931
H	5.22915128	6.92855581	-1.95880844
C	1.62299702	7.75519735	-0.77872815
H	1.69109254	6.65024645	-0.96612546
H	1.08444527	7.87442926	0.17415385

H	1.03764493	8.14622278	-1.62267477
C	3.61373007	8.63258869	0.55088995
H	3.58311322	7.75003133	1.21405341
H	3.07527941	9.43997723	1.08545125
H	4.64991611	8.96160245	0.40281874
C	6.36604729	6.24824571	1.83841596
C	5.3613842	5.99097288	2.85588465
H	4.34263752	5.81489098	2.51470477
C	5.63363412	5.99004405	4.18006127
C	7.02835064	6.28307468	4.629733
C	8.03049574	6.70914858	3.60542123
C	7.68008276	6.66954473	2.29908464
H	8.39852307	6.93886095	1.52187435
C	4.55204892	5.70233244	5.2312967
C	4.43350987	6.88725484	6.20756486
H	5.3732605	7.06044447	6.74730109
H	3.64179281	6.68977913	6.94848254
H	4.16668961	7.81203484	5.67054573
C	4.91224993	4.41567564	5.99879007
H	4.98213001	3.55672488	5.31168626
H	4.13176355	4.1865722	6.74262836
H	5.87107703	4.51668853	6.52320448
C	3.17936283	5.49405164	4.57780404
H	2.85009838	6.38287625	4.01763193
H	2.42719981	5.29997326	5.3581657
H	3.16671085	4.63409866	3.89008154
C	9.42089309	7.16224817	4.07443053
C	10.2921481	7.61440831	2.89516544
H	10.4774784	6.8022562	2.17463314
H	11.2712087	7.9489427	3.27230477
H	9.84344099	8.45999826	2.35021421
C	9.27601421	8.35916867	5.03360974
H	10.2701117	8.71235972	5.35236718
H	8.70393126	8.09117572	5.93104563
H	8.76473634	9.1996728	4.53622978
C	10.146154	6.00059069	4.78025237
H	10.2676199	5.14326515	4.09834353
H	9.59395041	5.6616363	5.66565597
H	11.151961	6.31969608	5.09911211
S	1.99340172	3.2286643	0.5284529
O	1.18211428	2.45721953	-0.38502133

O	2.67629582	2.63267746	1.65688203
C	0.83203684	4.48774089	1.27045048
F	1.52696859	5.51011662	1.74666689
H	1.90274125	4.13519718	-2.12332118

1s-Tf

O	1.91571531	9.38179551	-2.07184054
O	3.88336108	8.80900463	-2.97777809
O	7.26170071	6.34180261	5.85992756
F	-0.12036013	3.0652794	1.50325463
C	5.94993485	6.27967409	0.56385044
H	6.72449898	6.64532886	-0.12294379
C	3.01570286	8.79720236	-2.07656227
C	3.39589457	7.98636939	-0.77742539
C	3.33774492	5.86795294	-2.14787384
H	4.48329488	4.05335559	1.05470727
C	2.76578097	4.72609012	-1.77133901
H	3.16776128	6.23469344	-3.16227623
N	3.03391992	4.19125966	-0.47880643
C	4.11594781	4.70646053	0.26212079
F	0.14679839	4.73050574	0.15581296
C	4.70806969	5.86305527	-0.08889781
C	4.18031948	6.71328794	-1.23050745
H	5.02741966	7.10928402	-1.81252463
C	2.15544457	7.62637649	0.03534298
H	1.45296996	7.01141734	-0.54636881
H	2.41508607	7.0742963	0.9553612
H	1.61750609	8.54046745	0.32121119
C	4.29465806	8.9180888	0.04875394
H	4.51115619	8.51749496	1.05187414
H	3.79070357	9.8885325	0.18700476
H	5.2509047	9.11095508	-0.46273164
C	6.24146509	6.29708162	1.8940593
C	5.26090129	6.01456399	2.92601141
H	4.24164676	5.81506269	2.597783
C	5.55263993	6.0372872	4.24623322
C	6.9496375	6.35721432	4.6725087
C	7.95029668	6.72449634	3.62407165
C	7.57403533	6.68378239	2.32525952
H	8.2813813	6.93539163	1.53224146
C	4.48422906	5.76991757	5.31647775

C	4.34446037	6.99985061	6.23278446
H	5.28212324	7.2164065	6.76054598
H	3.55773647	6.82620229	6.98506152
H	4.06023344	7.89153437	5.65053435
C	4.87253347	4.53051347	6.14508148
H	4.95250775	3.63893732	5.50213927
H	4.10066469	4.32617762	6.90501888
H	5.83238189	4.67323007	6.65756334
C	3.11493771	5.49763606	4.67905483
H	2.75886625	6.35012505	4.07920376
H	2.37031817	5.3189809	5.47024251
H	3.1279544	4.60675097	4.03202931
C	9.3641387	7.13831777	4.0591345
C	10.2429117	7.49103652	2.85126101
H	10.374361	6.63719648	2.16791851
H	11.2435263	7.7881323	3.20214604
H	9.83476338	8.3342055	2.27210724
C	9.2859391	8.38577698	4.9601538
H	10.2991056	8.70647948	5.25262065
H	8.7112601	8.18871465	5.87409115
H	8.80872539	9.22420311	4.42683331
C	10.0439736	5.97915223	4.81229901
H	10.1042242	5.08102501	4.17607439
H	9.49520412	5.71693183	5.72554378
H	11.0722147	6.26008584	5.0931095
S	2.09805999	2.99041252	0.14452854
O	1.44128542	2.29092688	-0.93591942
O	2.82843426	2.3112706	1.19133379
C	0.7523606	3.92656912	1.01217786
F	1.2727953	4.63989254	1.99709821
H	2.12162837	4.11403661	-2.40314947

1s-Tf TS

O	-3.48607763	1.93030942	-4.60723811
O	-3.20407841	1.44981695	-2.43740372
O	2.37595465	-0.09807839	3.86291038
F	-6.49967672	-1.31933197	-0.83056863
C	0.40760474	0.07366733	-1.24727202
H	1.11575009	0.61329892	-1.88396229
C	-2.80198093	1.6330277	-3.60940573
C	-1.25098015	1.48277611	-3.81942816

C	-1.69373732	-0.97966324	-4.18901136
H	-1.94029448	-1.13009291	-0.34665172
C	-2.67974038	-1.63934596	-3.58414146
H	-1.56279835	-1.13369524	-5.26229843
N	-2.83928128	-1.53911772	-2.17946769
C	-1.82478327	-0.97096503	-1.41467221
F	-5.6185871	-0.32031593	-2.52419329
C	-0.77745325	-0.3327209	-1.98300017
C	-0.81270063	0.01034311	-3.4692914
H	0.21615459	-0.0982581	-3.85610673
C	-0.55765015	2.51079568	-2.91982468
H	-0.83412004	2.36815285	-1.86570641
H	0.5418531	2.47491869	-3.00896957
H	-0.87580728	3.52703804	-3.20599933
C	-0.86494933	1.7572527	-5.27401249
H	0.22571552	1.6664093	-5.4210463
H	-1.16873768	2.77284746	-5.56602175
H	-1.36629505	1.06874348	-5.96894113
C	0.85150316	-0.07264485	0.04410015
C	0.18909598	-0.78088302	1.12084859
H	-0.71648646	-1.33507954	0.89557096
C	0.65824272	-0.81086528	2.39213007
C	1.93203234	-0.10111603	2.71565791
C	2.6583164	0.58140391	1.61112432
C	2.11453424	0.56614849	0.37058945
H	2.62019154	1.06972754	-0.45600203
C	-0.07556232	-1.56647097	3.5115317
C	-0.47570868	-0.58377446	4.62983251
H	0.40289693	-0.09999209	5.07455113
H	-1.01673394	-1.1189776	5.42732997
H	-1.14581792	0.20100001	4.24281538
C	0.83478733	-2.67149739	4.08009038
H	1.10761065	-3.39618984	3.29581584
H	0.30886093	-3.22239794	4.87688397
H	1.75790954	-2.25432551	4.50237302
C	-1.3599152	-2.2333556	2.99906637
H	-2.07909973	-1.50073513	2.60024728
H	-1.85543143	-2.75718285	3.83128351
H	-1.16288331	-2.97938251	2.21304303
C	3.99192902	1.28030055	1.915375
C	4.5920313	1.92178483	0.65728735

H	4.80001261	1.17939319	-0.12944014
H	5.5468913	2.40700977	0.91302177
H	3.93439449	2.69593954	0.23111536
C	3.77309946	2.3972114	2.95335421
H	4.72283142	2.91862036	3.15615998
H	3.38999957	1.99476863	3.89968435
H	3.05413497	3.14422493	2.57892717
C	5.00730729	0.25291	2.45192564
H	5.1832505	-0.54579874	1.71274584
H	4.65323666	-0.2085358	3.38265981
H	5.97473469	0.74176755	2.65289278
S	-4.15656747	-2.20167455	-1.43257417
O	-4.74685175	-3.17628322	-2.32245669
O	-3.81158635	-2.50710538	-0.0611851
C	-5.3787344	-0.8088349	-1.32769863
F	-4.93242487	0.12178394	-0.51435502
H	-3.36933451	-2.31870382	-4.08459863

1t

C	4.7584066	7.62436945	-3.56050132
C	5.07554827	5.98629647	-0.3704954
O	3.72957666	8.71973056	-3.90373444
O	2.89466551	8.06843001	-1.82799951
O	0.1810247	1.92607705	-5.31613899
F	9.02695507	0.72131021	-0.70914515
C	4.90126657	2.94680048	-2.46929137
H	5.66729576	2.2146305	-2.74343023
C	3.62198742	8.06143773	-2.77335732
C	6.52795556	6.07709153	-0.36167733
H	8.35931879	4.97596246	-0.6074481
C	7.26811347	4.99009403	-0.63168501
H	7.02728496	7.02095678	-0.13377325
N	6.62174686	3.76482676	-0.96385578
C	5.27636623	3.83436951	-1.51076248
F	9.36425029	2.69958096	-1.5048043
C	4.48069031	4.90421472	-0.91817032
H	4.47835177	6.80207167	0.04070372
C	6.12851545	8.17366416	-3.28529288
H	6.10711507	8.99288427	-2.55362495
H	6.76763	7.36833853	-2.89520446
H	6.57522312	8.538038	-4.2234705

C	4.66638678	6.41820987	-4.4476124
H	5.12347198	5.55729804	-3.93650205
H	5.21598242	6.59356854	-5.38493417
H	3.62418454	6.16614746	-4.68087037
C	3.64697046	2.75525726	-3.17645539
C	3.46890992	1.53367021	-3.86656536
H	4.28899783	0.81253837	-3.81083386
C	2.32788322	1.21137783	-4.57916506
C	1.23346154	2.17545537	-4.6640187
C	1.42397604	3.45146153	-3.97552231
C	2.59204122	3.69311926	-3.27150219
H	2.71913653	4.6683813	-2.80341538
C	2.18534258	-0.14372735	-5.29617139
C	2.0137469	0.08292185	-6.81017653
H	1.14930822	0.73196816	-7.00225242
H	1.86174907	-0.87579911	-7.33536593
H	2.91157952	0.55816597	-7.23929025
C	0.95954719	-0.8959994	-4.74400808
H	1.08209217	-1.10544343	-3.66823226
H	0.82677996	-1.86230608	-5.26029205
H	0.05177038	-0.29388649	-4.87940481
C	3.4126389	-1.04303322	-5.09563747
H	4.3325902	-0.59193521	-5.50143803
H	3.25903906	-2.00016356	-5.62007204
H	3.59063089	-1.27693896	-4.03356013
C	0.31884487	4.52054798	-4.07499308
C	0.66989969	5.79805502	-3.30181487
H	0.83539909	5.60857678	-2.22954448
H	-0.15571256	6.52345128	-3.38426837
H	1.57142903	6.28690315	-3.70132408
C	0.10935621	4.91040278	-5.55061309
H	-0.69696262	5.65721995	-5.65047063
H	-0.15201	4.02504962	-6.14482009
H	1.02772673	5.35338206	-5.97158891
C	-0.99643424	3.96892447	-3.49289247
H	-0.88156654	3.73478873	-2.4213583
H	-1.28983531	3.05275126	-4.02132229
H	-1.80672412	4.71212802	-3.5876281
S	7.14864142	2.42192751	-0.13774032
O	7.90235993	2.81880911	1.03589985
O	6.11815573	1.40919223	-0.08779126

C	8.45144944	1.76385289	-1.28433874
F	7.92755	1.39125313	-2.43994003
H	3.39209331	4.82479096	-0.94858369

1t-Tf

C	4.64881024	6.94269528	-1.17827859
C	5.16522542	5.64010318	-0.48373188
O	2.57546882	6.89732676	-2.42370051
O	2.50040789	6.52495996	-0.21740128
O	0.11995877	2.04210323	-4.93717547
F	8.28526892	4.22921882	-5.48234315
C	4.87746628	2.28522226	-2.38178696
H	5.33873847	1.35255947	-2.04016549
C	3.08759139	6.77803708	-1.28848153
C	6.65628697	5.5584637	-0.31547313
H	8.50382921	4.61786184	-0.8465412
C	7.42236025	4.68384472	-0.96630215
H	7.15044036	6.22885564	0.39242518
N	6.86487544	3.71445566	-1.85755457
C	5.44121982	3.53388896	-1.85252231
F	7.98572321	5.71693808	-3.95034296
C	4.67228529	4.4130029	-1.18969343
H	4.68163414	5.6553499	0.50695096
C	4.93644364	8.14987893	-0.27906817
H	4.4593195	8.02097765	0.70376124
H	6.01688527	8.3103947	-0.12762222
H	4.52863068	9.06943467	-0.73030192
C	5.29674524	7.13827491	-2.54327884
H	6.38363877	7.29867676	-2.45488154
H	4.858261	8.01027619	-3.04914175
H	5.13157104	6.27083072	-3.19900474
C	3.77432961	2.22164472	-3.16972233
C	3.1277772	0.93710552	-3.39693445
H	3.62821762	0.06263179	-2.97576026
C	1.94179473	0.81847518	-4.03355962
C	1.28043021	2.06306413	-4.54294872
C	2.0747282	3.32834452	-4.56731815
C	3.21365968	3.38406476	-3.84332174
H	3.77881188	4.3141312	-3.78394239
C	1.2275983	-0.52842545	-4.20928742
C	0.91356065	-0.76553778	-5.69822189

H	0.23478942	0.00090247	-6.09420426
H	0.43456987	-1.74916997	-5.83061858
H	1.83567351	-0.75816854	-6.30186925
C	-0.07562108	-0.53636869	-3.38686666
H	0.13565438	-0.37733542	-2.31698046
H	-0.58151348	-1.51065471	-3.48803996
H	-0.76561607	0.24850116	-3.72135669
C	2.10005658	-1.69315035	-3.72191103
H	3.05734916	-1.74953967	-4.2640306
H	1.56861302	-2.6429472	-3.88887653
H	2.31912829	-1.62824734	-2.64459252
C	1.55659933	4.53075796	-5.36880244
C	2.63996919	5.61330097	-5.4927697
H	2.8803355	6.06055702	-4.51505919
H	2.26880087	6.42378117	-6.14047673
H	3.56210257	5.21913288	-5.95111665
C	1.16292922	4.09040971	-6.79151184
H	0.88260704	4.97261729	-7.38918146
H	0.31442758	3.39476607	-6.78792606
H	2.00846231	3.59951117	-7.30173104
C	0.34969359	5.15263743	-4.64105986
H	0.67472663	5.57751139	-3.67795599
H	-0.44244085	4.41083677	-4.46769246
H	-0.07532907	5.97368733	-5.24264066
S	7.79904187	3.2327814	-3.12876785
O	9.18674578	3.32455386	-2.73326387
O	7.24514369	2.03986691	-3.72666426
C	7.57225037	4.55178368	-4.41713729
F	6.29496141	4.63409018	-4.7532088
H	3.60705926	4.18795946	-1.10260595

4s

O	3.18599116	10.0550697	-0.08706619
O	4.69937516	8.68209602	0.67211452
O	7.13243321	5.98677796	5.87387727
F	1.27066183	2.29818915	1.80300413
C	5.51817826	7.47261218	0.68124724
H	6.41147527	7.72978581	0.08440269
C	3.67808748	8.9538752	-0.14257023
C	3.10729219	7.86639794	-1.06691611
C	3.70302514	5.60094622	-2.12644519

H	5.08159614	4.83297735	1.4228495
C	3.60903355	4.3771711	-1.6055573
H	3.42624018	5.72858838	-3.17544865
N	4.01823801	4.11645163	-0.27389618
C	4.67366094	5.13564472	0.45827692
F	1.04816069	3.64903584	0.13466864
C	4.79472335	6.36823569	-0.03732699
C	4.19494802	6.79173995	-1.34887653
H	4.98065328	7.2994818	-1.9405848
C	2.65224716	8.52876018	-2.36904004
H	3.50238685	8.97879977	-2.90530311
H	2.17886199	7.78948529	-3.03218
H	1.92101142	9.3219066	-2.16375483
C	1.89891869	7.26089337	-0.3338152
H	1.41420565	6.49995881	-0.96408427
H	1.15887136	8.0431775	-0.11061209
H	2.18862108	6.78321619	0.6143246
C	5.94369606	7.17334172	2.08602612
C	5.00138955	6.92400315	3.09354771
H	3.94354643	7.02943433	2.83793879
C	5.36106236	6.53644807	4.37711515
C	6.77081955	6.37046596	4.71847758
C	7.73970964	6.66021959	3.67161716
C	7.29777877	7.04157305	2.40770847
H	8.02637422	7.23765158	1.61538942
C	4.29170813	6.22363818	5.439316
C	4.50153474	7.1101329	6.68060714
H	5.51668757	6.97092091	7.07417152
H	3.77154289	6.86071421	7.46999285
H	4.36727759	8.17501717	6.42604982
C	4.39722911	4.73802113	5.83448374
H	4.20466131	4.09099025	4.96174247
H	3.65768599	4.48122453	6.61248987
H	5.40461938	4.52084576	6.21449795
C	2.86625834	6.46996885	4.92670669
H	2.70605564	7.51795487	4.62560083
H	2.14073575	6.24322251	5.72507396
H	2.61480046	5.82975743	4.06714578
C	9.24124269	6.50842917	3.97839962
C	10.1260544	6.87223912	2.77765333
H	9.93764261	6.22328543	1.90735911

H	11.1877784	6.7532289	3.04906186
H	9.98510705	7.91707489	2.45627043
C	9.63400969	7.43120793	5.1478885
H	10.698532	7.3038432	5.41053702
H	9.01903657	7.20039951	6.02751515
H	9.47974173	8.49027575	4.88103053
C	9.53897657	5.04494804	4.35515802
H	9.30617301	4.37143155	3.51305679
H	8.92494203	4.74957566	5.21652519
H	10.6044571	4.90927062	4.60978453
S	3.48564081	2.76551132	0.50443557
O	3.21698127	1.73642471	-0.47331807
O	4.29724451	2.53980651	1.6776175
C	1.82601721	3.29850486	1.14556672
F	1.98256362	4.33375708	1.95519295
H	3.25677218	3.50741428	-2.1611386

4s'

O	1.71384781	8.50728671	-1.50266917
O	2.60795242	7.51396906	0.22669467
O	7.47467494	6.33780735	5.96467546
F	-1.22896457	5.49808972	1.83989492
C	5.85432821	6.16689954	0.59162685
H	6.67499701	6.07642168	-0.13440021
C	2.55707825	7.74914306	-1.10436325
C	3.62261966	7.13885033	-2.01905851
C	3.32177187	4.69155238	-1.34525699
H	3.54332918	6.78020962	1.86072578
C	2.53923394	4.41989258	-0.29729684
H	3.29058201	4.01085736	-2.19875349
N	2.53109739	5.25510828	0.85163972
C	3.36278694	6.45497396	0.83142801
F	-0.23176764	5.6152844	-0.07186649
C	4.61578299	6.17496571	0.06642213
C	4.26107445	5.88262888	-1.36540914
H	5.16179105	5.6229391	-1.94169093
C	4.69575659	8.22729784	-2.20264484
H	5.17685782	8.4934974	-1.24997609
H	5.47451799	7.86580425	-2.89175869
H	4.25163714	9.13763581	-2.63163095
C	2.98505804	6.8130712	-3.37132991

H	3.73685437	6.36762616	-4.04114921
H	2.5964058	7.72486992	-3.84489508
H	2.15042694	6.10409032	-3.26832668
C	6.24447319	6.22827273	2.00273157
C	5.55630869	5.51616078	3.00351673
H	4.70487856	4.90232182	2.69889914
C	5.95114017	5.50686405	4.3339805
C	7.10315016	6.29700589	4.75437353
C	7.82458026	7.01954842	3.70967429
C	7.39316686	6.94194192	2.39386784
H	7.94591904	7.46919556	1.61133041
C	5.20676941	4.65278883	5.37714168
C	4.61413102	5.56704872	6.46555373
H	5.41209955	6.15871803	6.93356837
H	4.1047631	4.97388989	7.2447584
H	3.87147097	6.2589856	6.03393202
C	6.18573662	3.65076236	6.01775061
H	6.58050329	2.95238006	5.26064719
H	5.67877291	3.0512543	6.79326295
H	7.02956119	4.18308138	6.475509
C	4.05492496	3.84417368	4.76415741
H	3.28237408	4.48667152	4.3170747
H	3.56622894	3.2456431	5.55042587
H	4.40199595	3.1425886	3.98789169
C	9.06500017	7.84727221	4.09665682
C	9.70082327	8.55146745	2.89027386
H	10.0498491	7.83867465	2.12586688
H	10.5773841	9.13277157	3.21970371
H	9.00571562	9.25514351	2.40425098
C	8.6677541	8.93273836	5.11517279
H	9.54895224	9.51914788	5.42760534
H	8.21430852	8.47045762	6.00148344
H	7.93871105	9.6351519	4.67749331
C	10.1299213	6.92347328	4.71780671
H	10.4616591	6.16385851	3.99023652
H	9.71397116	6.40960265	5.59434544
H	11.0186865	7.49987122	5.02759044
S	1.33899386	5.10658823	1.97493936
O	0.97273875	3.71207208	2.08677976
O	1.68030521	5.92422491	3.11883631
C	-0.15015257	5.93298908	1.21198188

F	-0.06642005	7.24000576	1.33796511
H	1.87936911	3.55258728	-0.24878335

4t

C	5.02152469	7.08833182	-1.29614538
C	4.77081154	5.64867576	-0.77001429
O	3.64072789	9.0189067	-0.68642355
O	3.66347681	7.22202059	0.6235282
O	6.47271212	5.32323695	6.92270442
F	-0.97715962	6.70027302	0.11148643
C	3.34930123	4.28985594	2.39410975
H	2.66981436	3.43852282	2.5135093
C	4.03619827	7.90669662	-0.46559346
C	3.79570479	4.85136634	-1.58721188
H	1.93692963	3.81022165	-1.73285985
C	2.64438376	4.36974544	-1.11916097
H	4.05287681	4.63165828	-2.62579086
N	2.27981035	4.52244321	0.24543746
C	3.30122182	4.90180304	1.18742848
F	0.89112132	6.87125333	-0.95213692
C	4.27181074	5.9095098	0.66693962
H	5.72520604	5.10050282	-0.72997912
C	6.43420682	7.56093751	-0.91138224
H	6.64536019	7.40479552	0.15797141
H	7.1852547	7.00347364	-1.49125036
H	6.55562938	8.63287747	-1.12814846
C	4.77225448	7.3122009	-2.78387183
H	5.45221209	6.69701122	-3.39277365
H	4.94965294	8.36850907	-3.03367987
H	3.73695029	7.07049187	-3.06560729
C	4.15148943	4.60773378	3.56153402
C	4.39409651	3.59896633	4.51803865
H	3.95402442	2.61613037	4.32813124
C	5.15921369	3.79585962	5.65409544
C	5.75240045	5.10791952	5.90787529
C	5.44963193	6.1682766	4.9467647
C	4.67701283	5.88991659	3.83369268
H	4.42200289	6.70432332	3.15381579
C	5.4013437	2.65786789	6.66295767
C	4.86518254	3.06802042	8.0474901
H	5.34994757	3.99331806	8.38452274

H	5.0533662	2.2751636	8.79178171
H	3.77631469	3.2388651	8.00893825
C	6.90982344	2.36123095	6.75951202
H	7.30214329	2.00796166	5.79099612
H	7.10764918	1.57378151	7.50695318
H	7.45692906	3.26859621	7.04724668
C	4.69409718	1.35705488	6.25784041
H	3.6011922	1.47987624	6.18810035
H	4.88866585	0.57888778	7.0139125
H	5.05309107	0.96972132	5.29064211
C	6.00525612	7.58315808	5.19187389
C	5.575039	8.57656783	4.10312487
H	5.93041377	8.28279538	3.10228684
H	5.99776995	9.57073537	4.32092585
H	4.48035396	8.69081065	4.04900139
C	5.49955433	8.12160737	6.5432902
H	5.91409704	9.12408435	6.74568512
H	5.79480391	7.44473397	7.35540735
H	4.40008302	8.20791247	6.54232454
C	7.54484164	7.52980176	5.19894215
H	7.92744811	7.18152833	4.22466159
H	7.89457455	6.83669531	5.97529059
H	7.97334412	8.52847837	5.39090181
S	0.67785224	4.7424698	0.58021574
O	-0.09282007	4.00741848	-0.40171216
O	0.43727058	4.60767666	1.99822611
C	0.33310614	6.53432815	0.20298964
F	0.80468907	7.30353676	1.16303436
H	5.12880699	5.96098584	1.34919138

4t’

C	4.9670549	6.77630393	-0.66352168
C	5.1538453	5.20507488	-0.80940053
O	2.81477788	7.85204128	-0.48373341
O	3.48328916	6.96230763	1.41447679
O	6.54522853	5.29880259	6.72492889
F	-1.23081287	4.97509816	0.52090406
C	3.33217522	5.67273037	2.08778844
H	2.27752802	5.6440393	2.39160878
C	3.64378034	7.18254423	0.07339714
C	4.16293008	4.57391275	-1.75082126

H	2.18810664	3.76815438	-1.95259406
C	2.94815529	4.22993168	-1.32302879
H	4.42441526	4.39402121	-2.79492983
N	2.58848798	4.43627766	0.03380758
C	3.62844855	4.66850318	0.99478489
F	0.15108546	5.81299886	-0.91128145
C	4.90299822	4.70833947	0.5763647
H	6.18326047	5.03795132	-1.15797553
C	6.13742996	7.3722522	0.13721844
H	6.23062809	6.9421746	1.14325371
H	7.08229036	7.19745145	-0.39973372
H	6.00986453	8.45983718	0.25169712
C	4.94242877	7.40227834	-2.05952052
H	5.85327906	7.1190981	-2.60964813
H	4.908457	8.49921686	-1.99518726
H	4.06927165	7.07716012	-2.64158536
C	4.21782538	5.5955021	3.30628149
C	4.2110671	4.42975176	4.08196605
H	3.57507855	3.60292005	3.75225099
C	4.97477259	4.29416073	5.23656717
C	5.82364457	5.39420195	5.68126475
C	5.80268786	6.60283481	4.87325848
C	5.00931834	6.664417	3.72862151
H	4.98961547	7.57441975	3.12762533
C	4.94573426	2.99046028	6.05678303
C	4.47646428	3.29057955	7.49293652
H	5.13930344	4.034064	7.95475269
H	4.47877669	2.37384261	8.10778309
H	3.44817834	3.68976752	7.49198067
C	6.35695179	2.37423863	6.09364115
H	6.68784195	2.0966565	5.07866005
H	6.37398165	1.45982757	6.71176016
H	7.07005955	3.09972397	6.50740371
C	3.99140275	1.94415015	5.46415805
H	2.9493165	2.30114964	5.42893562
H	4.00478457	1.03484196	6.08730061
H	4.27891918	1.64507131	4.4432136
C	6.66937725	7.8042081	5.29558094
C	6.52130609	9.000584	4.34493088
H	6.82996743	8.75814294	3.31515231
H	7.15928368	9.83024101	4.69123928

H	5.4864884	9.37720606	4.3049498
C	6.25806479	8.27095643	6.70429486
H	6.8923908	9.10791523	7.04442567
H	6.35163925	7.4386784	7.4145312
H	5.21185782	8.62019368	6.70900197
C	8.15316788	7.39064044	5.30103563
H	8.48129997	7.10418078	4.28753858
H	8.29840853	6.53255373	5.97078853
H	8.79677549	8.22207157	5.63715948
S	1.10939653	3.85941015	0.53391171
O	0.60487888	2.9103258	-0.43429571
O	1.15240083	3.56747391	1.94866036
C	0.01947523	5.35014612	0.31894793
F	0.34317699	6.29190358	1.18336088
H	5.64805348	4.9250685	1.34498197

(1) Roiser, L.; Waser, M. Enantioselective Spirocyclopropanation of *Para* -Quinone Methides Using Ammonium Ylides. *Org. Lett.* **2017**, *19* (9), 2338–2341. https://doi.org/10.1021/acs.orglett.7b00869.

(2) Neese, F. Software Update: The ORCA Program System—Version 5.0. *Wiley Interdiscip Rev Comput Mol Sci* **2022**, *12* (5), e1606. https://doi.org/10.1002/WCMS.1606.

(3) Neese, F.; Wennmohs, F.; Becker, U.; Riplinger, C. The ORCA Quantum Chemistry Program Package. *J. Chem. Phys.* **2020**, *152* (22), 224108. https://doi.org/10.1063/5.0004608/1061982.

(4) Chai, J. Da; Head-Gordon, M. Systematic Optimization of Long-Range Corrected Hybrid Density Functionals. *J. Chem. Phys.* **2008**, *128* (8), 84106. https://doi.org/10.1063/1.2834918/71104.

(5) Caldeweyher, E.; Ehlert, S.; Hansen, A.; Neugebauer, H.; Spicher, S.; Bannwarth, C.; Grimme, S. A Generally Applicable Atomic-Charge Dependent London Dispersion Correction. *J. Chem. Phys.* **2019**, *150* (15), 154122. https://doi.org/10.1063/1.5090222/76314.

(6) Remya, K.; Suresh, C. H. Which Density Functional Is Close to CCSD Accuracy to Describe Geometry and Interaction Energy of Small Noncovalent Dimers? A Benchmark Study Using Gaussian09. *J. Comput. Chem.* **2013**, *34* (15), 1341–1353. https://doi.org/10.1002/JCC.23263.

(7) Weigend, F.; Ahlrichs, R. Balanced Basis Sets of Split Valence, Triple Zeta Valence and Quadruple Zeta Valence Quality for H to Rn: Design and Assessment of Accuracy. *Phys. Chem. Chem. Phys.* **2005**, *7* (18), 3297–3305. https://doi.org/10.1039/B508541A.

(8) Weigend, F. Accurate Coulomb-Fitting Basis Sets for H to Rn. *Phys. Chem. Chem. Phys.* **2006**, *8* (9), 1057–1065. https://doi.org/10.1039/B515623H.

(9) Neese, F. An Improvement of the Resolution of the Identity Approximation for the Formation of the Coulomb Matrix. *J. Comput. Chem.* **2003**, *24* (14), 1740–1747. https://doi.org/10.1002/JCC.10318.

(10) Neese, F.; Wennmohs, F.; Hansen, A.; Becker, U. Efficient, Approximate and Parallel Hartree–Fock and Hybrid DFT Calculations. A 'Chain-of-Spheres' Algorithm for the Hartree–Fock Exchange. *Chem. Phys.* **2009**, *356* (1–3), 98–109. https://doi.org/10.1016/J.CHEMPHYS.2008.10.036.

(11) Izsák, R.; Neese, F. An Overlap Fitted Chain of Spheres Exchange Method. *J. Chem. Phys.* **2011**, *135* (14), 144105. https://doi.org/10.1063/1.3646921/190211.

(12) Helmich-Paris, B.; de Souza, B.; Neese, F.; Izsák, R. An Improved Chain of Spheres for Exchange Algorithm. *J. Chem. Phys.* **2021**, *155* (10). https://doi.org/10.1063/5.0058766/1013244.

(13) Marenich, A. V.; Cramer, C. J.; Truhlar, D. G. Universal Solvation Model Based on Solute Electron Density and on a Continuum Model of the Solvent Defined by the Bulk Dielectric Constant and Atomic Surface Tensions. *J. Phys. Chem. B* **2009**, *113* (18), 6378–6396. https://doi.org/10.1021/JP810292N/SUPPL_FILE/JP810292N_SI_003.PDF.

(14) *README for AIMAll*. https://aim.tkgristmill.com/readme.html (accessed 2024-08-20).
